# Discovery of practical production processes for arylsulfur pentafluorides and their higher homologues, bis- and tris(sulfur pentafluorides): Beginning of a new era of “super-trifluoromethyl” arene chemistry and its industry

**DOI:** 10.3762/bjoc.8.53

**Published:** 2012-03-29

**Authors:** Teruo Umemoto, Lloyd M Garrick, Norimichi Saito

**Affiliations:** 1 IM&T Research, Inc./Ube America Inc., 6860 N. Broadway, Suite B, Denver, Colorado 80221, USA

**Keywords:** arylsulfur chlorotetrafluoride, arylsulfur pentafluoride, pentafluorosulfanyl, sulfur pentafluoride, super-trifluoromethyl

## Abstract

Various arylsulfur pentafluorides, ArSF_5_, have long been desired in both academic and industrial areas, and ArSF_5_ compounds have attracted considerable interest in many areas such as medicines, agrochemicals, and other new materials, since the highly stable SF_5_ group is considered a “super-trifluoromethyl group” due to its significantly higher electronegativity and lipophilicity. This article describes the first practical method for the production of various arylsulfur pentafluorides and their higher homologues, bis- and tris(sulfur pentafluorides), from the corresponding diaryl disulfides or aryl thiols. The method consists of two steps: (Step 1) treatment of a diaryl disulfide or an aryl thiol with chlorine in the presence of an alkali metal fluoride, and (step 2) treatment of the resulting arylsulfur chlorotetrafluoride with a fluoride source, such as ZnF_2_, HF, and Sb(III/V) fluorides. The intermediate arylsulfur chlorotetrafluorides were isolated by distillation or recrystallization and characterized. The aspects of these new reactions are revealed and reaction mechanisms are discussed. As the method offers considerable improvement over previous methods in cost, yield, practicality, applicability, and large-scale production, the new processes described here can be employed as the first practical methods for the economical production of various arylsulfur pentafluorides and their higher homologues, which could then open up a new era of “super-trifluoromethyl” arene chemistry and its applications in many areas.

## Introduction

Pentafluorosulfanyl (SF_5_) is considered a “super-trifluoromethyl group” as SF_5_ has the peculiarity of fluorine beyond a trifluoromethyl (CF_3_) group [1]. Arylsulfur pentafluorides (ArSF_5_) are very thermally and chemically stable [2]. Pioneering work by Sheppard half a century ago on the synthesis and properties of arylsulfur pentafluorides revealed that the SF_5_ group has absolutely unique properties [2–3]. SF_5_ is more electronegative (Hammet constants σ_I_: +0.55 for SF_5_; +0.39 for CF_3_) [2] and more lipophilic than CF_3_ (Hansch hydrophobicity constants π: 1.51 for SF_5_; 1.09 for CF_3_) [4]. The high electronegativity results in high polarity in molecules. Thus, it is of significant note that there is no functional group other than SF_5_ that has both high electronegativity and high lipophilicity, because the two natures are generally in conflict. In addition, SF_5_ has high hydrolytic stability, which equals or exceeds CF_3_ [2]. These extraordinary properties render SF_5_ compounds highly attractive particularly in medicinal [5–14], agrochemical [15–18], and new material [19–23] chemistry and industry. However, there have been no practical, economical methods for the production of arylsulfur pentafluorides. In comparison, trifluoromethyl arenes (ArCF_3_) have grown into a significantly large field in chemistry and industry since their practical two-step production method was developed in the 1930s through to the 1940s. The first step was chlorination of ArCH_3_ to ArCCl_3_ with Cl_2_ and the second step was its conversion to ArCF_3_ with HF or SbF_3_ [24–26]. A large number of trifluoromethyl arenes are currently produced on a large scale and used in many areas such as medicines, agrochemicals, dyes, materials for electronics, and others [27–30]. Accordingly, many chemists have long desired to have easy access to the “super-trifluoromethyl” arenes; however, there have been no economical methods for their production until now.

In 1961, Sheppard first reported the synthesis of phenylsulfur pentafluoride by stepwise fluorination of diphenyl disulfide with expensive silver difluorides (AgF_2_) in a fluorocarbon solvent [3]. However, the yield was only 9%. Since then, many substituted phenylsulfur pentafluorides have been prepared by this method, but still with very low yields [3,20,31–32]. In 2000, a new method using molecular fluorine (F_2_) was reported [33]. Thus, bis(*p*- or *m*-nitrophenyl) disulfide was treated with F_2_ diluted with nitrogen (F_2_:N_2_ = 1:9 v/v) at low temperature in acetonitrile to give the nitrophenylsulfur pentafluoride in ca. 40% yield. However, in addition to the low yields, this method requires F_2_, which is a highly toxic, corrosive and explosive gas, and it applies only for the case of electron-deficient nitrophenylsulfur pentafluorides, due to the extremely high reactivity of F_2_. These factors significantly limited the scope and application of this method. Another method reported used expensive xenon difluoride to fluorinate diphenyl disulfide giving phenylsulfur pentafluoride, but its yield was only 25% [34].

Multiple-step methods have previously been developed for the preparation of arylsulfur pentafluorides. In 1964, it was reported that the reaction of sparsely available and toxic gaseous SF_5_Cl with acetylene, followed by bromination, dehydrobromination, and reduction with zinc, giving pentafluorosulfanylacetylene (HC≡CSF_5_), which was then reacted with butadiene, followed by an aromatization reaction at very high temperature, gave phenylsulfur pentafluoride [35]. Recently, phenylsulfur pentafluoride was prepared by reaction of 1,4-bis(acetoxy)-2-cyclohexene with SF_5_Br under 250 W sunlamp irradiation, followed by dehydrobromination and then aromatization reactions [36]. A triethylborane-catalyzed reaction of 4,5-dichloro-1-cyclohexene with SF_5_Cl followed by dehydrochlorination has also been reported [37]. The multistep method has recently been extended to the preparation of 2-naphthylsulfur pentafluoride and heteroarylsulfur pentafluorides [22,38–41].

5-Nitrophenyl-1,3-bis(sulfur pentafluoride) was prepared by reaction of the corresponding polymeric disulfide with AgF_2_ in 12% yield [3]. Two isomers of phenyl tris(sulfur pentafluorides) were synthesized by many steps starting from the reaction of SF_5_Cl with acetylene via HC≡CSF_5_ [42]. Complex Co(CO)_4_(HC≡CSF_5_) derived from HC≡CSF_5_ and Co_2_(CO)_8_ was decomposed in the presence of Br_2_ to give phenyl-1,2,4-tris(sulfur pentafluoride). Photoreaction of HC≡CSF_5_ in the presence of SF_5_Cl gave phenyl-1,3,5-tris(sulfur pentafluoride), but in low yield (19%) [42].

All of the previous methods described above suffer from multiple drawbacks of low yields, the necessity of costly and dangerous fluoro-reagents, and the quite limited scope and applicability. In response to these, we now report practical, inexpensive, and widely applicable methods suitable for the large-scale production of arylsulfur pentafluorides and their higher homologues, which have the potential to open up a new era of sulfur pentafluoride chemistry and its industry. These new methods have been described in patents and patent applications [43–48].

## Results and Discussion

Recently we synthesized various arylsulfur trifluorides (ArSF_3_) by treatment of diaryl disulfides with chlorine in the presence of potassium [49–50] or cesium fluoride [50] and thus discovered 4-*tert*-butyl-2,6-dimethylphenylsulfur trifluoride (Fluolead reagent) as an excellent fluorinating agent with high thermal stability, ease of handling, and wide applicability [50–51]. During the research, we unexpectedly discovered that an arylsulfur chlorotetrafluoride (ArSF_4_Cl) is formed when a diaryl disulfide is treated with an excess of chlorine in the presence of an excess of the alkaline metal fluoride. Janzen et al. reported that *cis*- and *trans*-phenylsulfur chlorotetrafluoride and its *p*-methyl- and *p*-nitro-derivatives were formed by reaction of diaryl disulfide with XeF_2_ and tetraethylammonium chloride [52]. However, the physical properties of the arylsulfur chlorotetrafluorides were not determined since they were not isolated, presumably because the chlorotetrafluorides were considered to be unstable. Instead, their chemical structures were assigned by ^19^F- and ^13^C NMR spectroscopy of the reaction solution.

**Scheme 1:**
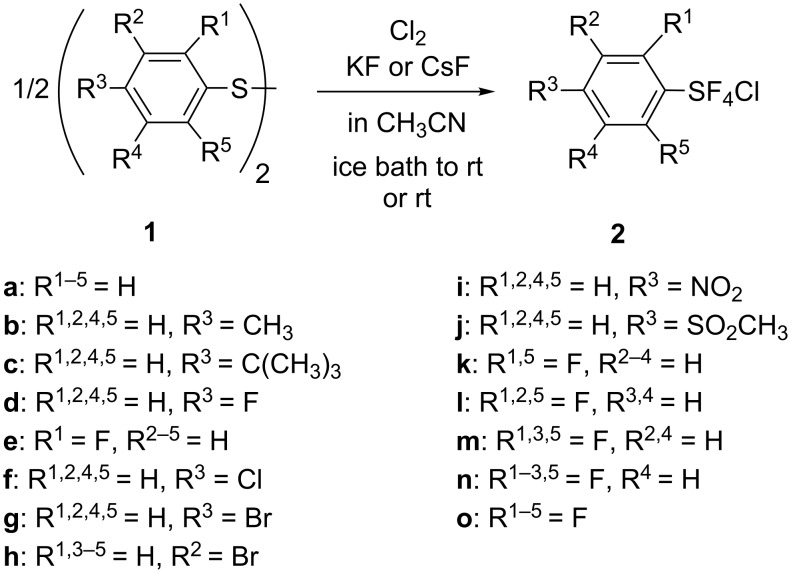
Preparation of ArSF_4_Cl **2**.

Arylsulfur chlorotetrafluorides were prepared in high yield by the reactions of diaryl disulfides with an excess amount of chlorine (ca. 7 mol or more per mole of the disulfide) in the presence of an excess amount of potassium or cesium fluoride (ca. 16 mol or more per mole of the disulfide) in dry acetonitrile at ice-bath temperature to room temperature. Although conventionally dried and powdered potassium fluoride can be used satisfactorily, spray-dried potassium fluoride, having a large surface area, is preferable. The normal dry potassium fluoride must be used in greater quantities than the spray-dried potassium fluoride. When the reaction is not taken to completion, the distilled product (ArSF_4_Cl) is contaminated with its intermediate ArSF_3_. By this method, many arylsulfur chlorotetrafluorides **2a–o** having different substituents on the aromatic ring were prepared, as summarized in Scheme 1 and Table 1. The products were *trans* isomers, except for in the case of polyfluorinated arylsulfur chlorotetrafluorides, which formed a mixture of *trans* and *cis* isomers. The products were distilled under reduced pressure or crystallized and then characterized.

**Table 1:** Preparation of arylsulfur chlorotetrafluorides **2a–o**.

run^a^	ArSSAr or ArSH (mmol)^b^	Cl_2_^c^	MF^d^	ArSF_4_Cl^e^	conditions^f^	yield (%)^g^

1	**1a** (150)	≈8	KF (16)	**2a** (*t*)	ice bath, 9.5 h	88
2	C_6_H_5_SH (91)	4.9	KF (9)	**2a** (*t*)	6–10 °C, 6.5 h	83
3	**1b** (500)	7.7	KF (16)	**2b** (*t*)	ice bath, 10.5 h	73
4	*p*-(*t*-Bu)C_6_H_4_SH (60)	7.5	CsF (10)	**2c** (*t*)	5–10 °C, 3.5 h to rt, 24 h	84
5	**1d** (39)	7.2	KF (16)	**2d** (*t*)	ice bath, 2.5 h to rt, o.n.	67
6	**1e** (39)	8	KF (16)	**2e** (*t*)	ice bath, 2.5 h to rt, o.n.	80
7	**1f** (87)	6.6	KF (17)	**2f** (*t*)	5–8 °C, 3.5 h	88
8	**1g** (100)	7.2	KF (16)	**2g** (*t*)	ice bath, 4.5 h to rt, o.n.	77
9	**1h** (127)	6.9	KF (15.7)	**2h** (*t*)	ice bath, 5.5 h to rt, o.n.	86
10	**1i** (100)	7.2	KF (16)	**2i** (*t*)	ice bath, 4.5 h to rt, o.n.	60
11	**1j** (26)	27	KF (40)	**2j** (*t*)	rt, 3 d	97^h^
12	**1k** (100)	10	CsF (18)	**2k** (*t*/*c* = 92/8)	ice bath, 5 h to rt, o.n.	82
13	**1l** (130)	16	KF (22)	**2l** (*t*/*c* = 89/11)	ice bath, 6 h to rt, o.n.	80
14	**1m** (77)	16	KF (24)	**2m** (*t*/*c* = 96/4)	ice bath, 6 h to rt, o.n.	87
15	**1n** (70)	15	KF (19)	**2n** (*t*/*c* = 86/14)	ice bath, 7.5 h to rt, o.n.	83
16	**1o** (65)	15	KF (22)	**2o** (*t*/*c* = 60/40)	ice bath, 5 h to rt, o.n.	86

^a^The experimental procedure is described in the Supporting Information File 1. ^b^The number in parentheses is the amount (mmol) of ArSSAr or ArSH used. ^c^Molar ratio of Cl_2_ per mole of ArSSAr or ArSH. ^d^The number in parentheses is molar ratio of MF per mole of ArSSAr or ArSH. ^e^*t* = *trans*-isomer, *c* = *cis*-isomer. The *t*/*c* ratio was determined by ^19^F NMR of the reaction mixture before post-treatment. ^f^rt = room temperature, o.n. = overnight. ^g^Isolated yields. ^h^Crude product.

Arylsulfur chlorotetrafluorides having an electron-donating alkyl group, such as methyl or *tert*-butyl, or an electron-withdrawing substituent, such as a halogen atom, a nitro-, or a methanesulfonyl group, were prepared in good to high yields from the corresponding diaryl disulfides. 2-Fluoro and 2,6-difluorophenylsulfur chlorotetrafluoride were formed in high yields due to the small steric effect of fluorine atom(s). However, bis(2-bromophenyl) disulfide gave a 11:1 mixture of 2-bromophenylsulfur trifluoride and chlorotetrafluoride. 2-Bromophenylsulfur chlorotetrafluoride was a minor product due to the steric hindrance of the relatively large bromo substituent at the *ortho* position. Arylsulfur chlorotetrafluorides were also prepared from the corresponding aryl thiols in high yields, as shown in runs 2 and 4, Table 1.

**Scheme 2:**
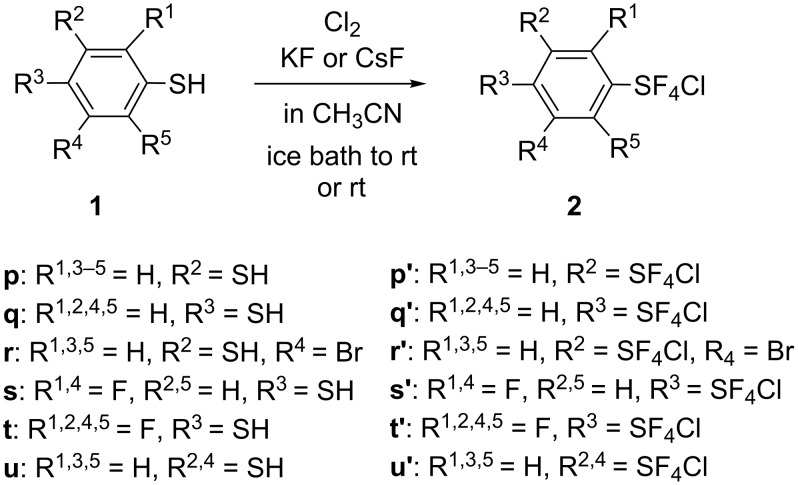
Preparation of Ar(SF_4_Cl)*_n_* from Ar(SH)*_n_* (*n* = 2, 3).

The method using aryl thiols as starting materials was successfully applied to the preparation of aryl bis- and tris(sulfur chlorotetrafluorides) as shown in Scheme 2 and Table 2. The method using the corresponding polymeric disulfides did not work well because of their extremely low solubility.

**Table 2:** Preparation of aryl bis- and tris(sulfur chlorotetrafluorides) **2p'–u'**.

run^a^	Ar(SH)*_n_* (mmol)^b^	Cl_2_^c^	KF^d^	Ar(SF_4_Cl)*_n_*^e^	conditions^f^	yield (%)^g^

1	**1p** (68.7)	18	25	**2p′** (*t*)	ice bath, 6 h to rt, 2 d	56
2	**1q** (64)	20	47	**2q′** (*t*)	ice bath, 6 h to rt, 2 d	74
3	**1r** (80)	23	38	**2r′** (*t*/*c* = 89/11)	rt, 2 d	96^h^
4	**1s** (63)	20	20	**2s′** (*t*/*c* = 95/5)	ice bath, 7 h to rt, o.n.	52
5	**1t** (64)	20	27	**2t′** (*t*/*c* = 58/42)	ice bath, 7 h to rt, o.n.	79
6	**1u** (57)	40	60	**2u′** (*t*/*c* = 79/21)	rt, 3 d	98^h^

^a^The experimental procedure is described in the Supporting Information File 1. ^b^The number in parentheses is the amount (mmol) of Ar(SH)*_n_* used. ^c^Molar ratio of Cl_2_ per mole of Ar(SH)*_n_*. ^d^Molar ratio of KF per mole of Ar(SH)*_n_*. ^e^*t* = *trans*-configuration, *c* = *cis*-configuration. The *t*/*c* ratio was determined by ^19^F NMR of the reaction mixture or crude product. ^f^rt = room temperature, o.n. = overnight. *^g^*Isolated yields. *^h^*Crude product.

The reaction of a diaryl disulfide with Cl_2_ and KF is given as Equation 1. Per 1 mol of a diaryl disulfide, 5 mol of Cl_2_, and 8 mol of KF are theoretically consumed.

[1]
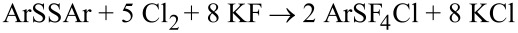


**Scheme 3:**
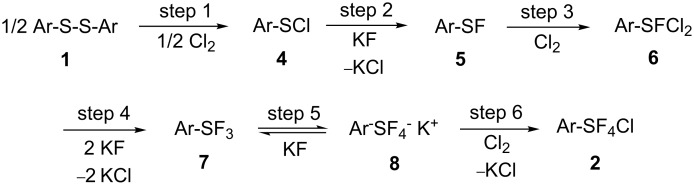
Reaction mechanism for the formation of ArSF_4_Cl.

Scheme 3 shows a postulated reaction mechanism, which consists of six steps including intermediates **4**, **5**, **6**, **7** and **8**. Treatment of *p*-nitrophenylsulfenyl chloride with Cl_2_/KF gave *p*-nitrophenylsulfur chlorotetrafluoride in 76% yield. Treatment of phenylsulfur trifluoride with Cl_2_/KF gave phenylsulfur chlorotetrafluoride in 84% yield. These results support the hypothesis that arylsulfenyl chloride **4** and trifluoride **7** are intermediates for the reaction of diaryl disulfide giving arylsulfur chlorotetrafluoride.

In a typical reaction of diphenyl disulfide, an orange color appears immediately as chlorine (Cl_2_) gas is introduced into a mixture of Ar_2_S_2_ and KF in acetonitrile. Cl_2_ is absorbed as fast as it is introduced until ArSF_3_ **7** is formed, at which point the solution becomes colorless. ^19^F NMR analysis of the reaction mixture at this moment confirms the formation of **7**. After that, the absorption of Cl_2_ becomes slow. Thus, the sequence of steps 1 to 4 giving **7** is fast, while the sequence of steps 5 and 6 giving the final product **2** is slow. The slow reaction is probably due to an equilibrium reaction (step 5) between **7** and **8**. The reaction of aryl thiol as a starting material is similar to that of the disulfide **1** since aryl thiol reacts with Cl_2_ to form disulfide **1**.

**Scheme 4:**
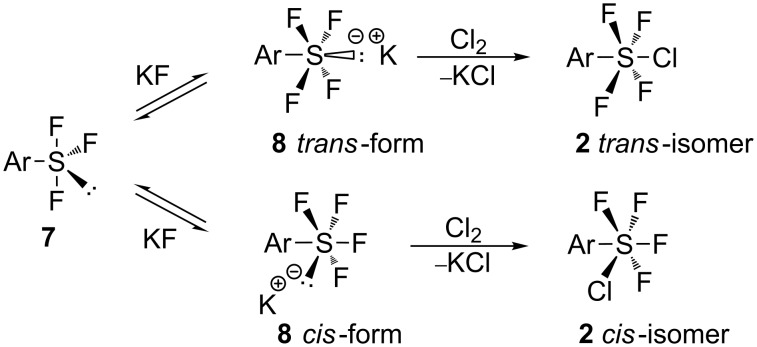
Reaction mechanism for the formation of *trans* and *cis*-ArSF_4_Cl.

The arylsulfur chlorotetrafluorides **2a**–**j** and bis(sulfur chlorotetrafluorides) **2p′** and **2q′** obtained were *trans*-isomers, while the polyfluorinated **2k**–**o**, bromo and polyfluoro bis(sulfur chlorotetrafluorides) **2r′–t′**, and tris(sulfur chlorotetrafluoride) **2u′** were a mixture of *trans*- and *cis*-configuration. Since we did not observe any isomerization at room temperature or upon distillation, it is reasonable to conclude that each isomer was formed through each isomeric salt of **8** as shown in Scheme 4. The multifluoro derivatives **2o** and **2t′** have the highest ratio of *cis*-configuration. This suggests that the relative stability of the *cis*-isomeric salts **8** increases particularly with increased fluorine substitution. The high ratio of *cis*-configuration of **2u′** suggests the ability of electron-withdrawing groups, such as –SF_4_Cl, to stabilize the *cis*-configuration salt form.

It is noticeable that, except for the polyfluorinated compounds and others discussed above, *trans*-isomeric ArSF_4_Cl was exclusively formed by the reactions of Ar_2_S_2_ with Cl_2_/KF, while the reactions of Ar_2_S_2_ with XeF_2_/Et_4_NCl gave a mixture of *cis-* and *trans*-isomers according to Janzen's report [52]. Janzen proposed radical reactions with Cl^·^ species [52]. The Cl_2_/KF reactions are ionic in nature, in which *trans*-form salts **8** exclusively form and react with Cl_2_ to give the *trans*-isomers.

Arylsulfur chlorotetrafluorides are stable during long periods of storage in a fluoropolymer vessel at room temperature. We did not observe any isomerization between *trans*- and *cis*-isomers on standing. Whereas arylsulfur trifluorides are extremely sensitive to moisture (water) [50,53], with the exception of the Fluolead reagent [50], arylsulfur chlorotetrafluorides are relatively insensitive to moisture. The half life time of decomposition of phenylsulfur chlorotetrafluoride (**2a)** in a CDCl_3_ solution (≈1.2 mol/L) on direct contact with water was 300 to 500 min at room temperature. The tracing experiment was conducted with a NMR tube. The ^19^F NMR was measured by using an internal standard (*p*-chlorobenzotrifluoride) and the NMR tube was shaken between the measurements. Gas chromatography (GC) could not be used because of decomposition of arylsulfur chlorotetrafluorides in the GC column. When a few drops of D_2_O were added to a solution of **2a** in CD_3_CN (1 mL), **2a** decomposed in 1 h and the decomposition product was phenylsulfonyl chloride. Phenylsulfonyl fluoride was detected in a trace amount (≤1%) by GC–mass analysis.

Arylsulfur chlorotetrafluorides **2** have relatively high thermal stability. Thus, **2a** did not decompose during 134 hours at 100 °C or 48 h at 150 °C in a Teflon tube. The isomerization of the *trans-* to the *cis*-isomer occurred very slowly. A very small amount (3–4%) of the *cis*-isomer was formed after heating of **2a** (*trans*-isomer) at 150 °C for 48 h. The real thermal decomposition temperatures of **2** could not be determined by a differential scanning calorimeter (DSC), because **2** reacted with the cell materials, i.e., stainless steel and gold, at elevated temperature due to their strongly oxidizing effect on the hexavalent sulfur(VI) element. Many arylsulfur chlorotetrafluorides were measured with DSC and these are discussed in Supporting Information File 1.

We examined reaction conditions for the conversion of *trans*-PhSF_4_Cl (**2a**) to PhSF_5_ (**3a**) with various reactive fluorides using an approximately one-gram scale of **2a**, as seen in Table 3. Janzen et al. described in the experimental section that bubbling an excess of BF_3_ into a mixture of *cis*- and *trans*-**2a** in CD_2_Cl_2_ at 25 °C led to the gradual disappearance of **2a** and the formation of PhSF_5_ (**3a**) [52]. We conducted the reaction of *trans*-**2a** (isolated) with BF_3_ in a sealed reactor and found that all the starting material became a solid residue, probably a polymer (run 1, Table 3). The reaction in dichloromethane solvent resulted in only a 28% yield of **3a** (run 2). The use of HBF_4_·OEt_2_ at room temperature provided a better yield (40%) (run 3). A strong Lewis acid SbF_5_ led to polymeric product, but SbF_3_ at 80 °C gave 33% (run 4). A combination of SbF_3_/SbCl_5_(cat.) at room temperature provided a better yield (54%) (run 5). At 80 °C, transition-metal fluorides TiF_4_ and CuF_2_ afforded 35 and 57%, respectively (runs 7, 8). Finally, we found that inexpensive and easily handled ZnF_2_ produced **3a** in high yield (runs 9, 10).

**Table 3:** Conversion of phenylsulfur chlorotetrafluoride (**2a**) to PhSF_5_ (**3a**).

run^a^	**2a** (mmol)^b^	fluoride (mmol)^c^	solvent (mL)^d^	temperature (°C)	time	yield of **3a** (%)^e^

1	4.5	BF_3_^f^	none	rt	3 d	0
2	6.4	BF_3_^f^	CH_2_Cl_2_ (6.4)	rt	5 h	28
3	4.5	HBF_4_·OEt_2_ (5.4)	CH_2_Cl_2_ (4.5)	rt	21 h	40
4	4.5	SbF_3_ (2.2)	none	80	5 h	33
5	4.5	SbF_3_/SbCl_5_ (2.0/cat.)	hexane (2)	rt	3 d	54
6	4.5	SnF_4_ (1.4)	none	80	2 h	34
7	4.5	TiF_4_ (1.4)	none	80	16 h	35
8	4.5	CuF_2_ (2.8)	none	80	22 h	57
9	4.5	ZnF_2_ (2.7)	none	80	20 h	85
10	4.5	ZnF_2_ (2.7)	none	120	4 h	88
11	13.6	ZnF_2_/SbCl_5_ (8.2/1.4)	heptane (5)	rt	17 h	53

^a^The experimental procedure is described in Supporting Information File 1. ^b^The amount (mmol) of **2a** used. ^c^The number in parentheses is the amount (mmol) of fluoride used. ^d^The number in parentheses is the amount (mL) of solvent used. ^e^Determined by ^19^F NMR. ^f^See Supporting Information File 1 for the amount of BF_3_ used.

A ^19^F NMR tracing experiment of the conversion reaction of *trans*-PhSF_4_Cl (*trans*-**2a**) provided some information on the reaction mechanism. With HBF_4_·OEt_2_, it was observed that the molar ratio of *trans*-**2a**:*cis*-**2a**:PhSF_5_ was 156:172:100 in the reaction mixture after 7 h, and 3:6:100 after 21 h. With ZnF_2_, the ratio observed was 22:117:100 during the reaction. A considerable amount of the *cis*-isomer was formed as an intermediate. It may thus be suggested that there are two routes, a direct route of the *trans*-isomers to the SF_5_ products and an indirect route via *cis*-isomers. The experiment with HBF_4_·OEt_2_ or ZnF_2_ may be considered to be thermal isomerization of *trans*-**2a** to *cis*-**2a** with an acid catalyst, suggesting that the *cis*-isomer is more thermodynamically stable than the *trans*-isomer. With ZnF_2_/SbCl_5_ the *cis*-isomer was barely detected (run 11, Table 3). The ratio of *trans*-**2a**:*cis*-**2a**:PhSF_5_ was 385:0:100 after 10 min; 63:trace:100 after 1.5 h; 34:trace:100 after 3 h; and 18:2:100 after 17 h. Thus, the lack of detectable *cis*-isomer may suggest that the addition of a strong Lewis acid such as SbCl_5_ gives priority to the direct route. However, we cannot rule out the possibility that the conditions could very quickly convert the *cis*-isomer to the product.

**Scheme 5:**
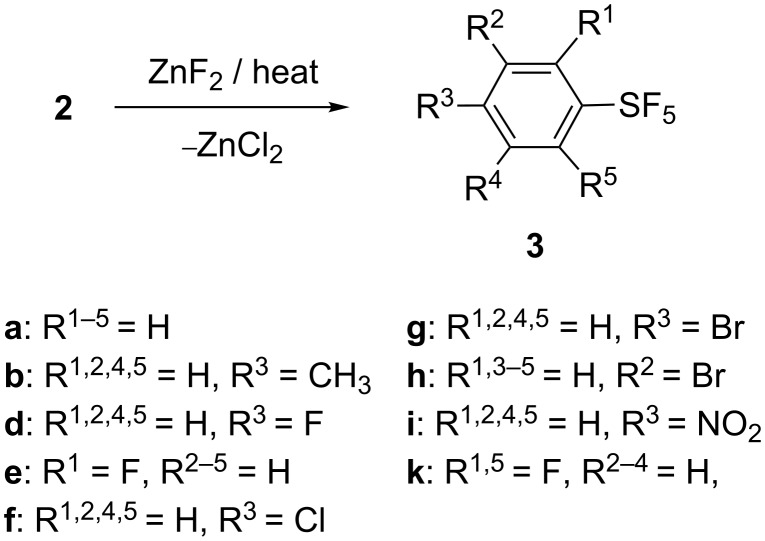
Preparation of ArSF_5_ with ZnF_2_.

The method with ZnF_2_ was applied to 10–50 gram scale reactions of PhSF_4_Cl (**2a**) and its derivatives **2b,d,e–i,k** (Scheme 5). A fluoropolymer reactor charged with the reactants (ArSF_4_Cl and ZnF_2_) was heated under the pressure of a balloon filled with N_2_ gas (no flow of N_2_). The reaction conditions and yields are shown in Table 4. Liquid **2a,b,d–f,h** efficiently reacted with solid ZnF_2_ (powder) under stirring without solvent. The two fluorine atoms of ZnF_2_ were effectively consumed for the reaction. **2a** and monohalogenated **2d–h** were converted to the corresponding products **3** in good to high yields at 120 °C (bath temperature). It was observed that the start of the exothermic reaction of halogenated **2f** was significantly delayed compared to that of unsubstituted **2a**. *p*-Methyl-**2b** reacted with ZnF_2_ at 90 °C (run 2). *p*-Nitro-**2i** and 2,6-difluoro-**2k** required a high temperature of 150 °C or more and their yields were fair to poor (runs 8 and 9). Thus, the electron-donating substituents increase the reactivity of –SF_4_Cl, while the electron-withdrawing ones decrease it.

**Table 4:** Preparation of various arylsulfur pentafluorides **3** with ZnF_2_.

run^a^	**2** (mmol)^b^	solvent	temp (°C)	time (h)	product **3**	yield (%)^c^

1	**2a** (200)	none	120	20	**3a**^d^	75
2	**2b** (137)	none	90	overnight	**3b**^e^	71
3	**2d** (42)	none	120	16	**3d**^f^	62
4	**2e** (42)	none	120	15	**3e**	59
5	**2f** (175)	none	120	16	**3f**^d,g^	73
6	**2g** (100)	heptane (20 mL)	reflux	17	**3g**^d^	79
7	**2h** (33)	none	120	15	**3h**^d^	78
8	**2i** (100)	none	150	72	**3i**^d^	36
9	**2k** (160)	none	130→180	4→6	**3k**	52

^a^The amount of ZnF_2_ used was 0.6 mol per 1 mol of **2** in runs 1–4 and 6–8, 0.53 mol in run 5, and 1.06 mol in run 9. The experimental procedure is described in Supporting Information File 1. ^b^The number in parentheses is the amount (mmol) of **2** used. ^c^Isolated yields. ^d^See [2]. ^e^See [52]. ^f^See [54]. ^g^Product **3f** (purity 97%) obtained after distillation was contaminated with 3% of *p*-dichlorobenzene (major) and trichlorobenzene (minor). Purity was determined by GC.

It was found that the product *p*-chloro-**3f** obtained after distillation (run 5, Table 4) was contaminated (3%) with *p*-dichlorobenzene (major) and trichlorobenzene (minor), which were formed by cleavage of the C–S bond, in the case of the major byproduct, and further chlorination, in the case of the minor one, during the reaction. The complete removal of the byproducts from **3f** was difficult due to similar boiling points. We found that the byproducts were suppressed by the addition of a strong Lewis acid AlCl_3_ and that the addition of ZnCl_2_ modified the exothermic reaction of **2f** with ZnF_2_. The addition of AlCl_3_ decreased the reaction temperature and the addition of ZnCl_2_ probably helped to form reactive “ZnFCl” species. Finally the byproduct was restricted to less than 1% with ZnF_2_/ZnCl_2_/AlCl_3_ (molar ratio 100:10:5). The detailed experimental procedure is described in Supporting Information File 1.

The reaction of PhSF_4_Cl (**2a**) with ZnF_2_ in Table 4 was conducted under nonflowing N_2_ gas (under the pressure of a N_2_ balloon). When the reaction of **2a** was conducted under a flow of N_2_ gas, the reaction rate became low. We then examined the reaction conditions for **2a** in more detail and found that the reaction was dependent upon the atmosphere of the reaction mixture. While the reaction conducted under a N_2_ balloon (no flow of N_2_) was completed in 4 h in 88% yield, the reaction conducted under a flow of N_2_ (through a reactor) was not completed in 5 h and its yield was down to 67%. With a faster flow of N_2_, it became slower and the yield decreased. Apparently, a small amount of gas was generated at the beginning of the reaction. The gas was not analyzed because of experimental difficulty. Therefore, it was most likely that removal of the gas by the flow of N_2_ gas made the reaction slow. Surprisingly, when the reactor was filled with Cl_2_ gas, the reaction was completed in a short time (1.7 h) and its yield was very high (92%). Thus, the presence of Cl_2_ significantly accelerated the reaction rate and increased the yield. We assumed that one of the effects of the Cl_2_ atmosphere may be to inhibit a possible disproportionation reaction [2PhSF_4_Cl (**2a**) → PhSF_5_ + PhSF_3_ + Cl_2_ ↑], as the disproportionation leads to the formation of Cl_2_. Although it remains unclear as to why Cl_2_ accelerated the reaction, Cl_2_ may also activate ZnF_2_ or intermediate “ZnFCl” species.

Interestingly, this Cl_2_ atmosphere was effective for the reaction of the *p*-methyl derivative **2b** with ZnF_2_, but not for the *p*-chloro derivative **2f**. The reaction of **2f** with ZnF_2_ was not affected by N_2_ flow. As it is known that sulfur-related disproportionation, for instance arylsulfinic acid giving arylsulfonic acid and *S*-aryl arylthiosulfonate ester, is retarded by an electron-withdrawing group [55], it may thus be suggested that an electron-withdrawing substituent limits the disproportionation. Possibly the electron-withdrawing substituent lowers the disproportionation rate or increases the temperature necessary for disproportionation such that it is greater than that required for the replacement reaction of –SF_4_Cl to –SF_5_.

The method with easily handled and inexpensive ZnF_2_ under a Cl_2_ atmosphere was successfully applied to a large-scale production (≈0.5 kg) of **3a** from **2a**, in which the addition method was adopted and a small amount of product **3a** was used as the reaction solvent. This procedure is described in Supporting Information File 1.

In contrast, anhydrous hydrogen fluoride (HF) is not easy to handle under normal laboratory conditions due to its high toxicity. However, in industry, in addition to its availability as a cheap fluorine source, the gaseous or liquid nature of HF (bp 19 °C) is quite suitable for large-scale industrial processes due to its ease in transfer, recovery and recycling.

**Scheme 6:**
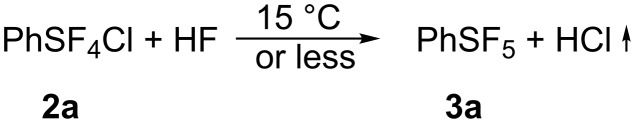
Preparation of PhSF_5_ with anhydrous HF.

**2a** satisfactorily reacted with HF at less than its boiling point to produce **3a** along with the evolution of hydrogen chloride (Scheme 6). As seen in Table 5, this method has successfully been applied to various substituted arylsulfur chlorotetrafluorides. The products obtained by the method were of high purity (≥99%) except for the cases of methyl derivative **2b** (runs 4–6). When the reaction of **2a** was conducted with the addition of KHF_2_ (KF:HF = 1:1), the yield improved (run 3). KF suppressed the formation of impurities, such as polymeric residue and chlorinated byproducts, because basic KF neutralizes the strong acid HCl formed in the reaction.

**Table 5:** Preparation of arylsulfur pentafluorides **3** with anhydrous hydrogen fluoride.

run^a^	ArSF_4_Cl **2** (mmol)^b^	molar ratio **2**/HF/additive	additive^c^	temperature (°C)	time (h)	ArSF_5_ **3**	yield (%)^d^

1	**2a** (87.1)	1/29/–	none	15	20	**3a**	62
2	**2a** (152)	1/24/–	none	−10	20	**3a**	66
3	**2a** (96.2)	1/25/1.1	KHF_2_	15	18	**3a**	73
4	**2b** (144)	1/22/–	none	15	19	**3b**	73^e^
5	**2b** (89.8)	1/22/1.2	KHF_2_	15	20	**3b**	79^f^
6	**2b** (80.8)	1/30/0.37	PhH	15	79	**3b**	57^g^
7	**2b** (91.8)	1/23/1.2/0.33^i^	KHF_2_, PhH	15	17	**3b**	56^h^
8	**2d** (90.2)	1/28/-	none	15	21	**3d**	67
9	**2e**^j^ (292)	1/23/-	none	19	22	**3e**	76^h^
10	**2f** (146)	1/23/-	none	15	20	**3f**	71
11	**2g** (250)	1/32/-	none	20	2 d	**3g**	77

^a^The experimental procedure is described in Supporting Information File 1. ^b^The number in parentheses is the amount (mmol) of **2** used. ^c^KHF_2_ = potassium hydrogen difluoride. PhH = benzene. ^d^Isolated yields after distillation. Purities of the products were >99% except for the cases labeled with superscripts e, f, g, h. Purity was determined by GC. ^e^Purity was 91%. ^f^Purity was 97%. ^g^Purity was 90%. ^h^Purity was 99%. ^i^Molar ratio: 1/23/1.2/0.33 = **2b**/HF/KHF_2_/PhH. ^j^**2e** (71 wt %) in CH_3_CN was used, which was obtained by concentration (with a vacuum pump) of the filtrate of the reaction mixture after completion of reaction of Scheme 1.

In run 4, the purity of product **3b** was 91%, which was contaminated with 8% of 3-chloro-4-methylphenylsulfur pentafluoride (**3b·Cl**) as the main byproduct. Compound **3b·Cl** was tentatively assigned by GC–mass analysis. When the reaction was conducted with the addition of KHF_2_, the purity greatly increased to 97% (run 5). The occurrence of an intermolecular side chlorination reaction was clearly demonstrated by the experiment of run 6 in which benzene was used as an additive. The distilled product **3b** (purity 90%) was contaminated with *p*-dichlorobenzene (6%) and *o*-dichlorobenzene (3%) in addition to the byproduct **3b·Cl** (1%). The dichlorobenzenes were formed by chlorination of the added benzene. Chlorobenzene formed in this reaction was contained in an initial distillation fraction, which was separated. The method of run 7 using both KHF_2_ and benzene as additives provided product **3b** with 99% purity, as the formation of impurity **3b·Cl** was completely suppressed. It is likely that the SF_4_Cl part of **2b** acts as a chlorinating agent toward **3b**, or another **2b** and benzene, under the strong acidic conditions that are formed from the HCl generated in the anhydrous HF.

As seen in runs 8–11, halogenated arylsulfur pentafluorides **3d**–**g** of high purity (≥99%) were obtained in good isolated yields without any additive. The side reactions such as polymerization and chlorination are restrained, as the aromatic nuclei are deactivated by the electron-withdrawing effect of the halogen atoms.

**Scheme 7:**
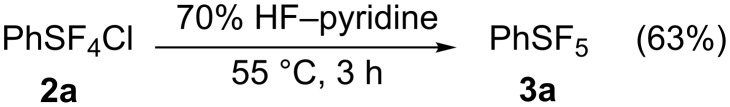
Preparation of **3a** with HF–pyridine.

It was also found that when PhSF_4_Cl (**2a)** was treated with a 70:30 w/w mixture of HF–pyridine at 55 °C, it gave PhSF_5_ (**3a)** in 63% isolated yield (Scheme 7). The HF–pyridine reagent is a nonvolatile and easily handled chemical in the laboratory.

**Scheme 8:**
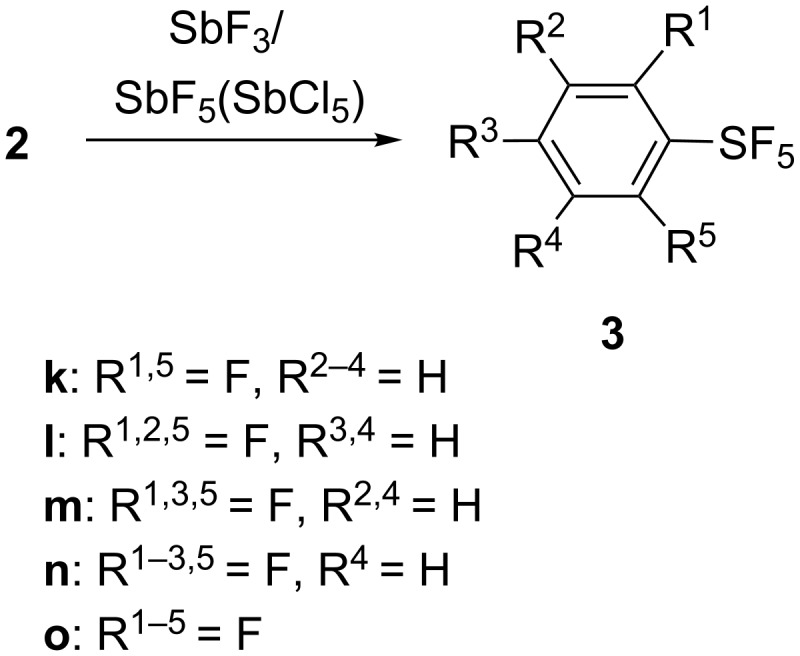
Preparation of polyfluorinated ArSF_5_.

Polyfluorinated arylsulfur chlorotetrafluorides **2k**–**o** were smoothly converted to the corresponding sulfur pentafluorides **3k**–**o** in good yields by treatment with a combination of SbF_3_ and a strong Lewis acid, such as SbF_5_ or SbCl_5_, as shown in Scheme 8 and Table 6.

**Table 6:** Preparation of polyfluorinated arylsulfur pentafluorides with Sb(III)/(V) fluorides.

run^a^	**2** (mmol)^b^	Sb(III) (mmol)^c^	Sb(V) (mmol)^c^	molar ratio Sb(III)/Sb(V)	solvent (mL)^d^	temperature (°C)	time (h)	**3**	yield (%)^e^

1	**2k** (3.9)	SbF_3_ (5.7)	SbCl_5_ (0.4)	14/1	FC-72 (8)	rt	1	**3k**	71
2	**2l** (36)	SbF_3_ (39)	SbF_5_ (9)	4.3/1	FC-72 (40)	−60 → rt	5	**3l**	70
3	**2m** (106)	SbF_3_ (121)	SbF_5_ (26.7)	4.5/1	FC-72 (110)	−60 → rt	≈5	**3m**	77
4	**2n** (118)	SbF_3_ (145)	SbF_5_ (22.3)	6.5/1	FC-72 (200)	rt	4.5	**3n**	61
5	**2o** (30.3)	SbF_3_ (32)	SbF_5_ (30.4)	1.05/1	FC-72 (40)	rt	1	**3o**	71

^a^The experimental procedure is described in Supporting Information File 1. ^b^The number in parentheses is the amount (mmol) of **2** used. ^c^The number in parentheses is the amount (mmol) of the Sb halide used. ^d^The number of parentheses is the amount (mL) of the solvent used. FC-72 is a perfluorocarbon with bp 56 °C (3M Fluorinert™ Electronic Liquid FC-72, 3M Specialty Materials, St. Paul, MN, USA). ^e^Isolated yields.

While treatment of 2,4,6-trifluoro-**2m** with a mixture of SbF_3_/SbF_5_ (4.5/1) gave 77% of product **3m** (run 3, Table 6), treatment of **2m** with SbF_5_ alone gave a byproduct (20%) in addition to **3m** (60%). The byproduct was tentatively assigned as 3-chloro-2,4,6-trifluorophenylsulfur pentafluoride by ^1^H and ^19^F NMR and GC–mass analysis. The chlorination as a side reaction may occur from the action of a strong Lewis acid SbF_5_ on the fluorine atoms of the SF_4_Cl group of ArSF_4_Cl **2m**, forming a [ArSF_3_Cl]^+^ [SbF_6_]^−^ species (Ar = 2,4,6-trifluorophenyl), which may act as a strong chlorinating agent (Cl^+^) toward **3m** or another molecule of **2m**. Pentafluoro-**2o** was converted to **3o** in good yield with a high molar ratio of SbF_5_ (run 5) as the reactivity of **2o** was considerably decreased by the five fluorine atoms.

**Scheme 9:**
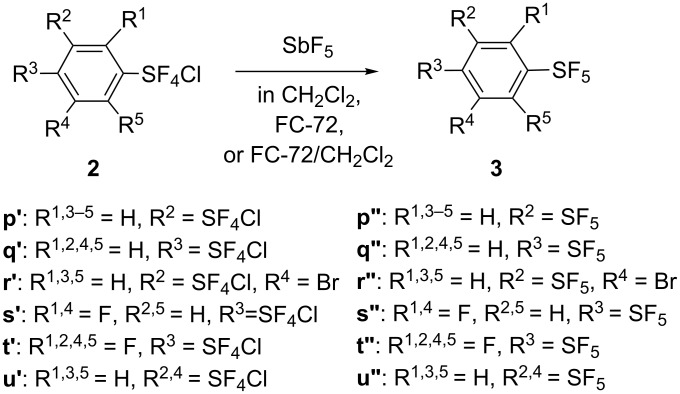
Preparation of aryl bis- and tris(sulfur pentafluorides), Ar(SF_5_)*_n_* (*n* = 2,3).

Phenyl bis(sulfur chlorotetrafluorides) **2p′** and **2q′**, bromo derivative **2r′,** and fluoro derivatives **2s′** and **2t′** were smoothly converted to the corresponding bis(sulfur pentafluorides) **3p″–t″** in fair to good yields with SbF_5_ alone, as shown in Scheme 9 and Table 7 (runs 1–5). Phenyl tris(sulfur chlorotetrafluoride) **2u′** was converted to phenyl tris(sulfur pentafluoride) **3u″** in 55% yield under similar conditions (run 6, Table 7).

**Table 7:** Preparation of aryl bis- and tris(sulfur pentafluorides) with SbF_5_.

run^a^	**2** (mmol)^b^	SbF_5_ (mmol)^c^	solvent (mL)^d^	temperature (°C)	time (h)^e^	product **3**	yield (%)^f^

1	**2p′** (10.8)	21.4	CH_2_Cl_2_ (45)	−85 → −25	2.5	**3p″**	57
2	**2q′** (11)	24	CH_2_Cl_2_ (80)	−85 → −15	1.5	**3q″**	71
3	**2r′** (77)	130	CH_2_Cl_2_ (450)	ca. −85 → −15	5	**3r″**	66
4	**2s′** (27.8)	30.7	FC-72 (390)	−80 → rt, then rt	8, then o.n.	**3s″**	52
5	**2t′** (12.4)	14.4	FC-72 (70)	rt	o.n.	**3t″**	67
6	**2u′** (32)	99	CH_2_Cl_2_/FC-72 (160/68)	−95 → +7	6	**3u″**	55

^a^The experimental procedure is described in Supporting Information File 1. ^b^The number in parentheses is the amount (mmol) of **2** used. ^c^The number is the amount (mmol) of SbF_5_ used. ^d^The number in parentheses is the amount (mL) of the solvent used. FC-72 is a perfluorocarbon having bp 56 °C (3M Fluorinert™ Electronic Liquid FC-72, 3M Specialty Materials, St. Paul, MN, USA). ^e^o.n. = overnight. ^f^Isolated yields.

## Conclusion

We have developed the first practical and economical method for the production of various arylsulfur pentafluorides and their higher homologues, which consists of the treatment of diaryl disulfides or aryl thiols with chlorine in the presence of potassium or cesium fluoride, followed by treatment of the resulting arylsulfur chlorotetrafluorides with a fluoride, such as ZnF_2_, HF, and Sb(III/V) fluorides. The important characteristics of these new reactions were revealed and some reactions were modified to provide the products in high purity and in high yields. Since these methods are superior to the previous methods using AgF_2_, F_2_, or XeF_2_, or multiple-step methods starting from SF_5_Br or SF_5_Cl, in terms of cost, applicability, and scalability of production, the processes developed here can be used as the first practical and economical methods for the production of many kinds of arylsulfur pentafluorides. Thus, it is expected that this will lead to new and rapid advances in “super-trifluoromethyl” arene chemistry and associated industries in many areas.

## Supporting Information

File 1 Experimental details and copies of ^1^H-, ^19^F-, and ^13^C NMR spectra of new products.
