# Supplementary material for: Discovery of practical production processes for arylsulfur pentafluorides and their higher homologues, bis- and tris(sulfur pentafluorides): Beginning of a new era of “super-trifluoromethyl” arene chemistry and its industry
Source: Beilstein J Org Chem. 2012 Mar 29;8:461–71. doi: 10.3762/bjoc.8.53 (PMC3326626; doi:10.3762/bjoc.8.53)
Supplement: File 1 — Experimental details and copies of 1H-, 19F-, and 13C NMR spectra of new products. [file Beilstein_J_Org_Chem-08-461-s001.pdf]

Supporting Information

for

Discovery of practical production processes for arylsulfur pentafluorides and their higher homologues, bis- and tris(sulfur pentafluorides): Beginning of a new era of "super-trifluoromethyl" arene chemistry and its industry

Teruo Umemoto*[§], Lloyd M. Garrick[§] and Norimichi Saito[§]

Address: IM&T Research, Inc./Ube America Inc., 6860 N. Broadway, Suite B, Denver, Colorado 80221, U. S. A.

E-mail: Teruo Umemoto* - teruoumemoto@comcast.net

*Corresponding author

[§] Currently: Ube America Inc.

Experimental details and copies of ^{1}H-, ^{19}F-, and ^{13}C NMR spectra of new products

Table of Contents

Experimental Section

1. **General** p. S2
2. **Preparation of arylsulfur chlorotetrafluorides ($ArSF_4Cl$)** p. S3
   - **2-1. Preparation of $ArSF_4Cl$ from diaryl disulfides** p. S3
   - **2-2. Preparation of $ArSF_4Cl$ from aryl thiols (ArSH)** p. S7

2-3. Preparation of *p*-(NO_2)$C_6H_4SF_4Cl$ from *p*-(NO_2)C_6H_4SCl p. S7

2-4. Preparation of $PhSF_4Cl$ from $PhSF_3$ p. S8

3. Preparation of aryl bis- and tris(sulfur chlorotetrafluoride), Ar(SF_4Cl)$_n$ (n = 2 and 3) p. S8

4. Preparation of arylsulfur pentafluorides ($ArSF_5$) from $ArSF_4Cl$ p. S10

4-1. Examination on the reactivity of $PhSF_4Cl$ to different fluorides p. S10

4-2. Preparation of various $ArSF_5$ from $ArSF_4Cl$ with ZnF_2 p. S11

4-3. Effect of the atmosphere on the reaction of $PhSF_4Cl$ with ZnF_2 p. S11

4-4. Large scale preparation of $PhSF_5$ from $PhSF_4Cl$ with ZnF_2 in Cl_2 atmosphere p. S13

4-5. Preparation of *p*-$ClC_6H_4SF_5$ from *p*-$ClC_6H_4SF_4Cl$ with ZnF_2/$ZnCl_2$/$AlCl_3$ p. S14

5. Preparation of $ArSF_5$ from $ArSF_4Cl$ with anhydrous hydrogen fluoride p. S15

6. Preparation of $PhSF_5$ from $PhSF_4Cl$ with 70% (w/w) HF-pyridine p. S15

7. Preparation of polyfluorinated $ArSF_5$ with SbF_3/SbF_5($SbCl_5$) p. S16

8. Preparation of aryl bis- and tris(sulfur pentafluoride), Ar(SF_5)$_n$ (n = 2,3) with SbF_5 p. S17

9. DSC measurement of arylsulfur chlorotetrafluorides p. S19

10. Preparation of starting materials 1j–n and 1r–u p. S21

11. References and Notes p. S27

12. ^{1}H-, ^{19}F-, and ^{13}C NMR spectra of new products p. S28

1. General

Chemicals were purchased and used without prior purification unless otherwise noted. Solvents CH_2Cl_2 and CH_3CN were distilled from CaH_2 before use. Spray-dried potassium fluoride and dry caesium fluoride were purchased from Sigma-Aldrich and used without further drying. Potassium fluoride (No. 402931) from Sigma-Aldrich was also used after being ground to a powder with a mortar and pestle and being dried at 250 °C for 2 or 3 days with a vacuum pump. Fluorinert® FC-72 (3M Company) (from SynQuest Labs.) was perfluorocarbon having bp 56 °C. Bis(pentafluorophenyl) disulfide (**1o**) was prepared in 92% yield by treating 2,3,4,5,6-pentafluorothiophenol with bromine in acetic acid according to the literature [S1]. Benzene-1,3-dithiol (**1p**) and benzene-1,4-dithiol (**1q**) were

prepared according to the literature procedure except that *N*-methylpyrrolidinone was used instead of HMPA [S2].

^{1}H, ^{19}F and ^{13}C-NMR spectra were recorded on a JEOL at 300.53, 282.78, and 75.57 MHz, respectively. Chemical shifts are reported in ppm with TMS (δ = 0.00); ^{19}F, $CFCl_3$ (δ=0.00).

2. Preparation of arylsulfur chlorotetrafluorides ($ArSF_4Cl$)

2-1. Preparation of $ArSF_4Cl$ from diaryl disulfides

$$\text{Ar-S-S-Ar} \xrightarrow[\text{KF}]{\text{Cl}_2} \text{ArSF}_4\text{Cl}$$

General procedure: A 500 mL round-bottom glassware flask equipped with gas (N_2, Cl_2) inlet and outlet tubes was charged with a diaryl disulfide (0.15 mol), dry KF (140 g, 2.4 mol, dried at 250 °C for 2 days under vacuum) and 300 mL of dry CH_3CN. The mixture was heated with stirring under N_2 to almost reflux to dissolve as much KF as possible. Then, the stirred reaction mixture was cooled on an ice/water bath under a flow of N_2 (about 20 mL/min). (Note: When spray-dried KF was used, the preheating to dissolve KF was not conducted.) After N_2 was stopped, chlorine (Cl_2) was bubbled into a stirred reaction mixture at a rate of about 70 mL/min. The Cl_2 bubbling took about 6.5 h. The total amount of Cl_2 used was about 1.2 mol. After Cl_2 was stopped, the reaction mixture was stirred overnight at room temperature. N_2 was then bubbled through for 2 h to remove an excess of Cl_2. The reaction mixture was then filtered and rinsed with 100 mL of dry hexanes in dry air or nitrogen. About 1 g of dry KF was added to the filtrate. KF may restrain possible decomposition (hydrolysis) of the product. The filtrate was evaporated under vacuum (with a vacuum pump) and the resulting residue was distilled at reduced pressure to give an arylsulfur chlorotetrafluoride. The reaction conditions and results for each $ArSF_4Cl$ are shown in Table 1 (Text). The physical and spectral data of the products are shown below.

Phenylsulfur chlorotetrafluorides (2a) [S3]: *trans*-Isomer; bp 80 °C /20 mmHg; ^{1}H NMR (CD_3CN) 7.79–7.75 (m, 2H), 7.53–7.49 (m, 3H); ^{19}F NMR (CD_3CN) 136.7 (s, SF_4); ^{13}C NMR ($CDCl_3$) δ 155.5 (quintet, J = 18 Hz), 131.7, 128.8, 125.9 (m); IR (neat) 3074, 1487, 1452, 1194, 1097, 809, 740, 687, 638, 604, 544 cm^{-1}.

***p*-Methylphenylsulfur chlorotetrafluoride (2b)** [S3]: *trans*-Isomer; bp 74–75 °C/5 mmHg; ^{1}H NMR (CD_3CN) 7.65 (d, 2H), 7.29 (d, 2H), 2.36 (s, 3H, CH_3); ^{19}F NMR (CD_3CN) 137.66 (s, SF_4); ^{13}C NMR ($CDCl_3$) δ 153.1 (quintet, J = 18 Hz), 142.3, 129.2, 125.7 (m), 21.0; IR (neat) 3040, 2929, 1602, 1504, 1195, 1100, 1087, 805, 699, 566, 545 cm^{-1}. High resolution mass spectrum (HRMS); found 235.986234 (34.9%) (calcd for $C_7H_7F_4S^{37}Cl$; 235.986363), found 233.989763 (75.6%) (calcd for $C_7H_7F_4S^{35}Cl$; 233.989313).

***p*-(*tert*-Butyl)phenylsulfur chlorotetrafluoride (2c)** [S3]: *trans*-Isomer; bp 98 °C/0.3 mmHg; mp 93 °C; ^{1}H NMR ($CDCl_3$) δ 1.32 (s, 9H, $C(CH_3)_3$), 7.43 (d, *J* = 9.2 Hz, 2H), 7.64 (d, *J* = 9.2 Hz, 2H); ^{19}F NMR δ 138.3 (s, SF_4); ^{13}C NMR (CD_3CN) 126.1, 125.5 (m), 117.4, 34.8, 30.2. HRMS; found 278.034576 (8.8%) (calcd for $C_{10}H_{13}{}^{37}ClF_4S$; 278.033313), found 276.037526 (24.7%) (calcd for $C_{10}H_{13}{}^{35}ClF_4S$; 276.036263). Anal. Calcd for $C_{10}H_{13}ClF_4S$; C, 43.40%; H, 4.74%. Found; C, 43.69%, H, 4.74%.

***p*-Fluorophenylsulfur chlorotetrafluoride (2d)** [S3]: *trans*-Isomer; bp 60 °C/8 mmHg; ^{1}H NMR (CD_3CN) 7.85–7.78 (m, 2H), 7.25–7.15 (m, 2H); ^{19}F NMR (CD_3CN) 137.6 (s, SF_4), −108.3 (s, CF); ^{13}C NMR ($CDCl_3$) δ 163.7 (d, *J* = 254 Hz), 151.2 (m), 128.4 (m), 115.6 (d, *J* = 23 Hz); IR (neat) 3123, 3084, 1599, 1504, 1412, 1246, 1170, 1091, 814, 707, 566, 550 cm^{-1}. HRMS; found 239.961355 (37.4%) (calcd for $C_6H_4F_5S^{37}Cl$; 239.961291), found 237.964201(100%) (calcd for $C_6H_4F_5S^{35}Cl$; 237.964241).

***o*-Fluorophenylsulfur chlorotetrafluoride (2e)** [S3]: *trans*-Isomer; bp 96–97 °C/20 mmHg; ^{1}H NMR (CD_3CN) 7.77-7.72 (m, 1H), 7.60–7.40 (m, 1H), 7.25–7.10 (m, 2H); ^{19}F NMR ($CDCl_3$) δ 140.98 (d, *J* = 24.1 Hz, SF_4), −107.69 (m, CF); ^{13}C NMR ($CDCl_3$) δ 155.8 (d, *J* = 260 Hz), 141.4 (m), 133.9 (d, *J* = 9 Hz), 128.4 (m), 124.0 (d, *J* = 24 Hz), 117.9 (d, *J* = 24 Hz). HRMS; found 239.961474 (25.4%) (calcd for $C_6H_4F_5S^{37}Cl$; 239.961291), found 237.964375 (69.8%) (calcd for $C_6H_4F_5S^{35}Cl$; 237.964241).

***p*-Chlorophenylsulfur chlorotetrafluoride (2f)** [S3]: *trans*-Isomer; bp 65–66 °C/2 mmHg; ^{1}H NMR ($CDCl_3$) δ 7.38 (d, 2H, *J* = 9.1 Hz), 7.65 (d, 2H, *J* = 9.1 Hz); ^{19}F NMR ($CDCl_3$) 137.4 (s, 4F, SF_4); ^{13}C NMR (CD_3CN) δ 153.4 (quintet, *J* = 19 Hz), 137.7, 129.0, 127.4 (m); IR (neat) 3107, 1580, 1480, 1401, 1196, 1082, 1014, 810, 763, 671, 621, 545, 493 cm^{-1}. HRMS; found 257.927507 (13.3%) (calcd for $C_6H_4F_4S^{37}Cl_2$; 257.928790), found 255.930746 (68.9%) (calcd for $C_6H_4F_4S^{37}Cl^{35}Cl$; 255.931740), found 253.933767 (100.0%) (calcd for $C_6H_4F_4S^{35}Cl_2$; 253.934690).

***p*-Bromophenylsulfur chlorotetrafluoride (2g)** [S3]: *trans*-Isomer; mp 58–59 °C; ^{1}H NMR (CD_3CN) δ 7.67 (s, 4H); ^{19}F NMR (CD_3CN) δ 136.56 (s, SF_4); ^{13}C NMR ($CDCl_3$) δ 154.2 (m), 131.9, 127.5 (m), 126.2; IR (nujol) 1577, 1396, 1070, 1010, 819, 752, 660, 619, 546 cm^{-1}. HRMS; found 301.877066 (16.5%) (calcd for $C_6H_4{}^{81}Br\,{}^{37}ClF_4S$; 301.879178), found 299.880655 (76.6%) (calcd for $C_6H_4{}^{81}Br\,{}^{35}ClF_4S$; 299.881224 and calcd for $C_6H_4{}^{79}Br^{37}ClF_4S$; 299.882128), found 297.882761 (77.4%) (calcd for $C_6H_4{}^{79}Br^{35}ClF_4S$; 297.884174). Anal. Calcd for $C_6H_4BrClF_4S$; C, 24.06%; H, 1.35%. Found, C, 24.37%; H, 1.54%.

***m*-Bromophenylsulfur chlorotetrafluoride (2h)** [S3]: *trans*-Isomer; bp 57–59 °C/0.8 mmHg; ^{1}H NMR (CD_3CN) 7.90–7.88 (m, 1H), 7.70–7.50 (m, 2H), 7.40–7.30 (m, 1H); ^{19}F NMR (CD_3CN)

136.74 (s, SF_4); ^{13}C NMR ($CDCl_3$) δ 156.1 (quintet, J = 19 Hz), 134.8, 130.1, 129.1 (m), 124.6 (m), 122.3; IR (neat) 3102, 1578, 1472, 1420, 1124, 1108, 1073, 812, 778, 670, 639, 609 cm^{-1}. HRMS; found 301.878031 (29.1%) (calcd for $C_6H_4{}^{81}Br\,{}^{37}ClF_4S$; 301.879178), found 299.881066 (100%) (calcd for $C_6H_4{}^{81}Br\,{}^{35}ClF_4S$; 299.881224 and calcd for $C_6H_4{}^{79}Br{}^{37}ClF_4S$; 299.882128), found 297.883275 (77.4%) (calcd for $C_6H_4{}^{79}Br{}^{35}ClF_4S$; 297.884174).

***p*-Nitrophenylsulfur chlorotetrafluoride (2i)** [S3]: *trans*-Isomer; mp 130−131 °C; ^{1}H NMR (CD_3CN) 8.29 (d, J = 7.8 Hz, 2H), 8.02 (d, J = 7.8 Hz, 2H); ^{19}F NMR (CD_3CN) 134.96 (s, SF_4); ^{13}C NMR ($CDCl_3$) δ 159.3 (m), 149.1, 127.6 (m), 124.1; IR (nujol) 1538, 1354, 1316, 1091, 859, 748, 665, 618, 538 cm^{-1}. HRMS; found 266.956490 (38.4%) (calcd for $C_6H_4{}^{37}ClF_4NO_2S$; 266.955791), found 264.959223 (100%) (calcd for $C_6H_4{}^{35}ClF_4NO_2S$; 264.958741). Anal. Calcd for $C_6H_4ClF_4NO_2S$; C, 27.13%; H, 1.52%; N, 5.27%. Found, C, 27.16%; H, 1.74%; N, 4.91%.

***p*-(Methanesulfonyl)phenyl chlorotetrafluoride (2j)**: *trans*-isomer; mp 159.5−160.3 °C; ^{1}H NMR ($CDCl_3$) δ 3.08 (s, CH_3), 7.96 (d, J = 8.7 Hz, 2H), 8.05 (d, J = 8.7 Hz, 2H); ^{19}F NMR ($CDCl_3$) δ 135.55 (s); ^{13}C NMR ($CDCl_3$) δ 159.0 (quintet, J = 20 Hz), 143.5, 128.3, 127.4 (m), 44.4; IR (nujol) 1302, 1149, 1095, 1089, 801, 747, 666, 618, 567, 548, 503 cm^{-1}. Anal. Calcd for $C_7H_7ClF_4O_2S_2$: C, 28.15%; H, 2.36%. Found: C, 28.19%; H, 2.51%.

2,6-Difluorophenylsulfur chlorotetrafluoride (2k) [S3]: The product (bp 120−122 °C/95−100 mmHg) obtained by distillation from Example 8 is a 90:10 mixture of *trans*- and *cis*-isomers of 2,6-difluorophenylsulfur chlorotetrafluoride. The *trans*-isomer was isolated in pure form by crystallization (pentane). *trans*-Isomer; mp. 47.6–48.3 °C; ^{1}H NMR ($CDCl_3$) δ 6.97–7.09 (m, 2H, 3,5-H), 7.43–7.55 (m, 1H, 4-H); ^{19}F NMR ($CDCl_3$) δ 143.9 (t, J = 26.0 Hz, 4F, SF_4), −104.1 (quintet, J = 26.0 Hz, 2F, 2,6-F): ^{13}C NMR ($CDCl_3$) δ 157.2 (d, J = 262.3 Hz), 133.7 (t, J = 11.6 Hz), 130.6 (m), 113.5 (d, J = 14.6 Hz). HRMS; found 257.950876 (37.6%) (calcd for $C_6H_3{}^{37}ClF_6S$; 257.951869), found 255.955740 (100%) (calcd for $C_6H_3{}^{35}ClF_6S$; 255.954819). Anal. Calcd for $C_6H_3ClF_6S$; C, 28.08%, H, 1.18%. Found; C, 28.24%, H, 1.24%. Although the *cis*-isomer of 2,6-difluorophenylsulfur chlorotetrafluoride was not isolated in pure form, the *cis*-isomer was assigned in the following; ^{19}F NMR ($CDCl_3$) δ 158.2 (quartet, J = 161.8 Hz, 1F, SF), 121.9 (m, 2F, SF_2), 76.0 (m, 1F, SF). The ^{19}F NMR assignment of aromatic fluorine atoms of the *cis*-isomer could not be done because of overlapping of the peaks of the *trans*-isomer. IR (mixture of *trans*/*cis*-isomers) (neat) 1614, 1592, 1296, 1240, 1105, 1015, 843, 789, 686, 642, 545 cm^{-1}.

2,3,6-Trifluorophenylsulfur chlorotetrafluoride (2l) [S4]: The product (bp 125−129 °C/95 mmHg) obtained by distillation was a mixture of a 87:13 mixture of *trans*- and *cis*-isomers by NMR analysis. *trans*-Isomer: ^{1}H NMR ($CDCl_3$) δ 7.38 (m, 1H), 7.01 (m, 1H); ^{19}F NMR ($CDCl_3$) δ 143.19 (t, J

= 27.2 Hz, 4F, SF_4), −109.24 (m, 1F), −127.20 (m, 1F), −137.97 (m, 1F); ^{13}C NMR ($CDCl_3$) δ 152.5 (d, *J* = 258.7 Hz), 147.5 (ddd, *J* = 249.3, 14.4, 3.6 Hz), 146.3 (dd, *J* = 264.4, 17.0 Hz), 131.6 (m), 120.6 (dd, *J* = 18.1, 10.8 Hz), 112.4 (dm, *J* = 27.5 Hz). *cis*-Isomer: ^{19}F NMR ($CDCl_3$) δ 155.50 (quartet, *J* = 160.9 Hz, 1F, SF), 122.12 (m, 2F, SF_2), 76.64 (m, 1F, SF), and aromatic fluorine atoms were not assigned because of overlapping with those of the major *trans*-isomer; ^{1}H and ^{13}C NMR of the minor *cis*-isomer was not assigned because of overlapping with those of the major trans-isomer. IR (*trans*/*cis* mixture); (neat) 3112, 1620, 1604, 1497, 1523, 1200, 1020, 890, 838, 746, 688, 643, 616, 547 cm^{-1}. Elemental analysis of liquid **2l** (*trans*/*cis* mixture) failed: Found; C, 27.53%; H, 0.84% (Calcd for $C_6H_2ClF_7S$: C, 26.24%; H, 0.73%). However, **2l** was derived to **3l** which provided a satisfactory elemental analysis.

2,4,6-Trifluorophenylsulfur chlorotetrafluoride (2m) [S4]: Purification by recrystallization of the crude product (*trans*/*cis* = 96/4) from pentane gave pure *trans*-isomer: mp 55.8–56.7 °C; ^{1}H NMR ($CDCl_3$) δ 6.79 (t, *J* = 17.5 Hz, m-H); ^{19}F NMR ($CDCl_3$) δ 144.07 (t, *J* = 26.0 Hz, 4F, SF_4), −99.80 (t, *J* = 26.0 Hz, 2F, o-F), −100.35 (s, 1F, p-F); ^{13}C NMR ($CDCl_3$) δ 164.2 (dt, *J* = 164.2 Hz, 15.2 Hz, 4-C), 158.2 (dm, *J* = 260.7 Hz, 2-C), 127.7 (m, 1-C), 102.1 (tm, *J* = 27.8 Hz, 3-C); IR (nujol) 1607, 1176, 1136, 1092, 1049, 1011, 847, 710, 581, 551 cm^{-1}. Anal. Calcd for $C_6H_2ClF_7S$: C, 26.24%; H, 0.73%. Found: C, 26.23%; H, 1.01%. *cis*-Isomer: ^{19}F NMR ($CDCl_3$) δ 157.8 (m, 1F, SF_4), 122.3 (m, 2F, SF_4), 77.0 (m, 1F, SF_4), and aromatic fluorine atoms could not be assigned.

2,3,4,6-Tetrafluorophenylsulfur chlorotetrafluoride (2n) [S4]: The product (bp 115–120 °C/120 mmHg) obtained by distillation was a 86:14 mixture of *trans*- and *cis*-isomers by ^{19}F NMR analysis. *trans*-Isomer of **2n** was isolated by crystallization of the mixture on long standing in a freezer. *trans*-Isomer: mp 38.8–40.0 °C; ^{19}F NMR ($CDCl_3$) δ 143.70 (t, *J* = 26.9 Hz, 4F, SF_4), −106.77 (m, 1F), −124.22 (m, 2F), −160.64 (m, 1F); ^{1}H NMR ($CDCl_3$) δ 6.9 (m); ^{13}C NMR ($CDCl_3$) δ 152.5 (dm, *J* = 259.8 Hz), 147.9 (dm, *J* = 269.0 Hz), 137.5 (dm, *J* = 253.0 Hz), 128.2 (m), 102.1 (m). *cis*-Isomer: ^{19}F NMR ($CDCl_3$) δ 154.92 (quartet, *J* = 160.6 Hz, 1F, SF), 122.90 (m, 2F, SF_2), 77.74 (m, 1F, SF), and aromatic fluorine atoms were not assigned because of overlapping with those of the major *trans*-isomer: ^{1}H and ^{13}C NMR of the minor *cis*-isomer was not assigned because of overlapping with those of the major *trans*-isomer. IR (mixture of *trans*/*cis*-isomers); (neat) 3094, 1633, 1514, 1450, 1205, 1089, 1061, 837, 703, 666, 559 cm^{-1}. Anal. (for *trans*-isomer) Calcd for C_6HClF_8S; C, 24.63%; H, 0.34%. Found: C, 24.06%; H. 0.62%.

2,3,4,5,6-Pentafluorophenylsulfur chlorotetrafluoride (2o) [S4]: The product (bp 95–112 °C/100 mmHg) obtained by distillation was a 63:37 mixture of *trans* and *cis* isomers of 2,3,4,5,6-pentafluorophenylsulfur chlorotetrafluoride. The isomers were assigned by ^{19}F NMR. *trans*-Isomer;

^{19}F NMR ($CDCl_3$) δ 144.10 (t, J = 26.0 Hz, 4F, SF_4), −132.1 to −133.1 (m, 2F, 2,6-F), −146.6 (m, 1F, 4-F), −158.9 (m, 2F, 3,5-F). *cis*-Isomer; ^{19}F NMR ($CDCl_3$) δ 152.39 (quartet, J = 158.9 Hz, 1F, SF), 124.32 (m, 2F, SF_2), 79.4 (m, 1F, SF), −132.1 to −133.1 (m, 2F, 2,6-F), −146.6 (m, 1F, 4-F), −158.9 (m, 2F, 3,5-F). ^{13}C NMR could not be assigned for each of the *trans* and *cis* isomers. HRMS of a 63:37 mixture of the *trans* and *cis* isomers; found 311.923124 (15.5%) (calcd for $C_6{}^{37}ClF_9S$; 311.923604), found 309.926404 (43.1%) (calcd for $C_6{}^{35}ClF_9S$; 309.926554). IR (mixture of *trans*/*cis*-isomers); (neat) 1644, 1525, 1503, 1110, 1002, 850, 795, 745, 680, 641, 534 cm^{-1}.

2-2. Preparation of $ArSF_4Cl$ from aryl thiols (ArSH)

Arylsulfur chlorotetrafluorides were also prepared from aryl thiols by the following procedure.

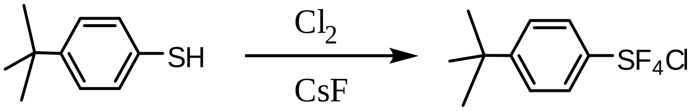

A typical procedure [S3]: Chlorine (Cl_2; 10.1 L, 451 mmol) was passed with a flow rate of 35 mL/min into a stirred mixture of 10.1 g (60.2 mmol) of *p*-*tert*-butylthiophenol and 91.6 g (602 mmol) of dry cesium fluoride in 150 mL of dry acetonitrile at 6–10 °C. The reaction mixture turned yellow and then colorless. After chlorine was passed, the reaction mixture was stirred at room temperature for 24 h. The reaction mixture was filtered and washed with 100 mL of dry acetonitrile in dry air or nitrogen. After complete removal of the solvent in vacuum at room temperature, the obtained solid was mixed with 100 mL of dry hexane and filtered under nitrogen. The filtrate (hexane) was concentrated and *p*-(*tert*-butyl)phenylsulfur chlorotetrafluoride (**2c**) (14 g, 84%) was obtained by crystallization. The product was a *trans* isomer.

The reaction conditions and results for thiophenol are shown in Table 1 (Text).

2-3. Preparation of *p*-(NO_2)$C_6H_4SF_4Cl$ from *p*-(NO_2)C_6H_4SCl

p-Nitrophenylsulfur chlorotetrafluoride (**2i**) was also prepared from *p*-nitrophenylsulfenyl chloride by the following procedure [S3].

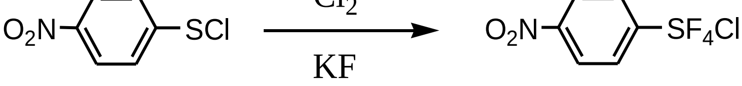

2i

Chlorine (Cl_2) was passed with a flow rate of 37 mL/min into a stirred mixture of 5.00 g (26.4 mmol) of p-nitrobenzenesulfenyl chloride and 15.3 g (264 mmol) of spray-dried KF in 40 mL of dry

acetonitrile at 5–11 °C. The total amount of chlorine passed was 2.54 L (113 mmol). The reaction mixture was filtered in dry air or nitrogen. After removal of the solvent in vacuum, 4.69 g (76%) of *trans-p*-nitrophenylsulfur chlorotetrafluoride (**2i**) as a solid was obtained.

2-4. Preparation of $PhSF_4Cl$ from $PhSF_3$

Phenylsulfur chlorotetrafluoride (**2a**) was also prepared from phenylsulfur trifluoride by the following procedure [S3].

$$PhSF_3 \xrightarrow[KF]{Cl_2} PhSF_4Cl\ (\mathbf{2a})$$

Chlorine (Cl_2) was passed with a flow rate of 34 mL/min into a stirred mixture of 5.00 g (30.1 mmol) of phenylsulfur trifluoride and 8.74 g (150 mmol) of spray-dried KF in 20 mL of dry acetonitrile at 6–9 °C. Chlorine was passed for 43 min and the total amount of chlorine passed was 1.47 L (65.5 mmol). The reaction mixture was filtered in dry air or nitrogen. After removal of the solvent in vacuum, 5.62 g (84%) of *trans*-phenylsulfur chlorotetrafluoride was obtained as a colorless liquid.

3. Preparation of aryl bis- and tris(sulfur chlorotetrafluorides), $Ar(SF_4Cl)_n$ (n = 2 and 3)

$$Ar(SH)_n \xrightarrow[KF]{Cl_2} Ar(SF_4Cl)_n \quad n = 2, 3$$

A typical procedure [S5]: A 500 mL fluoropolymer (PFA) reactor was set up with a magnetic stirrer, a gas (N_2, Cl_2) inlet tube, and a gas outlet tube protected by a $CaCl_2$ tube. Gas flow was controlled by a digital controller and measured by a digital integrator. The vessel was charged with dry KF (100 g, 1.72 mol), and set up for reaction under nitrogen flow. Anhydrous acetonitrile (300 mL) was added, followed by 1,3-benzenedithiol (9.78 g, 68.7 mmol). After cooling in the ice bath with stirring under N_2 flow for 1 hour, N_2 was stopped and then chlorine gas was introduced below the surface at 60–80 mL/min with vigorous stirring. Over approximately 6 h, a total of 27.1 L (1.21 mol) of Cl_2 was added. The reaction was then allowed to come to room temperature with stirring. After being stirred for two days at room temperature, the reaction mixture was filtered and washed through with dry acetonitrile (200 mL). The solvent was then removed at room temperature under vacuum, leaving the crude product (23.2 g, crude yield 93%) as a white solid, which was recrystallized from pentane in a freezer to give white crystals of 1,3-bis(chlorotetrafluorosulfanyl)benzene (**2p’**) (14.1 g, yield 56%). To obtain a sample for analysis, some of the crystals were further recrystallized. The reaction conditions and results for each of the $Ar(SF_4Cl)_n$ are shown in Table 2 (Text). The physical and spectral data of the products are shown below.

Phenyl-1,3-bis(sulfur chlorotetrafluoride) (**2p'**) [S5]: mp 82–83.5 °C (in a sealed capillary); ^{1}H NMR ($CDCl_3$) δ 8.12 (t, *J* = 2.0 Hz, 1H, 2-H), 7.89 (dd, *J* = 8.3, 2.0 Hz, 2H, 4,6-H), 7.58 (t, *J* = 8.3 Hz, 1H, 5-H); ^{19}F NMR ($CDCl_3$) δ 136.14 (s, SF_4); ^{13}C NMR ($CDCl_3$) δ 155.1 (quintet, *J* = 20.6 Hz), 129.3, 129.0 (t, *J* = 4.3 Hz), 124.1 (quintet, *J* = 4.9 Hz). Anal. Calcd for $C_6H_4Cl_2F_8S_2$; C, 19.85%, H, 1.11%. Found, C, 20.30%, H, 1.20%. The NMR showed that product **2p'** obtained is a *trans,trans*-isomer.

Phenyl-1,4-bis(sulfur chlorotetrafluoride) (**2q'**) [S5]: mp 200.8–201.6 °C; ^{1}H NMR ($CDCl_3$) δ 7.84 (s, 4H); ^{19}F NMR ($CDCl_3$) δ 135.72 (s, 8F); ^{13}C NMR ($CDCl_3$) δ 156.9 (quintet, *J* = 20.2 Hz), 126.7 (m). Anal. Calcd for $C_6H_4Cl_2F_8S_2$; C, 19.85%, H, 1.11%. Found, C, 19.71%, H, 1.10%. NMR analysis showed that product **2q'** obtained is a *trans,trans*-isomer.

5-Bromobenzene-1,3-bis(sulfur chlorotetrafluoride) (**2r'**) [S5]: The product having all *trans*-configuration was isolated by recrystallization from chloroform: mp 146.1–148 °C (with decomposition); ^{1}H NMR ($CDCl_3$) δ 8.05 (m, 1H), 8.02 (m, 2H); ^{19}F NMR ($CDCl_3$) δ 135.76 (s); ^{13}C NMR ($CDCl_3$) δ 155.6 (quintet, *J* = 21.7 Hz), 132.2 (m), 122.8 (m), 122.3. Anal. Calcd for $C_6H_3BrCl_2F_8S_2$; C, 16.30%, H, 0.68%. Found, C, 16.35%, H, 0.72%. The formation of the minor products having *cis*-configuration at SF_4Cl was confirmed by ^{19}F NMR of the crude product: ^{19}F NMR ($CDCl_3$) δ 159 (m, 1F), 102 (m, 2F), 69 (m, 1F).

1,4-Difluorophenyl-2,5-bis(sulfur chlorotetrafluoride) (**2s'**) [S5]: The product was isolated by washing the crude product with a small amount of dichloromethane: mp 161.5–162.9 °C (a 97:3 mixture of *trans* and *cis* configuration); ^{1}H NMR ($CDCl_3$) (a 97:3 mixture of *trans* and cis configuration) δ 7.63 (m, 2H); ^{19}F NMR ($CDCl_3$) (a 97:3 mixture of *trans* and *cis* configuration) δ 155.63 (m, *cis*-SF), 138.52 (m, *trans*-SF_4), 112.95 (m, *cis*-SF_2), 71.37 (m, *cis*-SF), −109.25 (m, CF); ^{13}C NMR ($CDCl_3$) (a 97:3 mixture of *trans* and *cis* configuration) δ 150.8 (d, *J* = 263.3 Hz), 143.9 (m), 118.5 (m). Anal. (a 97:3 mixture of *trans* and *cis* configuration) Calcd for $C_6H_2Cl_2F_{10}S_2$; C, 18.06%, H, 0.51%. Found, C, 18.01%, H, 0.51%.

2,3,5,6-Tetrafluorophenyl-1,4-bis(sulfur chlorotetrafluoride) (**2t'**) [S5]: The product was recrystallized from dichloromethane/pentane; mp 135–141 °C (a 78:22 mixture of *trans* and *cis* configuration); ^{19}F NMR ($CDCl_3$) (a 78:22 mixture of *trans* and *cis* configuration) δ 148.6–150.4 (m, *cis*-SF), 142.22 (m, *trans*-SF_4), 122.9–124.2 (m, *cis*-SF_2), 77.7–79.2 (m, *cis*-SF), −129.2 to −130.3 (m, CF, *trans* and *cis*); ^{13}C NMR ($CDCl_3$) (a 63:37 mixture of *trans* and *cis* configuration) δ 143.7–144.7 (m), 140.2–141.2 (m), 134.5 (m). Anal. (a 78:22 mixture of *trans* and *cis* configuration) Calcd for $C_6Cl_2F_{12}S_2$; C, 16.56%, H, 0.00%. Found, C, 16.54%, H, <0.05%.

Phenyl-1,3,5-tris(sulfur chlorotetrafluoride) (**2u'**) [S5]: The product [mp 140–150 °C (sealed capillary)] having all *trans*-configuration at SF_4Cl was obtained by recrystallization of the mixture twice from pentane, but it was contaminated with a small amount of impurity, presumably compounds having SO_2F and SOF, and further purification failed. The isomer having all *trans* configuration at SF_4Cl was identified: ^{1}H NMR ($CDCl_3$) δ 8.24 (s); ^{19}F NMR ($CDCl_3$) δ 135.30 (s); ^{13}C NMR ($CDCl_3$) δ 127.0 (m), 155.0 (quintet, *J* = 22.9 Hz). The products having *cis* configuration at SF_4Cl were identified by ^{19}F NMR: ^{19}F NMR ($CDCl_3$) δ 158 (m, 2F of *cis*-SF_4Cl), 103 (m, 1F of *cis*-SF_4Cl), 69 (m, 1F of cis-SF_4Cl). We failed to get a pure sample of **2u'** which satisfied elemental analysis: Found; C, 15.10%; H, 0.61% (Calcd for $C_6H_3Cl_3F_{12}S_3$: C, 14.25%; H, 0.60%). However, **2u'** was derived to **3u"** which provided a satisfactory elemental analysis.

4. Preparation of arylsulfur pentafluorides ($ArSF_5$) from $ArSF_4Cl$

4-1. Examination on the reactivity of $PhSF_4Cl$ to different fluorides

$PhSF_4Cl$ (**2a**) $\xrightarrow{\text{Fluoride}}$ $PhSF_5$ (**3a**)

Phenylsulfur chlorotetrafluoride (**2a**) was treated with a different fluoride as shown in Table 3 in the text. For BF_3 (gas) (Runs 1-2), the reaction was carried out in the following way [S3].

Run 1: A steel reactor (25–30 mL) was charged with 1.0 g (4.5 mmol) of *trans*-**2a** and cooled in a dry ice-acetone bath. The reactor was then evacuated by a vacuum pump and BF_3 gas was introduced into the reactor until the pressure reached 18 psi. The reaction mixture (reactor) was warmed to room temperature and stood for three days. After that, the reactor was opened and the reaction mixture was analyzed. All of the starting material became a solid residue.

Run 2: A steel reactor (25–30 mL) was charged with 1.42 g (6.4 mmol) of *trans*-**2a** and 6.4 mL of dry dichloromethane and cooled to around −100 °C with a liquid N_2 bath. The reactor was evacuated by a vacuum pump and BF_3 gas was introduced into the reactor until the pressure reached 80 psi. The reaction mixture (reactor) was warmed to room temperature and stood for 5 h. In the meantime, additional BF_3 was added until the pressure reached 100 psi. After the reaction, the reactor was opened and the reaction mixture was analyzed. ^{19}F NMR showed that the yield of **3a** was 28%.

For Runs 3–11, the reaction was carried out as shown in the following typical procedure [S3].

A typical procedure: In a dry box, a reaction vessel made of fluoropolymer was charged with 1.0 g (4.54 mmol) of *trans*-**2a** and 0.26 g (1.4 mmol) of dry SnF_4. The reaction vessel was brought out from the dry box and equipped with a rubber balloon filled with N_2. The mixture was stirred at 80 °C for 2 h. The analysis of the reaction mixture by ^{19}F-NMR showed that product **3a** was produced in 34% yield.

The results with other different fluorides are shown together with reaction conditions in Table 3 in the text.

4-2. Preparation of various $ArSF_5$ from $ArSF_4Cl$ with ZnF_2

$$ArSF_4Cl \xrightarrow[\text{under a } N_2 \text{ balloon}]{ZnF_2} ArSF_5$$

A typical procedure [S3]: A 100 mL fluoropolymer (TEFLON®·PFA) vessel was charged with $PhSF_4Cl$ (**2a**) (44 g, 0.2 mol) and dry ZnF_2 (12.3 g, 0.12 mol) in a dry box filled with N_2. The vessel was brought out from the dry box and equipped with a condenser made of fluoropolymer and a balloon filled with N_2. The reaction mixture was slowly heated to 120 °C over a period of one hour. The reaction mixture changed from colorless to yellow, pink, and then eventually green. The reaction mixture was stirred at 120 °C for 20 h. After being cooled to room temperature, about 50 mL of pentane was added to the reaction mixture. The mixture was filtered to remove all insoluble solid to give a yellow solution, which was concentrated. The resulting residue was distilled at reduced pressure to give 30.6 g (75%) of $PhSF_5$ (**3a**); bp 70–71 °C/120 mmHg. The reaction conditions and results for other $ArSF_4Cl$ are shown in Table 4 (Text). The physical and spectral data of new compounds are shown below.

***o*-Fluorophenylsulfur pentafluoride (3e)** [S3]: bp 75 °C/27 mmHg; ^{1}H NMR ($CDCl_3$) δ 7.78–7.73 (m, 1H), 7.55–7.48 (m, 1H), 7.27–7.17 (m, 2H); ^{19}F NMR ($CDCl_3$) δ 81.77 (quintet, J = 150 Hz, 4F, SF_4), 68.06 (dd, J = 150, 26 Hz, 1F, SF), −108.50 (m, 1F, CF); ^{13}C NMR ($CDCl_3$) δ 156 (d, J = 260 Hz), 140 (m), 134 (m), 129 (m), 124 (m), 118 (d, J = 24 Hz); IR (neat) 2360, 1596, 1489, 1452, 1276, 1244, 846, 811, 759, 641, 599, 584, 497 cm^{-1}; GC-Mass *m*/*z* 222 (M^+). Anal. Calcd for $C_6H_4F_6S$; C, 32.44%, H, 1.81%. Found; C, 32.51%, H, 1.95%.

2,6-Difluorophenylsulfur pentafluoride (3k) [S3,4]: mp 40.3–41.1 °C; ^{1}H NMR ($CDCl_3$) δ 7.51 (m, 1H), 7.04 (m, 2H); ^{19}F NMR ($CDCl_3$) 82.32–80.69 (m, 1F, SF), 62.76 (d, 4F, SF_4); ^{13}C NMR ($CDCl_3$) δ 157 (d, J = 262 Hz), 134 (t, J = 23 Hz), 129 (m), 113 (d, J = 28 Hz); IR (nujol) 1616, 1593, 1568, 1295, 1241, 1222, 1112, 1054, 1010, 876, 843, 790, 756, 720, 684, 608, 588, 554, 542, 516 cm^{-1}; GC-Mass *m*/*z* 240 (M^+). HRMS; found 239.984509 (calcd for $C_6H_3F_7S$; 239.984370). Anal. Calcd for $C_6H_3F_7S$; C, 30.01%, H, 1.26%. Found, C, 30.20%, H, 1.47%.

4-3. Effect of the atmosphere on the reaction of $PhSF_4Cl$ with ZnF_2

Reactions of phenylsulfur chlorotetrafluoride with ZnF_2 were conducted under no flow of N_2 gas (Run 1), a slow flow (Run 2) and a fast flow (Run 3) of N_2 gas, and a flow of Cl_2 gas (Run 4).

$$PhSF_4Cl \xrightarrow[120\ °C]{ZnCl_2} PhSF_5$$

Run 1; no flow of N_2 (pressure of a N_2 balloon)
Run 2; slow flow of N_2
Run 3; fast flow of N_2
Run 4; flow of Cl_2

The prodedure for each of Runs 1–4 is shown below [S3].

[Run 1] In a dry box, a reaction vessel made of fluoropolymer was charged with 1.0 g (4.54 mmol) of *trans*-phenylsulfur chlorotetrafluoride (**2a**) and 0.28 g (2.7 mmol) of anhydrous ZnF_2. The reaction vessel was removed from the dry box and equipped with a rubber ballon filled with N_2 gas. The reaction mixture was stirred at 120 °C for 4 h. After being cooled to room temperature, the reaction mixture was analyzed by ^{19}F NMR. The reaction was completed. The results are shown in Table S-1.

[Run 2] In a dry box, a 50 mL reaction vessel made of fluoropolymer was charged with 10.0 g (45.4 mmol) of *trans*-**2a** and 2.8 g (27 mmol) of anhydrous ZnF_2. The reaction vessel was removed from the dry box, and equipped with a condenser made of fluoropolymer and connected to a N_2 gas flowing device. The reaction mixture was slowly heated to 120 °C with N_2 flowing at a rate of 5.4 mL/min. The reaction mixture was stirred at 120 °C with the N_2 flowing for 5 h. After being cooled to room temperature, the reaction mixture was analyzed with ^{19}F NMR. The reaction was not completed. The results are shown in Table S-1.

[Run 3] In a dry box, a 50 mL reaction vessel made of fluoropolymer was charged with 10.0 g (45.4 mmol) of *trans*-**2a** and 2.8 g (27 mmol) of anhydrous ZnF_2. The reaction vessel was removed from the dry box, and equipped with a condenser made of fluoropolymer and connected to a N_2 gas flowing device. The reaction mixture was slowly heated to 120 °C with N_2 flowing at the rate of 26.9 mL/min. The reaction mixture was stirred at 120 °C with the N_2 flowing for 5 h. After being cooled to room temperature, the reaction mixture was analyzed with ^{19}F NMR. The reaction was not completed. The results are shown in Table S-1.

[Run 4] In a dry box, a 50 mL reaction vessel made of fluoropolymer was charged with 10.0 g (45.4 mmol) of *trans*-**2a** and 2.8 g (27 mmol) of anhydrous ZnF_2. The reaction vessel was removed from the dry box, and equipped with a condenser made of fluoropolymer and connected to a Cl_2 gas flowing device. The reaction mixture was slowly heated to 120 °C with Cl_2 flowing at a rate of 4.6 mL/min. The reaction mixture was stirred at 120 °C with the Cl_2 flowing for 1.7 h. After being cooled to room temperature, the reaction mixture was analyzed with ^{19}F NMR. The reaction was completed. The results are shown in Table S-1.

Table S-1: Atmospheric Effect on Reaction of $PhSF_4Cl$ with ZnF_2.

$$\underset{\mathbf{2a}}{PhSF_4Cl} + ZnF_2 \xrightarrow[120\ °C]{\text{Atmosphere}} \underset{\mathbf{3a}}{PhSF_5}$$

Run	Atmosphere	Time(h)	Conversion[1]	Y(%)/**3a**[1]
1	no flow of N_2[2]	4	100%	88
2	slow flow of N_2	5	85%	67
3	fast flow of N_2	5	51%	38
4	Cl_2 gas	1.7	100%	92

1) Determined by ^{19}F NMR. 2) Under a pressure ofa N_2 balloon.

The results showed that the reaction of **2a** with ZnF_2 became slow as the flow rate of N_2 gas increased and that the reaction was fast and its yield was high under the Cl_2 atmosphere.

4-4. Large-scale preparation of $PhSF_5$ from $PhSF_4Cl$ with ZnF_2 in Cl_2 atmosphere

$PhSF_5$ (**3a**) was prepared in a large scale according to the following procedure.

$$\underset{\mathbf{2a}}{PhSF_4Cl} \xrightarrow[Cl_2\ \text{atmosphere}]{ZnF_2} \underset{\mathbf{3a}}{PhSF_5}$$

A 1 liter three-neck reactor (made of fluoropolymer FEP) was charged with anhydrous zinc fluoride (ZnF_2; 162.8 g, 1.58 mol) in a dry box filled with N_2. The reactor was taken out of the dry box and set up with a condenser (made of fluoropolymer), a dropping funnel (made of fluoropolymer), a thermometer (protected with a fluoropolymer), a T-shaped gas inlet connector, and a T-shaped gas outlet connector (this gas outlet connector was equipped at the top of the condenser). The gas line (N_2 and Cl_2) was connected to the reactor through the T-shaped inlet connector, and the last one of three ports of the T-shaped inlet connector was directly connected to one of three ports of the T-shaped gas outlet connector. The last one of three ports of the T-shaped gas outlet connector was connected to a solution of an aqueous sodium sulfite solution for neutralization of the discharged Cl_2 gas. All the tubing and connectors were made of fluoropolymer. Phenylsulfur pentafluoride (**3a**) (180 g, 120 mL) as a solvent was added into the reactor. The reactor was purged with N_2 and then Cl_2 was flowed into the reactor at 200 mL/min flow rate for 10 min. After that, Cl_2 was flowed at 100 mL/min into both the reactor and the gas outlet by opening the line to the gas outlet connector (*Note*: the reactor was under the pressure of Cl_2 flow of 100 mL/min). The discharged Cl_2 gas was neutralized through the aq. sodium sulfite solution. The reactor was then put in an oil bath and heated to 110 °C (oil bath temperature) under stirring with a magnetic stirrer. *trans*-Phenylsulfur chlorotetrafluoride (**2a**) [662 g, 2.85 mol,

purity 95%: Note; impurity (5%) was phenylsulfur trifluoride] was dropwise added to the stirred mixture through the dropping funnel for 72 min. During the addition, the reaction temperature (inside) was mostly 105–101 °C. After the additon, the reaction mixture was stirred for 30 min at 110 °C (oil bath temperature) and then for 3 h at 115 °C (oil bath temperature). After the reaction, the Cl_2 flow was stopped and N_2 flow was started, and the oil bath was removed and the reactor was left at room temperature to cool. ^{19}F NMR analysis at this moment showed that the reaction was completed. The cooled reaction mixture was filtered with 600 mL of dichloromethane and the filtrate was mixed with 300 mL of a cooled aq. 10% KOH solution. The mixture was stirred for 1 hour and the organic layer was separated, washed with an aq. NaCl solution (300 mL × 2), dried over anhydrous magnesium sulfate, and filtered. The filtrate was concentrated under reduced pressure (30 °C, 100 mmHg) and the resulting residue was distilled under reduced pressure to give 658 g of phenylsulfur pentafluoride (**3a**), bp 67–71 °C/26–29 mmHg). The real yield was (658 g −180 g =) 478 g (yield 82%). Its purity was >99% (by GC).

4-5. Preparation of *p*-$ClC_6H_4SF_5$ from *p*-$ClC_6H_4SF_4Cl$ with $ZnF_2/ZnCl_2/AlCl_3$ (100/10/5)

The experimental procedure is shown below.

A 125 mL fluoropolymer reactor with a magnetic stir bar was charged with anhydrous ZnF_2 (10.9 g, 105 mmol), anhydrous $ZnCl_2$ (1.4 g, 10 mmol), and $AlCl_3$ (0.7 g, 5 mmol) in a dry box filled with N_2 and then the reactor was taken from the dry box. Dry dichloromethane (5 mL) was added to the reactor and the mixture was stirred for 1 hour under N_2. Dichloromethane was removed by a vacuum pump and then the reactor was equipped with a condenser made of fluoropolymer connected to a T-shaped N_2 gas inlet. The reactor was purged with N_2 and *p*-chlorophenylsulfur chlorotetrafluoride (**2f**) [50.8 g, purity 90%, 180 mmol: Note; impurity (10%) was *p*-chlorophenylsulfur trifluoride] was added by syringe. Heating of the reaction mixture under N_2 to 100 °C (oil bath temperature) was started. It took 15 min for the bath temperature to reach 100 °C. Heating of the reactor at the bath temperature (100 °C) was continued for 20 h. The inside temperature rose to 114 °C (maximum) in 30 min and fell to 100 °C in 60 min. After the reaction, the reaction mixture was cooled to room temperature and filtered with dichloromethane (80 mL). The combined filtrate was poured into a 10% aq. KOH solution (100 mL) cooled in an ice bath and the mixture was stirred for 30 min. The organic layer was separated, washed with aq. NaCl solution two times, dried over anhydrous magnesium sulfate, and filtered. The filtrate was concentrated at 30 °C under 100 mmHg and the resulting residue was distilled under reduced pressure to give 30.3 g (yield 70%) of product *p*-chlorophenylsulfur pentafluoride (**3f**); bp 61–64 °C/9.8 mmHg, purity 99.5% by GC.

5. Preparation of $ArSF_5$ from $ArSF_4Cl$ with anhydrous hydrogen fluoride

$$ArSF_4Cl \xrightarrow[(KHF_2)]{HF} ArSF_5$$

Caution! Anhydrous hydrogen fluoride (bp 19 °C) is very toxic. The following experiment should be carried out in an efficient fume hood, and the experimenters should be familiar with the precautions necessary for safe handling of anhydrous hydrogen fluoride (For example, see Safetygram #29 on the Website of Air Products and Chemicals, Inc.).

A typical procedure: While N_2 gas was flowed through a 250 mL fluoropolymer (FEP) vessel set with a condenser (made of fluoropolymer), the vessel was cooled in a bath at –20 °C. A coolant (−15 °C) was flowed through the condenser. The vessel cooled to –20 °C was charged with 48 g (2.4 mol) of anhydrous hydrogen fluoride. Into the vessel, 8.6 g (0.11 mol) of potassium hydrogen difluoride (KHF_2) was added. While the vessel was warmed to +12 °C, 22.1 g (96.2 mmol) of *trans*-phenylsulfur chlorotetrafluoride (**2a**) [purity 96 wt %: Note; impurity (4 wt %) was phenylsulfur trifluoride] was added over 1 hour by a syringe pump. The molar ratio of **2a**/hydrogen fluoride/KHF_2 was 1/25/1.1. The reaction mixture was then stirred at 15 °C for 18 h. After that, the reaction mixture was warmed to 25 °C and hydrogen fluoride was removed at that temperature under atmospheric pressure. The residue was neutralized with 15% aqueous KOH and extracted with dichloromethane. The organic layer was separated, dried over anhydrous magnesium sulfate, and filtered. The filtrate was concentrated by distilling the solvent at 75 °C under atmospheric pressure. The resulting residue was distilled under reduced pressure (35 mmHg and bath temperature about 106–140 °C) to give 14.4 g (yield 73%) of phenylsulfur pentafluoride (**3a**). The purity of the product was determined to be 100 % by GC analysis. The product was identified by spectral comparison with an authentic sample.

The reaction conditions and results for the fluorinations with anhydrous hydrogen fluoride are shown in Table 5 (Text).

6. Preparation of $PhSF_5$ from $PhSF_4Cl$ with 70 wt % HF-pyridine

Under nitrogen, phenylsulfur chlorotetrafluoride (**2a**) (32.4 g, 141 mmol) [purity 96 wt %; the impurity (4 wt %) was phenylsulfur trifluoride] was added over 1 hour by syringe pump into a stirred liquid (38.2 mL) of 70 wt % HF-pyridine (available from Sigma-Aldrich) in a fluoropolymer vessel heated in an oil bath at 55 °C. After the addition, the reaction mixture was stirred for an additional 2 h at that temperature (55 °C) and cooled to room temperature. ^{19}F NMR of the reaction mixture showed that the starting material **2a** was consumed. The reaction mixture was slowly poured into a stirred, cooled 25% aq. KOH solution (KOH, 104 g). The resulting alkaline solution was extracted with

dichloromethane and the organic layer was separated, washed with water, 2 M aq. HCl, and water, dried over anhydrous magnesium sulfate, and filtered. The filtrate was concentrated at atmospheric pressure and then distilled at reduced pressure to give 18.1 g (63%) of phenylsulfur pentafluoride (**3a**); bp 62 °C/44 mmHg. Its purity was determined to be 100% by GC.

As another experiment, 32.4 g (141 mmol) (purity 96 wt %) of **2a** was mixted with 38.2 mL of 70 wt % HF-pyridine in a fluoropolymer vessel at room temperature under nitrogen, and the stirred mixture was heated at 50 °C (oil bath temperature) for 6 h. The mixture was then cooled to room temperature. The reaction mixture was treated in a similar way as above and 16.4 g (57%) of product **3a** was obtained after the distillation.

7. Preparation of polyfluorinated $ArSF_5$ with SbF_3/SbF_5 or $SbCl_5$

$$(F)_nArSF_4Cl \xrightarrow{SbF_3/SbF_5(SbCl_5)} (F)_nArSF_5$$

A typical procedure [S4]: A 100 mL fluoropolymer (PFA) reactor with a magnetic stir bar was charged with SbF_5 (6.59 g, 30.4 mmol), Fluorinart® FC-72 (40 mL), and SbF_3 (5.72 g, 32 mmol) in a dry box, and then transferred to a fume hood. Pentafluorophenylsulfur chlorotetrafluoride (**2o**) (9.41 g, 30.3 mmol) was then added by syringe slowly at room temperature with vigorous stirring. The mixture was stirred at room temperature for 1 hour and quenched with potassium fluoride (powder). The reaction mixture was then filtered and fractional distillation of the filtrate was carried out to give 6.24 g (71%) of pentafluorophenylsulfur pentafluoride (**3o**).

The reaction conditions and results for products **3l–n** are shown in Table 6 (Text). The boiling points and spectral data of products **3l–o** and spectral data of a byproduct, 3-chloro-2,4,6-trifluorophenylsulfur pentafluoride (see Text), are shown below. The byproduct was obtained as a mixture with product **3m** when **2m** was treated with SbF_5 alone.

2,3,6-Trifluorophenylsulfur pentafluoride (3l) [S4]: bp 154–156 °C; ^{1}H NMR ($CDCl_3$) δ 7.38 (m, 1H), 7.02 (m, 1H); ^{19}F NMR ($CDCl_3$) δ 75.5–77.6 (m, 1F), 72.6–73.6 (m, 4F), −110.0 (m, 1F), −127.8 (m, 1F), −138.0 (m, 1F); ^{13}C NMR ($CDCl_3$) δ 152.7 (d, *J* = 259.4 Hz), 147.6 (ddd, *J* = 249.3, 14.5, 4.3 Hz), 146.4 (ddt, *J* = 264.4, 16.6, 2.2 Hz), 130.3 (m), 120.6 (ddd, *J* = 19.5, 10.8, 1.4 Hz), 112.3 (dt, *J* = 26.7, 10.1 Hz); IR (neat) 3115, 1623, 1605, 1500, 1253, 1203, 1134, 1021, 917, 867, 808, 685, 609, 577, 548 cm^{-1}; GC-Mass *m/z* 258 (M^+). Anal. Calcd for $C_6H_2F_8S$; C, 27.92%, H, 0.78%. Found, C, 27.52%, H, 0.81%.

2,4,6-Trifluorophenylsulfur pentafluoride (3m) [S4]: bp 138–141 °C; ^{1}H NMR ($CDCl_3$) δ 6.80 (t, *J* = 8.6 Hz, 3,5-H); ^{19}F NMR ($CDCl_3$) δ 78.7–75.3 (m, SF), 73.8–72.9 (m, SF_4), −100.6 (m, 4-F),

−100.7 (m, 2,6-F) ; ^{13}C NMR ($CDCl_3$) δ 164.4 (dt, *J* = 257.9, 32.5 Hz), 158.4 (dm, *J* = 260.0 Hz), 126.2 (m), 101.9 (m); IR (neat) 3111, 1640, 1606, 1451, 1178, 1135, 1098, 1048, 1011, 870, 690, 625, 584, 556, 514 cm^{-1}; GC-Mass *m*/*z* 258 (M^+). Anal. Calcd for $C_6H_2F_8S$; C, 27.92%, H, 0.78%. Found, C, 27.75%, H, 0.84%.

2,3,4,6-Tetrafluorophenylsulfur pentafluoride (3n) [S4]: bp 141–142 °C; ^{1}H NMR ($CDCl_3$) δ 6.9–7.0 (m); ^{19}F NMR ($CDCl_3$) δ 75.1–77.3 (m, 1F), 73.3–74.3 (m, 4F), −107.5 (m, 1F), −124.1 (m, 1F), −125.0 (m, 1H), −160.6 (m, 1F); ^{13}C NMR ($CDCl_3$) δ 152.7 (dm, *J* = 259 Hz), 148.0 (dm, *J* = 265 Hz), 137.6 (dm, *J* = 253 Hz), 126.8 (m), 102.2 (m); GC-Mass *m*/*z* 276 (M^+). Anal. Calcd for C_6HF_9S; C, 26.10%, H, 0.37%. Found, C, 25.62%, H, 0.44%.

2,3,4,5,6-Pentafluorophenylsulfur pentafluoride (3o) [S3,4]: bp 135–137 °C; ^{19}F NMR ($CDCl_3$) δ 74.8 (m, 5F, SF_5), −133.4 (m, 2F, 2,6-F), −146.2 (m, 1F, 4-F), −158.6 (m, 2F, 3,5-F); ^{13}C NMR ($CDCl_3$) δ 143.6 (dm, *J* = 262 Hz), 137.9 (dm, *J* = 254 Hz), 126.7 (m); IR (neat) 1643, 1526, 1505, 1305, 1107, 1035, 1002, 874, 786, 685, 611, 574 cm^{-1}; GS-MS *m*/*z* 294 (M^+). HRMS; found 293.956492 (calcd for $C_6F_{10}S$; 293.956104).

3-Chloro-2,4,6-trifluorophenylsulfur pentafluoride [S3]: ^{1}H NMR ($CDCl_3$) δ 6.95 (br.t, *J* = 9.5 Hz, 5-H); ^{19}F NMR ($CDCl_3$) δ 78.7–75.3 (m, SF), 73.8–72.9 (m, SF_4), −101.3 (m, 2 or 6-F), −102.3 (m, 4-F), −102.6 (m, 2 or 6-F); GC-Mass *m*/*z* 294, 292 (M^+).

8. Preparation of aryl bis- and tris(sulfur pentafluorides), $Ar(SF_5)_n$ (n = 2, 3) with SbF_5

8-1. Preparation of aryl bis(sulfur pentafluorides)

$$Ar(SF_4Cl)_2 \xrightarrow{SbF_5} Ar(SF_5)_2$$

A typical procedure: A fluoropolymer (PFA) vessel was set up with a magnetic stirrer, septum, and nitrogen blanket, charged with 35 mL of dry dichloromethane, and cooled in a bath to approximately –25 °C. SbF_5 (1.5 mL, 21.4 mmol) was then added by syringe. After holding for 5–10 min, the clear golden solution was then cooled to –85 °C over 30 min. A solution of 3.92 g (10.8 mmol) of phenyl-1,3-bis(sulfur chlorotetrafluoride) (**2p’**) in 10 mL of dry dichloromethane was then added by syringe. Over 2.5 h the temperature of the stirred solution was allowed to rise to –25 °C, at which time the reaction was carefully quenched by adding KF (total 4 g) in portions. Cooling was removed, 2 g of celite was added to the mixture, and the mixture was stirred while being warmed to room temperature. After suction filtration, solvents were removed by evaporator and the residue was column chromatographed on silica gel (20 g) using pentane as an eluent to give 2.02 g (57%) of phenyl-1,3-

bis(sulfur pentafluoride) (**3p"**) as white crystals. The reaction conditions and results for others are shown in Table 7 (Text). The physical and spectral data of new compounds are shown below.

Phenyl-1,3-bis(sulfur pentafluoride) (3p") [S5]: mp 62.0–62.8 °C (in a sealed capillary); ^{1}H NMR ($CDCl_3$) δ 8.16 (t, *J* = 2.0 Hz, 1H, 2-H), 7.93 (dd, *J* = 8.2, 2.0 Hz, 2H, 4,6-H), 7.63 (t, *J* = 8.2 Hz, 1H, 5-H); ^{19}F NMR ($CDCl_3$) δ 81.88 (quintet, *J* = 150.4 Hz, 2F, 2xSF), 62.95 (d, *J* = 150.4 Hz, 8F, 2xSF_4); ^{13}C NMR ($CDCl_3$) δ 153.7 (quintet, *J* = 19.1 Hz), 129.5, 129.3 (t, *J* = 4.7 Hz), 124.3 (m); GC-Mass *m/z* 330 (M^+). Anal. Calcd for $C_6H_4F_{10}S_2$; C, 21.82%, H, 1.22%. Found, C, 21.79%, H, 1.52%.

Phenyl-1,4-bis(sulfur pentafluoride) (3q") [S5]: mp 108.8–109.7 °C; ^{1}H NMR ($CDCl_3$) δ 7.88 (s, 4H); ^{19}F NMR ($CDCl_3$) δ 81.68 (quintet, *J* = 150.3 Hz, 2F), 62.55 (d, *J* = 150.3 Hz, 8F); ^{13}C NMR ($CDCl_3$) δ 155.5 (quintet, *J* = 19.0 Hz), 126.9 (m); GC-Mass *m/z* 330 (M^+). Anal. Calcd for $C_6H_4F_{10}S_2$; C, 21.82%, H, 1.22%. Found, C, 21.59%, H, 1.23%.

5-Bromophenyl-1,3-bis(sulfur pentafluoride) (**3r"**) [S5]: mp 65.9–66.4 °C (in a sealed capillary); ^{1}H NMR δ ($CDCl_3$) 8.05–8.10 (m); ^{19}F NMR ($CDCl_3$) δ 80.49 (quintet, *J* = 151.2 Hz, 2F), 63.24 (d, *J* = 151.2 Hz, 8F); ^{13}C NMR ($CDCl_3$) δ 154.2 (quintet, J = 20.2 Hz), 132.4 (m), 123.0 (m), 122.5; IR (nujol) 1577, 1140, 867, 729, 662, 599, 574 cm^{-1}; GC-Mass *m/z* 410(M^+), 408 (M^+). Anal. Calcd for $C_6H_3BrF_{10}S_2$; C, 17.62%, H, 0.74%. Found, C, 17.61%, H, 0.73%.

2,5-Difluorophenyl-1,4-bis(sulfur pentafluoride) (**3s"**) [S5]: mp 73.0–74.6 °C; ^{1}H NMR ($CDCl_3$) δ 7.67 (m, 2H); ^{19}F NMR ($CDCl_3$) δ 76.3–78.5 (m, 2F), 67.71 (dd, *J* = 152.3, 24.8 Hz, 8F), −109.77 (m, 2F); ^{13}C NMR ($CDCl_3$) δ 151.1 (d, *J* = 262.3 Hz), 142.8 (m), 118.5 (dm, *J* = 26.0 Hz); GC-Mass *m/z* 366 (M^+). Anal. Calcd for $C_6H_2F_{12}S_2$; C, 19.68%, H, 0.55%. Found, C, 19.33%, H, 0.58%.

2,3,5,6-Tetrafluorophenyl-1,4-bis(sulfur pentafluoride) (**3t"**) [S5]: mp 129.2–130.0 °C; ^{19}F NMR ($CDCl_3$) δ 71.0–74.4 (m, 10F), −130.8 (m, 4F); ^{13}C NMR ($CDCl_3$/FC-72/CF_2ClCCl_2F=~5/2/3 v/v) δ 144.9 (m), 141.3 (m), 133.3 (m); GC-Mass *m/z* 402 (M^+). Anal. Calcd for $C_6F_{14}S_2$; C, 17.92%, H, 0.00%. Found, C, 17.99%, H, <0.05%.

8-2. Preparation of phenyl-1,3,5-tris(sulfur pentafluoride) (3u")

$$\underset{\textbf{2u'}}{1,3,5\text{-}Ph(SF_4Cl)_3} \xrightarrow{SbF_5} \underset{\textbf{3u''}}{1,3,5\text{-}Ph(SF_5)_3}$$

Procedure: A 500 mL fluoropolymer (PFA) jar (reactor) with septum, magnetic stirrer, nitrogen blanket, and cooling bath was set up and charged with 160 mL of dry dichloromethane. The reactor was cooled to –25 to –30 °C, and 67.5 mL (SbF_5, 99 mmol) of a solution of 17.4% (w/w) of SbF_5 in FC-72

was introduced by syringe over 8 min. After holding at this temperature for approximately 7 min, the reaction mixture was cooled to –95 °C over 15 min. A solution of 16.0 g (32 mmol) of phenyl-1,3,5-tris(sulfur chlorotetrafluoride) (**2u’**) in 50 mL of dry dichloromethane was introduced by syringe over 8 min as the temperature rose to –86 °C. The reaction mixture was allowed to warm to +7 °C over 6 h, at which time the reaction was quenched with the addition of KF (10 g) in portions over 20 min. Celite (5 g) was added, and the mixture was suction filtered. Solvents were removed by atmospheric fractional distillation to 70 °C leaving the crude residue as a yellow slush. The crude product was triturated with a minimum volume of ether and filtered to give a solid, which was then dried at room temperature in vacuum to give 8.03 g (55%) of product **3u”**. The physical and spectral data of **3u”** are shown below.

Phenyl-1,3,5-tris(sulfur pentafluoride) (**3u”**) [S5]: mp 132.8–133.5 °C (lit. [S6] 126–127 °C); ^{1}H NMR ($CDCl_3$) δ 8.31 (s); ^{19}F NMR ($CDCl_3$) δ 63.31 (dm, J = 151.8 Hz, 12F), 78.3–80.5 (m, 3F); ^{13}C NMR ($CDCl_3$) δ 153.7 (quintet, J = 21.3 Hz), 127.3 (m); GC-Mass m/z 456 (M^+). Anal. Calcd for $C_6H_3F_{15}S_3$; C, 15.79%, H, 0.66%. Found, C, 15.48%, H, 0.62%.

9. DSC measurement of arylsulfur chlorotetrafluorides

For DSC analysis, three types of cells were used as shown below.

Cell 1, a cell made of gold-coated stainless steel

Cell 2, a cell made of stainless steel

Cell 3, a cell made of stainless steel with a cap made of gold-coated copper

The analysis using Cells 1 and 2 was conducted by Kayaku Japan Ltd. with Rigaku DSC8230 instrument. We conducted the analysis using Cell 3 with Perkin Elmer Pyris 1. Samples were heated at 5 or 10 °C/min. The results are shown in Table S-2.

Table S-2: Results of DSC measurement of Arylsulfur Chlorotetrafluorides.

Run	$ArSF_4Cl$	Decomposition[a] (–ΔH[b])		
		Cell 1	Cell 2	Cell 3
		Gold (on SS)[c,d]	SS[d,e]	SS/Gold (on Cu)[f]
1	**2a**	131 °C (347 J/g)	190 °C (361 J/g)	147 °C (505 J/g)
2	**2b**	139 °C (343 J/g)	185 °C (497 J/g)	140 °C (401 J/g)
3	**2c**	–	–	129 °C (380 J/g)
4	**2d**	139 °C (291 J/g)	197 °C (189 J/g)	–
5	**2f**	–	–	159 °C (336 J/g)
6	**2g**	–	–	167 °C (322 J/g)
7	**2i**	–	–	187 °C (506 J/g)
8	**2j**	–	–	179 °C (236 J/g)

[a]Decomposition onset temperture. [b]Exotherm. [c]Gold-coated stainless steel (SS) cell was used. [d]DSC data was measured by Kayaku Japan, Ltd. Samples were heated at 10 °C/min. [e]A stainless steel (SS) cell was used. [f]A cell made of stainless steel (SS) with a cap made of gold-coated copper was used. Samples **2a**, **2f**, **2i** and **2j** were heated at 10 °C/min and **2c** and **2g** were heated at 5 °C/min.

Phenylsulfur chlorotetrafluoride (**2a**), *p*-methylphenylsulfur chlorotetrafluoride (**2b**), *p-tert*-butylphenylsulfur chlorotetrafluoride (**2c**), *p*-fluorophenylsulfur chlorotetrafluoride (**2d**), *p*-chlorophenylsulfur chlorotetrafluoride (**2f**), *p*-bromophenylsulfur chlorotetrafluoride (**2g**), *p*-nitrophenylsulfur chlorotetrafluoride (**2i**), and *p*-(methanesulfonyl)phenylsulfur chlorotetrafluoride (**2j**) were used for the measurement. Crystalline **2c**, **2g**, **2i**, and **2j** were pure samples obtained by crystallization. Liquid **2a**, **2b**, **2d**, and **2f** (obtained by distillation) used were contaminated by the corresponding arylsulfur trifluoride (< ~10%). As the decomposition temperatures of these arylsulfur trifluorides are very high, at more than 270 °C [S7], it may be reasonable that the results obtained for **2a**, **2b**, **2d**, and **2f** were not influenced by the impurities, i.e., the arylsulfur trifluorides.

As seen from Table S-2, the decomposition onset temperature by DSC varied depending on the material of the cell. For example, DSC showed that **2a** started to decompose at 190 °C in Cell 2 made of stainless steel (SS), but at 131 °C in Cell 1 made of gold-coated SS. It was a surprise that **2a** decomposed at a temperature (131 °C), with much less reactive gold, which was much lower than that (190 °C) with reactive SS. This means that **2a** reacted with both gold and SS metals of the cells. The observed higher decompostion temperature of SS than gold could probably be a matter of solid surface. The real thermal decomposition temperature of **2a** should be higher than at least 190 °C, which was measured with the SS cell, rather than 131 °C measured with the gold-coated SS. This is in accordance with the fact that **2a** was stable for 48 h at 150 °C in a Teflon tube that is inactive (see the Text). *Thus, it has been concluded that any data (Table S-2) obtained by the DSC measurement did not provide the real thermal decomposition data of arylsulfur chlorotetrafluorides.*

10. Preparation of starting materials 1j–n and 1r–u

10-1. Preparation of bis[4-(methanesulfonyl)phenyl] disulfide (1j)

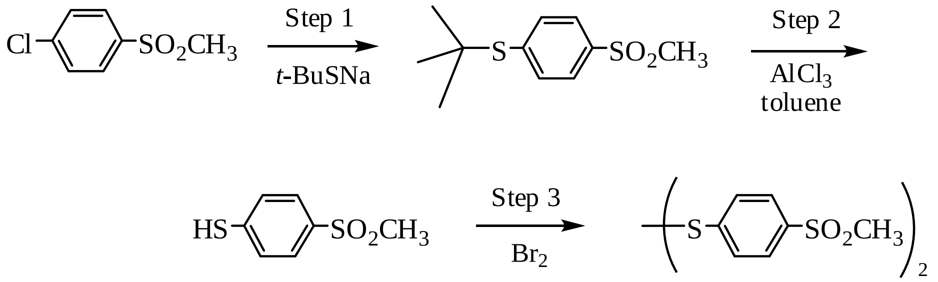

1j

Step 1: A 50 mL single-neck flask with magnetic stirrer and septum was flushed with nitrogen, and charged with sodium *tert*-butylthiolate (6.95 g, 62 mmol), dry *N*-methylpyrrolidinone (12 mL), and 4-chlorophenyl methyl sulfone (9.53 g, 50 mmol). The mixture was heated to 50 °C. An exothermic reaction occurred. GC/MS analysis verified complete conversion to the product in 2 h. The reaction mixture was quenched into dilute aq. HCl solution and a mixture of hexane and dichloromethane was added to the mixture. The organic layer was separated and stripped to yield an oil. This residue was then slurried in hexane plus water, and the precipitated solids were filtered and washed through water followed by hexane. The solids were then taken up in dichloromethane and the solution was dried over anhydrous magnesium sulfate, filtered and stripped to yield 10.5 g (93%) of 4-(*tert*-butylthio)phenyl methyl sulfone as white crystals: ^{1}H NMR ($CDCl_3$) δ 7.86–7.89 (d, 2H), 7.69–7.72 (d, 2H). 3.07 (s, 3H), 1.33 (s, 9H); GC-Mass *m*/*z* 244 (M^+). Note: 4-(*tert*-butylthio)phenyl methyl sulfone is poorly soluble in hexane.

Step 2: A 500 mL single-neck flask was flushed with nitrogen, then charged with 4-(*tert*-butylthio)phenyl methyl sulfone (29.6 g, 0.12 mol) obtained by step 1, followed by dry, deoxygenated toluene (200 mL), and finally aluminium chloride (21.7 g, 0.16 mol). The reaction mixture was stirred at room temperature for 4 h under nitrogen. The reaction was quenched with aq. dilute HCl solution and a small amount of ether. The organic layer was separated and the aq. layer was extracted with hexane. The combined organic layer was washed with water and dried over anhydrous sodium sulfate, and filtered. The solvent of the filtrate was evaporated with a fractional column at elevated temperature (160 °C max) under atmospheric pressure, leaving a biphase oil, which solidified out upon brief freezing and agitation. The oily layer consisting of *tert*-butylated toluenes was removed by decantation. The solid was slurried in pentane, collected by filtration and dried in vacuum at room temperature, yielding 20.7 g (91%) of 4-(methanesulfonyl)thiophenol [S8] as a fine white powder: ^{1}H NMR ($CDCl_3$) δ 7.77 (dm, J = 8.6 Hz, 2H), 7.39 (dm, J = 8.6 Hz, 2H), 3.71 (s, 1H), 3.02 (s, 3H); GC-Mass m/z 188 (M^+).

Step 3: A flask was charged with 4-(methanesulfonyl)thiophenol (10.5 g, 56 mmol), dichloromethane (40 mL), heptane (70 mL), and acetic acid (30 mL). The flask was placed in an ambient water bath. Bromine (Br_2) (1.6 mL, 31.1 mmol) was then added in portions by syringe over a few minutes. Reaction was immediate and exothermic, the bromine color persisted at the end, and a thick precipitate immediately formed. After stirring for 3 h, the solvent was evaporated in vacuum. Ether and hexane were then added to the residue and the precipitate was filtered, washed with pentane, and dried in vacuum to give 10.1 g (97%) of bis[4-(methanesulfonyl)phenyl] disulfide (**1j**) [S9]: ^{1}H NMR ($CDCl_3$) δ 7.88 (d, J = 8.5 Hz, 4H), 7.65 (d, J = 8.5 Hz, 4H), 3.04 (s, 6H); ^{13}C NMR ($CDCl_3$) δ 143, 139, 128, 127, 45; IR (nujol) 1579, 1299, 1150, 1089, 1070, 964, 826, 774, 730, 567, 529 cm^{-1}.

10-2. Preparation of polyfluorinated diaryl disulfides 1k–n

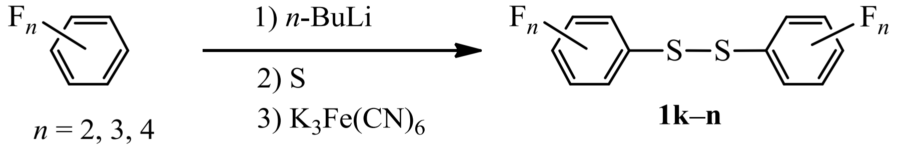

A typical procedure: A two-liter three-neck flask was set up with magnetic stirring, chiller, N_2 flow, thermometer, and condenser. It was charged with 1,3,5-trifluorobenzene (97.8 g, 0.74 mol) and dry THF (800 mL), and cooled to –40 °C under nitrogen. n-Butyllithium (1.6 N in hexane) (463 mL, 0.74 mol) was then added in portions by syringe over several hours, with the temperature maintained below –30 °C, after which it was stirred for an additional half hour at –30 °C. Powdered elemental sulfur (23.7 g, 0.74 mol) was then added in portions over 1–2 h, with the temperature maintained below –30 °C. The solution became clear yellow. It was then allowed to room temperature with stirring under

nitrogen overnight. The flask was then transfered to an oil bath and heated under reflux under nitrogen flow to remove 600 mL of the solvent. The reaction was cooled below 0 °C and a solution of $K_3Fe(CN)_6$ (244 g, 0.74 mol) in 800 mL of water was then added by addition funnel over 2 h, with the temperature maintained below 5 °C. A thick yellow precipitate formed. Water and ether were then added until two clear phases resulted. The organic phase was separated, washed with dilute brine, dried over anhydrous magnesium sulfate, filtered and stripped to give 108 g of crude product **1m**, which was flash vacuum distilled (85–105 °C/0.033 – 0.047 mmHg) to give 90.6 g (overall 75%) of pure **1m**. The product was analyzed by TLC, GC/MS, and NMR as pure. Bis(2,4,6-trifluorophenyl) disulfide (**1m**) [S10]: ^{1}H NMR ($CDCl_3$) δ 6.67–6.76 (m); ^{19}F NMR ($CDCl_3$) δ −100.32 (s, 4F), −102.31 (s, 2F); GC-Mass *m/z* 326 (M^+).

In the same way as for **1m**, bis(2,6-difluorophenyl) disulfide (**1k**), bis(2,3,6-trifluorophenyl) disulfide (**1l**), and bis(2,3,4,6-tetrafluorophenyl) disulfide (**1n**) were prepared from 1,3-difluorobenzene, 1,2,4-trifluorobenzene, and 1,2,3,5-tetrafluorobenzene, in overall 86%, 66%, and 82% yields, respectively.

Bis(2,6-difluorophenyl) disulfide (**1k**) [S11]: ^{1}H NMR ($CDCl_3$) δ 7.35 (m, 2H), 6.93 (m, 4H); ^{19}F NMR ($CDCl_3$) δ −103.84 (s); GC-Mass *m/z* 290 (M^+).

Bis(2,3,6-trifluorophenyl) disulfide (**1l**): ^{1}H NMR ($CDCl_3$) δ 7.2 (m, 2H), 6.9 (m, 2H); ^{19}F NMR ($CDCl_3$) δ −109.21 (m, 2F), −126 (dd, 2H), −140 (m, 2H); GC-Mass *m/z* 326 (M^+).

Bis(2,3,4,6-tetrafluorophenyl) disulfide (**1n**): ^{1}H NMR ($CDCl_3$) δ 6.86 (m); ^{19}F NMR ($CDCl_3$) δ −107.1 (m, 1F), −123.4 (m, 1F), −125.9 (m, 1F), −161.8 (m, 1F); GC-Mass *m/z* 362 (M^+).

10-3. Preparation of aryl dithols 1r–t

10-3-1. Preparation of 5-bromobenzene-1,3-dithiol (1r)

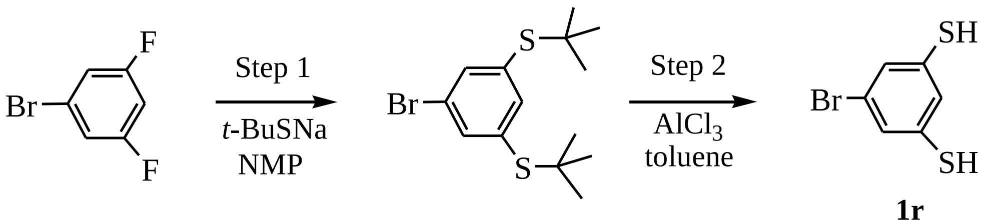

1r

Step 1: A 1 liter flask was charged with 1-bromo-3,5-difluorobenzene (67.3 g, 0.349 mol) and dry *N*-methylpyrrolidinone (300 mL), and sodium *tert*-butylthiolate (90.5 g, 0.81 mol) was added in portions. The reaction was stirred in the ice bath and warmed to room temperture overnight. The mixture was quenched with dilute aq. HCl, aq. NaCl, hexane, and ether. The phases were separated, and the aqueous layer was extracted again. The combined organic layer was washed once with aq. $NaHCO_3$,

dried over Na_2SO_4, and filtered. Evaporation of solvent from the filtrate gave the crude solid product, 116.8 g. Crystallization from hexane (200 mL) gave 98.6 g (85%) of 1,3-bis*(tert*-butylthio)-5-bromobenzene as white crystals. GC showed 99+% purity. ^{1}H NMR ($CDCl_3$) δ 7.64 (m, 1H), 7.68 (m, 2H), 1.29 (s, 18H); GC-Mass *m*/*z* 334, 332 (M^+).

Note: When the reaction was conducted using 1,3,5-tribromobenzene instead of 5-bromo-1,3-difluorobenzene in a way that sodium *tert*-butylthiolate was added stepwise, the product 1,3-bis(*tert*-butylthio)-5-bromobenzene was obtained in 72% yield.

Step 2: A 1 liter flask was charged with 1,3-bis(*tert*-butylthio)-5-bromobenzene (98.5 g, 0.295 mol) and dry deoxygenated toluene (700 mL). $AlCl_3$ (7.02 g, 0.0526 mol) was then quickly added into the mixture keeping a nitrogen blanket. After stirring at room temperature for 65 h, the reaction was quenched and agitated with 1N aq. HCl, aq. NaCl and pentane. The organic layer was separated, washed once with aq. NaCl, dried over $MgSO_4$, and filtered. Evaporation of the solvent from the filtrate under reduced pressure by heating gave a residue, which solidified into a white solid mass (66.9 g) on cooling. The solid was triturated in pentane, filtered, and dried in vacuum to give 57.8 g (88%) of 5-bromobenzene-1,3-dithiol (**1r**) [S12]. GC showed 99+% purity. ^{1}H NMR ($CDCl_3$) δ 7.08 (m, 1H), 7.19 (m, 2H), 3.46 (s, 2H); GC-Mass *m*/*z* 222, 220 (M^+).

10-3-2. Preparation of 2,5-difluorobenzene-1,4-dithiol (1s)

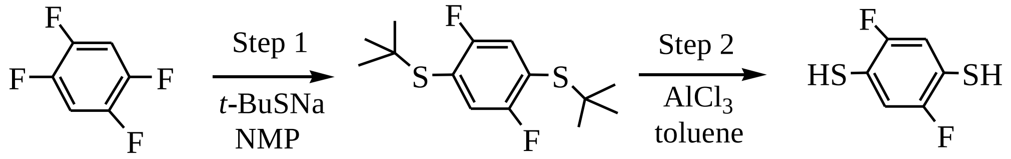

1s

Step 1: A 100 mL flask was charged with sodium *tert*-butylthiolate (35.9 g., 0.32 mol) and dry *N*-methylpyrrolidinone (160 mL), and the mixture was stirred under nitrogen for a few minutes. 1,2,4,5-Tetrafluorobenzene (23.6 g., 0.16 mol) was then introduced in one portion, slowly, by syringe. After a few minutes a strong exotherm was noted, and the mixture was allowed to come to room temperature under stirring for 1 day. The reaction was quenched and agitated with dilute aq. HCl, aq. NaCl, and hexane plus a small amount of diethyl ether. The organic phase was separated, dried over Na_2SO_4, filtered and evaporated to give the product as a fine white powder, 40.5 g (87% yield), which was analyzed by GC as 98+% purity. 1,4-Bis(*tert*-butylthio)-2,5-difluorobenzene [S13]: ^{1}H NMR ($CDCl_3$) δ 7.30 (dt, 2H), 1.33 (s, 18H); ^{19}F NMR ($CDCl_3$) δ −110.42 (t); ^{13}C NMR ($CDCl_3$) δ 160.8 (d), 157.5 (d), 125.7 (dt), 123.0 (dd), 48.2 (s), 31.1 (s); GC-Mass *m*/*z* 290 (M^+).

Step 2: 1,4-Bis(*tert*-butylthio)-2,5-difluorobenzene (39.7 g, 0.137 mol) was placed in a flask with 150 mL dry deoxygenated toluene and $AlCl_3$ (4.1 g, 0.031 mol). The reaction mixture was stirred for 2 days. The reaction was quenched and agitated with dilute aq.HCl, aq. NaCl, and ether. The organic layer was separated, washed with aq, NaCl, dried over anhydrous magnesium sulfate, and filtered. The filtrate was concentrated and distilled. In order to remove *tert*-butyltoluenes, the distillate was chromatographed with a short column of silica gel with pentane and then dichloromethane as eluents to give 14.6 g (60%) of 2,5-difluorobenzene-1,4-dithiol (**1s**). An analytical sample was obtained by recrystallization from pentane/dichloromethane; mp 59.9–61.1 °C; ^{1}H NMR ($CDCl_3$) δ 7.01 (t, J = 7.4 Hz, 2H), 3.61 (s, 2H); ^{19}F NMR ($CDCl_3$) δ −115.40 (t, J = 7.4 Hz); ^{13}C NMR ($CDCl_3$) δ 154.9 (dd, J = 242.8 Hz, 3.6 Hz), 116.5–118.2 (m); IR (nujol) 2572, 2530, 1281, 1181, 873, 777, 559 cm^{-1}; GC-Mass *m*/*z* 178 (M^+). Anal. Calcd for $C_6H_4F_2S_2$; C, 40.43%, H, 2.26%. Found, C, 40.55%, H, 2.26%

10-3-3. Preparation of 1,2,4,5-tetrafluorobenzene-1,4-dithiol (1t)

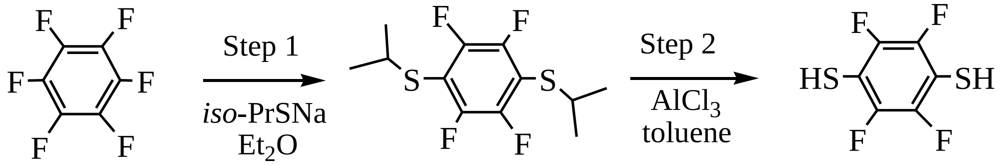

1t

Step 1: A 250 mL flask was charged with hexafluorobenzene (9.38 g, 0.05 mol) and diethyl ether (120 mL). Sodium isopropylthiolate (9.95 g, 0.10 mol) was added and the white slurry was stirred at room temperature for 2 days. A small amount of pentane was added and the reaction quenched with dilute aq. HCl to a pH of 5–6. The separated aqueous phase was extracted twice with diethyl ether, and the combined ether phases washed once with aq. sodium bicarbonate, dried over Na_2SO_4, filtered and evaporated to give a residue (12.7 g), which was dissolved in 20 mL pentane, and the solution was placed in a freezer overnight. The thick crop of resulting crystals was separated by efficient decantation, and then dried at room temperature in vacuum to give 10.7 g (71% yield) of 1,4-bis(isopropylthio)-2,3,5,6-tetrafluorobenzene [S14] as large needles. ^{1}H NMR ($CDCl_3$) δ 3.54 (septet, 2H), 1.28 (d, 12H); ^{19}F NMR ($CDCl_3$) δ −132.84 (s); ^{13}C NMR ($CDCl_3$) δ 148.9 (m), 145.5 (m), 114.3 (m), 39.1 (s), 23.3 (s); GC-MS *m*/*z* 298 (M^+).

Step 2: A 1 liter flask was charged with 1,4-bis(isopropylthio)-2,3,5,6-tetrafluorobenzene (55.9 g., 0.187 mol) and flushed with nitrogen. Dry, deoxygenated toluene (375 mL) was then added, maintaining nitrogen atmosphere. $AlCl_3$ (5.0 g, 0.037 mol) was added into the reaction mixture under nitrogen. An immediate red color developed along with a small white crystalline precipitate. The reaction was stirred at room temperature for 3 days. The reaction was quenched with dilute aq. HCl, aq.

NaCl, and ether, and the phases separated. The aqueous layer was extracted once with ether, and the combined organic phases washed once with dilute aq. NaCl, dried over Na_2SO_4, and filtered. After the removal of most volatiles on a rotovap, the residue solution was subjected to atmospheric distillation, raising the pot to a maximum of 230 °C until no more distillate came over. Upon cooling, crystals of the product appeared from the residue. The crystals were collected by suction filtration, washing through with a little pentane. An additional product was obtained by placing the filtrate in a freezer (−10 °C) overnight. The collected crystalline product was dried at 50 °C in vacuum (~1 mm Hg) for several hours, giving 29.9 g (74% yield) of 2,3,5,6-tetrafluorobenzene-1,4-dithiol (**1t**) [S15] as an off-white to light yellow crystalline solid: ^1H NMR ($CDCl_3$) δ 3.67 (s); ^{19}F NMR ($CDCl_3$) δ −136.83 (s); ^{13}C NMR ($CDCl_3$) δ 145.1 (m), 141.9 (m), 108.3 (m); GC-MS *m*/*z* 214 (M^+).

10-4. Preparation of aryl trithiol 1u

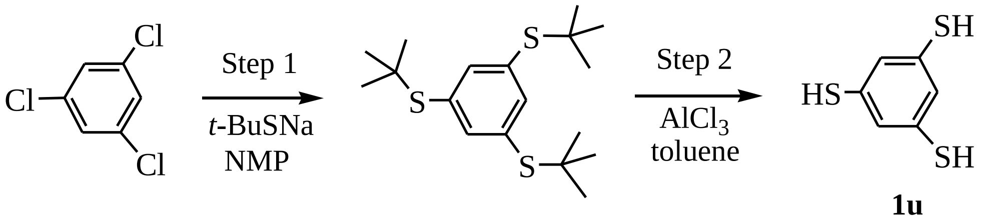

1u

Step 1: A 1 liter flask was charged with sodium *tert*-butylthiolate (81 g, 0.72 mol) and dry *N*-methylpyrrolidinone (200 mL). While the mixture was stirred, 1,3,5-trichlorobenzene (36.3 g, 0.20 mol) was added. The reaction mixture was then heated to 120 °C and stirred for 17 h at that temperature. The reaction was cooled and the solvent was removed by evaporation under reduced pressure on heating. The residue was dissolved in hexane and the hexane solution was washed with water and dried over magnesium sulfate, and filtered. Evaporation of the solvent from the filtrate gave a residue, which was then recrystallized from hexane to give 43.9 g (64%) of 1,3,5-tris(*tert*-butylthio)benzene; ^1H NMR ($CDCl_3$) δ 7.74 (s, 3H), 1.29 (s, 27H); ^{13}C NMR ($CDCl_3$) δ 146.3, 133.1, 46.6, 31.1; GC-MS *m*/*z* 342 (M^+).

Step 2: A 500 mL flask was charged with 1,3,5-tris(*tert*-butylthio)benzene (10.9 g, 31.8 mmol) and dry, deoxygenated toluene (250 mL), and finally $AlCl_3$ (2.81 g, 21 mmol). The reaction mixture was stirred at room temperature for 1 day. The reaction was quenched with dilute aq. HCl and ether, and the phases were seperated. The aqueous layer was extracted with pentane/ether, and the combined organic layer was washed with aq. NaCl, dried over magnesium sulfate, and filtered. Removal of the solvent including byproducts (*tert*-butylated toluenes) under reduced pressure on heating gave a residue, which solidified into a yellow crystal mass on cooling. The mass was then triturated in pentane and filtered to give 4.67 g (84%) of benzene-1,3,5-tri(thiol) (**1u**) as fine light yellow needles; mp 60.9–61.8 °C (lit. [S16] 59–60 °C). ^1H NMR ($CDCl_3$) δ 6.94 (s, 3H), 3.41 (s, 3H); ^{13}C NMR ($CDCl_3$) δ 133.2, 126.3.

11. References and Notes

S1. Robson, P.; Stacey, M.; Stephens, R.; Tatlow, J. C. *J. Chem. Soc.* **1960**, 4754-4760.

S2. Maiolo, F.; Testaferri, L.; Tiecco, M.; Tingoli, M. *J. Org. Chem.* **1981**, *46*, 3070-3073.

S3. Umemoto, T. *PCT Int. Appl. WO* 2008/118787 A1; *U.S. Pat. US* 7,592,491; *U.S. Pat. US* 7,820,864; *U.S. Pat. US* 7,851,646.

S4. Umemoto, T. *U.S. Pat. Appl. Publ. US* 2010/0130790 A1.

S5. Umemoto, T. *PCT Int. Appl. WO* 2010/033930 A1.

S6. Wessel, J.; Hartl, H.; Seppelt, K. *Chem. Ber.* **1986**, *119*, 453-463.

S7. Umemoto, T.; Singh, R. P.; Xu, Y.; Saito, N. *J. Am. Chem. Soc.* **2010**, *132*, 18199-18205 and Supporting Information.

S8. Yates, J.; Haddock, E. *GB Patent No.* 1128217 (1968).

S9. Hynd, G.; Ray, N. C.; Finch, H.; Middlemiss, D.; Cramp, M. C.; Blaney, P. M.; Williams, K.; Griffon, Y.; Harrison, T. K.; Crackett, P. *US Patent Application No.* 2010/0010034.

S10. Sakagami, M.; Mano, S. *Jpn Kokai Tokkyo Koho JP* 2002-338748.

S11. Nishino, S.; Yoneda, Y.; Oda, H. *Jpn Kokai Tokkyo Koho JP* 2011-153094.

S12. Commercially available from TCI America (B2655).

S13. Wang, Y.; Parkin, S. R.; Gierschner, J.; Watson, M. D. *Org. Lett.* **2008**, *10*, 3307-3310.

S14. Crowell, T. R.; Peach, M. E. *J. Fluorine Chem.* **1982**, *21*, 469-477.

S15. Mobbs, R. H. *Chem. & Ind. (London)* **1965**, *36*, 1562-1563.

S16. Dictionary of Organic Compounds Sixth Ed. Vol. 1, p. 585, Chapman & Hall (London): Commercially available from TCI America (B2656).

12. ^1H, ^{19}F, and ^{13}C-NMR spectra of new products

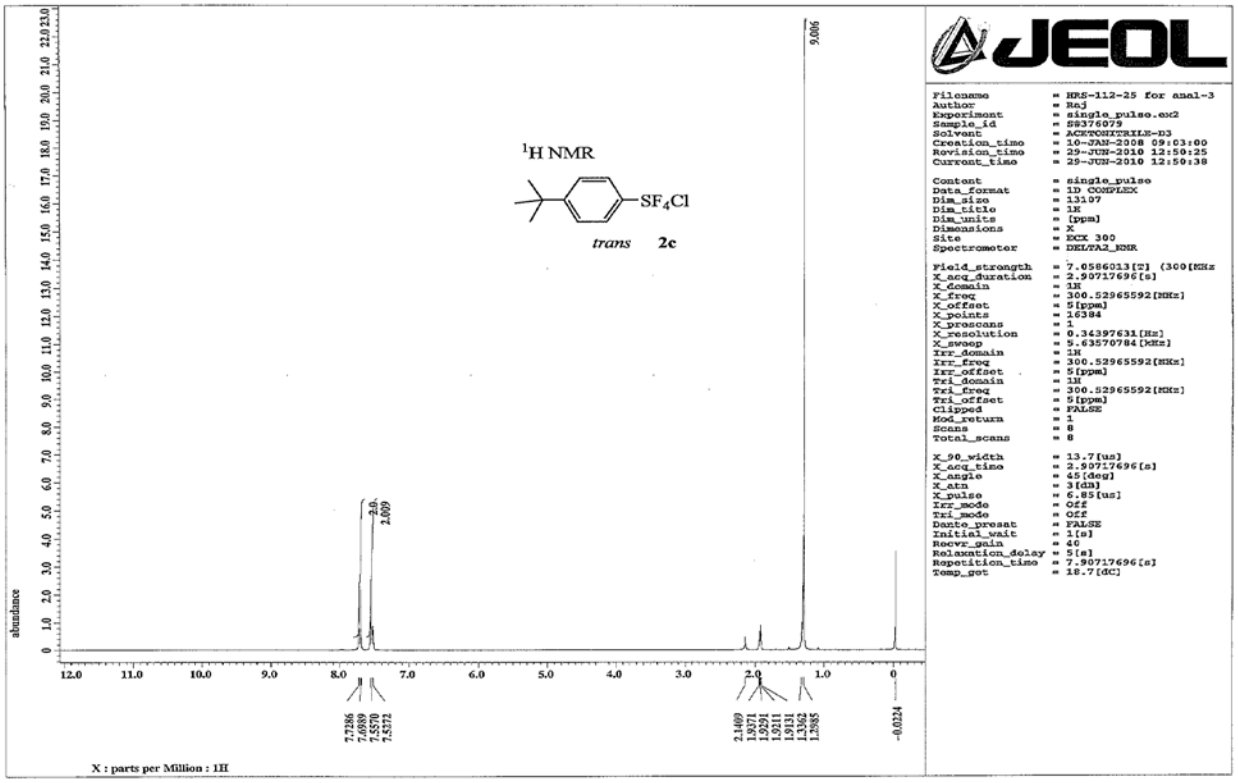

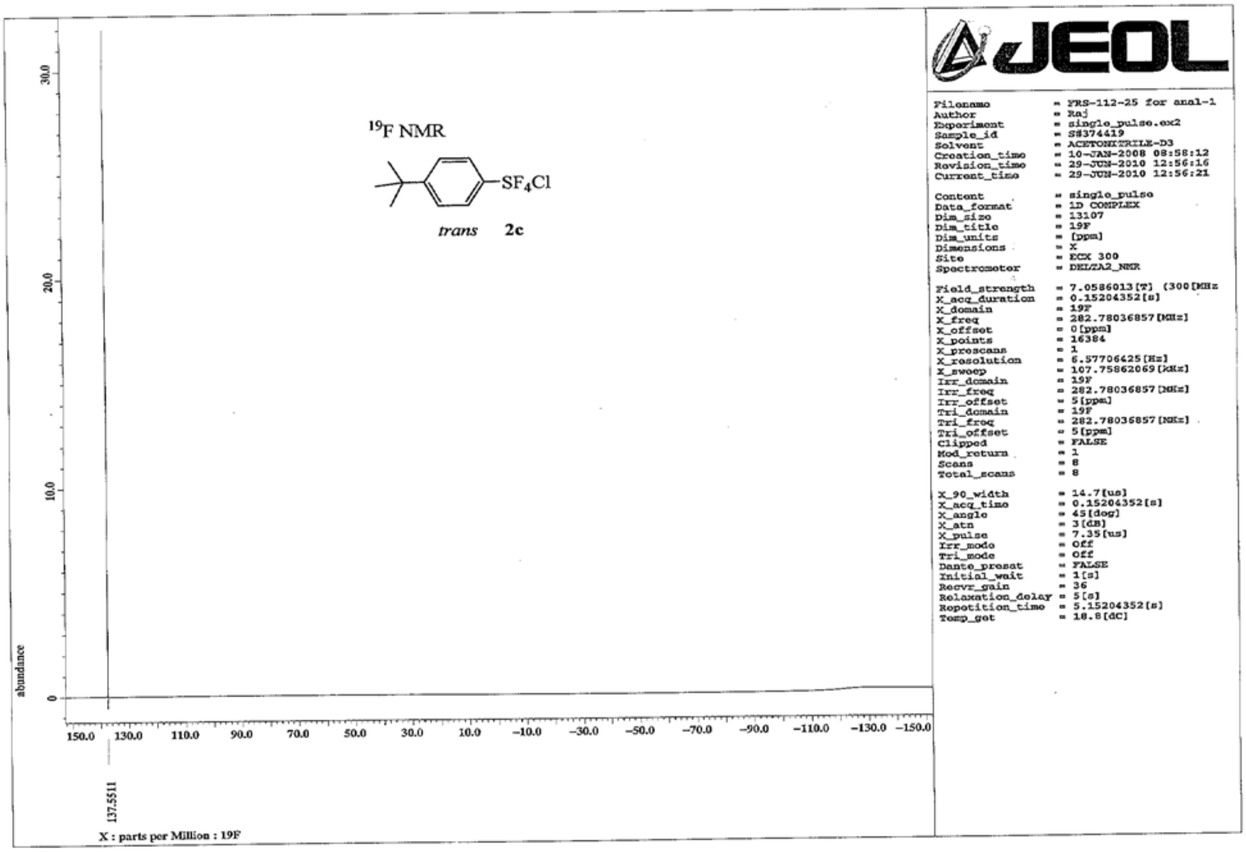

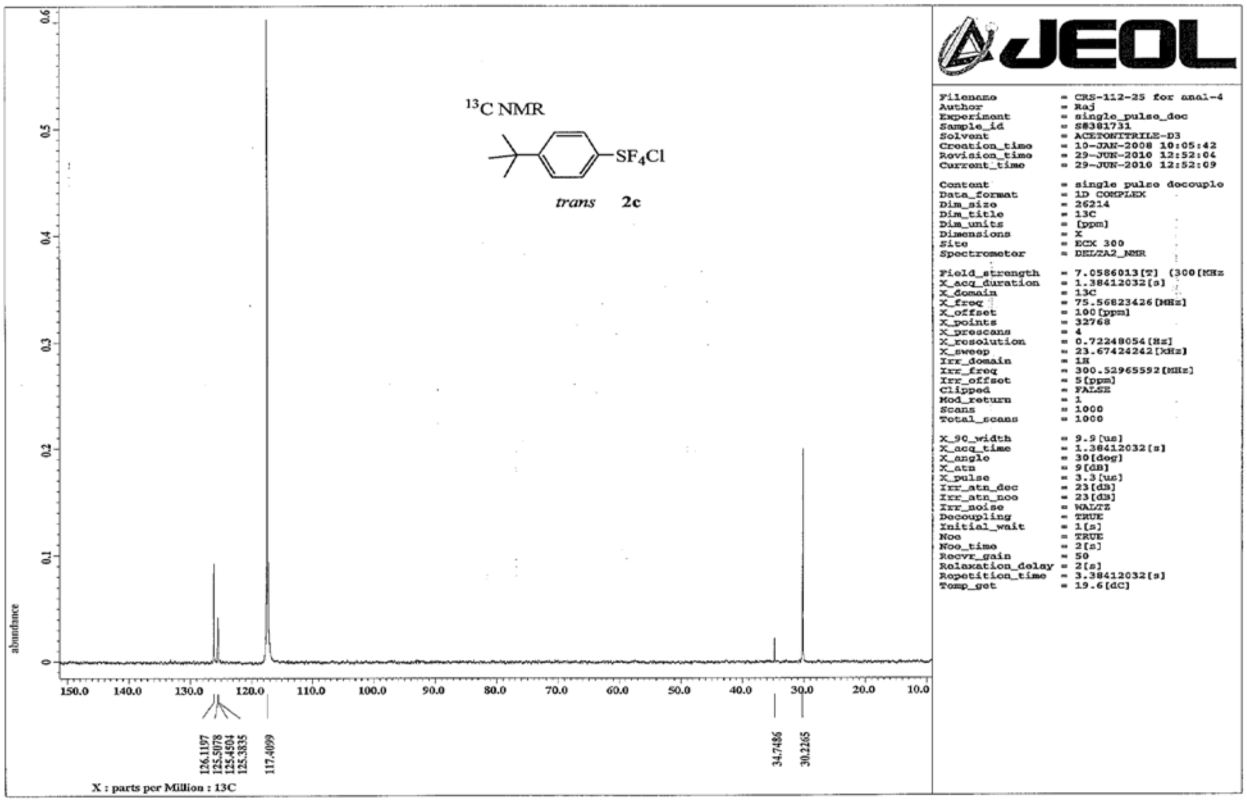

13C NMR
SF4Cl
trans 2c
abundance
126.1197
125.5078
125.4504
125.3835
117.4099
34.7486
30.2265
X : parts per Million : 13C
JEOL

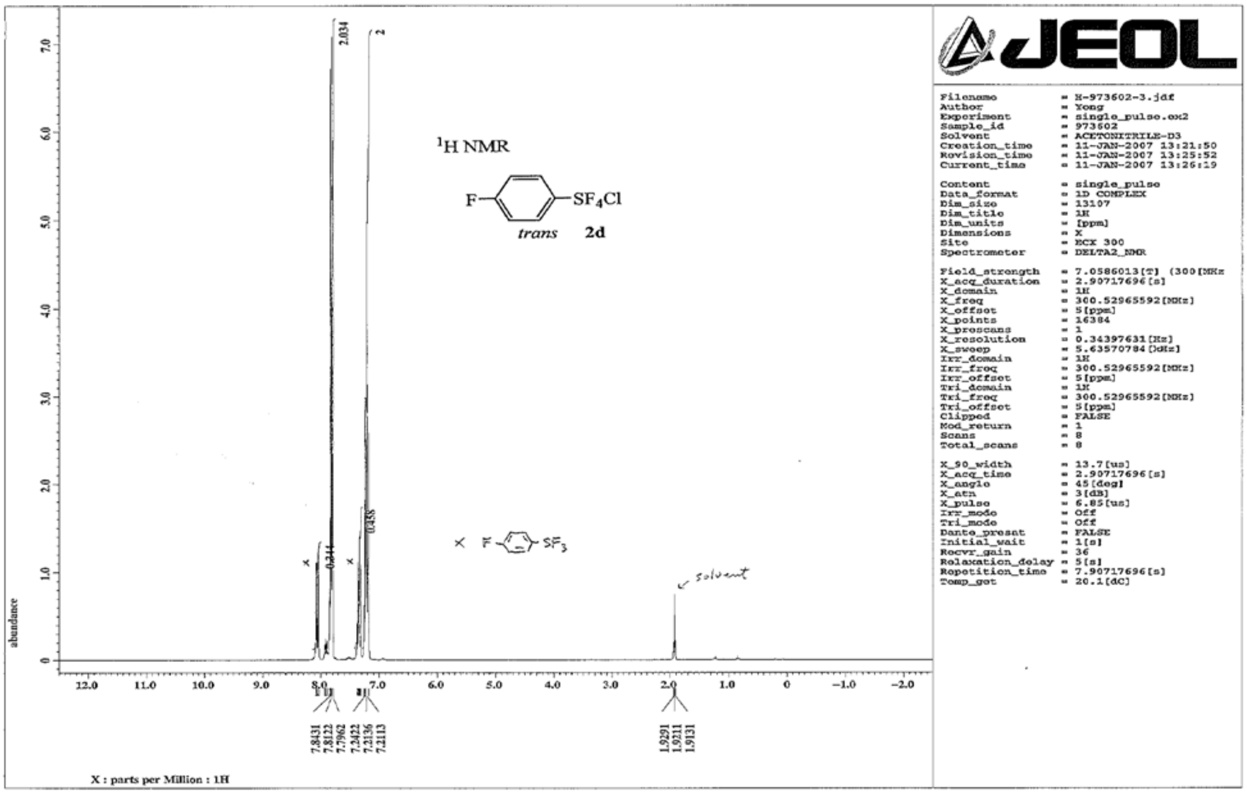

1H NMR
F
SF4Cl
trans 2d
abundance
2.034
2
0.311
0.458
F SF3
solvent
7.8431
7.8122
7.7962
7.2422
7.2136
7.2113
1.9291
1.9211
1.9131
X : parts per Million : 1H
JEOL

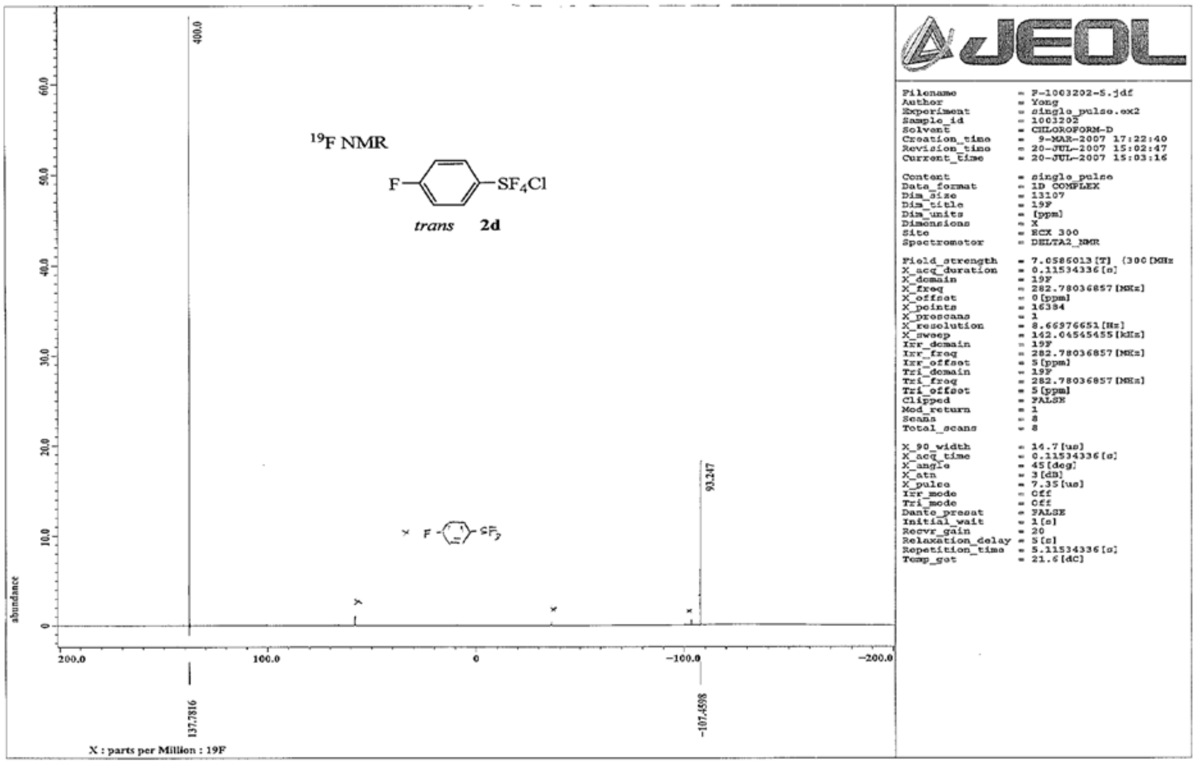
19F NMR
F–C6H4–SF4Cl
trans 2d
400.0
93.247
137.7816
−107.4598
abundance
X : parts per Million : 19F
JEOL

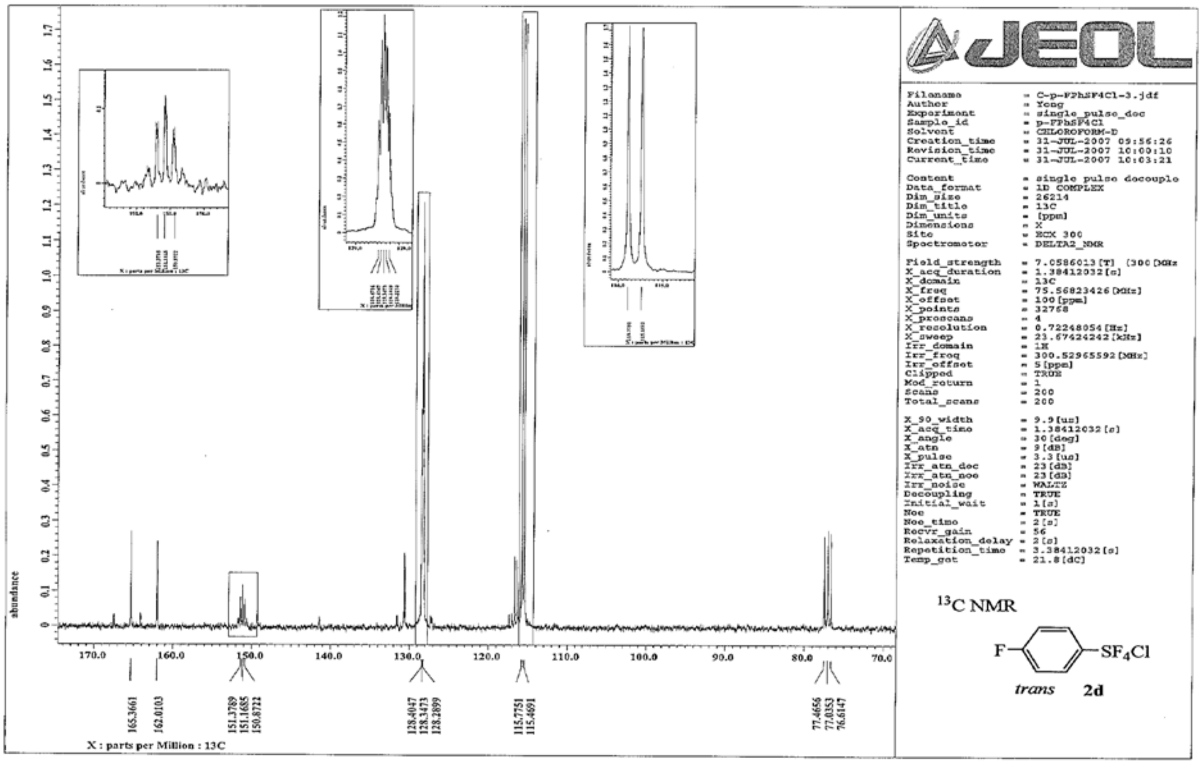
13C NMR
F–C6H4–SF4Cl
trans 2d
165.3661
162.0103
151.3789
151.1685
150.8722
128.4047
128.3473
128.2899
115.7751
115.4691
77.4656
77.0353
76.6147
abundance
X : parts per Million : 13C
JEOL

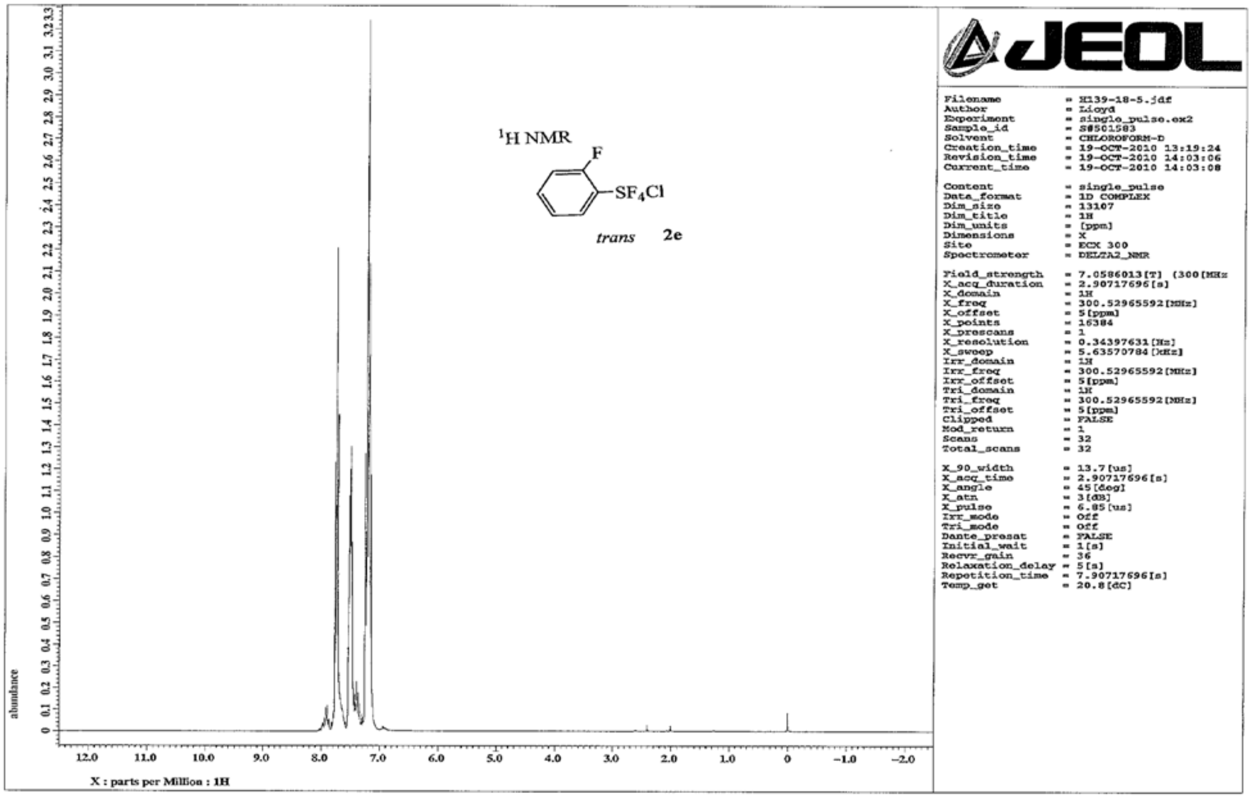

1H NMR
F
SF4Cl
trans 2e
abundance
X : parts per Million : 1H
JEOL

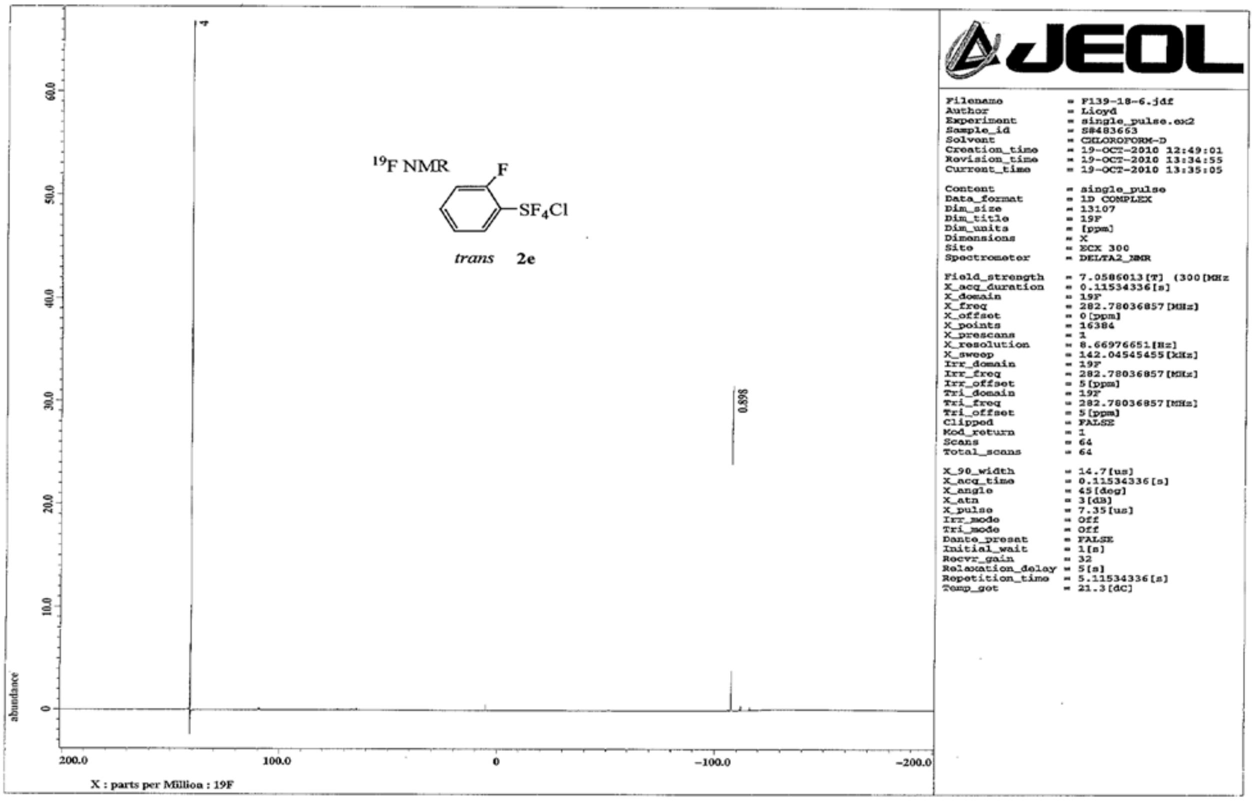

19F NMR
F
SF4Cl
trans 2e
0.898
abundance
X : parts per Million : 19F
JEOL

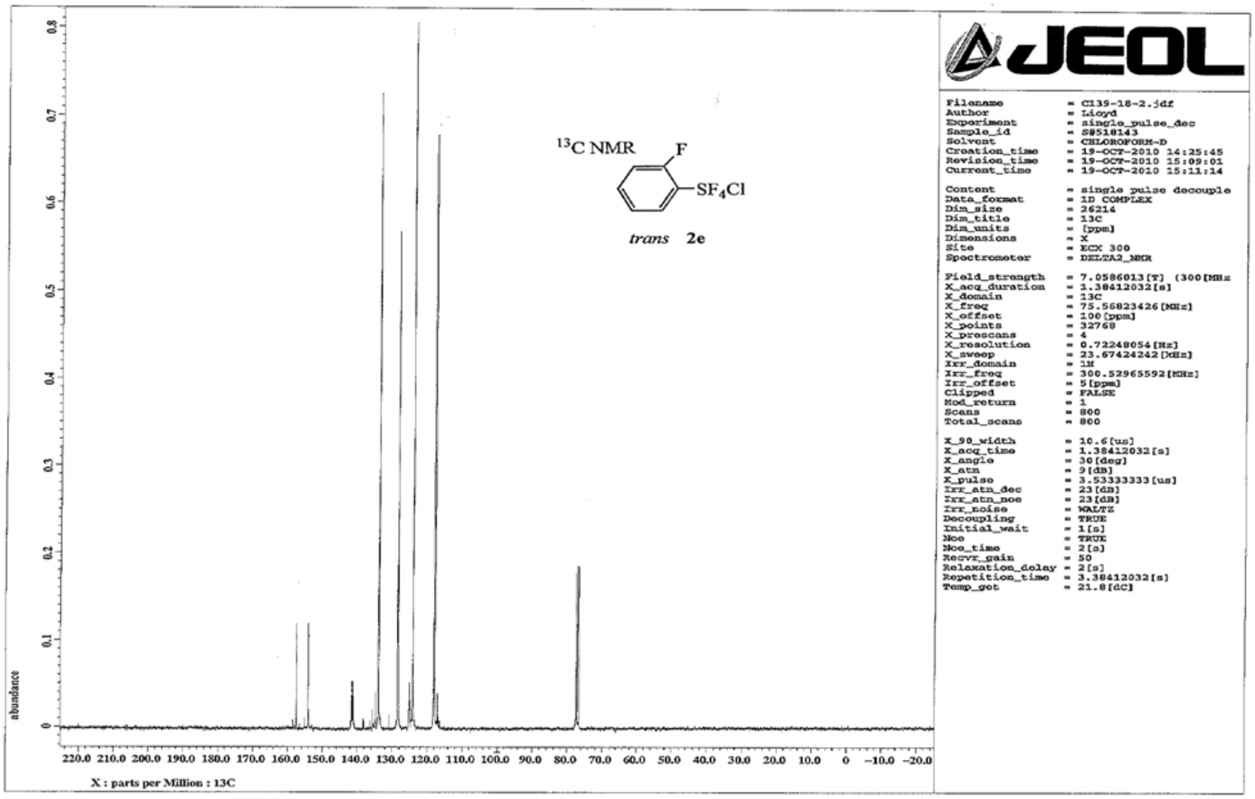
13C NMR
F
SF4Cl
trans 2e
JEOL
Filename = C139-18-2.jdf
Author = Lloyd
Experiment = single_pulse_dec
Sample_id = S8518143
Solvent = CHLOROFORM-D
Creation_time = 19-OCT-2010 14:25:45
Revision_time = 19-OCT-2010 15:09:01
Current_time = 19-OCT-2010 15:11:14
Content = single pulse decouple
Data_format = 1D COMPLEX
Dim_size = 26214
Dim_title = 13C
Dim_units = [ppm]
Dimensions = X
Site = ECX 300
Spectrometer = DELTA2_NMR
Field_strength = 7.0586013[T] (300[MHz
X_acq_duration = 1.38412032[s]
X_domain = 13C
X_freq = 75.56823426[MHz]
X_offset = 100[ppm]
X_points = 32768
X_prescans = 4
X_resolution = 0.72248054[Hz]
X_sweep = 23.67424242[kHz]
Irr_domain = 1H
Irr_freq = 300.52965592[MHz]
Irr_offset = 5[ppm]
Clipped = FALSE
Mod_return = 1
Scans = 800
Total_scans = 800
X_90_width = 10.6[us]
X_acq_time = 1.38412032[s]
X_angle = 30[deg]
X_atn = 9[dB]
X_pulse = 3.53333333[us]
Irr_atn_dec = 23[dB]
Irr_atn_noe = 23[dB]
Irr_noise = WALTZ
Decoupling = TRUE
Initial_wait = 1[s]
Noe = TRUE
Noe_time = 2[s]
Recvr_gain = 50
Relaxation_delay = 2[s]
Repetition_time = 3.38412032[s]
Temp_get = 21.8[dC]
abundance
X : parts per Million : 13C

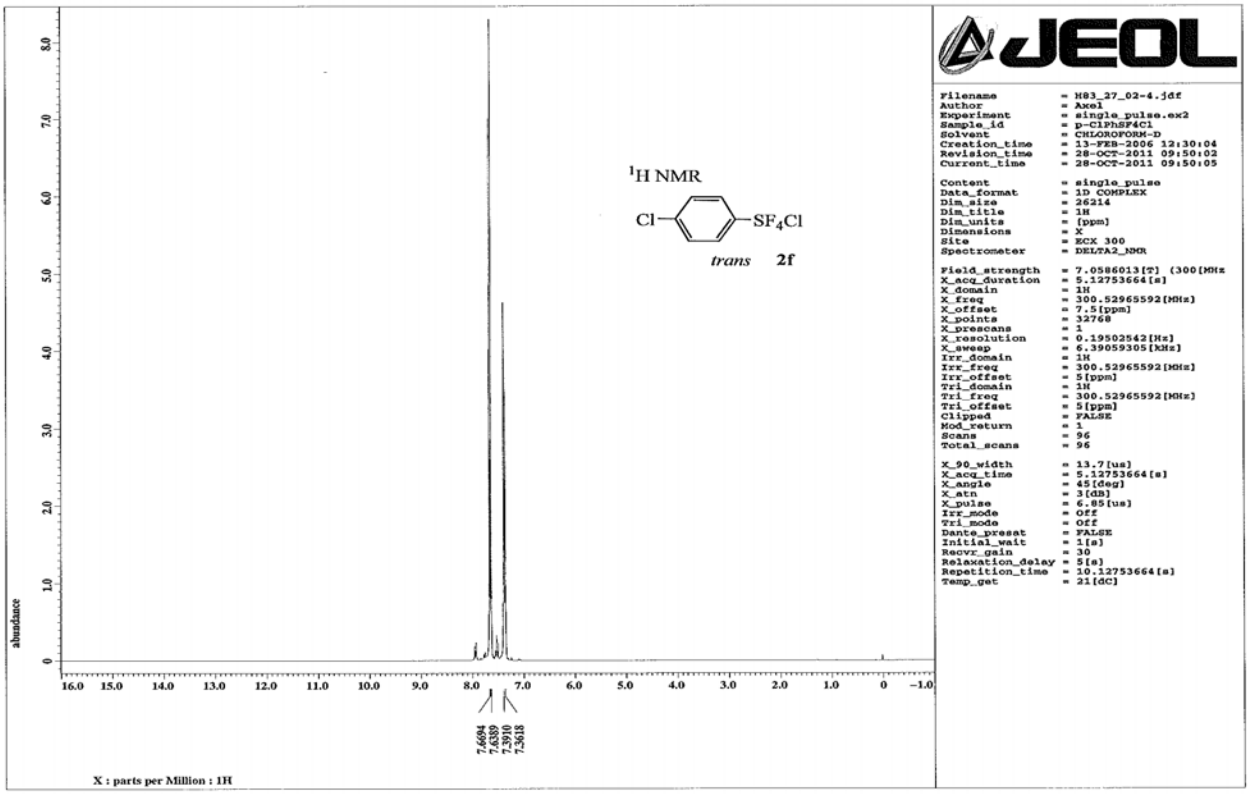
1H NMR
Cl
SF4Cl
trans 2f
JEOL
Filename = H83_27_02-4.jdf
Author = Axel
Experiment = single_pulse.ex2
Sample_id = p-ClPhSF4Cl
Solvent = CHLOROFORM-D
Creation_time = 13-FEB-2006 12:30:04
Revision_time = 28-OCT-2011 09:50:02
Current_time = 28-OCT-2011 09:50:05
Content = single_pulse
Data_format = 1D COMPLEX
Dim_size = 26214
Dim_title = 1H
Dim_units = [ppm]
Dimensions = X
Site = ECX 300
Spectrometer = DELTA2_NMR
Field_strength = 7.0586013[T] (300[MHz
X_acq_duration = 5.12753664[s]
X_domain = 1H
X_freq = 300.52965592[MHz]
X_offset = 7.5[ppm]
X_points = 32768
X_prescans = 1
X_resolution = 0.19502542[Hz]
X_sweep = 6.39059305[kHz]
Irr_domain = 1H
Irr_freq = 300.52965592[MHz]
Irr_offset = 5[ppm]
Tri_domain = 1H
Tri_freq = 300.52965592[MHz]
Tri_offset = 5[ppm]
Clipped = FALSE
Mod_return = 1
Scans = 96
Total_scans = 96
X_90_width = 13.7[us]
X_acq_time = 5.12753664[s]
X_angle = 45[deg]
X_atn = 3[dB]
X_pulse = 6.85[us]
Irr_mode = Off
Tri_mode = Off
Dante_presat = FALSE
Initial_wait = 1[s]
Recvr_gain = 30
Relaxation_delay = 5[s]
Repetition_time = 10.12753664[s]
Temp_get = 21[dC]
7.6694
7.6389
7.3910
7.3618
abundance
X : parts per Million : 1H

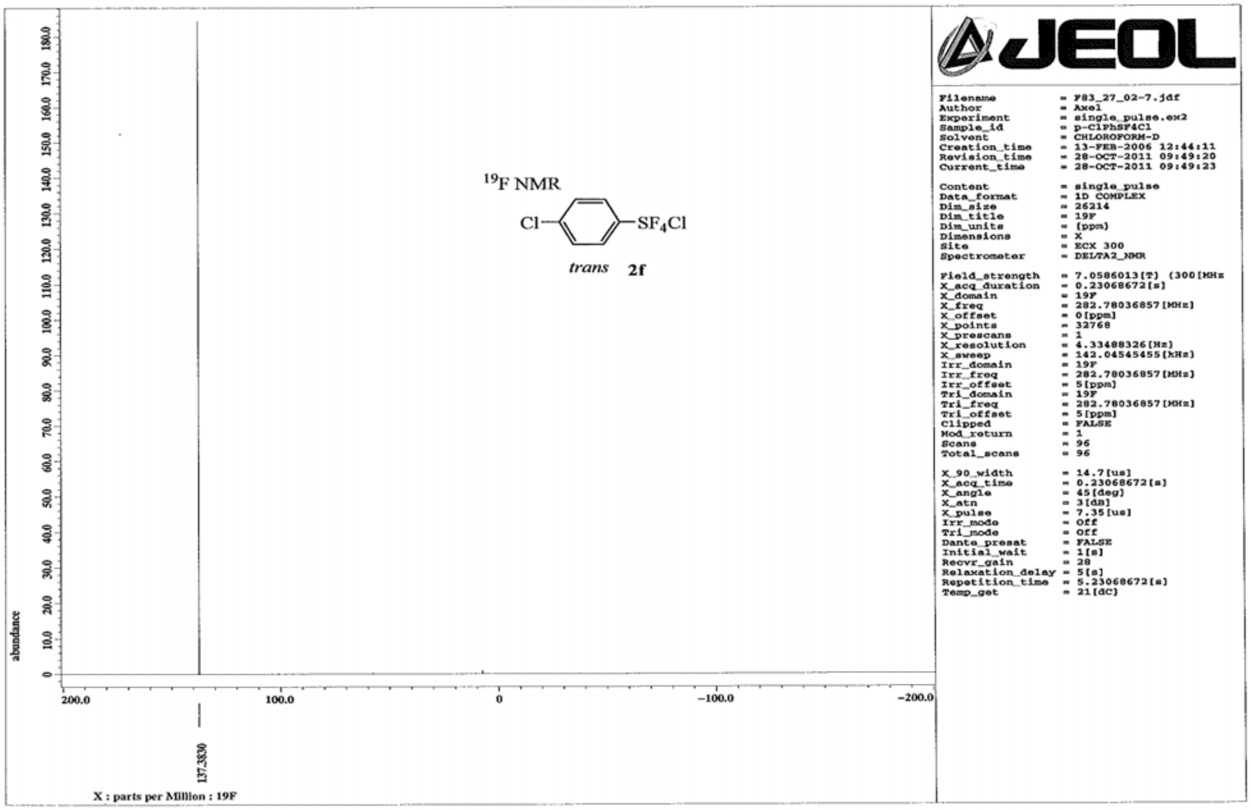

19F NMR
Cl
SF4Cl
trans 2f
Filename = F83_27_02-7.jdf
Author = Axel
Experiment = single_pulse.ex2
Sample_id = p-ClPhSF4Cl
Solvent = CHLOROFORM-D
Creation_time = 13-FEB-2006 12:44:11
Revision_time = 28-OCT-2011 09:49:20
Current_time = 28-OCT-2011 09:49:23
Content = single_pulse
Data_format = 1D COMPLEX
Dim_size = 26214
Dim_title = 19F
Dim_units = [ppm]
Dimensions = X
Site = ECX 300
Spectrometer = DELTA2_NMR
Field_strength = 7.0586013[T] (300[MHz
X_acq_duration = 0.23068672[s]
X_domain = 19F
X_freq = 282.78036857[MHz]
X_offset = 0[ppm]
X_points = 32768
X_prescans = 1
X_resolution = 4.33488326[Hz]
X_sweep = 142.04545455[kHz]
Irr_domain = 19F
Irr_freq = 282.78036857[MHz]
Irr_offset = 5[ppm]
Tri_domain = 19F
Tri_freq = 282.78036857[MHz]
Tri_offset = 5[ppm]
Clipped = FALSE
Mod_return = 1
Scans = 96
Total_scans = 96
X_90_width = 14.7[us]
X_acq_time = 0.23068672[s]
X_angle = 45[deg]
X_atn = 3[dB]
X_pulse = 7.35[us]
Irr_mode = Off
Tri_mode = Off
Dante_presat = FALSE
Initial_wait = 1[s]
Recvr_gain = 28
Relaxation_delay = 5[s]
Repetition_time = 5.23068672[s]
Temp_get = 21[dC]
abundance
137.3830
X : parts per Million : 19F

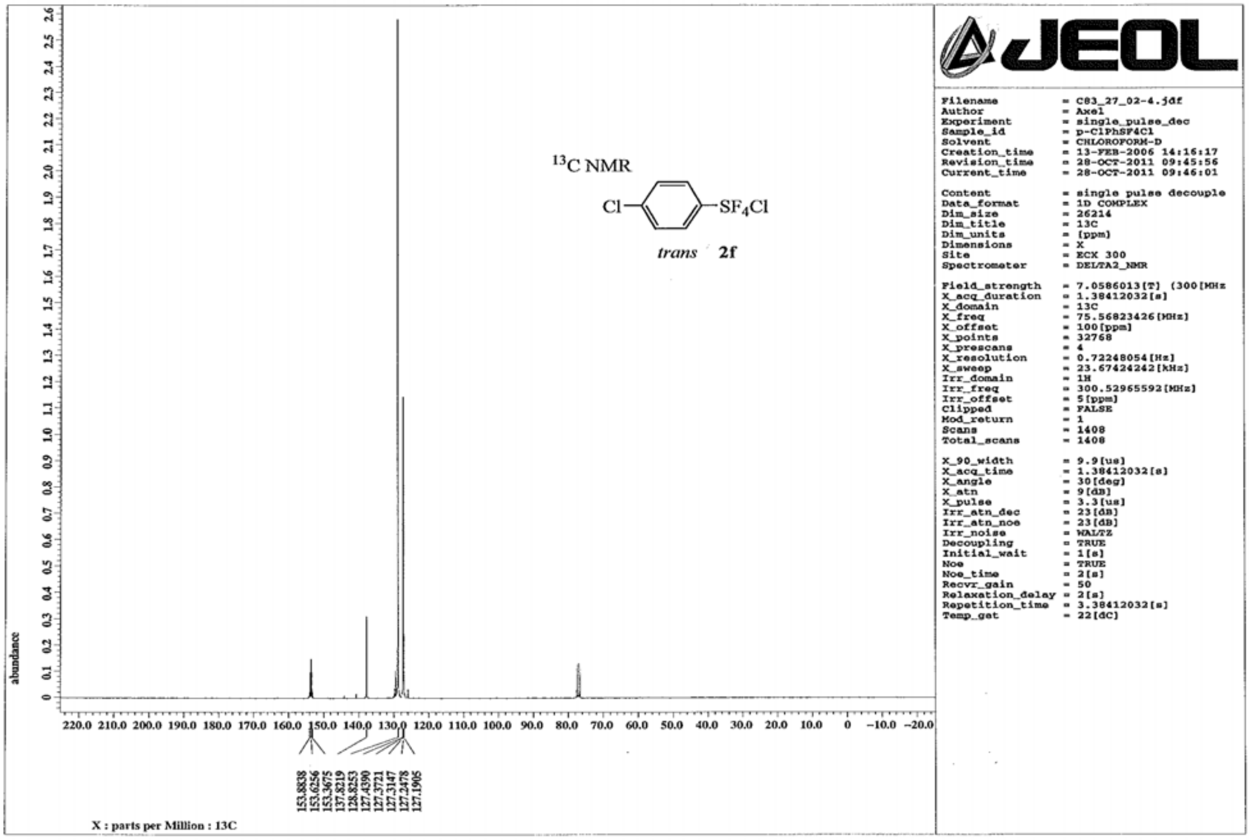

13C NMR
Cl
SF4Cl
trans 2f
Filename = C83_27_02-4.jdf
Author = Axel
Experiment = single_pulse_dec
Sample_id = p-ClPhSF4Cl
Solvent = CHLOROFORM-D
Creation_time = 13-FEB-2006 14:16:17
Revision_time = 28-OCT-2011 09:45:56
Current_time = 28-OCT-2011 09:46:01
Content = single pulse decouple
Data_format = 1D COMPLEX
Dim_size = 26214
Dim_title = 13C
Dim_units = [ppm]
Dimensions = X
Site = ECX 300
Spectrometer = DELTA2_NMR
Field_strength = 7.0586013[T] (300[MHz
X_acq_duration = 1.38412032[s]
X_domain = 13C
X_freq = 75.56823426[MHz]
X_offset = 100[ppm]
X_points = 32768
X_prescans = 4
X_resolution = 0.72248054[Hz]
X_sweep = 23.67424242[kHz]
Irr_domain = 1H
Irr_freq = 300.52965592[MHz]
Irr_offset = 5[ppm]
Clipped = FALSE
Mod_return = 1
Scans = 1408
Total_scans = 1408
X_90_width = 9.9[us]
X_acq_time = 1.38412032[s]
X_angle = 30[deg]
X_atn = 9[dB]
X_pulse = 3.3[us]
Irr_atn_dec = 23[dB]
Irr_atn_noe = 23[dB]
Irr_noise = WALTZ
Decoupling = TRUE
Initial_wait = 1[s]
Noe = TRUE
Noe_time = 2[s]
Recvr_gain = 50
Relaxation_delay = 2[s]
Repetition_time = 3.38412032[s]
Temp_get = 22[dC]
abundance
153.8838
153.6256
153.3675
137.8219
128.8253
127.4390
127.3721
127.3147
127.2478
127.1905
X : parts per Million : 13C

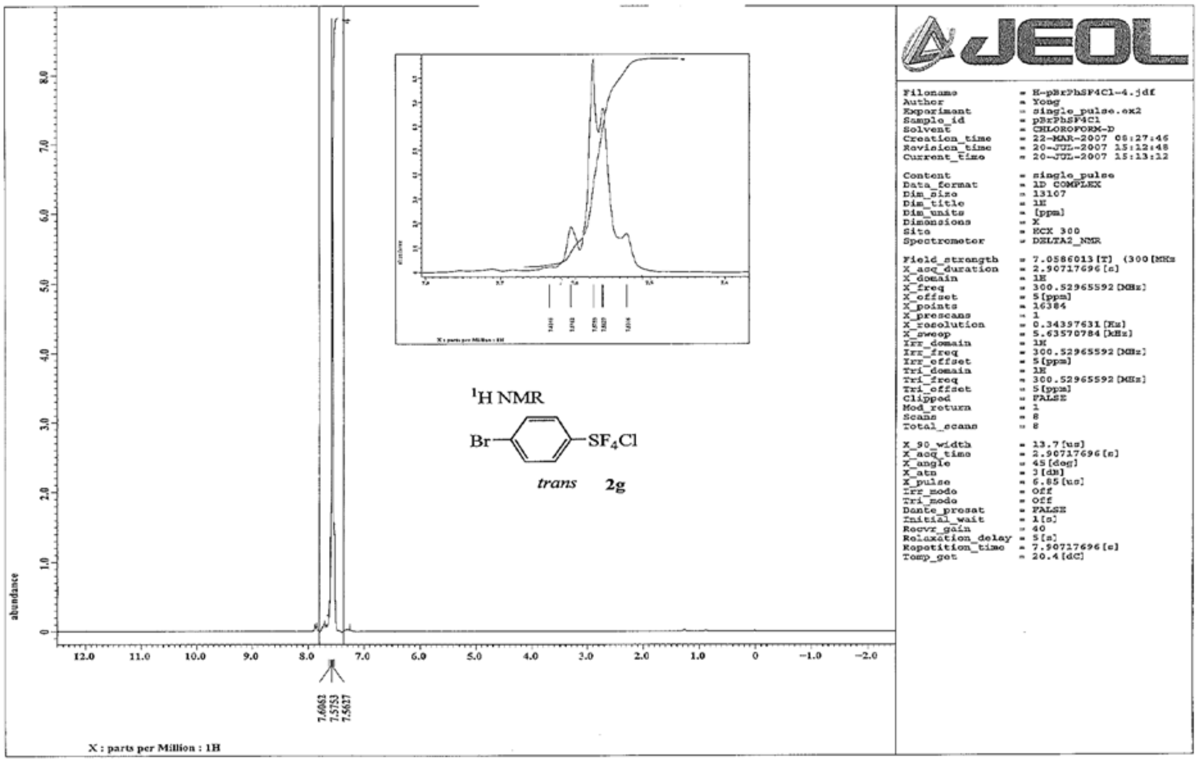

JEOL
Filename = H-pBrPhSF4Cl-4.jdf
Author = Yong
Experiment = single_pulse.ex2
Sample_id = pBrPhSF4Cl
Solvent = CHLOROFORM-D
Creation_time = 22-MAR-2007 08:27:46
Revision_time = 20-JUL-2007 15:12:48
Current_time = 20-JUL-2007 15:13:12
Content = single_pulse
Data_format = 1D COMPLEX
Dim_size = 13107
Dim_title = 1H
Dim_units = [ppm]
Dimensions = X
Site = ECX 300
Spectrometer = DELTA2_NMR
Field_strength = 7.0586013[T] (300[MHz
X_acq_duration = 2.90717696[s]
X_domain = 1H
X_freq = 300.52965592[MHz]
X_offset = 5[ppm]
X_points = 16384
X_prescans = 1
X_resolution = 0.34397631[Hz]
X_sweep = 5.63570784[kHz]
Irr_domain = 1H
Irr_freq = 300.52965592[MHz]
Irr_offset = 5[ppm]
Tri_domain = 1H
Tri_freq = 300.52965592[MHz]
Tri_offset = 5[ppm]
Clipped = FALSE
Mod_return = 1
Scans = 8
Total_scans = 8
X_90_width = 13.7[us]
X_acq_time = 2.90717696[s]
X_angle = 45[deg]
X_atn = 3[dB]
X_pulse = 6.85[us]
Irr_mode = Off
Tri_mode = Off
Dante_presat = FALSE
Initial_wait = 1[s]
Recvr_gain = 40
Relaxation_delay = 5[s]
Repetition_time = 7.90717696[s]
Temp_get = 20.4[dC]
¹H NMR
Br–C₆H₄–SF₄Cl
trans 2g
7.6063
7.5753
7.5627
abundance
X : parts per Million : 1H

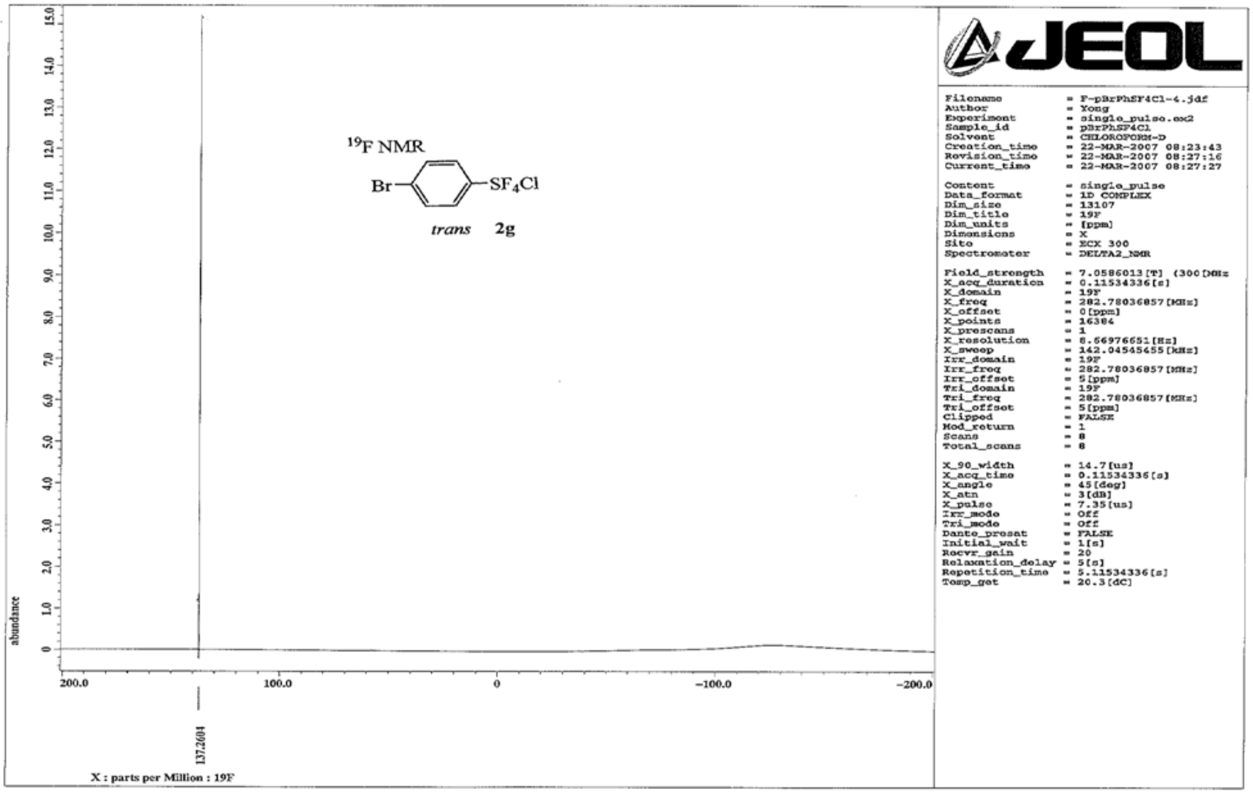

JEOL
Filename = F-pBrPhSF4Cl-4.jdf
Author = Yong
Experiment = single_pulse.ex2
Sample_id = pBrPhSF4Cl
Solvent = CHLOROFORM-D
Creation_time = 22-MAR-2007 08:23:43
Revision_time = 22-MAR-2007 08:27:16
Current_time = 22-MAR-2007 08:27:27
Content = single_pulse
Data_format = 1D COMPLEX
Dim_size = 13107
Dim_title = 19F
Dim_units = [ppm]
Dimensions = X
Site = ECX 300
Spectrometer = DELTA2_NMR
Field_strength = 7.0586013[T] (300[MHz
X_acq_duration = 0.11534336[s]
X_domain = 19F
X_freq = 282.78036857[MHz]
X_offset = 0[ppm]
X_points = 16384
X_prescans = 1
X_resolution = 8.66976651[Hz]
X_sweep = 142.04545455[kHz]
Irr_domain = 19F
Irr_freq = 282.78036857[MHz]
Irr_offset = 5[ppm]
Tri_domain = 19F
Tri_freq = 282.78036857[MHz]
Tri_offset = 5[ppm]
Clipped = FALSE
Mod_return = 1
Scans = 8
Total_scans = 8
X_90_width = 14.7[us]
X_acq_time = 0.11534336[s]
X_angle = 45[deg]
X_atn = 3[dB]
X_pulse = 7.35[us]
Irr_mode = Off
Tri_mode = Off
Dante_presat = FALSE
Initial_wait = 1[s]
Recvr_gain = 20
Relaxation_delay = 5[s]
Repetition_time = 5.11534336[s]
Temp_get = 20.3[dC]
¹⁹F NMR
Br–C₆H₄–SF₄Cl
trans 2g
137.2604
abundance
X : parts per Million : 19F

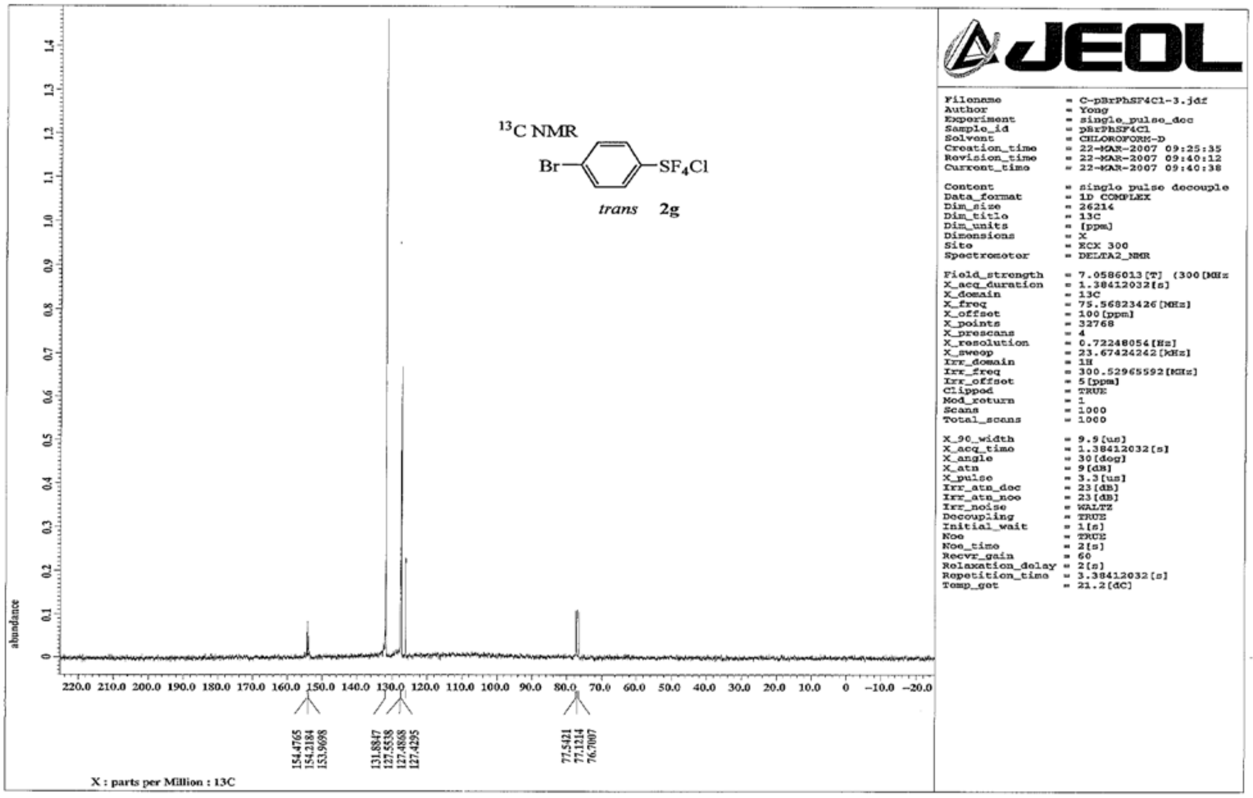
13C NMR
Br
SF4Cl
trans 2g
JEOL
Filename = C-pBrPhSF4Cl-3.jdf
Author = Yong
Experiment = single_pulse_dec
Sample_id = pBrPhSF4Cl
Solvent = CHLOROFORM-D
Creation_time = 22-MAR-2007 09:25:35
Revision_time = 22-MAR-2007 09:40:12
Current_time = 22-MAR-2007 09:40:38
Content = single pulse decouple
Data_format = 1D COMPLEX
Dim_size = 26214
Dim_title = 13C
Dim_units = [ppm]
Dimensions = X
Site = ECX 300
Spectrometer = DELTA2_NMR
Field_strength = 7.0586013[T] (300[MHz
X_acq_duration = 1.38412032[s]
X_domain = 13C
X_freq = 75.56823426[MHz]
X_offset = 100[ppm]
X_points = 32768
X_prescans = 4
X_resolution = 0.72248054[Hz]
X_sweep = 23.67424242[kHz]
Irr_domain = 1H
Irr_freq = 300.52965592[MHz]
Irr_offset = 5[ppm]
Clipped = TRUE
Mod_return = 1
Scans = 1000
Total_scans = 1000
X_90_width = 9.9[us]
X_acq_time = 1.38412032[s]
X_angle = 30[deg]
X_atn = 9[dB]
X_pulse = 3.3[us]
Irr_atn_dec = 23[dB]
Irr_atn_noe = 23[dB]
Irr_noise = WALTZ
Decoupling = TRUE
Initial_wait = 1[s]
Noe = TRUE
Noe_time = 2[s]
Recvr_gain = 60
Relaxation_delay = 2[s]
Repetition_time = 3.38412032[s]
Temp_get = 21.2[dC]
abundance
154.4765
154.2184
153.9698
131.8847
127.5538
127.4868
127.4295
77.5421
77.1214
76.7007
X : parts per Million : 13C

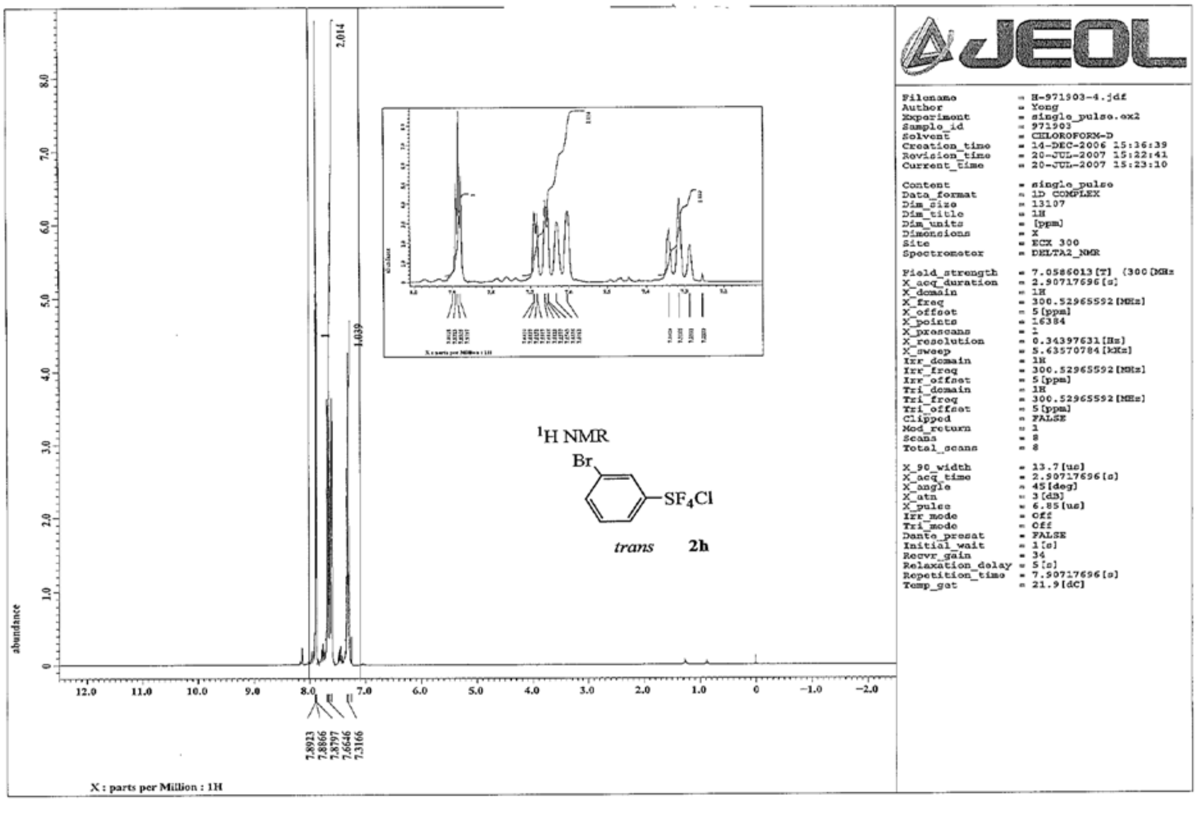
1H NMR
Br
SF4Cl
trans 2h
JEOL
Filename = H-971903-4.jdf
Author = Yong
Experiment = single_pulse.ex2
Sample_id = 971903
Solvent = CHLOROFORM-D
Creation_time = 14-DEC-2006 15:36:39
Revision_time = 20-JUL-2007 15:22:41
Current_time = 20-JUL-2007 15:23:10
Content = single_pulse
Data_format = 1D COMPLEX
Dim_size = 13107
Dim_title = 1H
Dim_units = [ppm]
Dimensions = X
Site = ECX 300
Spectrometer = DELTA2_NMR
Field_strength = 7.0586013[T] (300[MHz
X_acq_duration = 2.90717696[s]
X_domain = 1H
X_freq = 300.52965592[MHz]
X_offset = 5[ppm]
X_points = 16384
X_prescans = 1
X_resolution = 0.34397631[Hz]
X_sweep = 5.63570784[kHz]
Irr_domain = 1H
Irr_freq = 300.52965592[MHz]
Irr_offset = 5[ppm]
Tri_domain = 1H
Tri_freq = 300.52965592[MHz]
Tri_offset = 5[ppm]
Clipped = FALSE
Mod_return = 1
Scans = 8
Total_scans = 8
X_90_width = 13.7[us]
X_acq_time = 2.90717696[s]
X_angle = 45[deg]
X_atn = 3[dB]
X_pulse = 6.85[us]
Irr_mode = Off
Tri_mode = Off
Dante_presat = FALSE
Initial_wait = 1[s]
Recvr_gain = 34
Relaxation_delay = 5[s]
Repetition_time = 7.90717696[s]
Temp_get = 21.9[dC]
abundance
2.014
1.039
7.8923
7.8866
7.8797
7.6646
7.3166
X : parts per Million : 1H

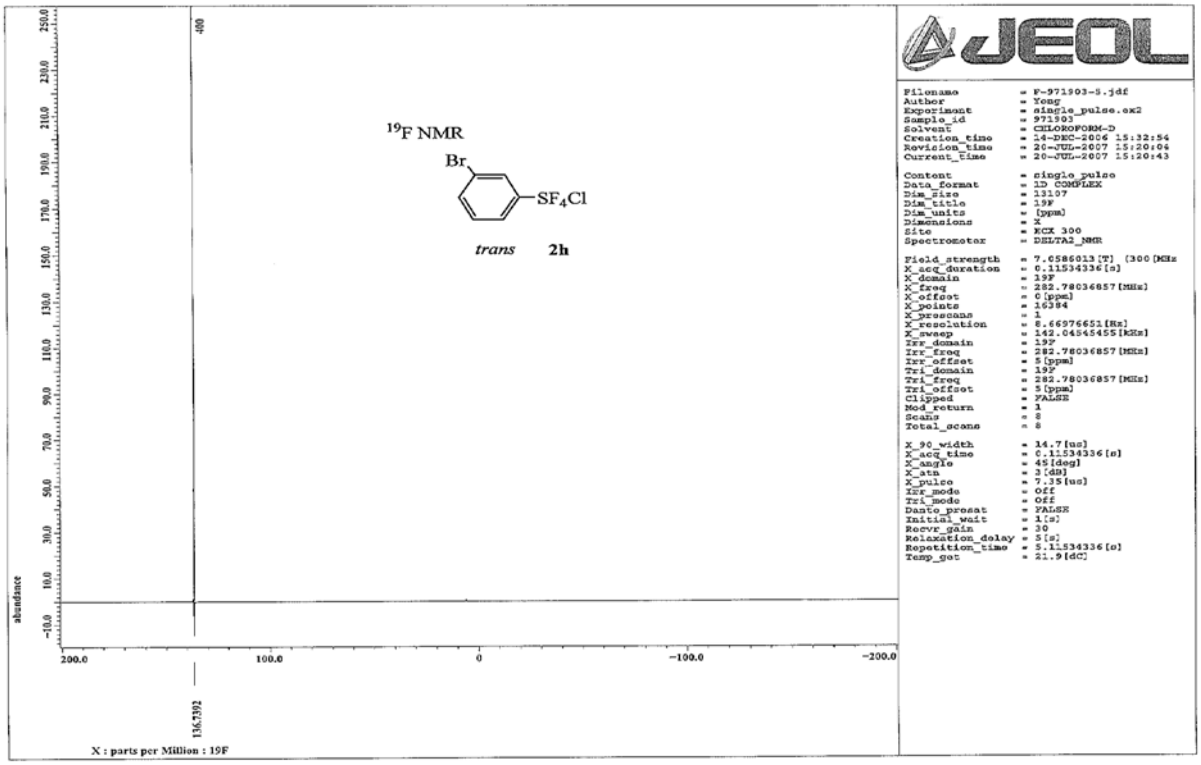

19F NMR
Br
SF4Cl
trans 2h
136.7392
X : parts per Million : 19F
abundance
Filename = F-971903-5.jdf
Author = Yong
Experiment = single_pulse.ex2
Sample_id = 971903
Solvent = CHLOROFORM-D
Creation_time = 14-DEC-2006 15:32:54
Revision_time = 20-JUL-2007 15:20:04
Current_time = 20-JUL-2007 15:20:43
Content = single_pulse
Data_format = 1D COMPLEX
Dim_size = 13107
Dim_title = 19F
Dim_units = [ppm]
Dimensions = X
Site = ECX 300
Spectrometer = DELTA2_NMR
Field_strength = 7.0586013[T] (300[MHz
X_acq_duration = 0.11534336[s]
X_domain = 19F
X_freq = 282.78036857[MHz]
X_offset = 0[ppm]
X_points = 16384
X_prescans = 1
X_resolution = 8.66976651[Hz]
X_sweep = 142.04545455[kHz]
Irr_domain = 19F
Irr_freq = 282.78036857[MHz]
Irr_offset = 5[ppm]
Tri_domain = 19F
Tri_freq = 282.78036857[MHz]
Tri_offset = 5[ppm]
Clipped = FALSE
Mod_return = 1
Scans = 8
Total_scans = 8
X_90_width = 14.7[us]
X_acq_time = 0.11534336[s]
X_angle = 45[deg]
X_atn = 3[dB]
X_pulse = 7.35[us]
Irr_mode = Off
Tri_mode = Off
Dante_presat = FALSE
Initial_wait = 1[s]
Recvr_gain = 30
Relaxation_delay = 5[s]
Repetition_time = 5.11534336[s]
Temp_get = 21.9[dC]

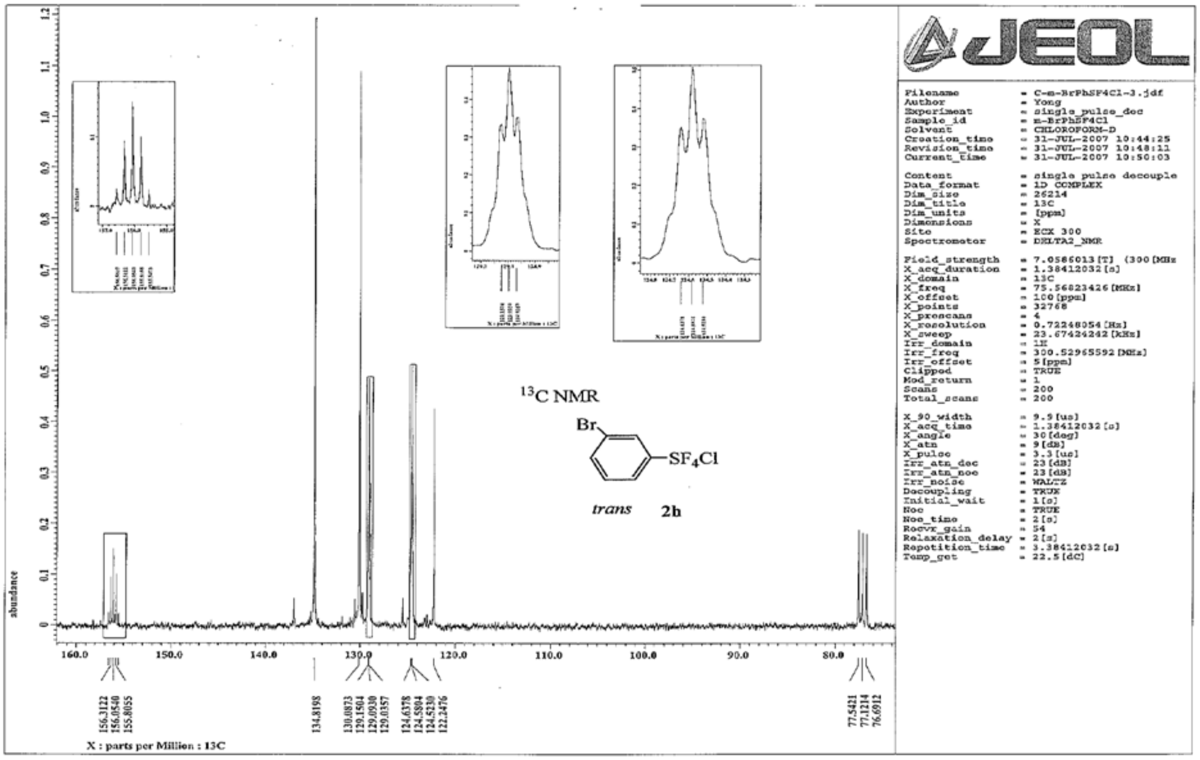

13C NMR
Br
SF4Cl
trans 2h
156.3122
156.0540
155.8055
134.8198
130.0873
129.1504
129.0930
129.0357
124.6378
124.5804
124.5230
122.2476
77.5421
77.1214
76.6912
X : parts per Million : 13C
abundance
Filename = C-m-BrPhSF4Cl-3.jdf
Author = Yong
Experiment = single_pulse_dec
Sample_id = m-BrPhSF4Cl
Solvent = CHLOROFORM-D
Creation_time = 31-JUL-2007 10:44:25
Revision_time = 31-JUL-2007 10:48:11
Current_time = 31-JUL-2007 10:50:03
Content = single pulse decouple
Data_format = 1D COMPLEX
Dim_size = 26214
Dim_title = 13C
Dim_units = [ppm]
Dimensions = X
Site = ECX 300
Spectrometer = DELTA2_NMR
Field_strength = 7.0586013[T] (300[MHz
X_acq_duration = 1.38412032[s]
X_domain = 13C
X_freq = 75.56823426[MHz]
X_offset = 100[ppm]
X_points = 32768
X_prescans = 4
X_resolution = 0.72248054[Hz]
X_sweep = 23.67424242[kHz]
Irr_domain = 1H
Irr_freq = 300.52965592[MHz]
Irr_offset = 5[ppm]
Clipped = TRUE
Mod_return = 1
Scans = 200
Total_scans = 200
X_90_width = 9.9[us]
X_acq_time = 1.38412032[s]
X_angle = 30[deg]
X_atn = 9[dB]
X_pulse = 3.3[us]
Irr_atn_dec = 23[dB]
Irr_atn_noe = 23[dB]
Irr_noise = WALTZ
Decoupling = TRUE
Initial_wait = 1[s]
Noe = TRUE
Noe_time = 2[s]
Recvr_gain = 54
Relaxation_delay = 2[s]
Repetition_time = 3.38412032[s]
Temp_get = 22.5[dC]

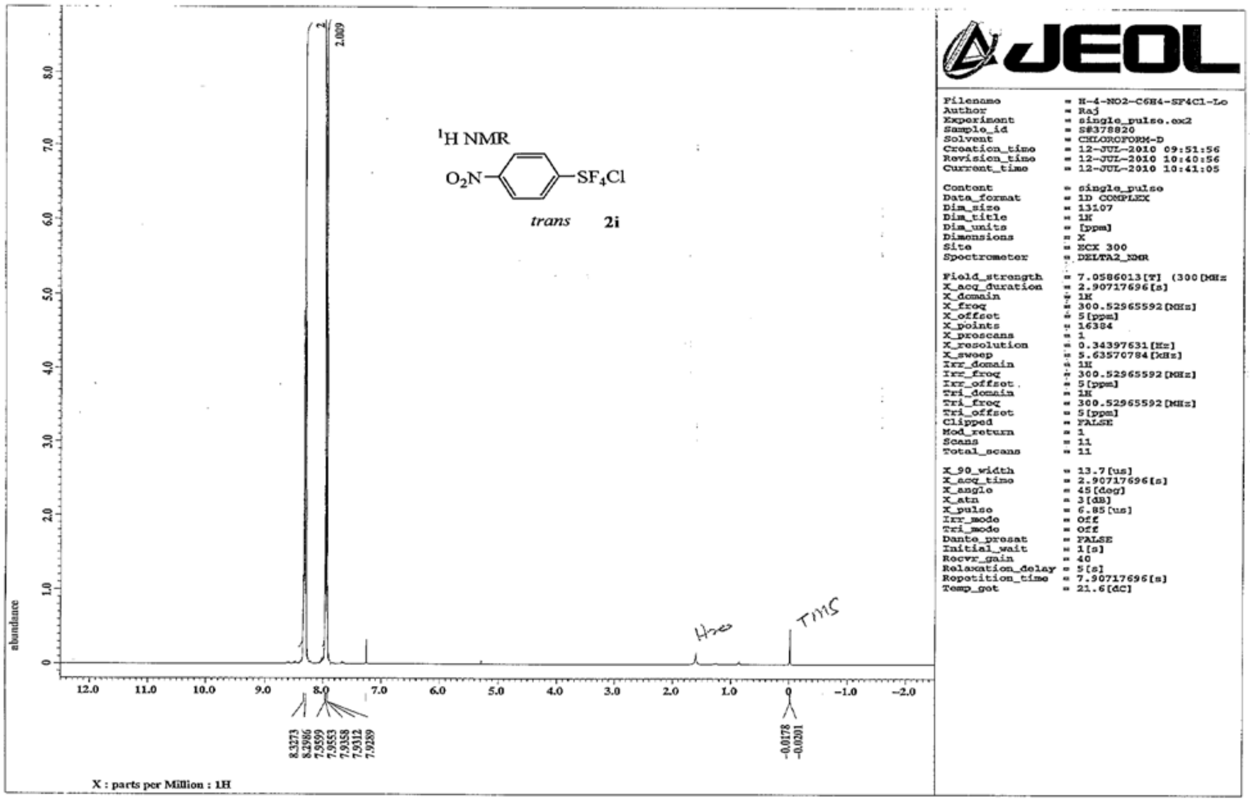
JEOL
^{1}H NMR
O_2N–C6H4–SF_4Cl
trans 2i
abundance
X : parts per Million : 1H
8.3273
8.2986
7.9599
7.9553
7.9358
7.9312
7.9289
-0.0178
-0.0201
TMS
2.009

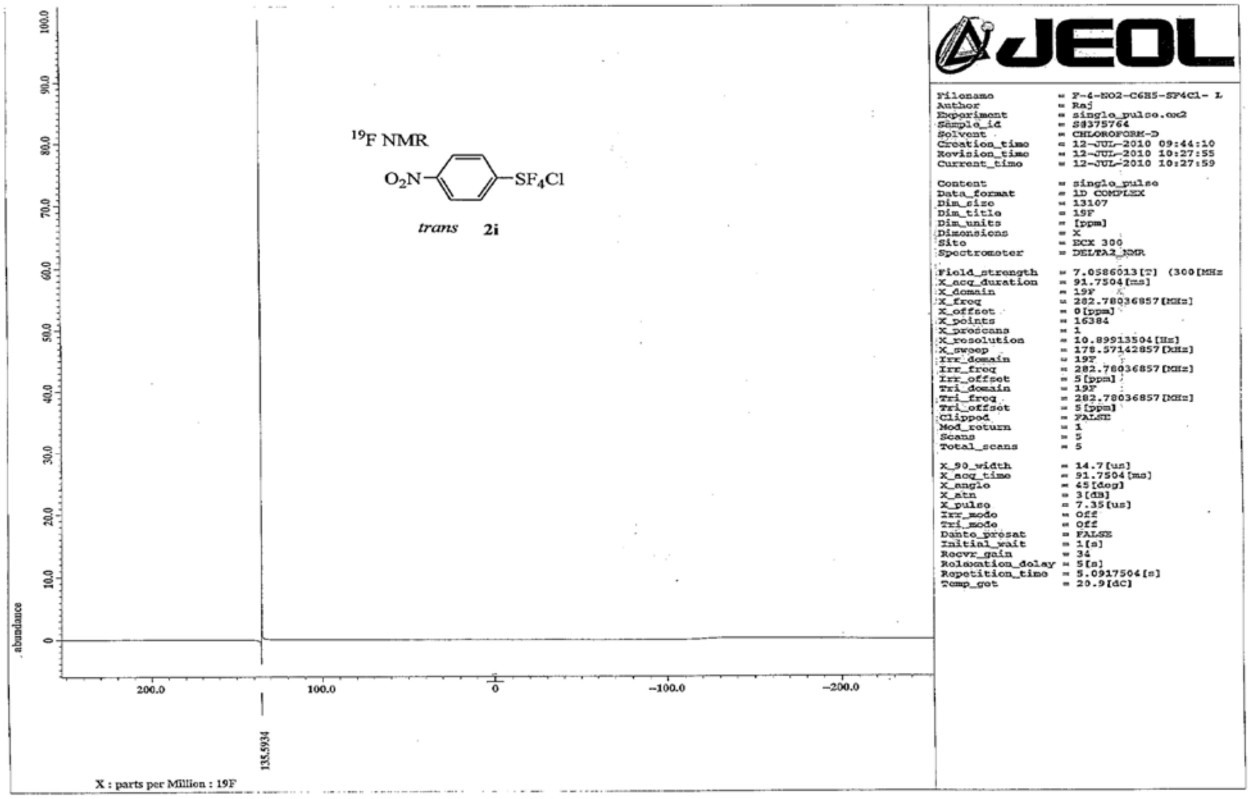
JEOL
^{19}F NMR
O_2N–C6H4–SF_4Cl
trans 2i
abundance
X : parts per Million : 19F
135.5934

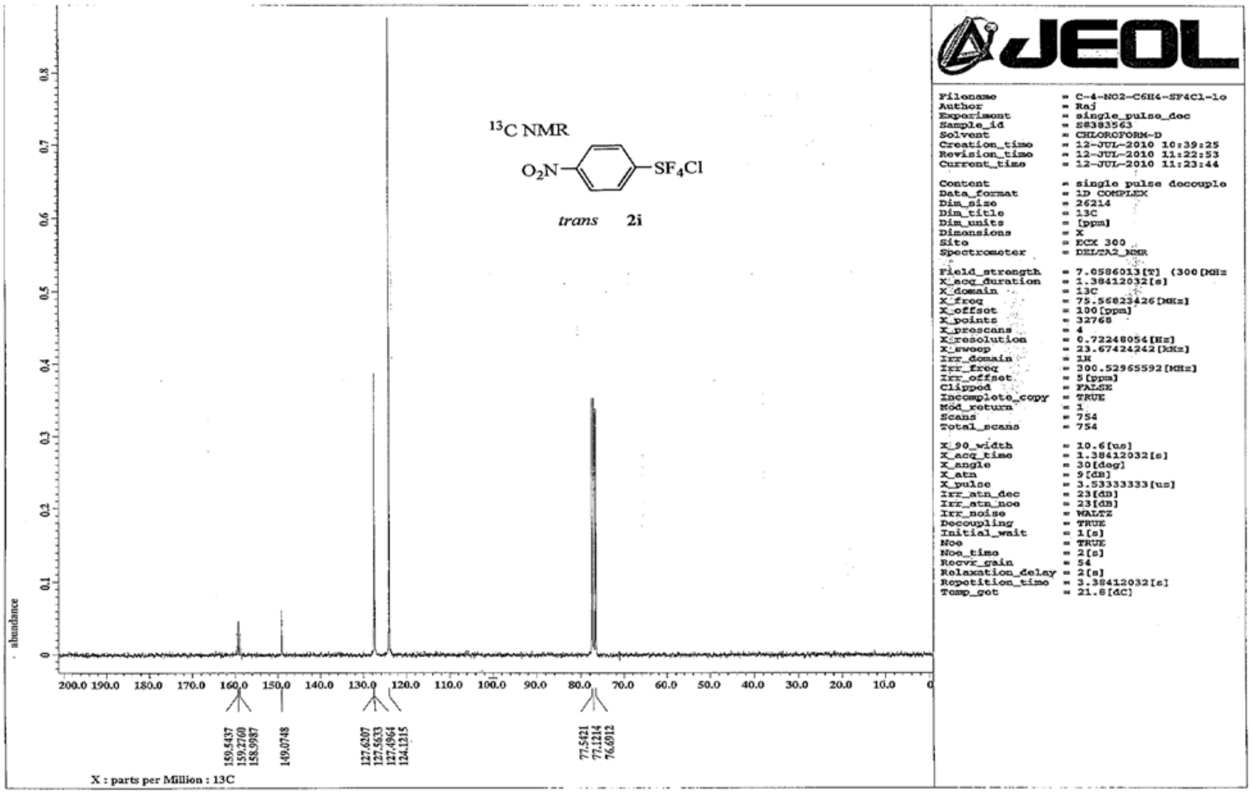

13C NMR
O2N–C6H4–SF4Cl
trans 2i
X : parts per Million : 13C
abundance
159.5437
159.2760
158.9987
149.0748
127.6207
127.5633
127.4964
124.1215
77.5421
77.1214
76.6912
JEOL
Filename = C-4-NO2-C6H4-SF4Cl-1o
Author = Raj
Experiment = single_pulse_dec
Solvent = CHLOROFORM-D
Spectrometer = DELTA2_NMR

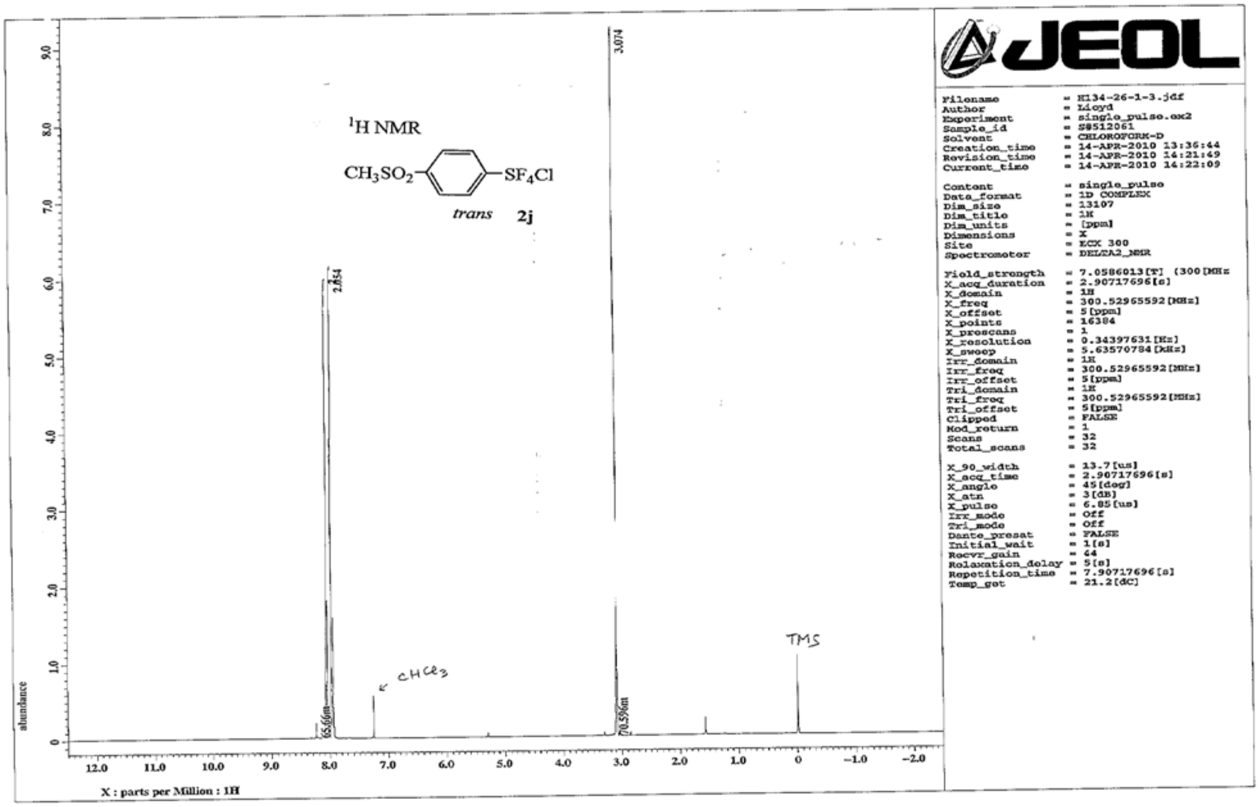

1H NMR
CH3SO2–C6H4–SF4Cl
trans 2j
X : parts per Million : 1H
abundance
3.074
2.054
CHCl3
TMS
JEOL
Filename = H134-26-1-3.jdf
Author = Lloyd
Experiment = single_pulse.ex2
Solvent = CHLOROFORM-D
Spectrometer = DELTA2_NMR

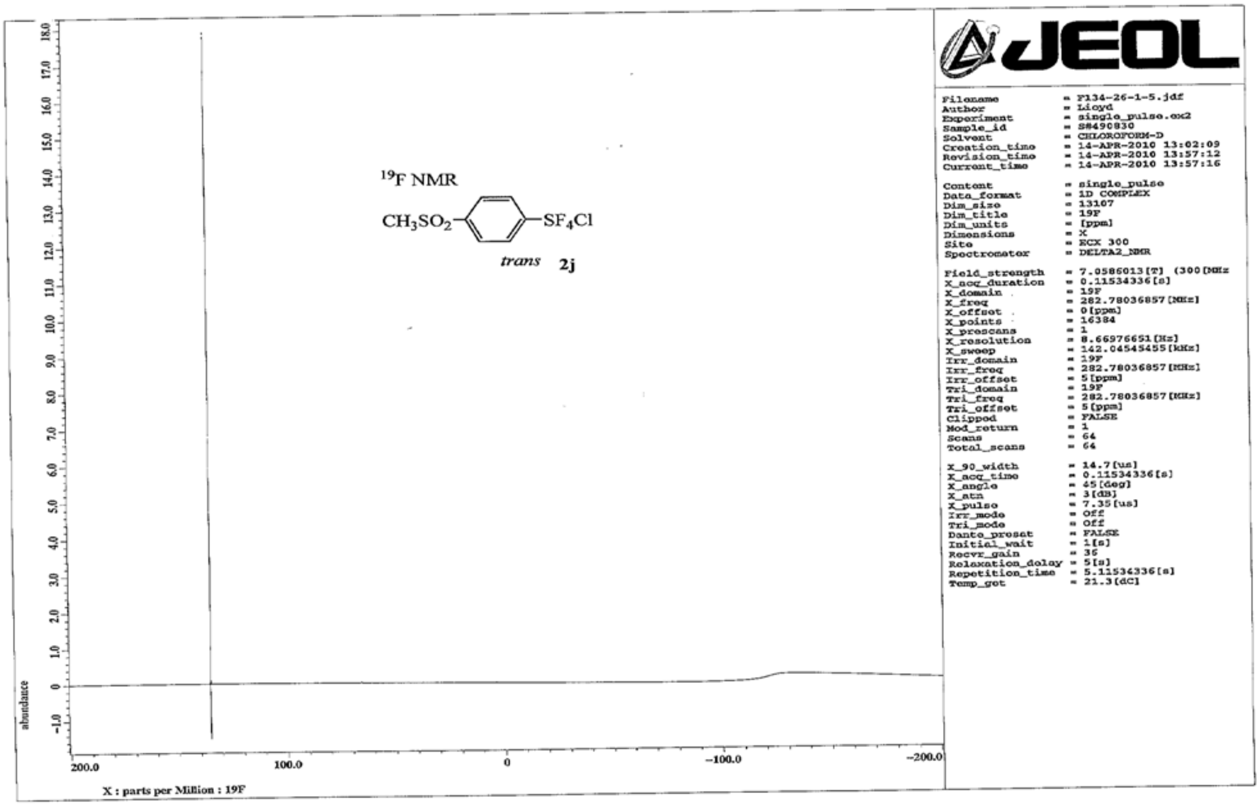

19F NMR
CH3SO2 SF4Cl
trans 2j
Filename = F134-26-1-5.jdf
Author = Lioyd
Experiment = single_pulse.ex2
Sample_id = S#490830
Solvent = CHLOROFORM-D
Creation_time = 14-APR-2010 13:02:09
Revision_time = 14-APR-2010 13:57:12
Current_time = 14-APR-2010 13:57:16
Content = single_pulse
Data_format = 1D COMPLEX
Dim_size = 13107
Dim_title = 19F
Dim_units = [ppm]
Dimensions = X
Site = ECX 300
Spectrometer = DELTA2_NMR
Field_strength = 7.0586013[T] (300[MHz
X_acq_duration = 0.11534336[s]
X_domain = 19F
X_freq = 282.78036857[MHz]
X_offset = 0[ppm]
X_points = 16384
X_prescans = 1
X_resolution = 8.66976651[Hz]
X_sweep = 142.04545455[kHz]
Irr_domain = 19F
Irr_freq = 282.78036857[MHz]
Irr_offset = 5[ppm]
Tri_domain = 19F
Tri_freq = 282.78036857[MHz]
Tri_offset = 5[ppm]
Clipped = FALSE
Mod_return = 1
Scans = 64
Total_scans = 64
X_90_width = 14.7[us]
X_acq_time = 0.11534336[s]
X_angle = 45[deg]
X_atn = 3[dB]
X_pulse = 7.35[us]
Irr_mode = Off
Tri_mode = Off
Dante_presat = FALSE
Initial_wait = 1[s]
Recvr_gain = 36
Relaxation_delay = 5[s]
Repetition_time = 5.11534336[s]
Temp_get = 21.3[dC]
abundance
X : parts per Million : 19F

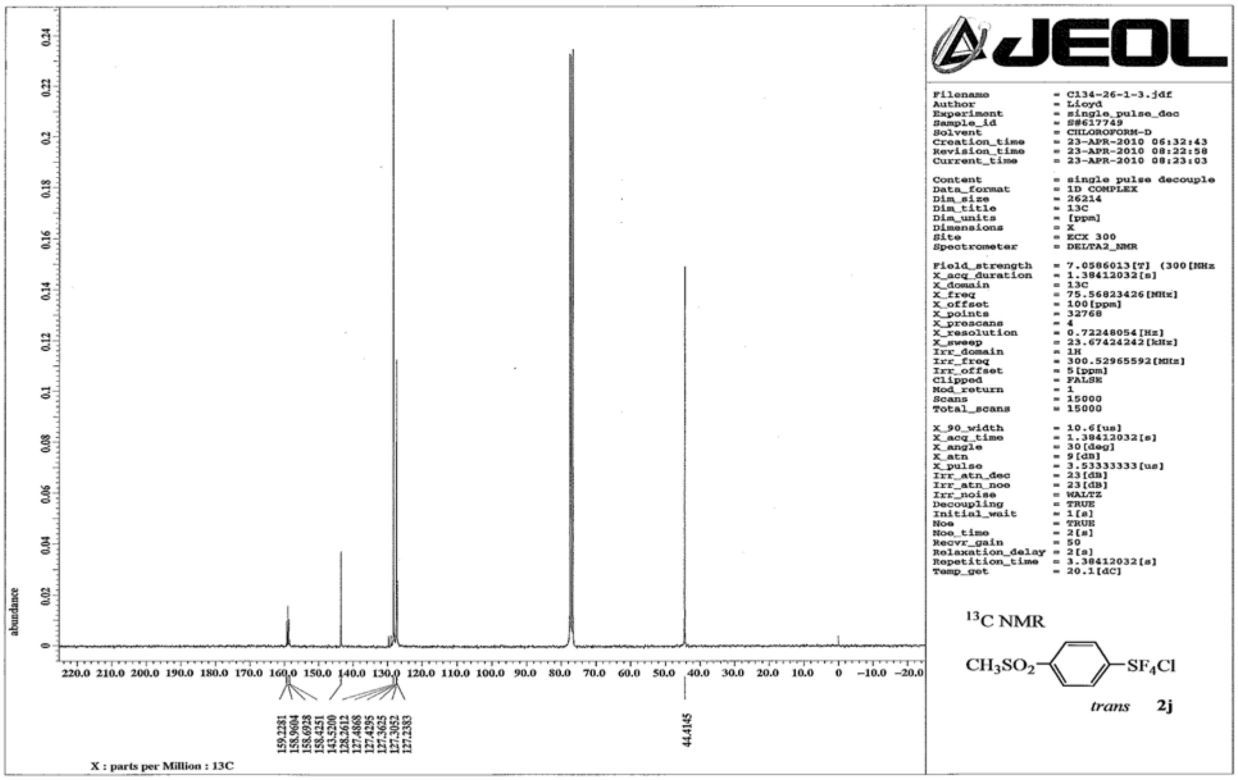

Filename = C134-26-1-3.jdf
Author = Lioyd
Experiment = single_pulse_dec
Sample_id = S#617749
Solvent = CHLOROFORM-D
Creation_time = 23-APR-2010 06:32:43
Revision_time = 23-APR-2010 08:22:58
Current_time = 23-APR-2010 08:23:03
Content = single pulse decouple
Data_format = 1D COMPLEX
Dim_size = 26214
Dim_title = 13C
Dim_units = [ppm]
Dimensions = X
Site = ECX 300
Spectrometer = DELTA2_NMR
Field_strength = 7.0586013[T] (300[MHz
X_acq_duration = 1.38412032[s]
X_domain = 13C
X_freq = 75.56823426[MHz]
X_offset = 100[ppm]
X_points = 32768
X_prescans = 4
X_resolution = 0.72248054[Hz]
X_sweep = 23.67424242[kHz]
Irr_domain = 1H
Irr_freq = 300.52965592[MHz]
Irr_offset = 5[ppm]
Clipped = FALSE
Mod_return = 1
Scans = 15000
Total_scans = 15000
X_90_width = 10.6[us]
X_acq_time = 1.38412032[s]
X_angle = 30[deg]
X_atn = 9[dB]
X_pulse = 3.53333333[us]
Irr_atn_dec = 23[dB]
Irr_atn_noe = 23[dB]
Irr_noise = WALTZ
Decoupling = TRUE
Initial_wait = 1[s]
Noe = TRUE
Noe_time = 2[s]
Recvr_gain = 50
Relaxation_delay = 2[s]
Repetition_time = 3.38412032[s]
Temp_get = 20.1[dC]
13C NMR
CH3SO2 SF4Cl
trans 2j
159.2281
158.9604
158.6928
158.4251
143.5200
128.2612
127.4868
127.4295
127.3625
127.3052
127.2383
44.4145
abundance
X : parts per Million : 13C

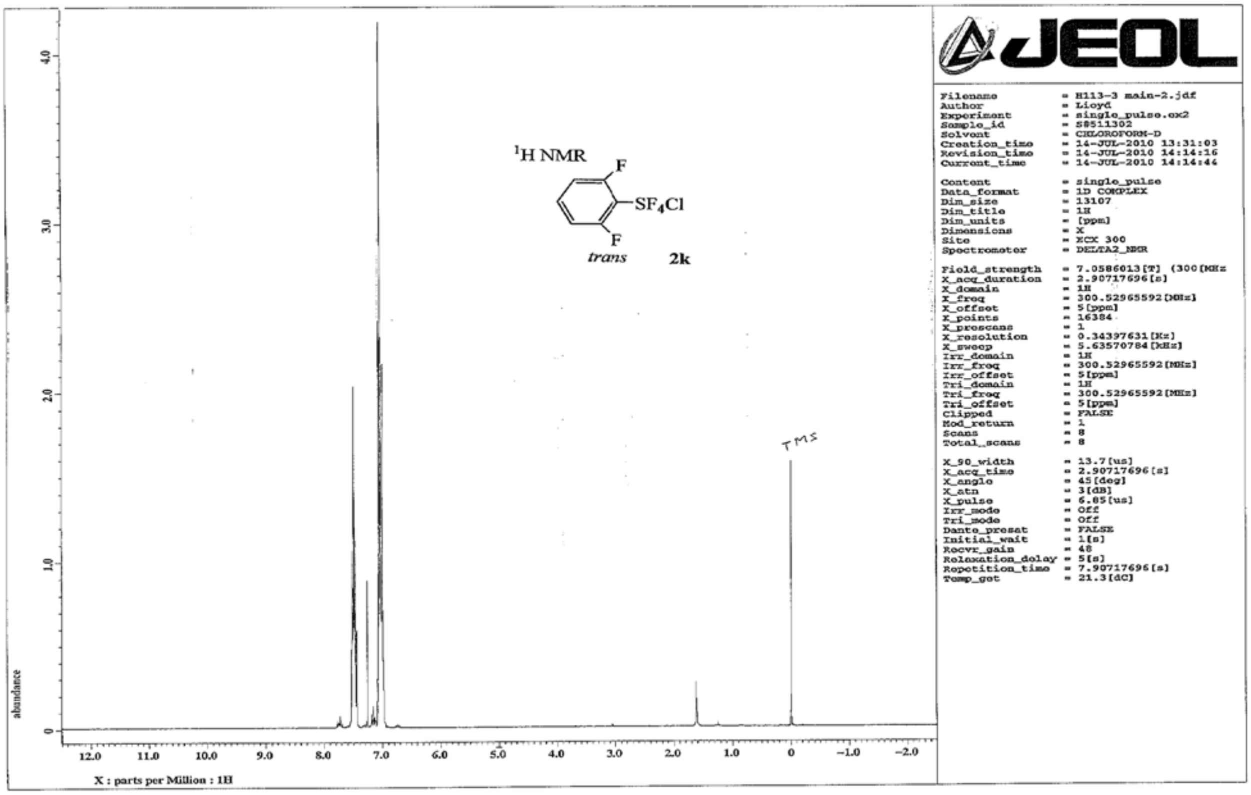

1H NMR
F
SF4Cl
F
trans
2k
TMS
abundance
X : parts per Million : 1H
JEOL
Filename = H113-3 main-2.jdf
Author = Lioyd
Experiment = single_pulse.ex2
Sample_id = S8511302
Solvent = CHLOROFORM-D
Creation_time = 14-JUL-2010 13:31:03
Revision_time = 14-JUL-2010 14:14:16
Current_time = 14-JUL-2010 14:14:44
Content = single_pulse
Data_format = 1D COMPLEX
Dim_size = 13107
Dim_title = 1H
Dim_units = [ppm]
Dimensions = X
Site = ECX 300
Spectrometer = DELTA2_NMR
Field_strength = 7.0586013[T] (300[MHz
X_acq_duration = 2.90717696[s]
X_domain = 1H
X_freq = 300.52965592[MHz]
X_offset = 5[ppm]
X_points = 16384
X_prescans = 1
X_resolution = 0.34397631[Hz]
X_sweep = 5.63570784[kHz]
Irr_domain = 1H
Irr_freq = 300.52965592[MHz]
Irr_offset = 5[ppm]
Tri_domain = 1H
Tri_freq = 300.52965592[MHz]
Tri_offset = 5[ppm]
Clipped = FALSE
Mod_return = 1
Scans = 8
Total_scans = 8
X_90_width = 13.7[us]
X_acq_time = 2.90717696[s]
X_angle = 45[deg]
X_atn = 3[dB]
X_pulse = 6.85[us]
Irr_mode = Off
Tri_mode = Off
Dante_presat = FALSE
Initial_wait = 1[s]
Recvr_gain = 48
Relaxation_delay = 5[s]
Repetition_time = 7.90717696[s]
Temp_get = 21.3[dC]

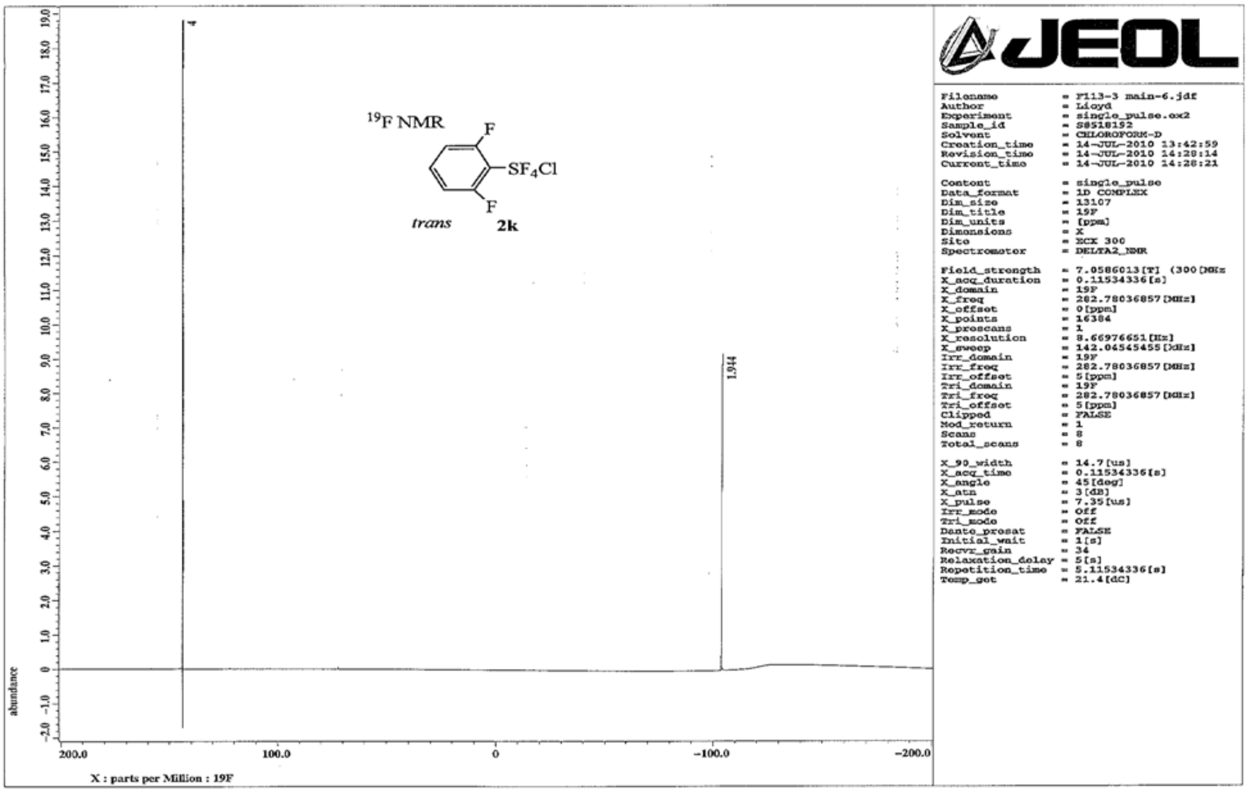

19F NMR
F
SF4Cl
F
trans
2k
1.944
abundance
X : parts per Million : 19F
JEOL
Filename = F113-3 main-6.jdf
Author = Lioyd
Experiment = single_pulse.ex2
Sample_id = S8518192
Solvent = CHLOROFORM-D
Creation_time = 14-JUL-2010 13:42:59
Revision_time = 14-JUL-2010 14:28:14
Current_time = 14-JUL-2010 14:28:21
Content = single_pulse
Data_format = 1D COMPLEX
Dim_size = 13107
Dim_title = 19F
Dim_units = [ppm]
Dimensions = X
Site = ECX 300
Spectrometer = DELTA2_NMR
Field_strength = 7.0586013[T] (300[MHz
X_acq_duration = 0.11534336[s]
X_domain = 19F
X_freq = 282.78036857[MHz]
X_offset = 0[ppm]
X_points = 16384
X_prescans = 1
X_resolution = 8.66976651[Hz]
X_sweep = 142.04545455[kHz]
Irr_domain = 19F
Irr_freq = 282.78036857[MHz]
Irr_offset = 5[ppm]
Tri_domain = 19F
Tri_freq = 282.78036857[MHz]
Tri_offset = 5[ppm]
Clipped = FALSE
Mod_return = 1
Scans = 8
Total_scans = 8
X_90_width = 14.7[us]
X_acq_time = 0.11534336[s]
X_angle = 45[deg]
X_atn = 3[dB]
X_pulse = 7.35[us]
Irr_mode = Off
Tri_mode = Off
Dante_presat = FALSE
Initial_wait = 1[s]
Recvr_gain = 34
Relaxation_delay = 5[s]
Repetition_time = 5.11534336[s]
Temp_get = 21.4[dC]

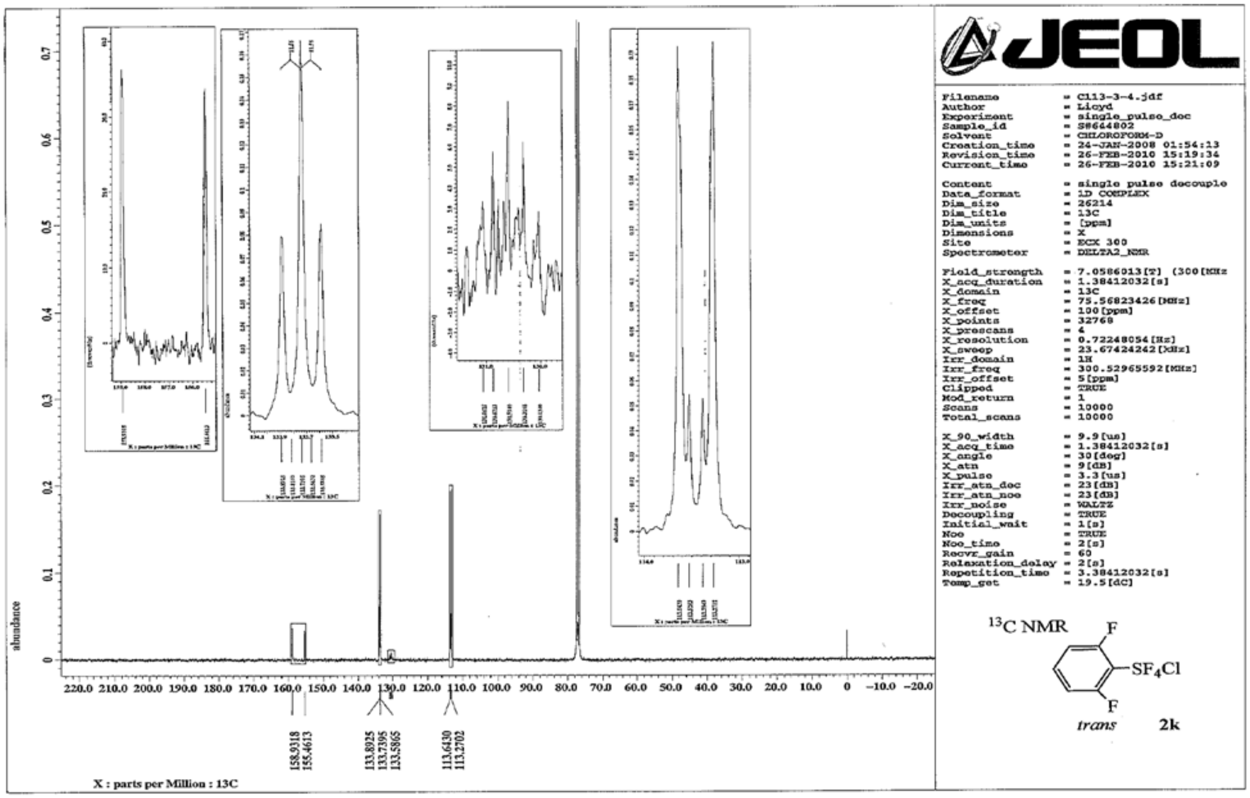
abundance
158.9318
155.4613
133.8925
133.7395
133.5865
113.6430
113.2702
X : parts per Million : 13C
JEOL
^{13}C NMR
F
SF$_4$Cl
F
trans
2k

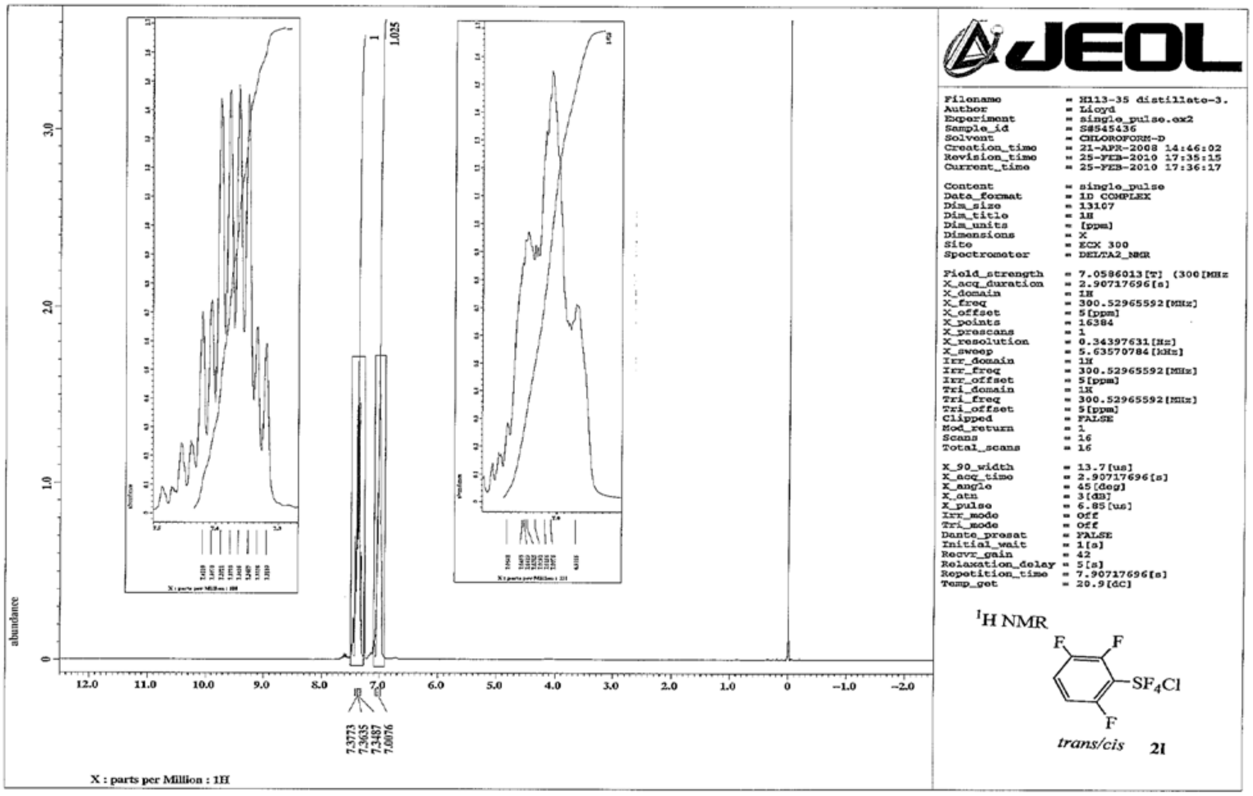
abundance
1
1.025
7.3773
7.3635
7.3487
7.0076
X : parts per Million : 1H
JEOL
^{1}H NMR
F
F
SF$_4$Cl
F
trans/cis
2l

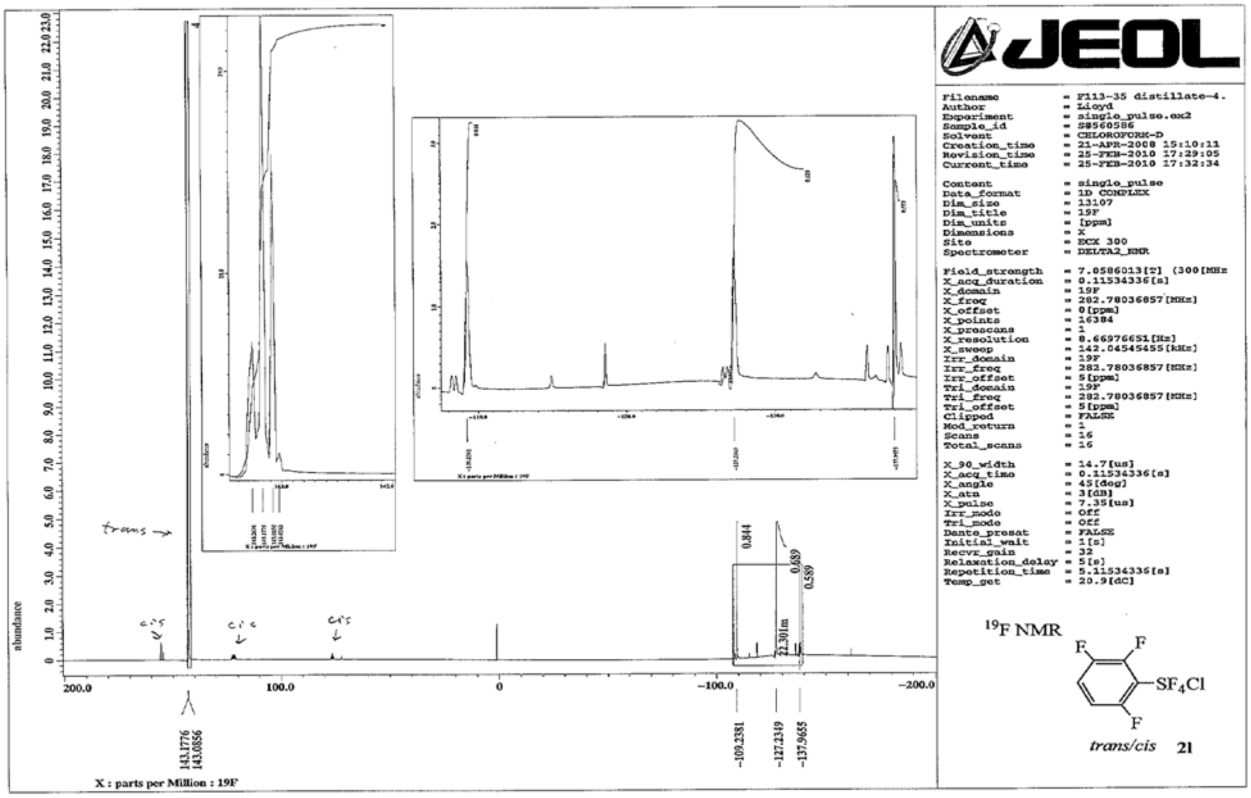

JEOL
19F NMR
SF4Cl
trans/cis 21
X : parts per Million : 19F
143.1776
143.0856
-109.2381
-127.2349
-137.9655
trans
cis

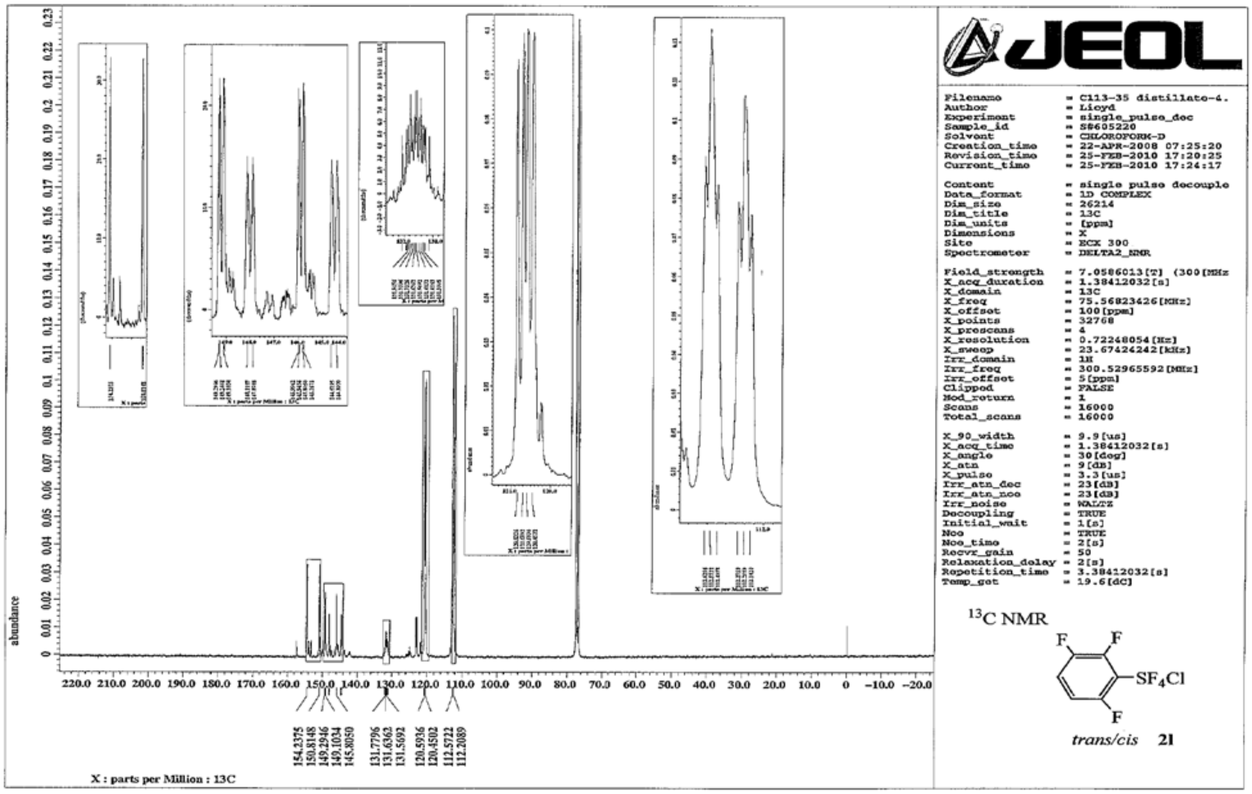

JEOL
13C NMR
SF4Cl
trans/cis 21
X : parts per Million : 13C
154.2375
150.8148
149.2946
149.1034
145.8050
131.7796
131.6363
131.5692
120.5936
120.4502
112.5722
112.2089

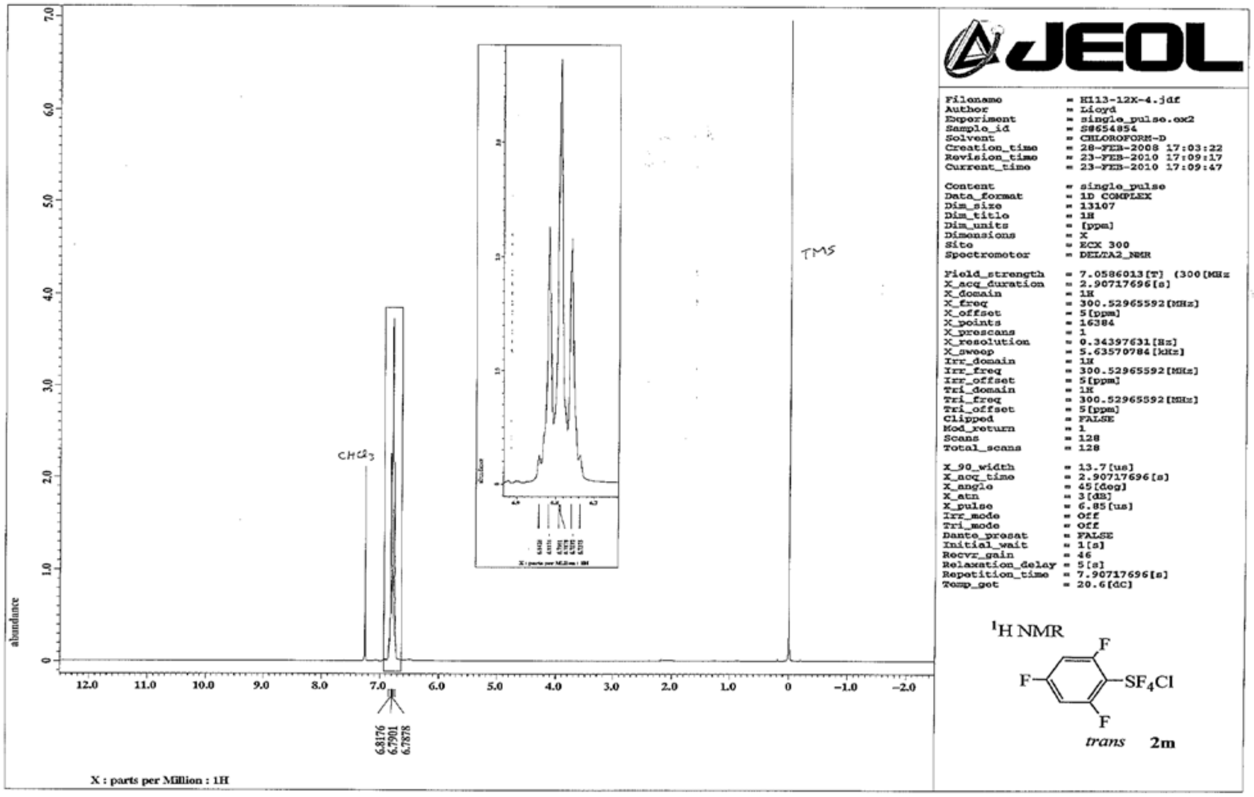
JEOL
Filename = H113-12X-4.jdf
Author = Lloyd
Experiment = single_pulse.ex2
Solvent = CHLOROFORM-D
Spectrometer = DELTA2_NMR
X : parts per Million : 1H
6.8176
6.7901
6.7878
CHCl3
TMS
abundance
1H NMR
F
SF4Cl
trans
2m

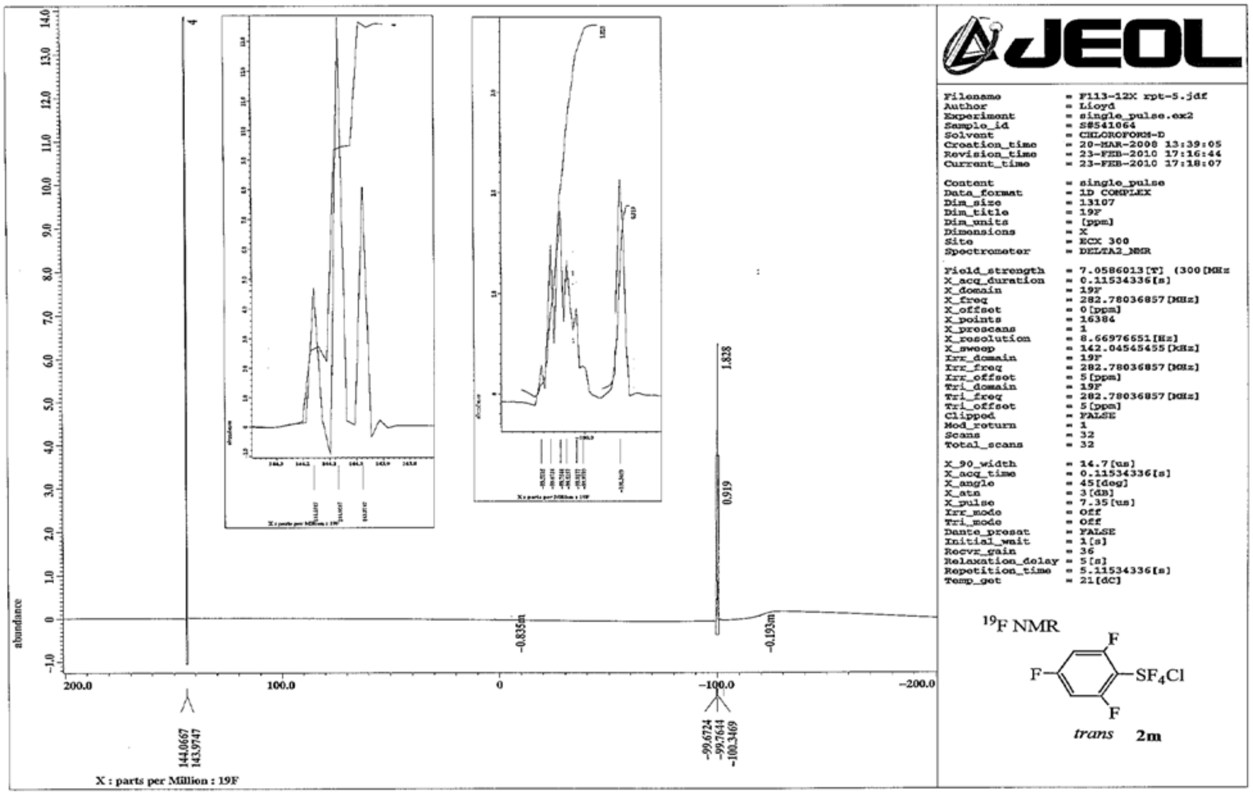
JEOL
Filename = F113-12X rpt-5.jdf
Author = Lloyd
Experiment = single_pulse.ex2
Solvent = CHLOROFORM-D
Spectrometer = DELTA2_NMR
X : parts per Million : 19F
144.0667
143.9747
-99.6724
-99.7644
-100.3469
1.828
0.919
abundance
19F NMR
F
SF4Cl
trans
2m

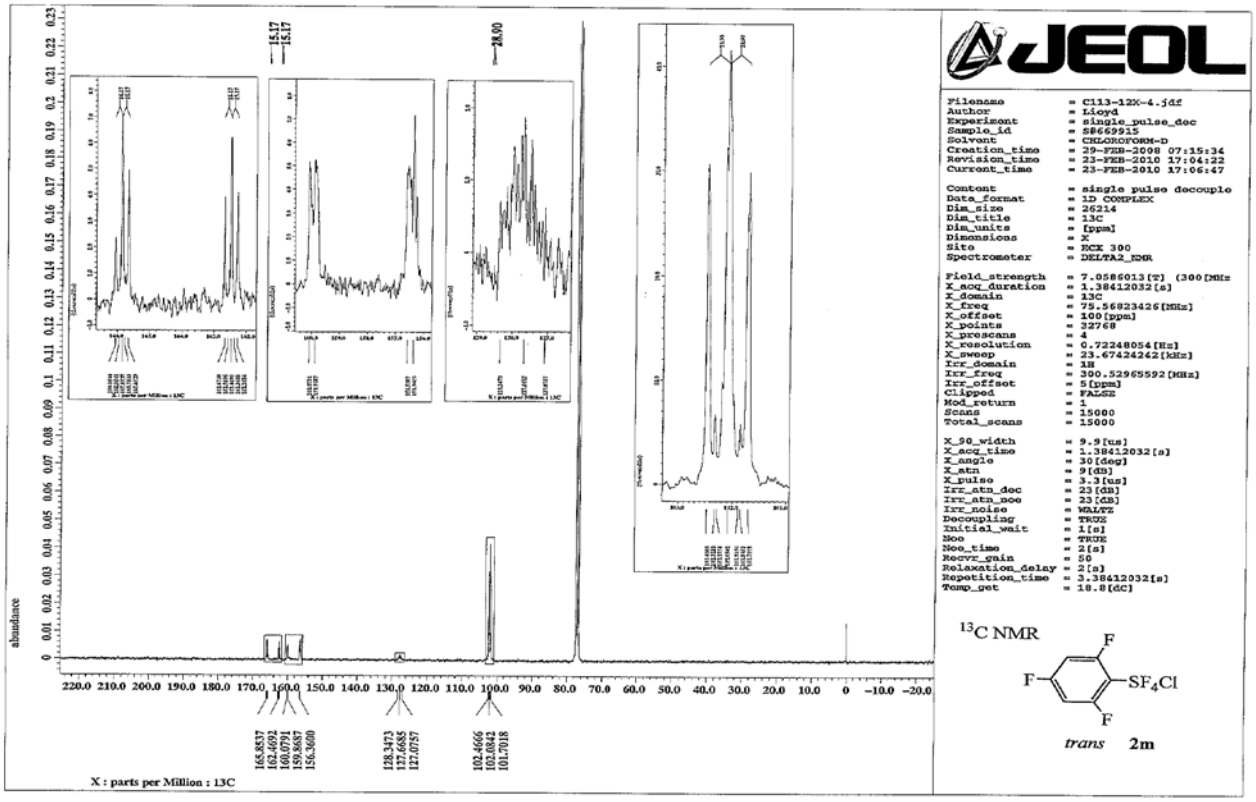
JEOL
13C NMR
F
SF4Cl
trans 2m
abundance
X : parts per Million : 13C
165.8837
162.4692
160.0791
159.8687
156.3600
128.3473
127.6685
127.0757
102.4666
102.0842
101.7018
15.17
28.90

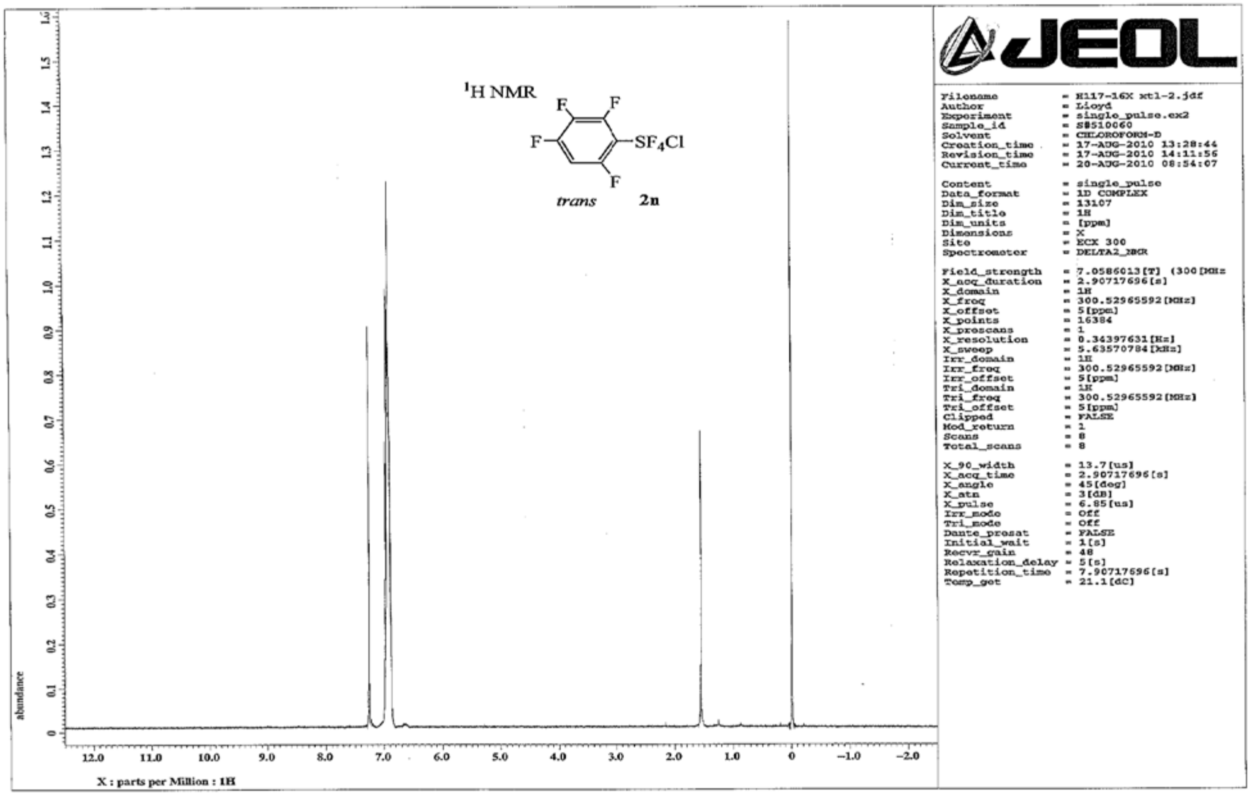
JEOL
1H NMR
F
SF4Cl
trans 2n
abundance
X : parts per Million : 1H

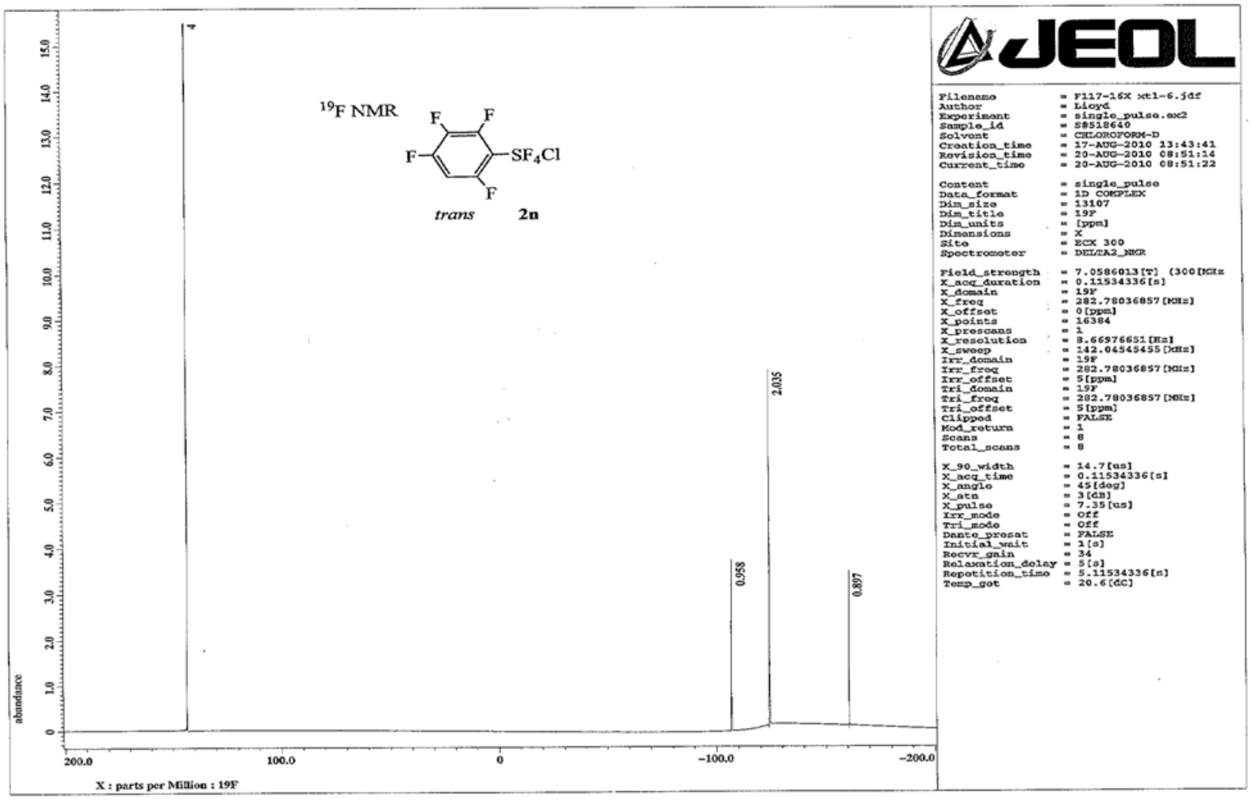

JEOL
19F NMR
SF4Cl
trans 2n
X : parts per Million : 19F
abundance
Filename = F117-16X xtl-6.jdf
Author = Lioyd
Experiment = single_pulse.ex2
Solvent = CHLOROFORM-D
Creation_time = 17-AUG-2010 13:43:41
Revision_time = 20-AUG-2010 08:51:14
Current_time = 20-AUG-2010 08:51:22
Spectrometer = DELTA2_NMR

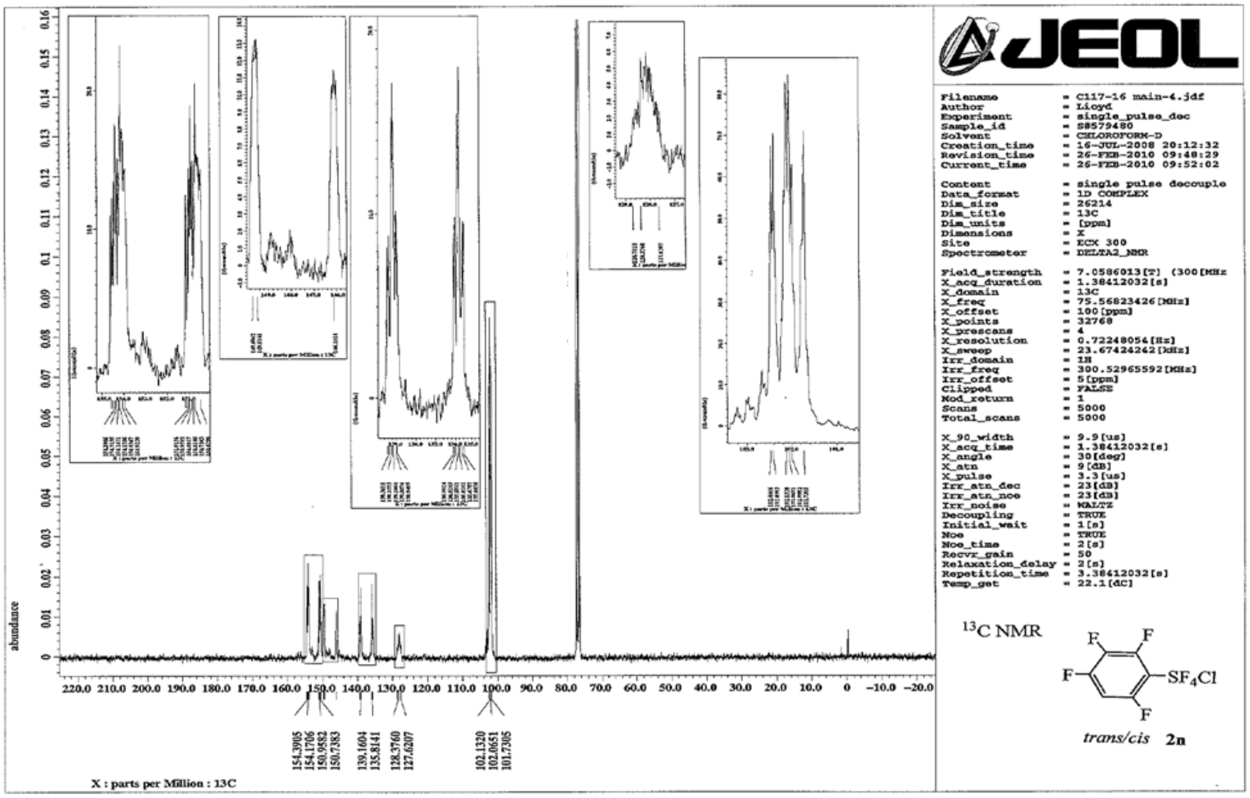

JEOL
13C NMR
SF4Cl
trans/cis 2n
X : parts per Million : 13C
abundance
154.3905
154.1706
150.9582
150.7383
139.1604
135.8141
128.3760
127.6207
102.1320
102.0651
101.7305
Filename = C117-16 main-4.jdf
Author = Lioyd
Experiment = single_pulse_dec
Solvent = CHLOROFORM-D
Creation_time = 16-JUL-2008 20:12:32
Revision_time = 26-FEB-2010 09:48:29
Current_time = 26-FEB-2010 09:52:02
Spectrometer = DELTA2_NMR

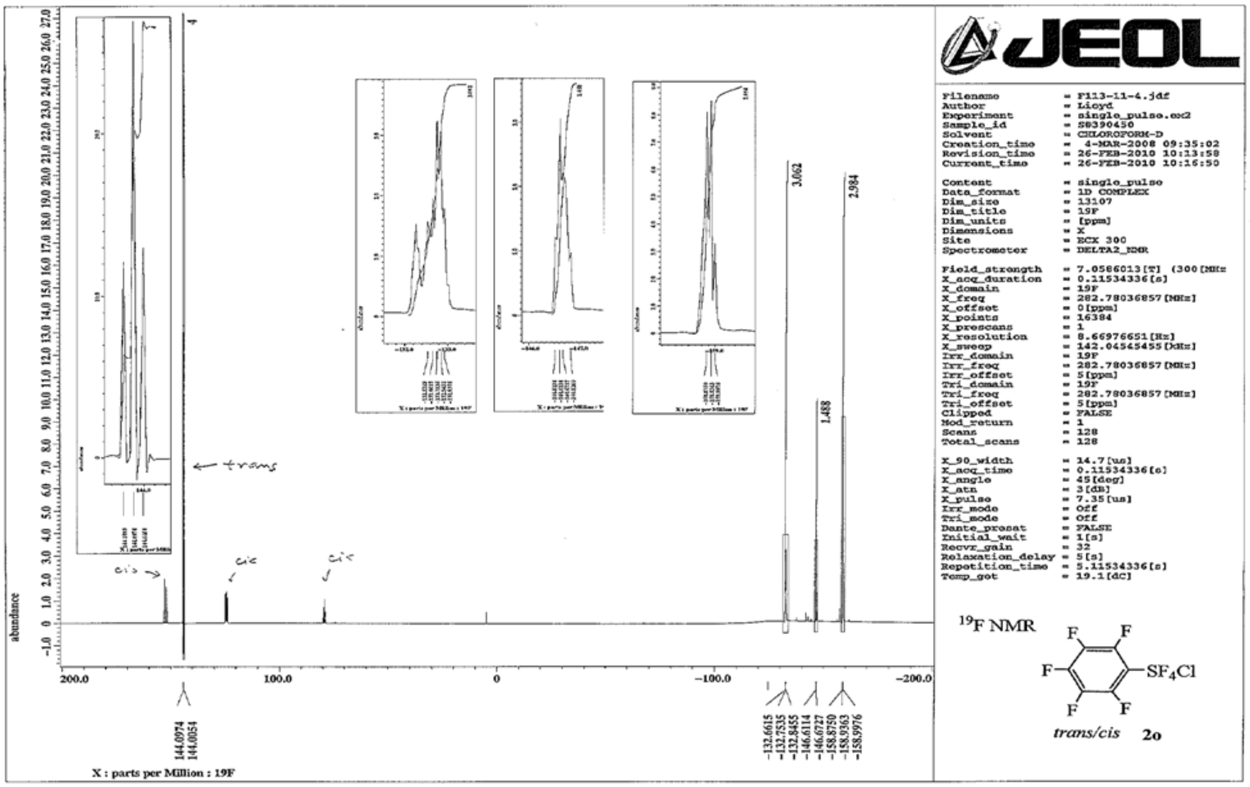

JEOL
Filename = F113-11-4.jdf
Author = Lloyd
Experiment = single_pulse.ex2
Solvent = CHLOROFORM-D
Creation_time = 4-MAR-2008 09:35:02
Revision_time = 26-FEB-2010 10:13:58
Current_time = 26-FEB-2010 10:16:50
Dim_title = 19F
Site = ECX 300
Spectrometer = DELTA2_NMR
Field_strength = 7.0586013[T] (300[MHz
X_freq = 282.78036857[MHz]
Scans = 128
Total_scans = 128
Temp_get = 19.1[dC]
trans
cis
3.062
1.488
2.984
144.0974
144.0054
-132.6615
-132.7535
-132.8455
-146.6114
-146.6727
-158.8750
-158.9363
-158.9976
X : parts per Million : 19F
^{19}F NMR
SF4Cl
trans/cis 2o

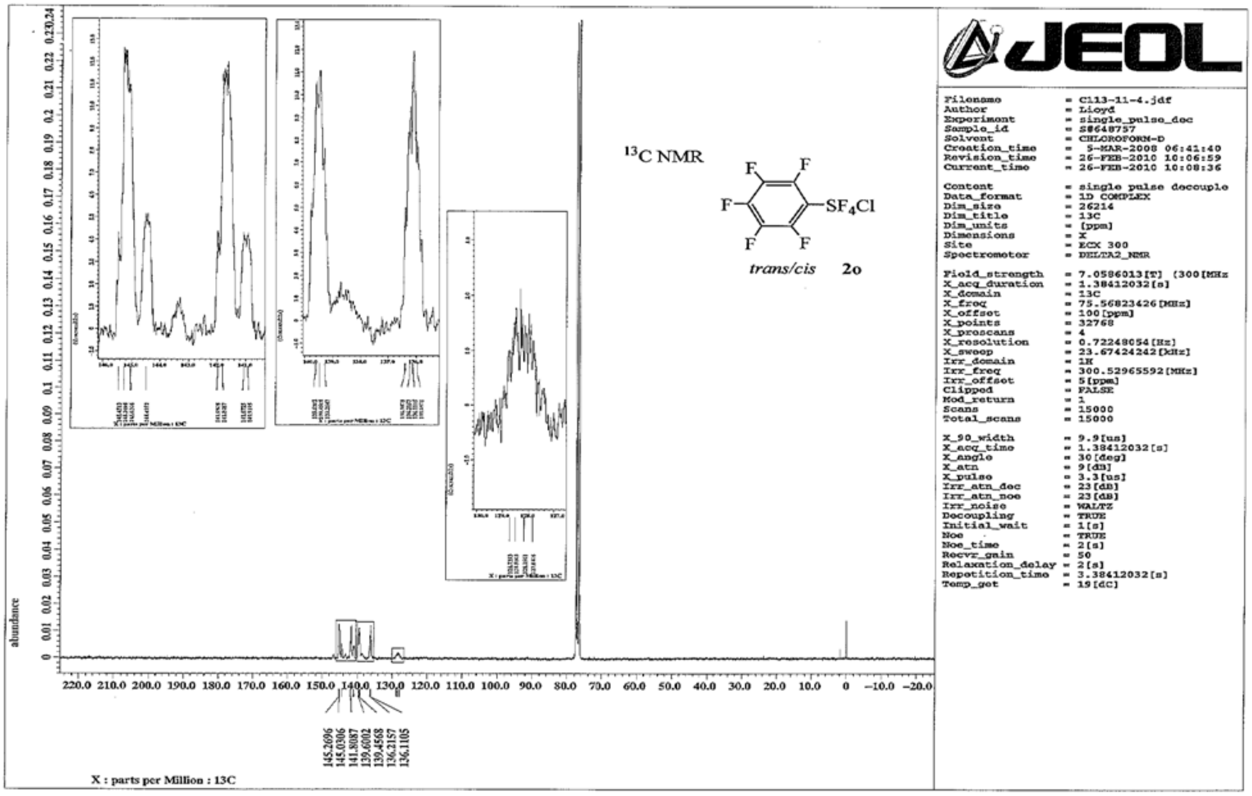

JEOL
Filename = C113-11-4.jdf
Author = Lloyd
Experiment = single_pulse_dec
Solvent = CHLOROFORM-D
Creation_time = 5-MAR-2008 06:41:40
Revision_time = 26-FEB-2010 10:06:59
Current_time = 26-FEB-2010 10:08:36
Dim_title = 13C
Site = ECX 300
Spectrometer = DELTA2_NMR
Field_strength = 7.0586013[T] (300[MHz
X_freq = 75.56823426[MHz]
Scans = 15000
Total_scans = 15000
Temp_get = 19[dC]
^{13}C NMR
SF4Cl
trans/cis 2o
145.2696
145.0306
141.8087
139.6002
139.4568
136.2157
136.1105
X : parts per Million : 13C

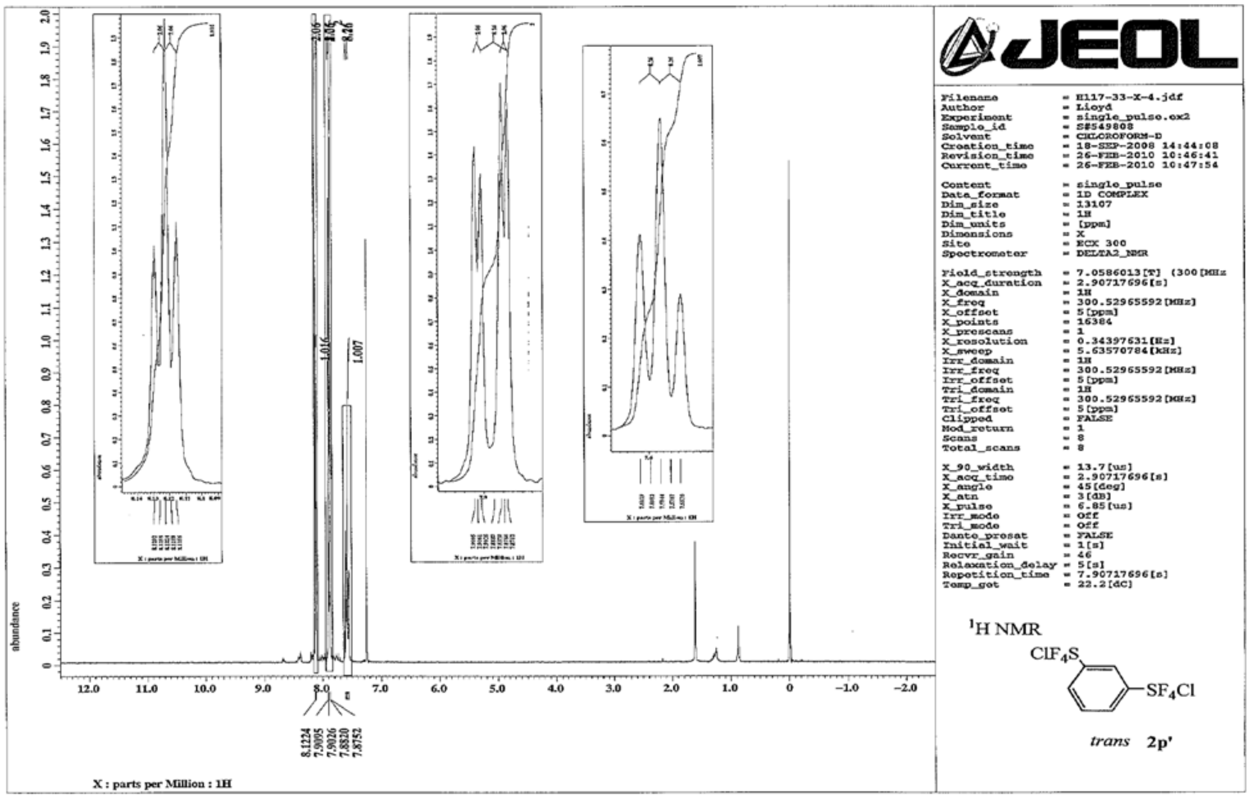
JEOL
Filename = H117-33-X-4.jdf
Author = Lioyd
Experiment = single_pulse.ex2
Sample_id = S#549808
Solvent = CHLOROFORM-D
Creation_time = 18-SEP-2008 14:44:08
Revision_time = 26-FEB-2010 10:46:41
Current_time = 26-FEB-2010 10:47:54
Content = single_pulse
Data_format = 1D COMPLEX
Dim_size = 13107
Dim_title = 1H
Dim_units = [ppm]
Dimensions = X
Site = ECX 300
Spectrometer = DELTA2_NMR
Field_strength = 7.0586013[T] (300[MHz
X_acq_duration = 2.90717696[s]
X_domain = 1H
X_freq = 300.52965592[MHz]
X_offset = 5[ppm]
X_points = 16384
X_prescans = 1
X_resolution = 0.34397631[Hz]
X_sweep = 5.63570784[kHz]
Irr_domain = 1H
Irr_freq = 300.52965592[MHz]
Irr_offset = 5[ppm]
Tri_domain = 1H
Tri_freq = 300.52965592[MHz]
Tri_offset = 5[ppm]
Clipped = FALSE
Mod_return = 1
Scans = 8
Total_scans = 8
X_90_width = 13.7[us]
X_acq_time = 2.90717696[s]
X_angle = 45[deg]
X_atn = 3[dB]
X_pulse = 6.85[us]
Irr_mode = Off
Tri_mode = Off
Dante_presat = FALSE
Initial_wait = 1[s]
Recvr_gain = 46
Relaxation_delay = 5[s]
Repetition_time = 7.90717696[s]
Temp_get = 22.2[dC]
1H NMR
ClF4S
SF4Cl
trans 2p'
2.06
1.016
1.007
8.1224
7.9095
7.9026
7.8820
7.8752
abundance
X : parts per Million : 1H

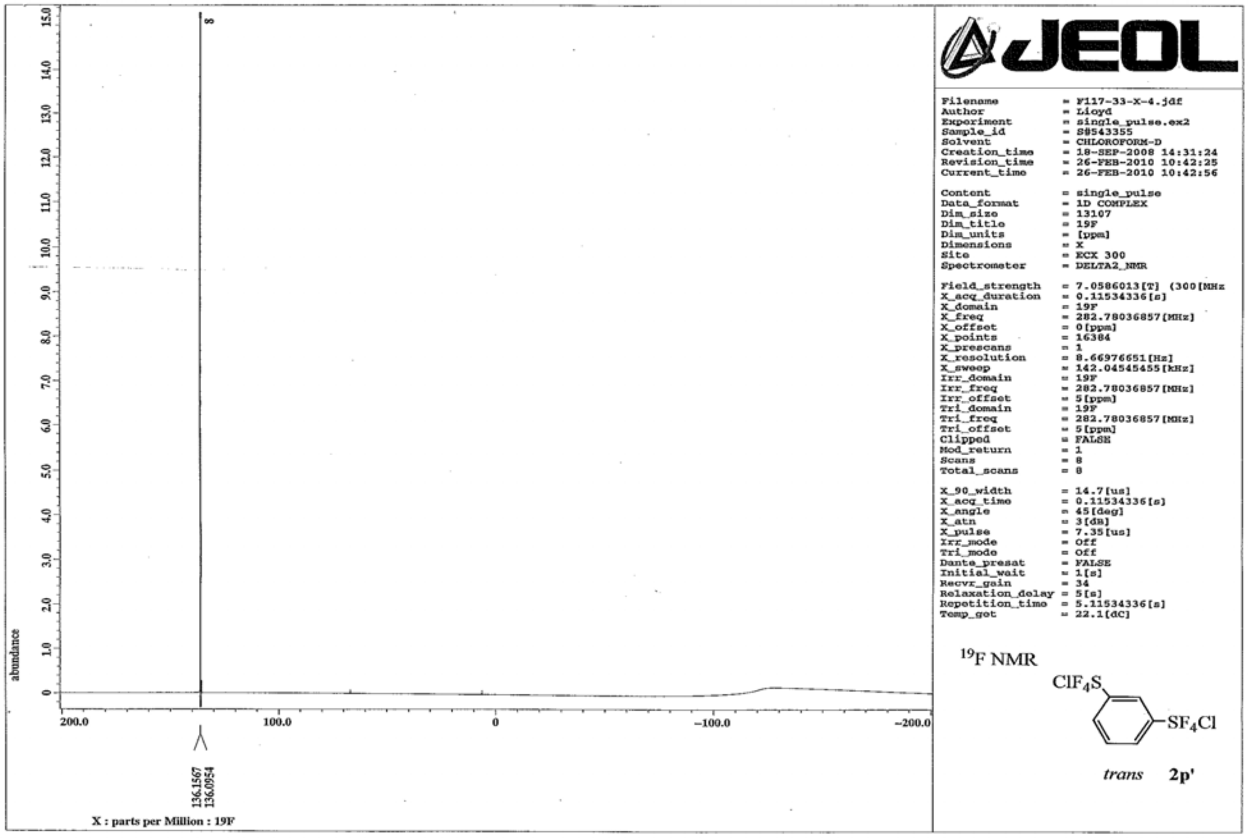
JEOL
Filename = F117-33-X-4.jdf
Author = Lioyd
Experiment = single_pulse.ex2
Sample_id = S#543355
Solvent = CHLOROFORM-D
Creation_time = 18-SEP-2008 14:31:24
Revision_time = 26-FEB-2010 10:42:25
Current_time = 26-FEB-2010 10:42:56
Content = single_pulse
Data_format = 1D COMPLEX
Dim_size = 13107
Dim_title = 19F
Dim_units = [ppm]
Dimensions = X
Site = ECX 300
Spectrometer = DELTA2_NMR
Field_strength = 7.0586013[T] (300[MHz
X_acq_duration = 0.11534336[s]
X_domain = 19F
X_freq = 282.78036857[MHz]
X_offset = 0[ppm]
X_points = 16384
X_prescans = 1
X_resolution = 8.66976651[Hz]
X_sweep = 142.04545455[kHz]
Irr_domain = 19F
Irr_freq = 282.78036857[MHz]
Irr_offset = 5[ppm]
Tri_domain = 19F
Tri_freq = 282.78036857[MHz]
Tri_offset = 5[ppm]
Clipped = FALSE
Mod_return = 1
Scans = 8
Total_scans = 8
X_90_width = 14.7[us]
X_acq_time = 0.11534336[s]
X_angle = 45[deg]
X_atn = 3[dB]
X_pulse = 7.35[us]
Irr_mode = Off
Tri_mode = Off
Dante_presat = FALSE
Initial_wait = 1[s]
Recvr_gain = 34
Relaxation_delay = 5[s]
Repetition_time = 5.11534336[s]
Temp_get = 22.1[dC]
19F NMR
ClF4S
SF4Cl
trans 2p'
136.1567
136.0954
abundance
X : parts per Million : 19F

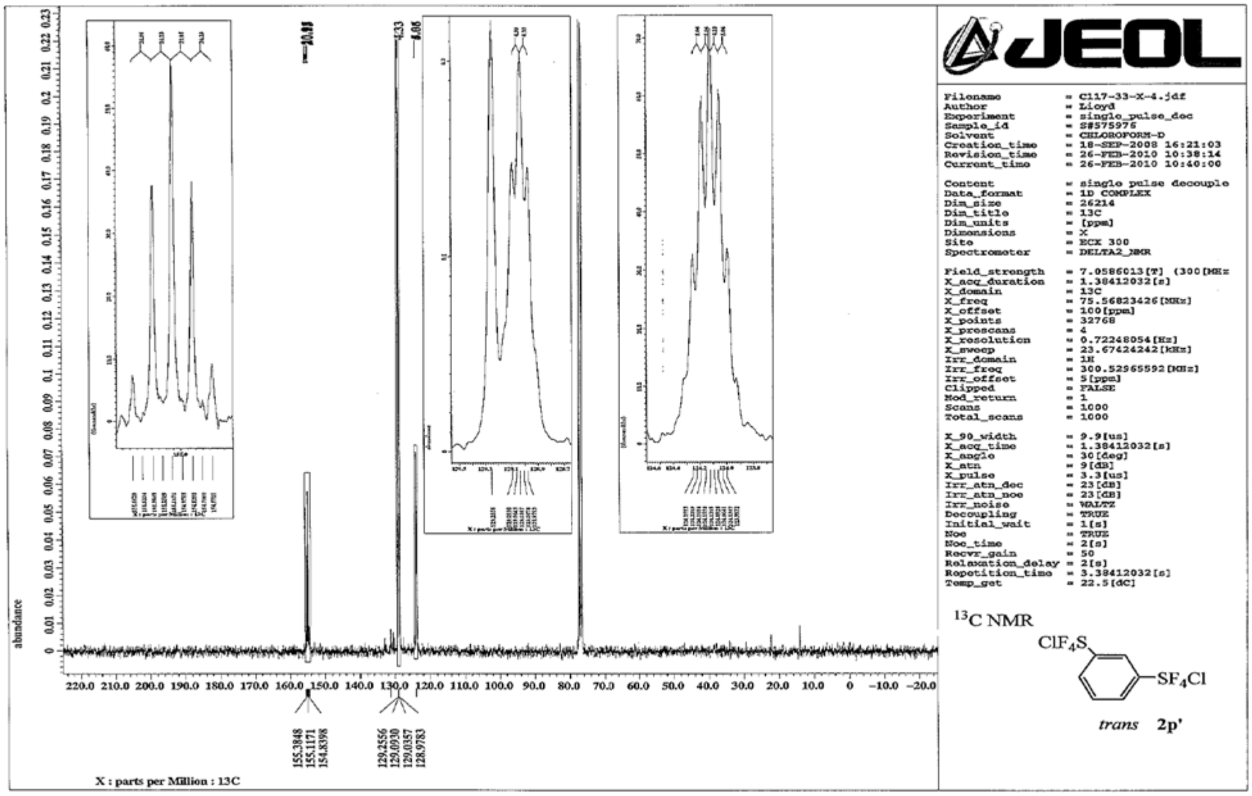
JEOL
Filename = C117-33-X-4.jdf
Author = Lloyd
Experiment = single_pulse_dec
Solvent = CHLOROFORM-D
Creation_time = 18-SEP-2008 16:21:03
Revision_time = 26-FEB-2010 10:38:14
Current_time = 26-FEB-2010 10:40:00
Content = single pulse decouple
Dim_title = 13C
Site = ECX 300
Spectrometer = DELTA2_NMR
Scans = 1000
Temp_get = 22.5[dC]
abundance
X : parts per Million : 13C
155.3848
155.1171
154.8398
129.2556
129.0930
129.0357
128.9783
^{13}C NMR
ClF_4S
SF_4Cl
trans 2p'

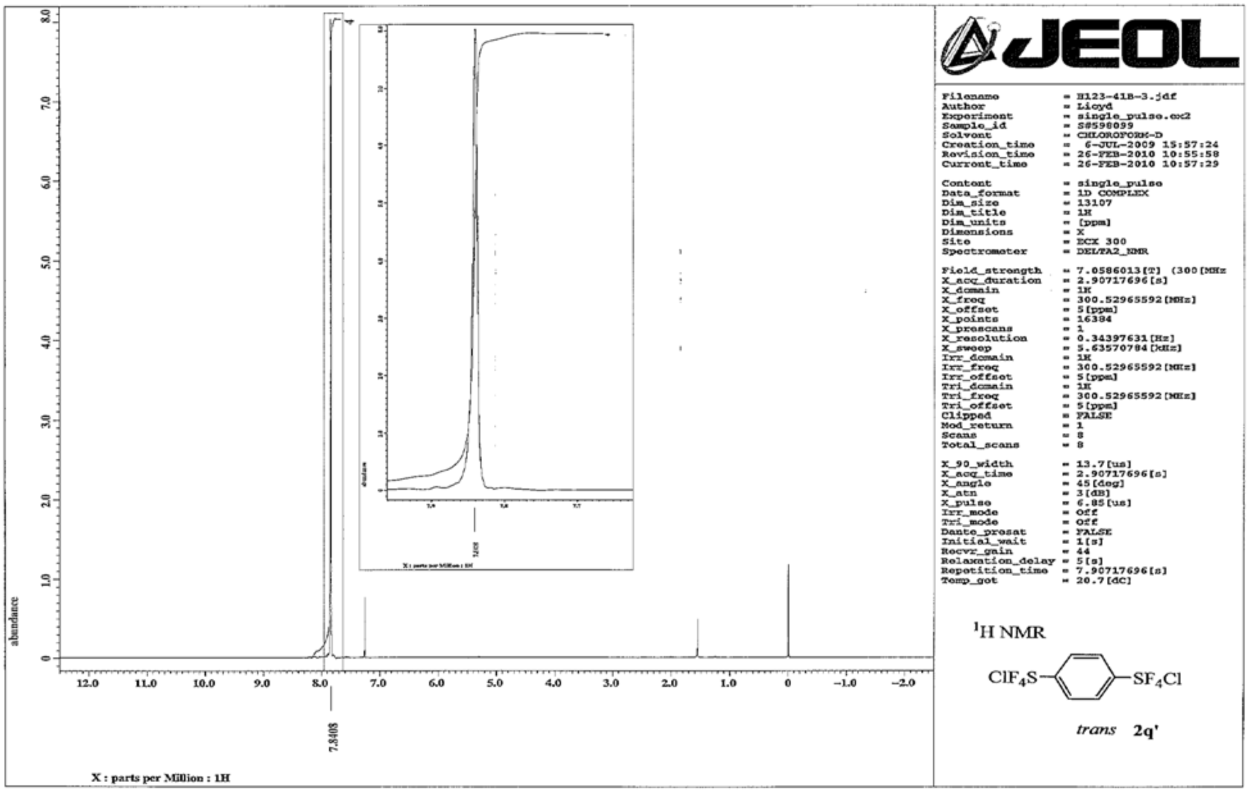
JEOL
Filename = H123-41B-3.jdf
Author = Lloyd
Experiment = single_pulse.ex2
Solvent = CHLOROFORM-D
Creation_time = 6-JUL-2009 15:57:24
Revision_time = 26-FEB-2010 10:55:58
Current_time = 26-FEB-2010 10:57:29
Content = single_pulse
Dim_title = 1H
Site = ECX 300
Spectrometer = DELTA2_NMR
Scans = 8
Temp_get = 20.7[dC]
abundance
X : parts per Million : 1H
7.8408
^{1}H NMR
ClF_4S
SF_4Cl
trans 2q'

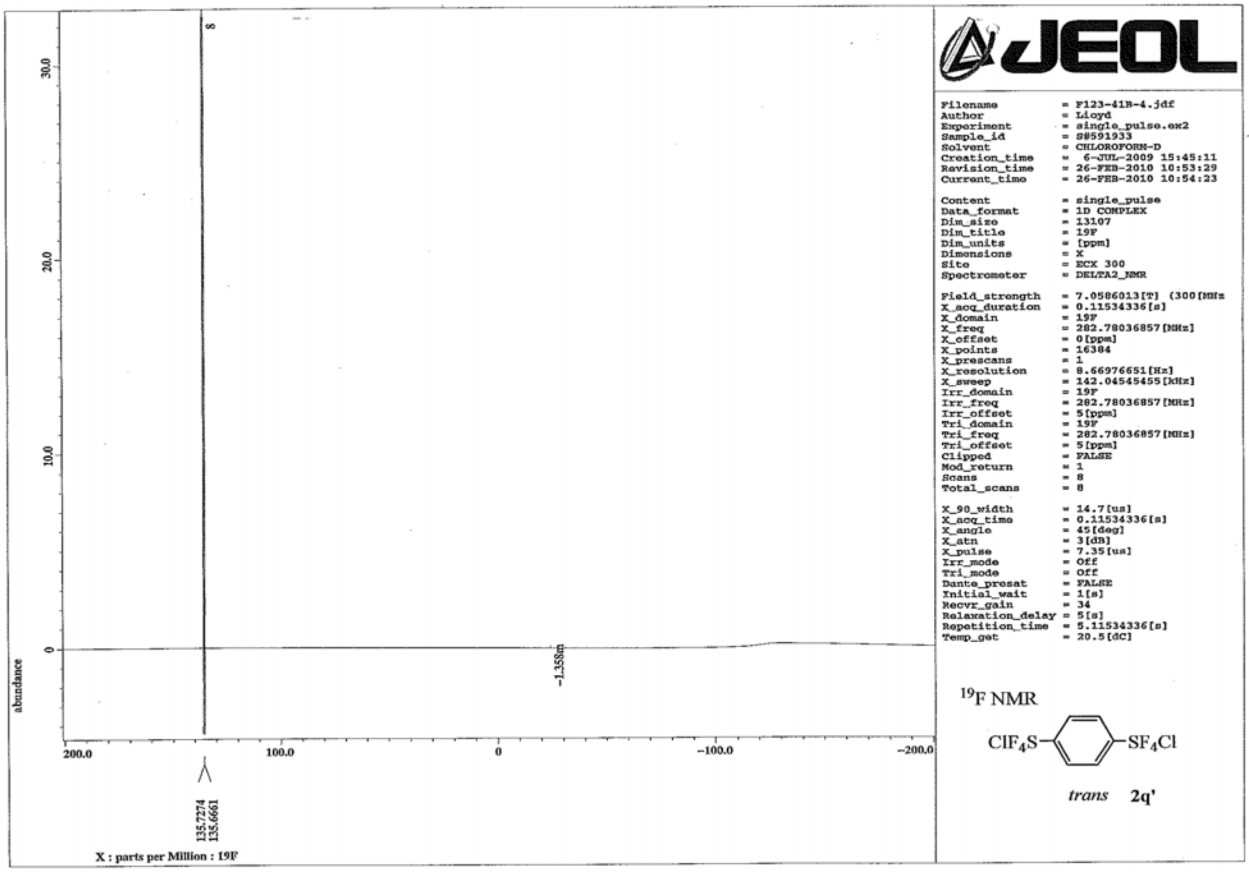
Filename = F123-41B-4.jdf
Author = Lioyd
Experiment = single_pulse.ex2
Sample_id = S#591933
Solvent = CHLOROFORM-D
Creation_time = 6-JUL-2009 15:45:11
Revision_time = 26-FEB-2010 10:53:29
Current_time = 26-FEB-2010 10:54:23
Content = single_pulse
Data_format = 1D COMPLEX
Dim_size = 13107
Dim_title = 19F
Dim_units = [ppm]
Dimensions = X
Site = ECX 300
Spectrometer = DELTA2_NMR
Field_strength = 7.0586013[T] (300[MHz
X_acq_duration = 0.11534336[s]
X_domain = 19F
X_freq = 282.78036857[MHz]
X_offset = 0[ppm]
X_points = 16384
X_prescans = 1
X_resolution = 8.66976651[Hz]
X_sweep = 142.04545455[kHz]
Irr_domain = 19F
Irr_freq = 282.78036857[MHz]
Irr_offset = 5[ppm]
Tri_domain = 19F
Tri_freq = 282.78036857[MHz]
Tri_offset = 5[ppm]
Clipped = FALSE
Mod_return = 1
Scans = 8
Total_scans = 8
X_90_width = 14.7[us]
X_acq_time = 0.11534336[s]
X_angle = 45[deg]
X_atn = 3[dB]
X_pulse = 7.35[us]
Irr_mode = Off
Tri_mode = Off
Dante_presat = FALSE
Initial_wait = 1[s]
Recvr_gain = 34
Relaxation_delay = 5[s]
Repetition_time = 5.11534336[s]
Temp_get = 20.5[dC]
19F NMR
ClF4S SF4Cl
trans 2q'
135.7274
135.6661
-1.358
abundance
X : parts per Million : 19F

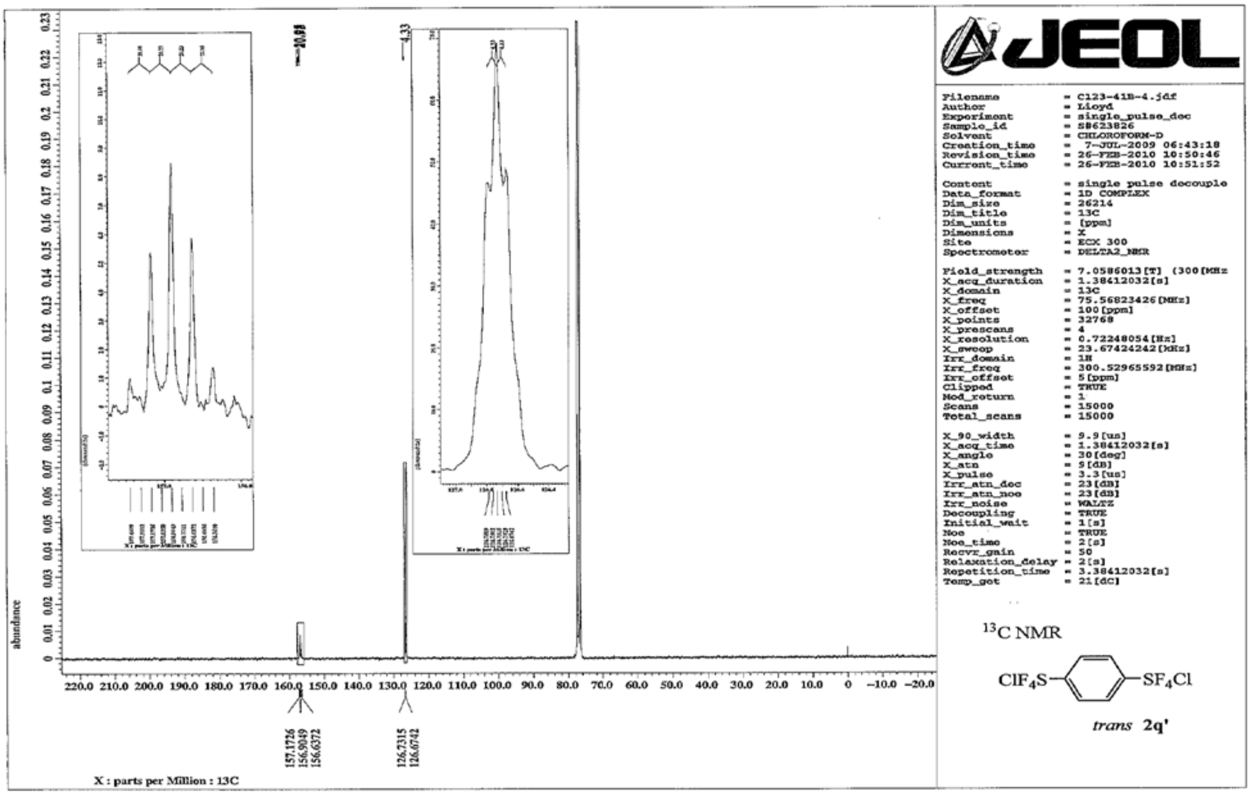
Filename = C123-41B-4.jdf
Author = Lioyd
Experiment = single_pulse_dec
Sample_id = S#623826
Solvent = CHLOROFORM-D
Creation_time = 7-JUL-2009 06:43:18
Revision_time = 26-FEB-2010 10:50:46
Current_time = 26-FEB-2010 10:51:52
Content = single pulse decouple
Data_format = 1D COMPLEX
Dim_size = 26214
Dim_title = 13C
Dim_units = [ppm]
Dimensions = X
Site = ECX 300
Spectrometer = DELTA2_NMR
Field_strength = 7.0586013[T] (300[MHz
X_acq_duration = 1.38412032[s]
X_domain = 13C
X_freq = 75.56823426[MHz]
X_offset = 100[ppm]
X_points = 32768
X_prescans = 4
X_resolution = 0.72248054[Hz]
X_sweep = 23.67424242[kHz]
Irr_domain = 1H
Irr_freq = 300.52965592[MHz]
Irr_offset = 5[ppm]
Clipped = TRUE
Mod_return = 1
Scans = 15000
Total_scans = 15000
X_90_width = 9.9[us]
X_acq_time = 1.38412032[s]
X_angle = 30[deg]
X_atn = 9[dB]
X_pulse = 3.3[us]
Irr_atn_dec = 23[dB]
Irr_atn_noe = 23[dB]
Irr_noise = WALTZ
Decoupling = TRUE
Initial_wait = 1[s]
Noe = TRUE
Noe_time = 2[s]
Recvr_gain = 50
Relaxation_delay = 2[s]
Repetition_time = 3.38412032[s]
Temp_get = 21[dC]
13C NMR
ClF4S SF4Cl
trans 2q'
157.1726
156.9049
156.6372
126.7315
126.6742
abundance
X : parts per Million : 13C

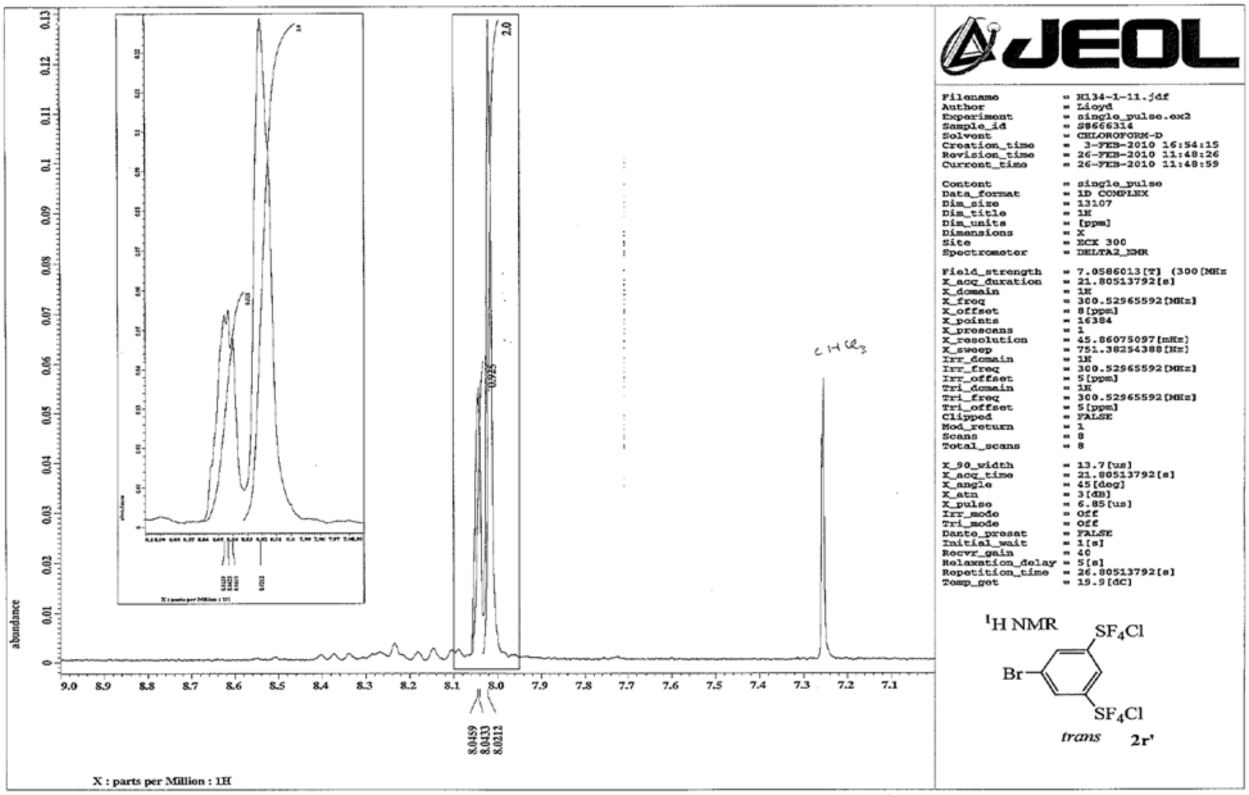
JEOL
Filename = H134-1-11.jdf
Author = Lioyd
Solvent = CHLOROFORM-D
^{1}H NMR
SF4Cl
Br
SF4Cl
trans 2r'
8.0459
8.0433
8.0212
CHCl3
abundance
X : parts per Million : 1H

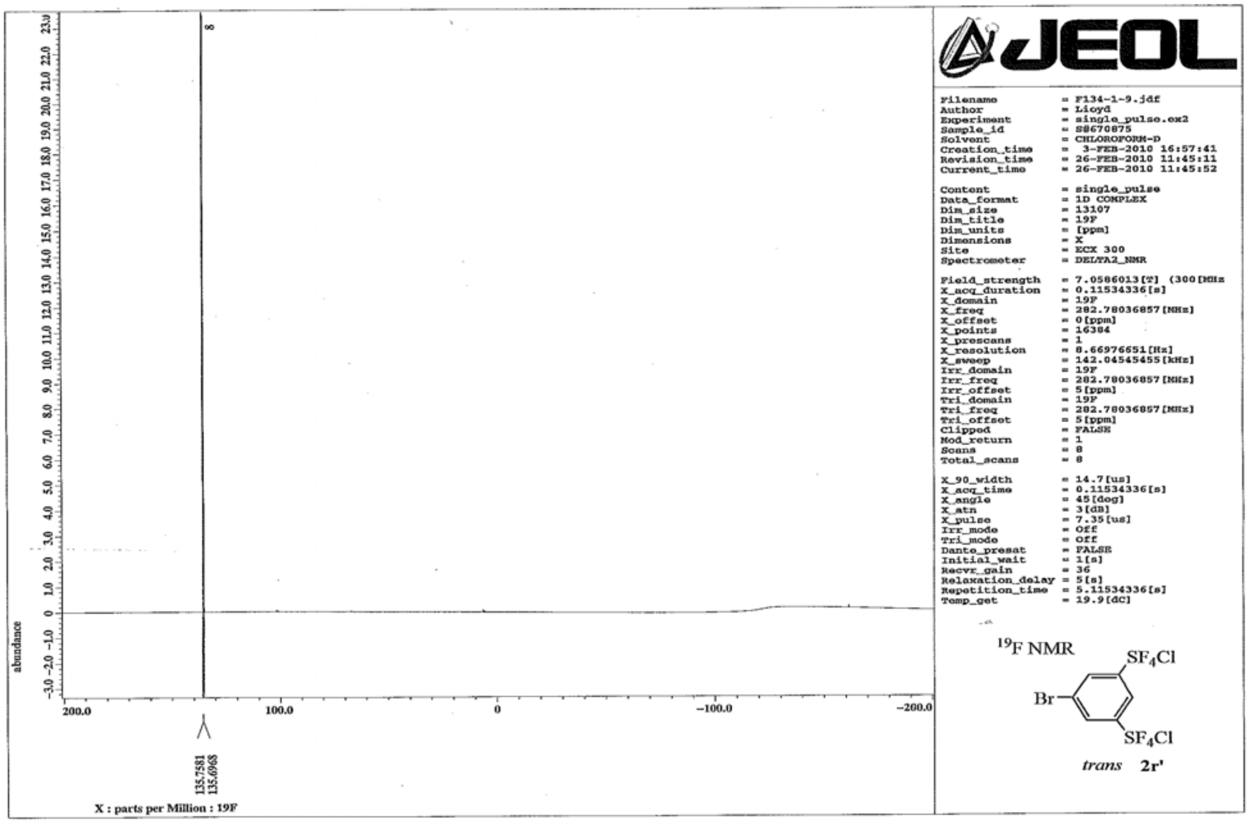
JEOL
Filename = F134-1-9.jdf
Author = Lioyd
Solvent = CHLOROFORM-D
^{19}F NMR
SF4Cl
Br
SF4Cl
trans 2r'
135.7581
135.6968
abundance
X : parts per Million : 19F

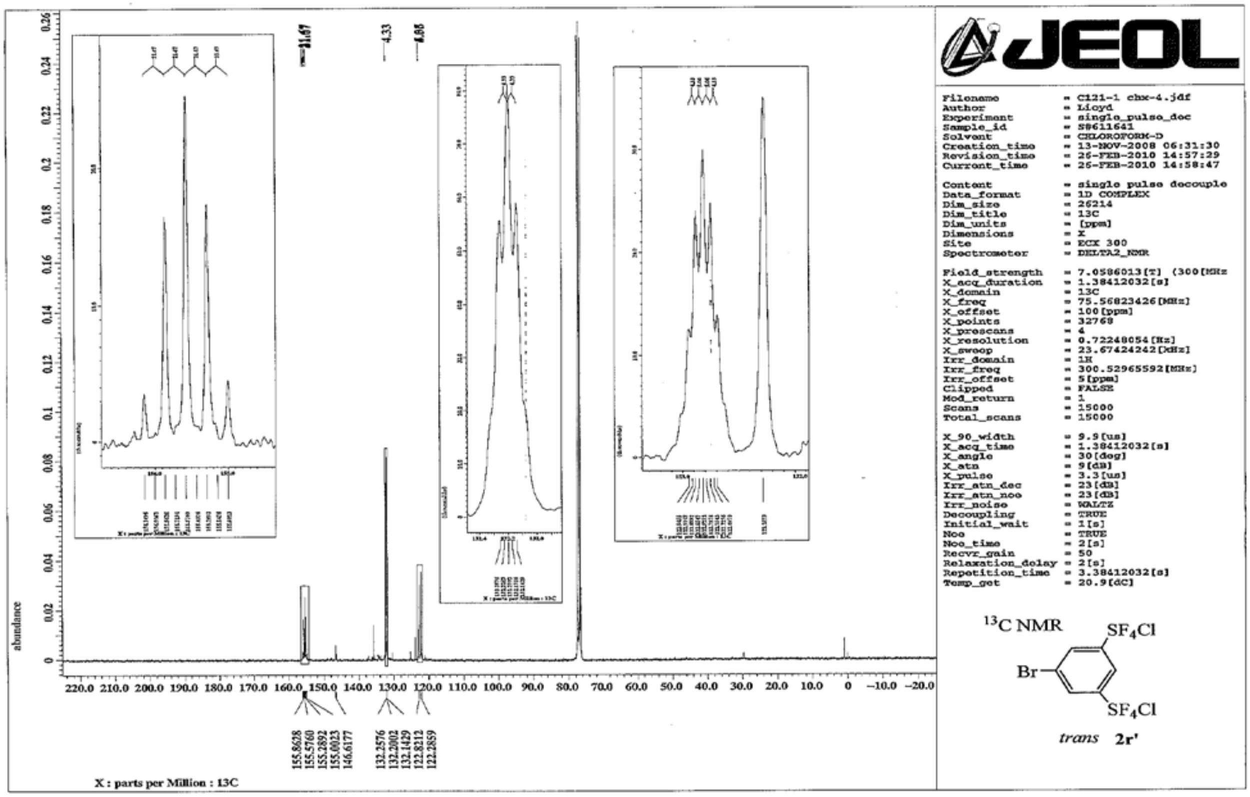

JEOL
Filename = C121-1 cbx-4.jdf
Author = Lloyd
Experiment = single_pulse_dec
Sample_id = S8611641
Solvent = CHLOROFORM-D
Creation_time = 13-NOV-2008 06:31:30
Revision_time = 26-FEB-2010 14:57:29
Current_time = 25-FEB-2010 14:58:47
Content = single pulse decouple
Data_format = 1D COMPLEX
Dim_size = 26214
Dim_title = 13C
Dim_units = [ppm]
Dimensions = X
Site = ECX 300
Spectrometer = DELTA2_NMR
Field_strength = 7.0586013[T] (300[MHz
X_acq_duration = 1.38412032[s]
X_domain = 13C
X_freq = 75.56823426[MHz]
X_offset = 100[ppm]
X_points = 32768
X_prescans = 4
X_resolution = 0.72248054[Hz]
X_sweep = 23.67424242[kHz]
Irr_domain = 1H
Irr_freq = 300.52965592[MHz]
Irr_offset = 5[ppm]
Clipped = FALSE
Mod_return = 1
Scans = 15000
Total_scans = 15000
X_90_width = 9.9[us]
X_acq_time = 1.38412032[s]
X_angle = 30[deg]
X_atn = 9[dB]
X_pulse = 3.3[us]
Irr_atn_dec = 23[dB]
Irr_atn_noe = 23[dB]
Irr_noise = WALTZ
Decoupling = TRUE
Initial_wait = 1[s]
Noe = TRUE
Noe_time = 2[s]
Recvr_gain = 50
Relaxation_delay = 2[s]
Repetition_time = 3.38412032[s]
Temp_get = 20.9[dC]
13C NMR
SF4Cl
Br
SF4Cl
trans 2r'
abundance
X : parts per Million : 13C
155.8628
155.5760
155.2892
155.0023
146.6177
132.2576
132.2002
132.1429
122.8212
122.2859
4.33

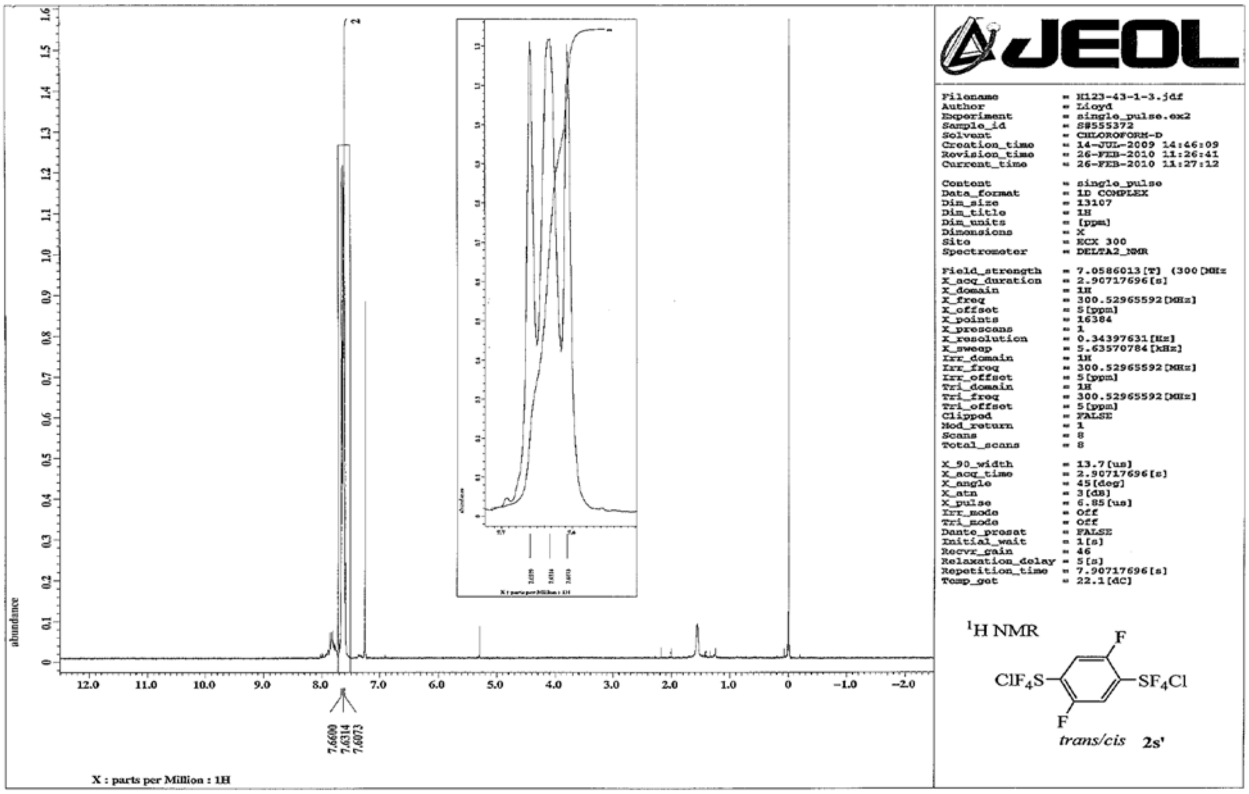

JEOL
Filename = H123-43-1-3.jdf
Author = Lloyd
Experiment = single_pulse.ex2
Sample_id = S8555372
Solvent = CHLOROFORM-D
Creation_time = 14-JUL-2009 14:46:09
Revision_time = 26-FEB-2010 11:26:41
Current_time = 26-FEB-2010 11:27:12
Content = single_pulse
Data_format = 1D COMPLEX
Dim_size = 13107
Dim_title = 1H
Dim_units = [ppm]
Dimensions = X
Site = ECX 300
Spectrometer = DELTA2_NMR
Field_strength = 7.0586013[T] (300[MHz
X_acq_duration = 2.90717696[s]
X_domain = 1H
X_freq = 300.52965592[MHz]
X_offset = 5[ppm]
X_points = 16384
X_prescans = 1
X_resolution = 0.34397631[Hz]
X_sweep = 5.63570784[kHz]
Irr_domain = 1H
Irr_freq = 300.52965592[MHz]
Irr_offset = 5[ppm]
Tri_domain = 1H
Tri_freq = 300.52965592[MHz]
Tri_offset = 5[ppm]
Clipped = FALSE
Mod_return = 1
Scans = 8
Total_scans = 8
X_90_width = 13.7[us]
X_acq_time = 2.90717696[s]
X_angle = 45[deg]
X_atn = 3[dB]
X_pulse = 6.85[us]
Irr_mode = Off
Tri_mode = Off
Dante_presat = FALSE
Initial_wait = 1[s]
Recvr_gain = 46
Relaxation_delay = 5[s]
Repetition_time = 7.90717696[s]
Temp_get = 22.1[dC]
1H NMR
F
ClF4S
SF4Cl
F
trans/cis 2s'
abundance
X : parts per Million : 1H
7.6600
7.6314
7.6073

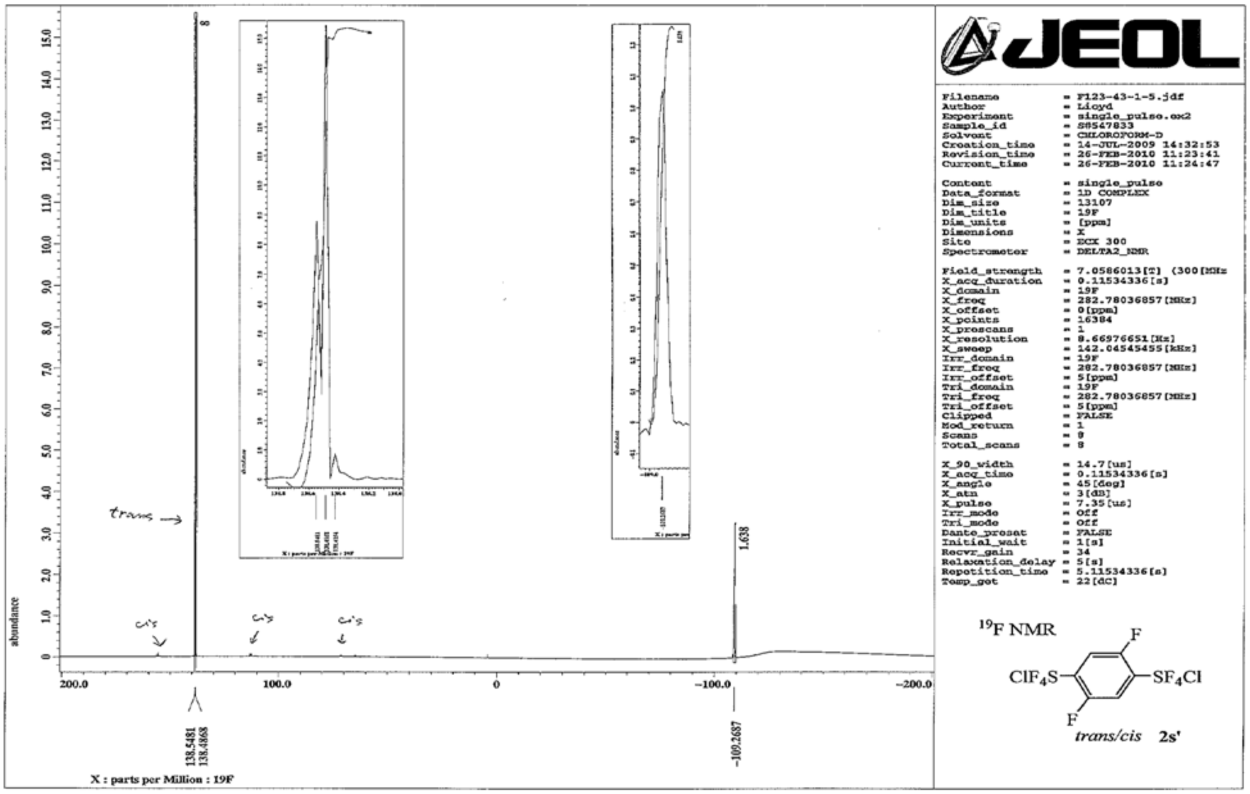
JEOL
Filename = F123-43-1-5.jdf
Author = Lloyd
Experiment = single_pulse.ex2
Sample_id = S0547833
Solvent = CHLOROFORM-D
Creation_time = 14-JUL-2009 14:32:53
Revision_time = 26-FEB-2010 11:23:41
Current_time = 26-FEB-2010 11:24:47
Content = single_pulse
Data_format = 1D COMPLEX
Dim_size = 13107
Dim_title = 19F
Dim_units = [ppm]
Dimensions = X
Site = ECX 300
Spectrometer = DELTA2_NMR
Field_strength = 7.0586013[T] (300[MHz
X_acq_duration = 0.11534336[s]
X_domain = 19F
X_freq = 282.78036857[MHz]
X_offset = 0[ppm]
X_points = 16384
X_prescans = 1
X_resolution = 8.66976651[Hz]
X_sweep = 142.04545455[kHz]
Irr_domain = 19F
Irr_freq = 282.78036857[MHz]
Irr_offset = 5[ppm]
Tri_domain = 19F
Tri_freq = 282.78036857[MHz]
Tri_offset = 5[ppm]
Clipped = FALSE
Mod_return = 1
Scans = 8
Total_scans = 8
X_90_width = 14.7[us]
X_acq_time = 0.11534336[s]
X_angle = 45[deg]
X_atn = 3[dB]
X_pulse = 7.35[us]
Irr_mode = Off
Tri_mode = Off
Dante_presat = FALSE
Initial_wait = 1[s]
Recvr_gain = 34
Relaxation_delay = 5[s]
Repetition_time = 5.11534336[s]
Temp_get = 22[dC]
19F NMR
ClF4S
SF4Cl
F
F
trans/cis 2s'
trans
cis
cis
cis
1.638
138.5481
138.4868
-109.2687
abundance
200.0
100.0
0
-100.0
-200.0
X : parts per Million : 19F

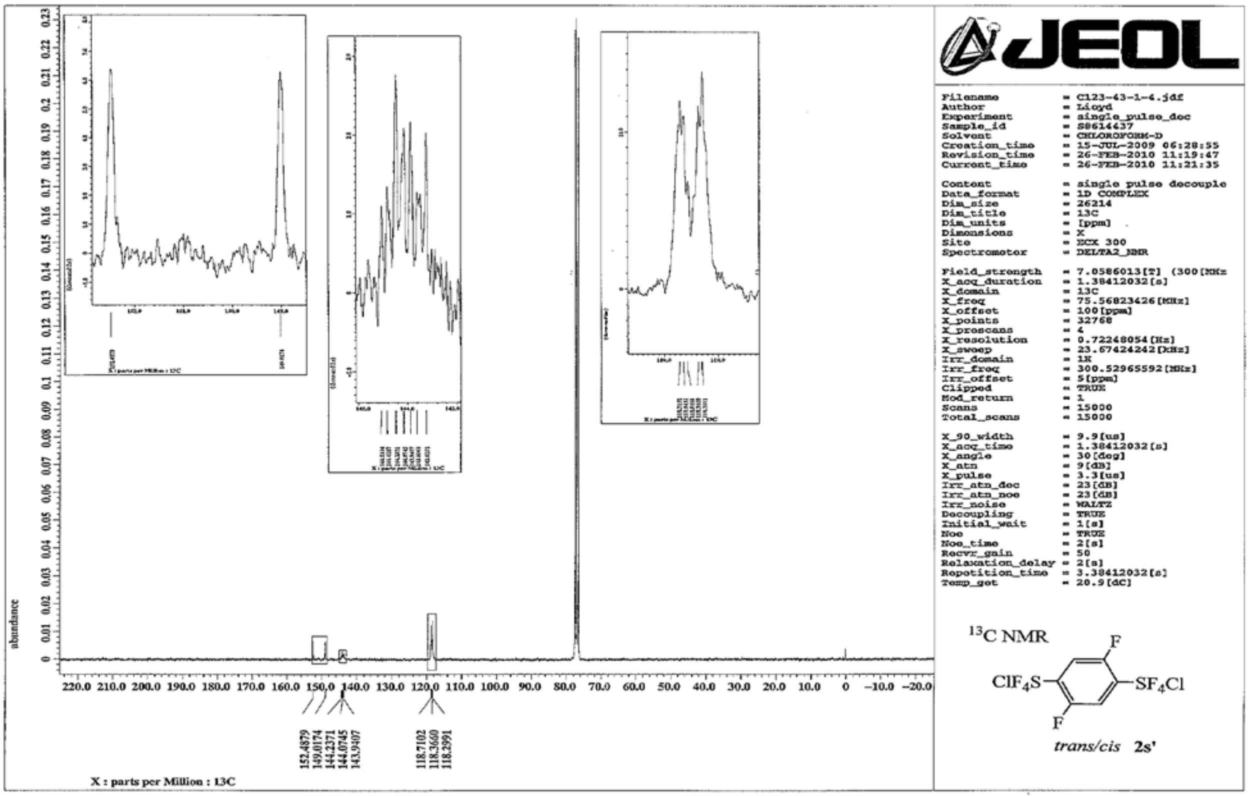
JEOL
Filename = C123-43-1-4.jdf
Author = Lloyd
Experiment = single_pulse_dec
Sample_id = S0614437
Solvent = CHLOROFORM-D
Creation_time = 15-JUL-2009 06:28:55
Revision_time = 26-FEB-2010 11:19:47
Current_time = 26-FEB-2010 11:21:35
Content = single pulse decouple
Data_format = 1D COMPLEX
Dim_size = 26214
Dim_title = 13C
Dim_units = [ppm]
Dimensions = X
Site = ECX 300
Spectrometer = DELTA2_NMR
Field_strength = 7.0586013[T] (300[MHz
X_acq_duration = 1.38412032[s]
X_domain = 13C
X_freq = 75.56823426[MHz]
X_offset = 100[ppm]
X_points = 32768
X_prescans = 4
X_resolution = 0.72248054[Hz]
X_sweep = 23.67424242[kHz]
Irr_domain = 1H
Irr_freq = 300.52965592[MHz]
Irr_offset = 5[ppm]
Clipped = TRUE
Mod_return = 1
Scans = 15000
Total_scans = 15000
X_90_width = 9.9[us]
X_acq_time = 1.38412032[s]
X_angle = 30[deg]
X_atn = 9[dB]
X_pulse = 3.3[us]
Irr_atn_dec = 23[dB]
Irr_atn_noe = 23[dB]
Irr_noise = WALTZ
Decoupling = TRUE
Initial_wait = 1[s]
Noe = TRUE
Noe_time = 2[s]
Recvr_gain = 50
Relaxation_delay = 2[s]
Repetition_time = 3.38412032[s]
Temp_get = 20.9[dC]
13C NMR
ClF4S
SF4Cl
F
F
trans/cis 2s'
152.4879
149.0174
144.2371
144.0745
143.9407
118.7102
118.3660
118.2991
abundance
X : parts per Million : 13C

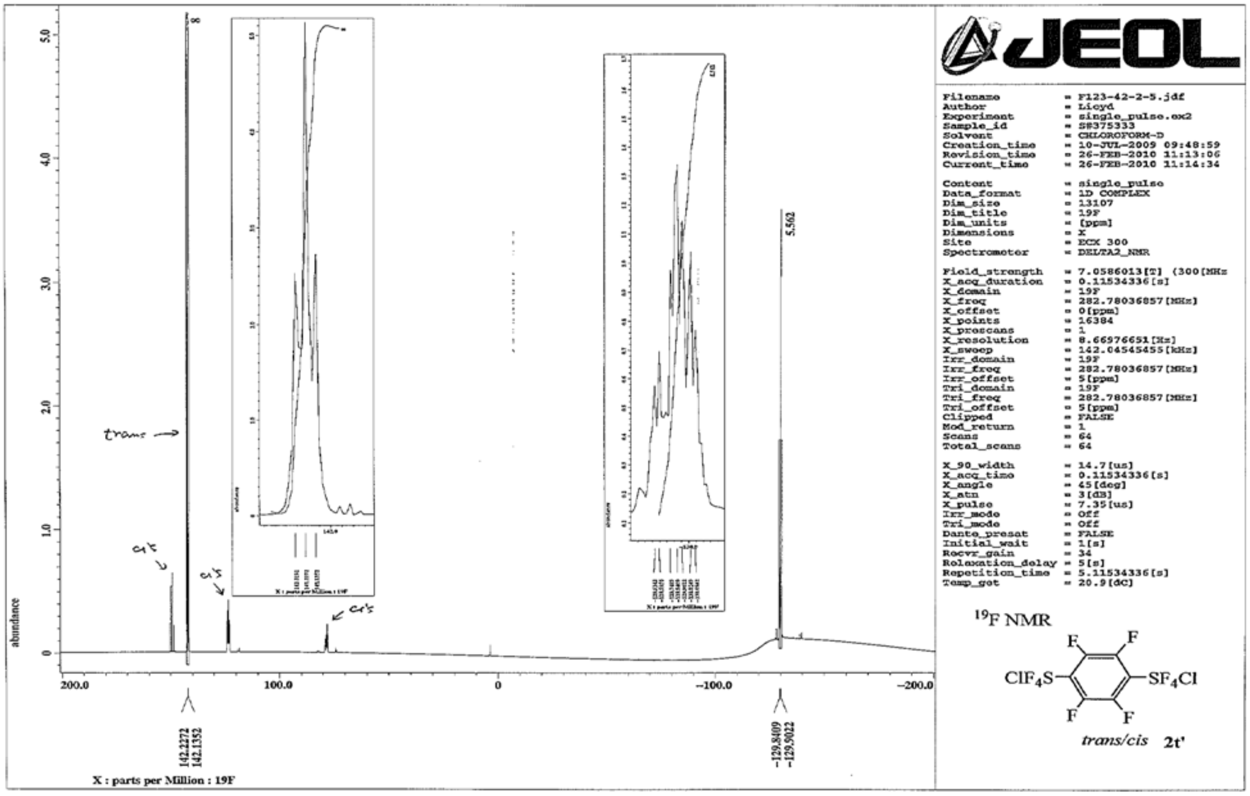
19F NMR
ClF4S
SF4Cl
F
trans/cis 2t'
X : parts per Million : 19F
142.2272
142.1352
-129.8409
-129.9022
5.562
trans
cis

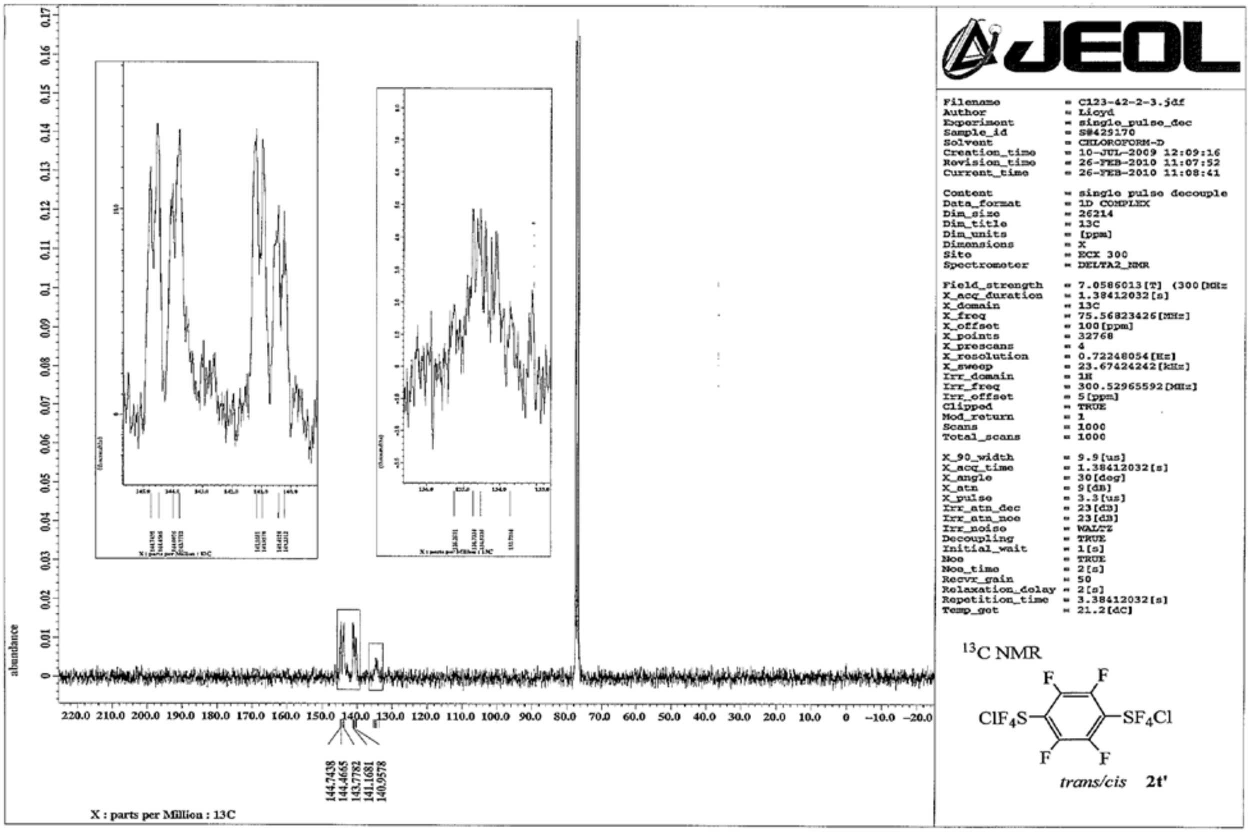
13C NMR
ClF4S
SF4Cl
F
trans/cis 2t'
X : parts per Million : 13C
144.7438
144.4665
143.7782
141.1681
140.9578

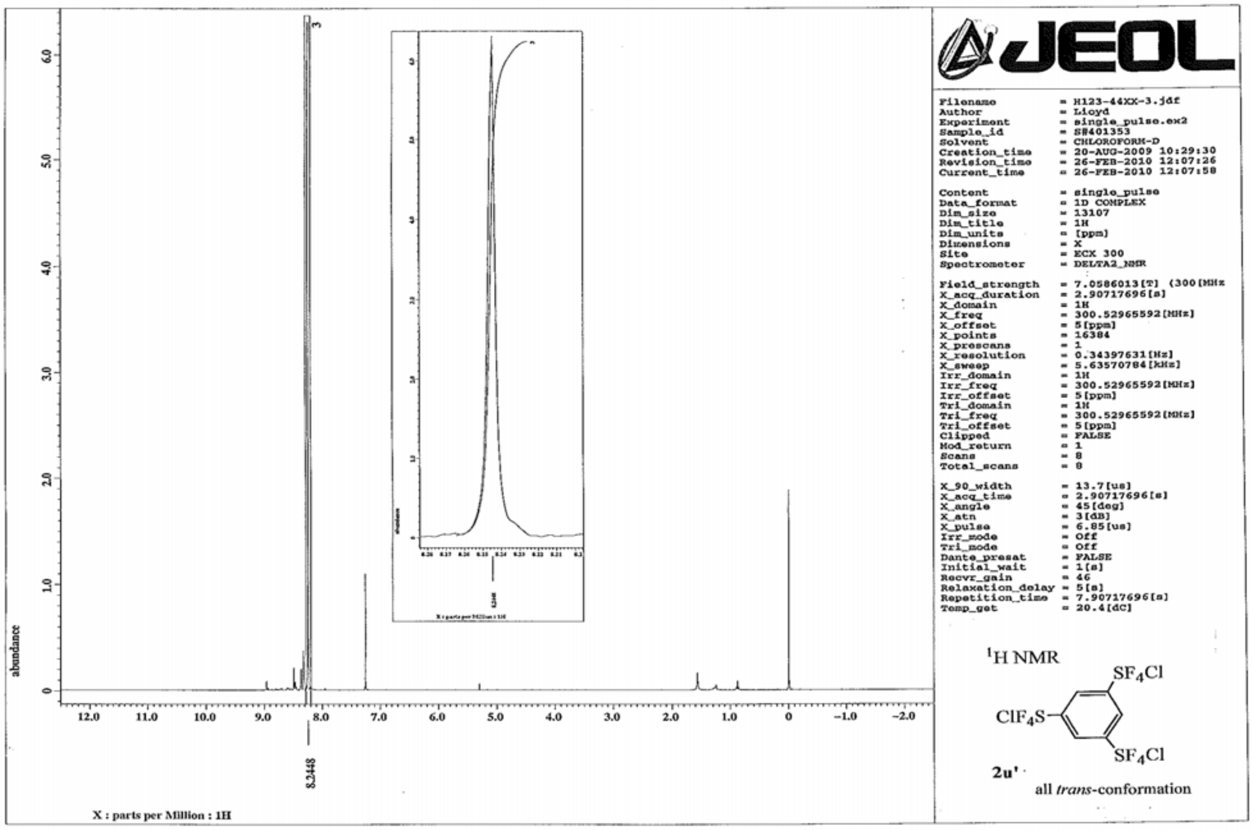
JEOL
Filename = H123-44XX-3.jdf
Author = Lioyd
Experiment = single_pulse.ex2
Sample_id = S#401353
Solvent = CHLOROFORM-D
Creation_time = 20-AUG-2009 10:29:30
Revision_time = 26-FEB-2010 12:07:26
Current_time = 26-FEB-2010 12:07:58
Content = single_pulse
Data_format = 1D COMPLEX
Dim_size = 13107
Dim_title = 1H
Dim_units = [ppm]
Dimensions = X
Site = ECX 300
Spectrometer = DELTA2_NMR
Field_strength = 7.0586013[T] (300[MHz
X_acq_duration = 2.90717696[s]
X_domain = 1H
X_freq = 300.52965592[MHz]
X_offset = 5[ppm]
X_points = 16384
X_prescans = 1
X_resolution = 0.34397631[Hz]
X_sweep = 5.63570784[kHz]
Irr_domain = 1H
Irr_freq = 300.52965592[MHz]
Irr_offset = 5[ppm]
Tri_domain = 1H
Tri_freq = 300.52965592[MHz]
Tri_offset = 5[ppm]
Clipped = FALSE
Mod_return = 1
Scans = 8
Total_scans = 8
X_90_width = 13.7[us]
X_acq_time = 2.90717696[s]
X_angle = 45[deg]
X_atn = 3[dB]
X_pulse = 6.85[us]
Irr_mode = Off
Tri_mode = Off
Dante_presat = FALSE
Initial_wait = 1[s]
Recvr_gain = 46
Relaxation_delay = 5[s]
Repetition_time = 7.90717696[s]
Temp_get = 20.4[dC]
1H NMR
SF4Cl
ClF4S
SF4Cl
2u'
all trans-conformation
abundance
8.2448
X : parts per Million : 1H

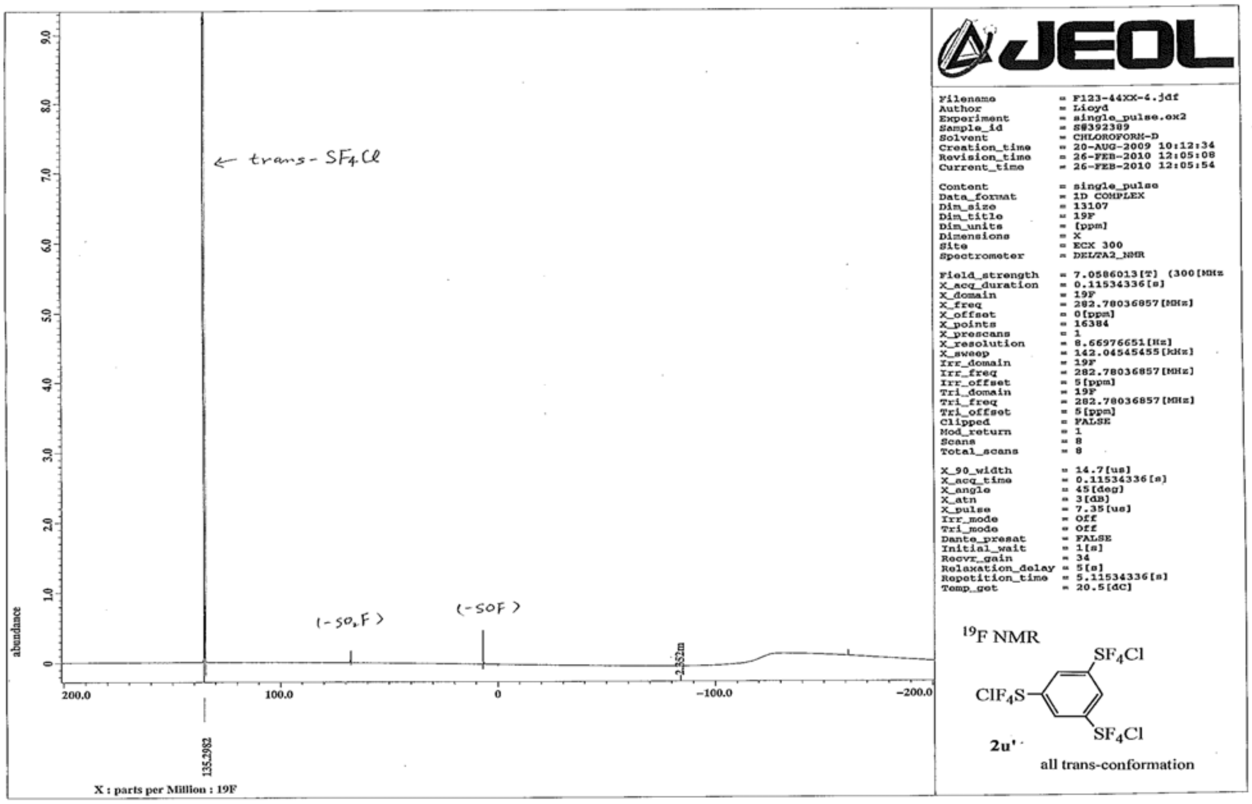
JEOL
Filename = F123-44XX-4.jdf
Author = Lioyd
Experiment = single_pulse.ex2
Sample_id = S#392389
Solvent = CHLOROFORM-D
Creation_time = 20-AUG-2009 10:12:34
Revision_time = 26-FEB-2010 12:05:08
Current_time = 26-FEB-2010 12:05:54
Content = single_pulse
Data_format = 1D COMPLEX
Dim_size = 13107
Dim_title = 19F
Dim_units = [ppm]
Dimensions = X
Site = ECX 300
Spectrometer = DELTA2_NMR
Field_strength = 7.0586013[T] (300[MHz
X_acq_duration = 0.11534336[s]
X_domain = 19F
X_freq = 282.78036857[MHz]
X_offset = 0[ppm]
X_points = 16384
X_prescans = 1
X_resolution = 8.66976651[Hz]
X_sweep = 142.04545455[kHz]
Irr_domain = 19F
Irr_freq = 282.78036857[MHz]
Irr_offset = 5[ppm]
Tri_domain = 19F
Tri_freq = 282.78036857[MHz]
Tri_offset = 5[ppm]
Clipped = FALSE
Mod_return = 1
Scans = 8
Total_scans = 8
X_90_width = 14.7[us]
X_acq_time = 0.11534336[s]
X_angle = 45[deg]
X_atn = 3[dB]
X_pulse = 7.35[us]
Irr_mode = Off
Tri_mode = Off
Dante_presat = FALSE
Initial_wait = 1[s]
Recvr_gain = 34
Relaxation_delay = 5[s]
Repetition_time = 5.11534336[s]
Temp_get = 20.5[dC]
19F NMR
SF4Cl
ClF4S
SF4Cl
2u'
all trans-conformation
← trans-SF4Cl
(-SO2F)
(-SOF)
abundance
135.2982
X : parts per Million : 19F

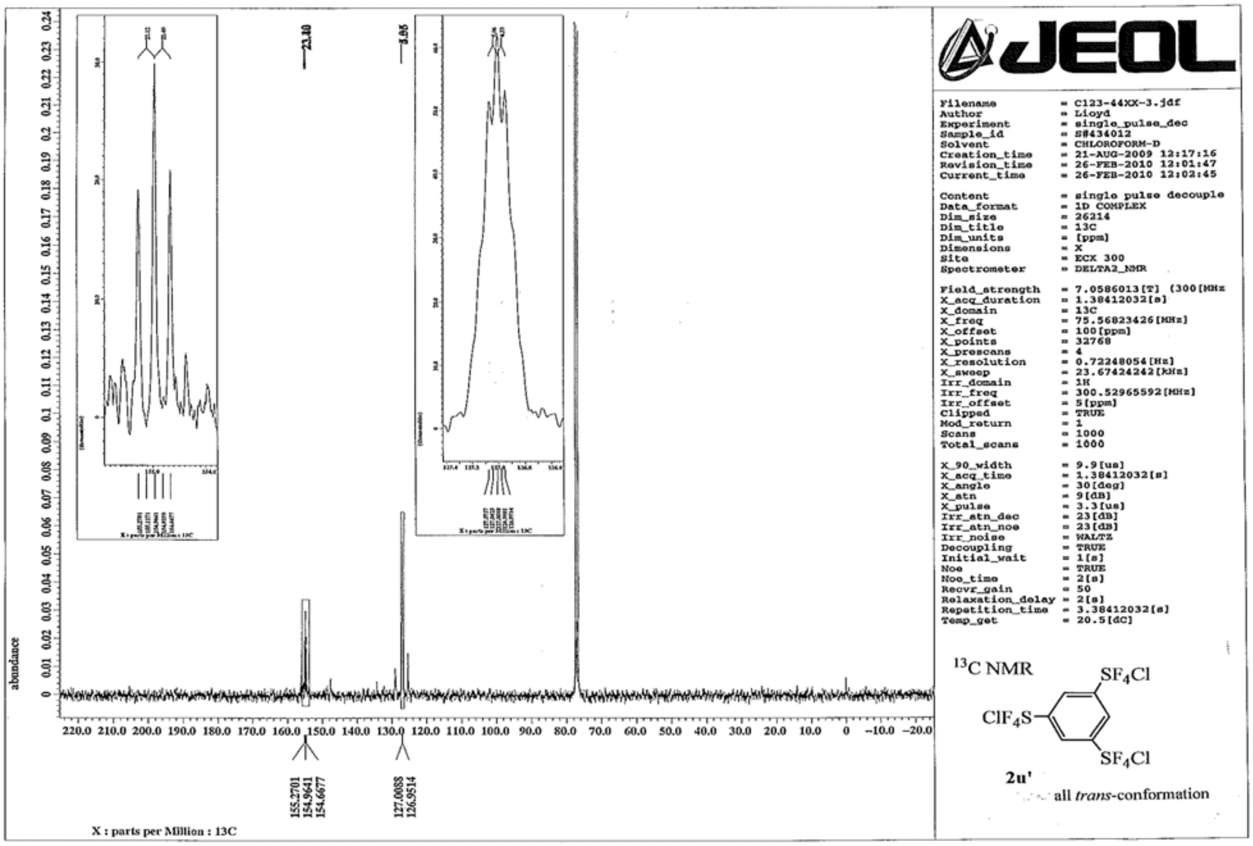
13C NMR
SF4Cl
ClF4S
SF4Cl
2u'
all trans-conformation
X : parts per Million : 13C
abundance

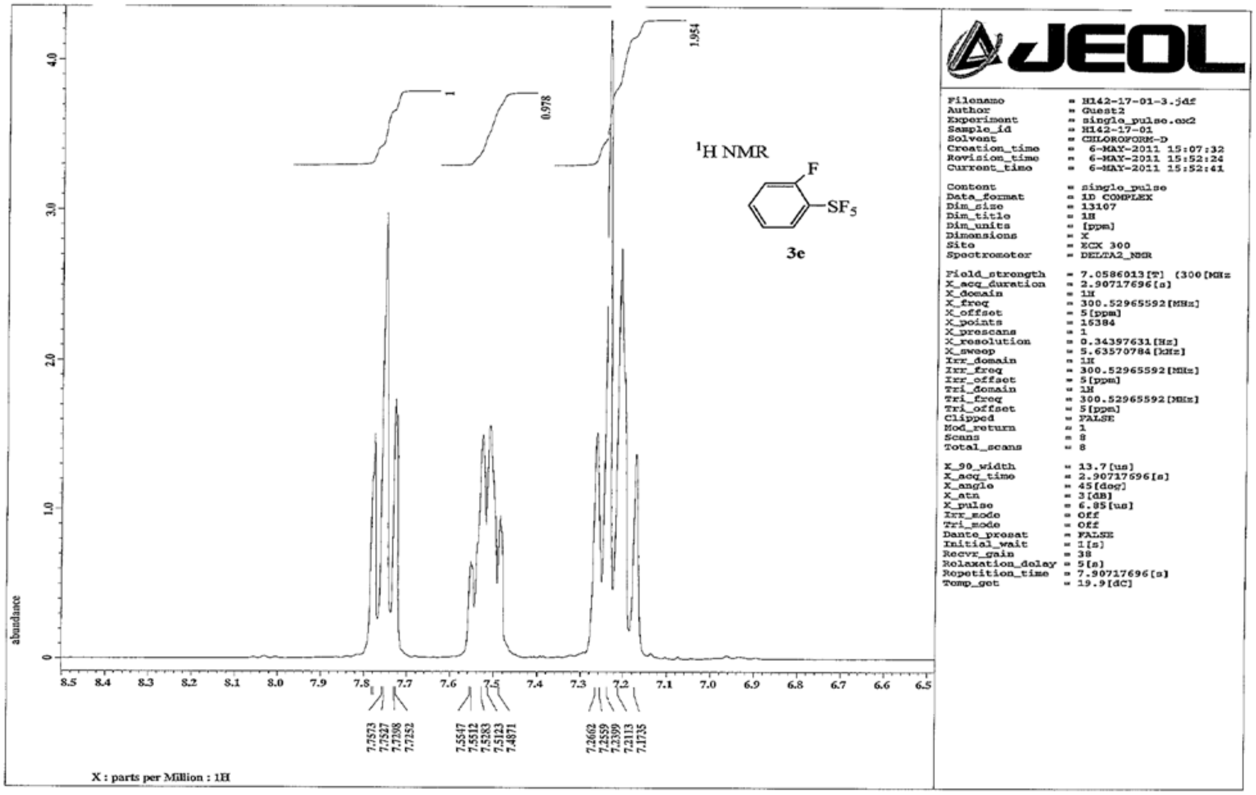
1H NMR
F
SF5
3e
X : parts per Million : 1H
abundance

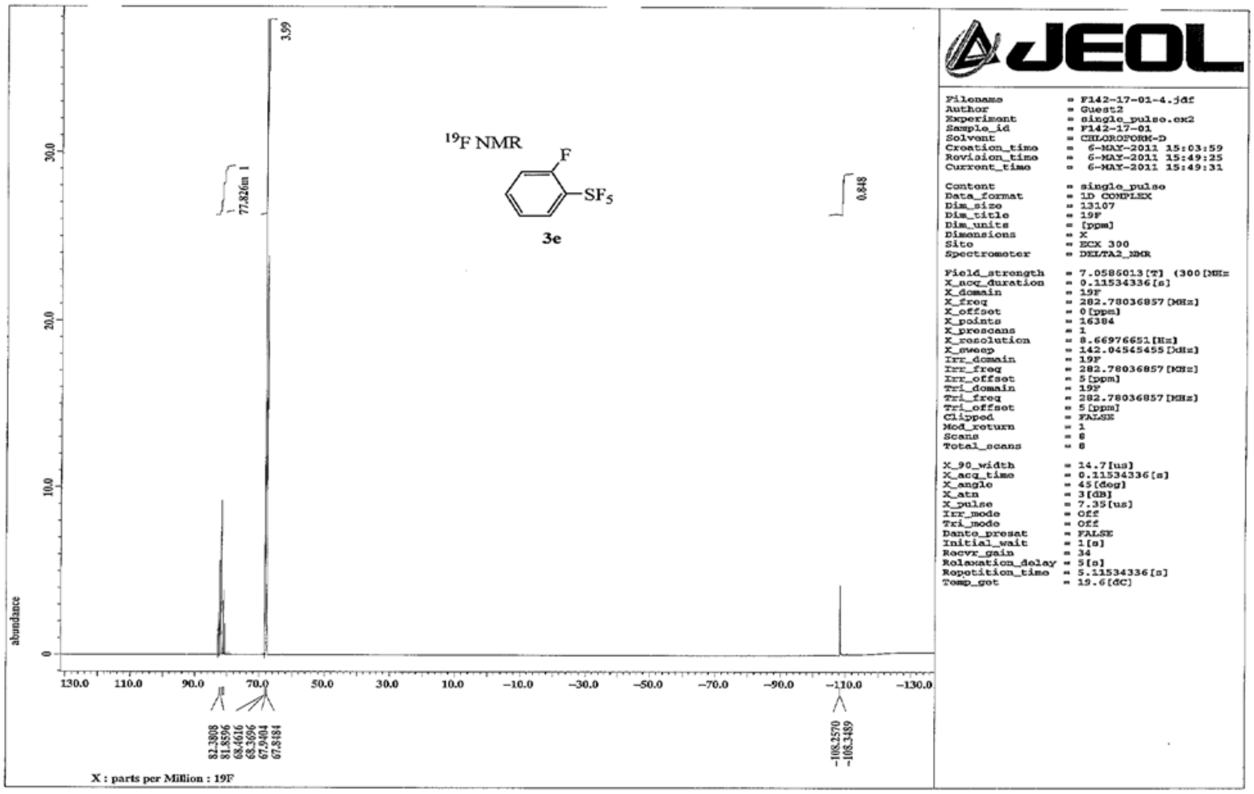
19F NMR
F
SF5
3e
3.99
77.826m 1
0.848
abundance
82.3808
81.8596
68.4616
68.3696
67.9404
67.8484
-108.2570
-108.3489
X : parts per Million : 19F
JEOL

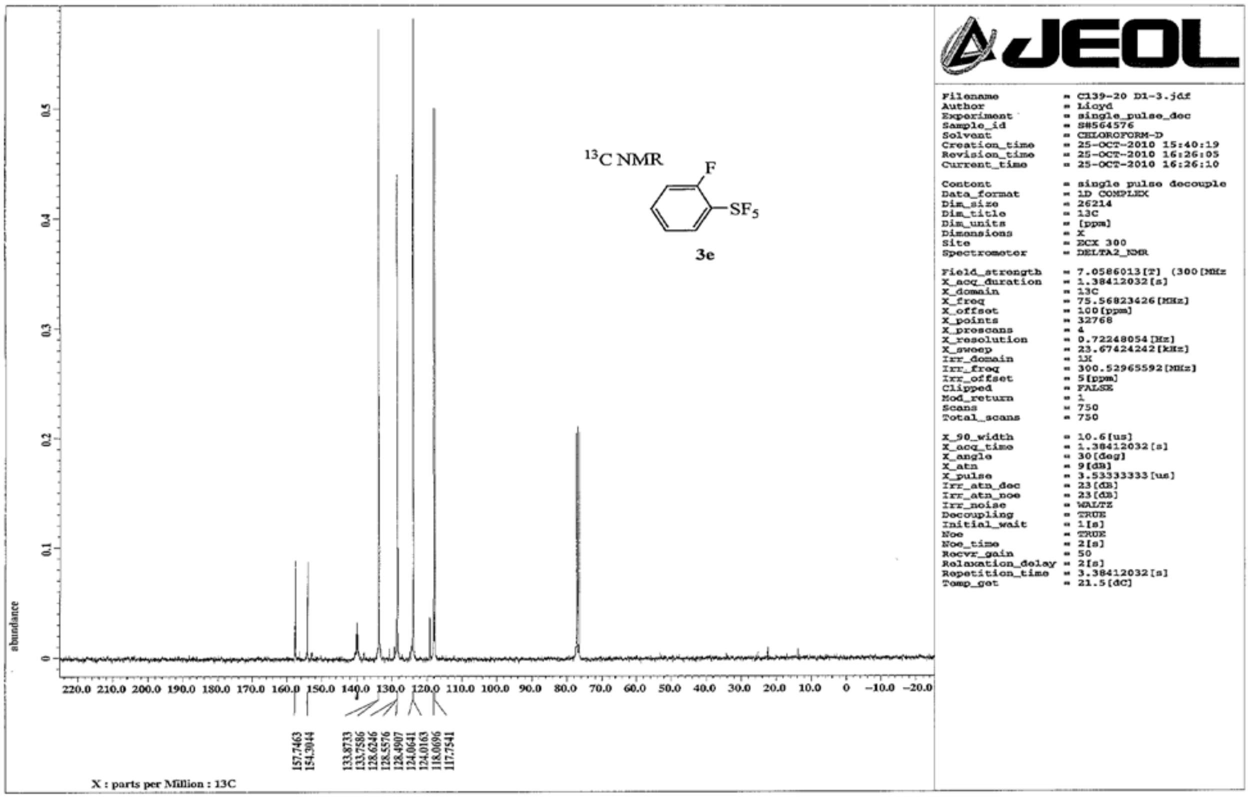
13C NMR
F
SF5
3e
abundance
157.7463
154.3044
133.8733
133.7586
128.6246
128.5576
128.4907
124.0641
124.0163
118.0696
117.7541
X : parts per Million : 13C
JEOL

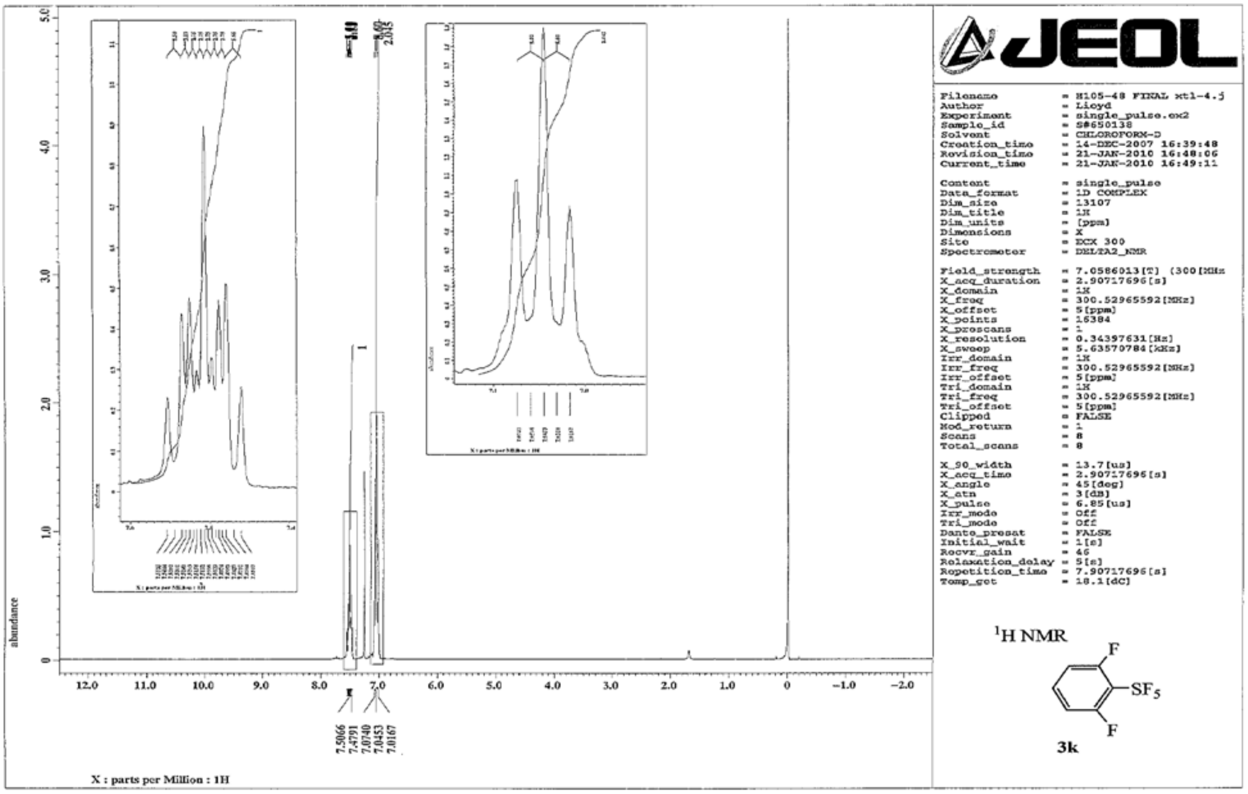
JEOL
Filename = H105-48 FINAL xtl-4.j
Author = Lloyd
Experiment = single_pulse.ex2
Sample_id = S#650138
Solvent = CHLOROFORM-D
Creation_time = 14-DEC-2007 16:39:48
Revision_time = 21-JAN-2010 16:48:06
Current_time = 21-JAN-2010 16:49:11
Content = single_pulse
Data_format = 1D COMPLEX
Dim_size = 13107
Dim_title = 1H
Dim_units = [ppm]
Dimensions = X
Site = ECX 300
Spectrometer = DELTA2_NMR
Field_strength = 7.0586013[T] (300[MHz
X_acq_duration = 2.90717696[s]
X_domain = 1H
X_freq = 300.52965592[MHz]
X_offset = 5[ppm]
X_points = 16384
X_prescans = 1
X_resolution = 0.34397631[Hz]
X_sweep = 5.63570784[kHz]
Irr_domain = 1H
Irr_freq = 300.52965592[MHz]
Irr_offset = 5[ppm]
Tri_domain = 1H
Tri_freq = 300.52965592[MHz]
Tri_offset = 5[ppm]
Clipped = FALSE
Mod_return = 1
Scans = 8
Total_scans = 8
X_90_width = 13.7[us]
X_acq_time = 2.90717696[s]
X_angle = 45[deg]
X_atn = 3[dB]
X_pulse = 6.85[us]
Irr_mode = Off
Tri_mode = Off
Dante_presat = FALSE
Initial_wait = 1[s]
Recvr_gain = 46
Relaxation_delay = 5[s]
Repetition_time = 7.90717696[s]
Temp_get = 18.1[dC]
1H NMR
F
SF5
F
3k
abundance
2.045
1
7.5066
7.4791
7.0740
7.0453
7.0167
X : parts per Million : 1H

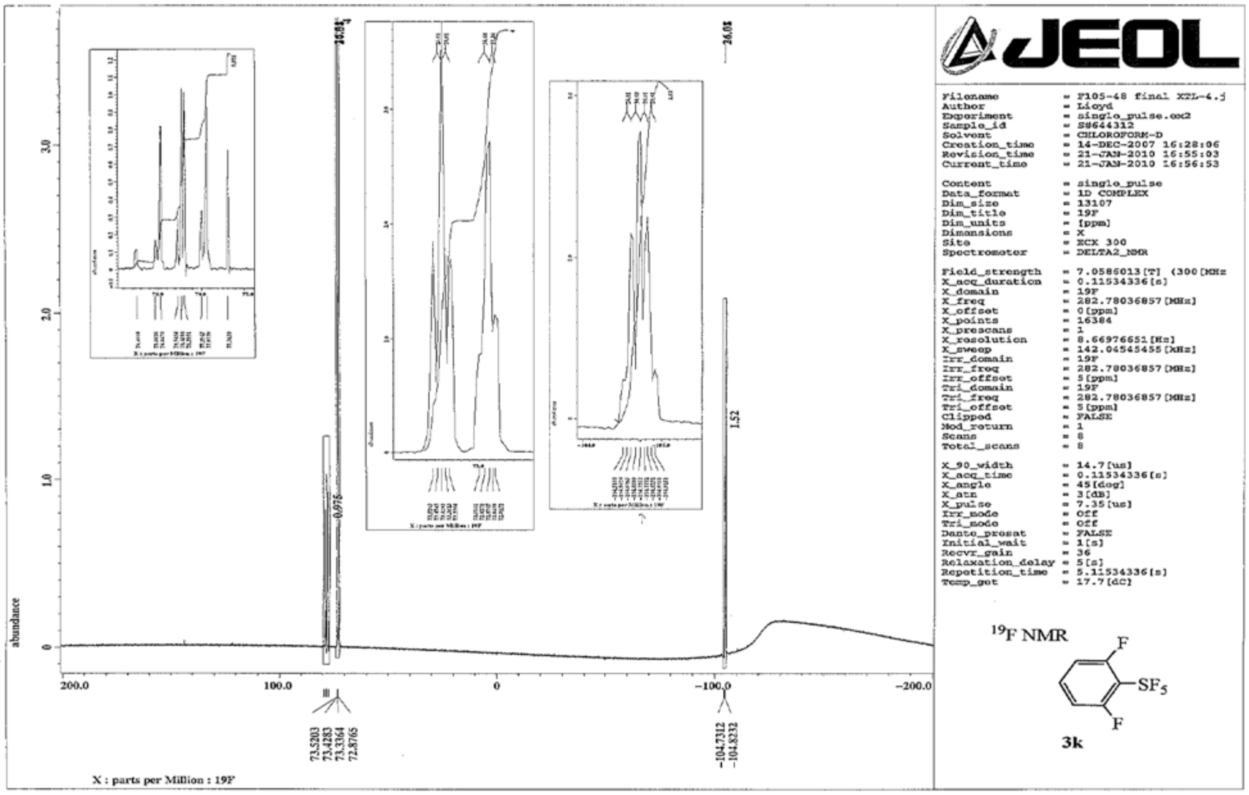
JEOL
Filename = F105-48 final XTL-4.j
Author = Lloyd
Experiment = single_pulse.ex2
Sample_id = S#644312
Solvent = CHLOROFORM-D
Creation_time = 14-DEC-2007 16:28:06
Revision_time = 21-JAN-2010 16:55:03
Current_time = 21-JAN-2010 16:56:53
Content = single_pulse
Data_format = 1D COMPLEX
Dim_size = 13107
Dim_title = 19F
Dim_units = [ppm]
Dimensions = X
Site = ECX 300
Spectrometer = DELTA2_NMR
Field_strength = 7.0586013[T] (300[MHz
X_acq_duration = 0.11534336[s]
X_domain = 19F
X_freq = 282.78036857[MHz]
X_offset = 0[ppm]
X_points = 16384
X_prescans = 1
X_resolution = 8.66976651[Hz]
X_sweep = 142.04545455[kHz]
Irr_domain = 19F
Irr_freq = 282.78036857[MHz]
Irr_offset = 5[ppm]
Tri_domain = 19F
Tri_freq = 282.78036857[MHz]
Tri_offset = 5[ppm]
Clipped = FALSE
Mod_return = 1
Scans = 8
Total_scans = 8
X_90_width = 14.7[us]
X_acq_time = 0.11534336[s]
X_angle = 45[deg]
X_atn = 3[dB]
X_pulse = 7.35[us]
Irr_mode = Off
Tri_mode = Off
Dante_presat = FALSE
Initial_wait = 1[s]
Recvr_gain = 36
Relaxation_delay = 5[s]
Repetition_time = 5.11534336[s]
Temp_get = 17.7[dC]
19F NMR
F
SF5
F
3k
abundance
0.978
1.52
73.5203
73.4283
73.3364
72.8765
-104.7312
-104.8232
X : parts per Million : 19F

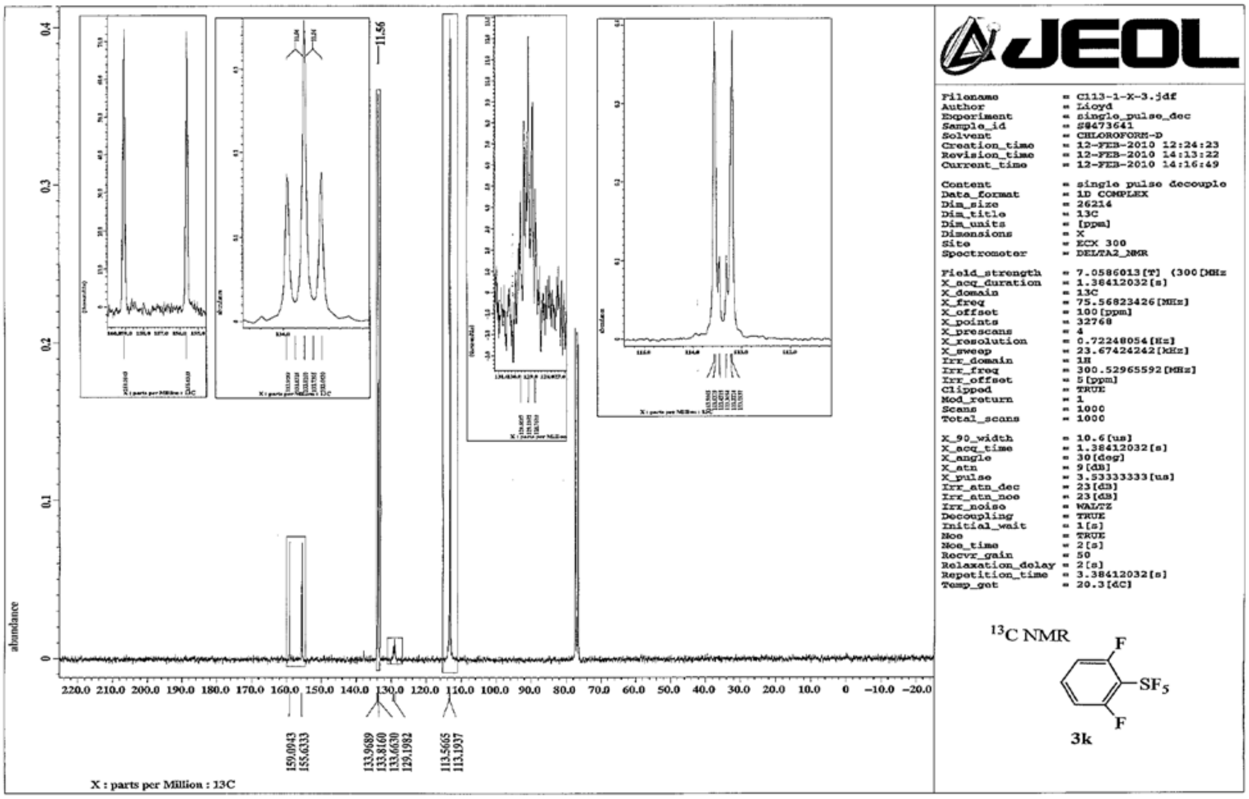
JEOL
13C NMR
3k
X : parts per Million : 13C
abundance
159.0943
155.6333
133.9689
133.8160
133.6630
129.1982
113.5665
113.1937

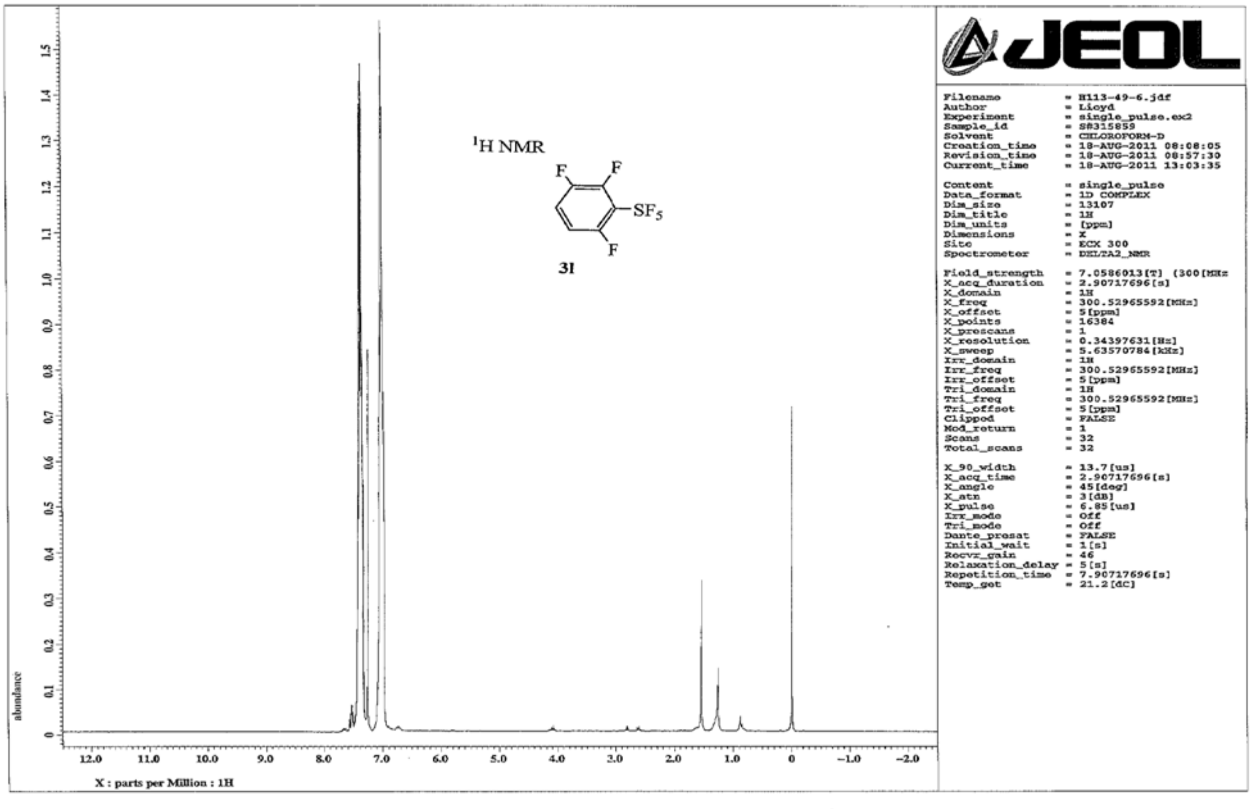
JEOL
1H NMR
3l
X : parts per Million : 1H
abundance

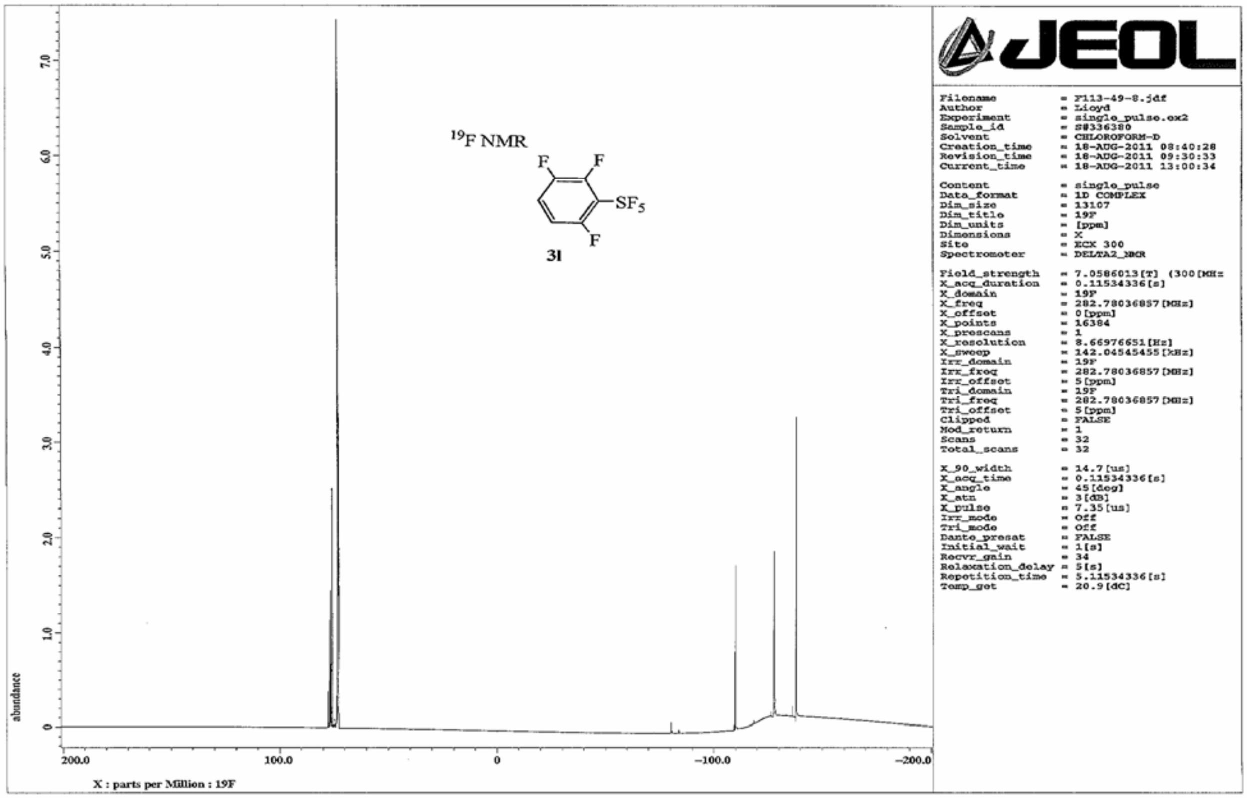
19F NMR
F
F
SF5
F
31
Filename = F113-49-8.jdf
Author = Lioyd
Experiment = single_pulse.ex2
Sample_id = S#336380
Solvent = CHLOROFORM-D
Creation_time = 18-AUG-2011 08:40:28
Revision_time = 18-AUG-2011 09:30:33
Current_time = 18-AUG-2011 13:00:34
Content = single_pulse
Data_format = 1D COMPLEX
Dim_size = 13107
Dim_title = 19F
Dim_units = [ppm]
Dimensions = X
Site = ECX 300
Spectrometer = DELTA2_NMR
Field_strength = 7.0586013[T] (300[MHz
X_acq_duration = 0.11534336[s]
X_domain = 19F
X_freq = 282.78036857[MHz]
X_offset = 0[ppm]
X_points = 16384
X_prescans = 1
X_resolution = 8.66976651[Hz]
X_sweep = 142.04545455[kHz]
Irr_domain = 19F
Irr_freq = 282.78036857[MHz]
Irr_offset = 5[ppm]
Tri_domain = 19F
Tri_freq = 282.78036857[MHz]
Tri_offset = 5[ppm]
Clipped = FALSE
Mod_return = 1
Scans = 32
Total_scans = 32
X_90_width = 14.7[us]
X_acq_time = 0.11534336[s]
X_angle = 45[deg]
X_atn = 3[dB]
X_pulse = 7.35[us]
Irr_mode = Off
Tri_mode = Off
Dante_presat = FALSE
Initial_wait = 1[s]
Recvr_gain = 34
Relaxation_delay = 5[s]
Repetition_time = 5.11534336[s]
Temp_get = 20.9[dC]
abundance
X : parts per Million : 19F

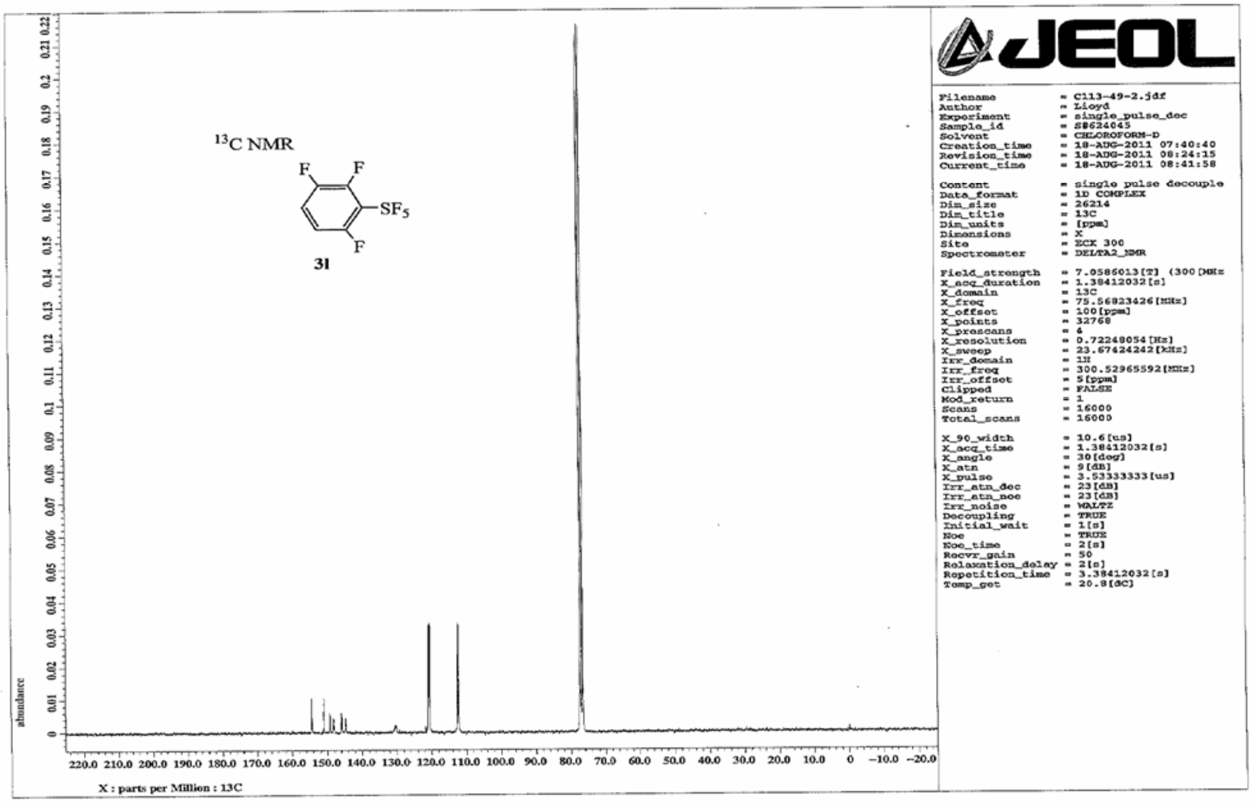
13C NMR
F
F
SF5
F
31
Filename = C113-49-2.jdf
Author = Lioyd
Experiment = single_pulse_dec
Sample_id = S#624045
Solvent = CHLOROFORM-D
Creation_time = 18-AUG-2011 07:40:40
Revision_time = 18-AUG-2011 08:24:15
Current_time = 18-AUG-2011 08:41:58
Content = single pulse decouple
Data_format = 1D COMPLEX
Dim_size = 26214
Dim_title = 13C
Dim_units = [ppm]
Dimensions = X
Site = ECX 300
Spectrometer = DELTA2_NMR
Field_strength = 7.0586013[T] (300[MHz
X_acq_duration = 1.38412032[s]
X_domain = 13C
X_freq = 75.56823426[MHz]
X_offset = 100[ppm]
X_points = 32768
X_prescans = 4
X_resolution = 0.72248054[Hz]
X_sweep = 23.67424242[kHz]
Irr_domain = 1H
Irr_freq = 300.52965592[MHz]
Irr_offset = 5[ppm]
Clipped = FALSE
Mod_return = 1
Scans = 16000
Total_scans = 16000
X_90_width = 10.6[us]
X_acq_time = 1.38412032[s]
X_angle = 30[deg]
X_atn = 9[dB]
X_pulse = 3.53333333[us]
Irr_atn_dec = 23[dB]
Irr_atn_noe = 23[dB]
Irr_noise = WALTZ
Decoupling = TRUE
Initial_wait = 1[s]
Noe = TRUE
Noe_time = 2[s]
Recvr_gain = 50
Relaxation_delay = 2[s]
Repetition_time = 3.38412032[s]
Temp_get = 20.8[dC]
abundance
X : parts per Million : 13C

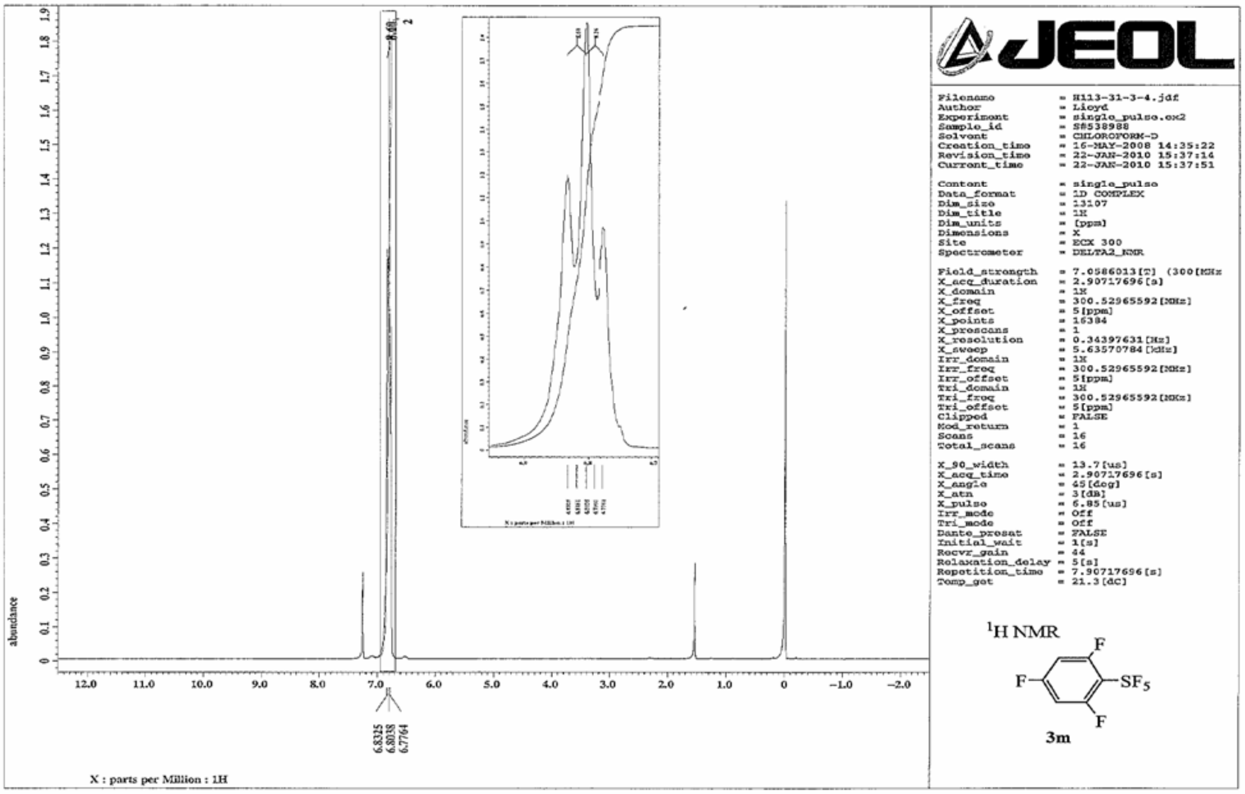

abundance
X : parts per Million : 1H
6.8325
6.8038
6.7764
Filename = H113-31-3-4.jdf
Author = Lioyd
Experiment = single_pulse.ex2
Sample_id = S#538988
Solvent = CHLOROFORM-D
Creation_time = 16-MAY-2008 14:35:22
Revision_time = 22-JAN-2010 15:37:14
Current_time = 22-JAN-2010 15:37:51
Content = single_pulse
Data_format = 1D COMPLEX
Dim_size = 13107
Dim_title = 1H
Dim_units = [ppm]
Dimensions = X
Site = ECX 300
Spectrometer = DELTA2_NMR
Field_strength = 7.0586013[T] (300[MHz
X_acq_duration = 2.90717696[s]
X_domain = 1H
X_freq = 300.52965592[MHz]
X_offset = 5[ppm]
X_points = 16384
X_prescans = 1
X_resolution = 0.34397631[Hz]
X_sweep = 5.63570784[kHz]
Irr_domain = 1H
Irr_freq = 300.52965592[MHz]
Irr_offset = 5[ppm]
Tri_domain = 1H
Tri_freq = 300.52965592[MHz]
Tri_offset = 5[ppm]
Clipped = FALSE
Mod_return = 1
Scans = 16
Total_scans = 16
X_90_width = 13.7[us]
X_acq_time = 2.90717696[s]
X_angle = 45[deg]
X_atn = 3[dB]
X_pulse = 6.85[us]
Irr_mode = Off
Tri_mode = Off
Dante_presat = FALSE
Initial_wait = 1[s]
Recvr_gain = 44
Relaxation_delay = 5[s]
Repetition_time = 7.90717696[s]
Temp_get = 21.3[dC]
1H NMR
F
F
SF5
F
3m

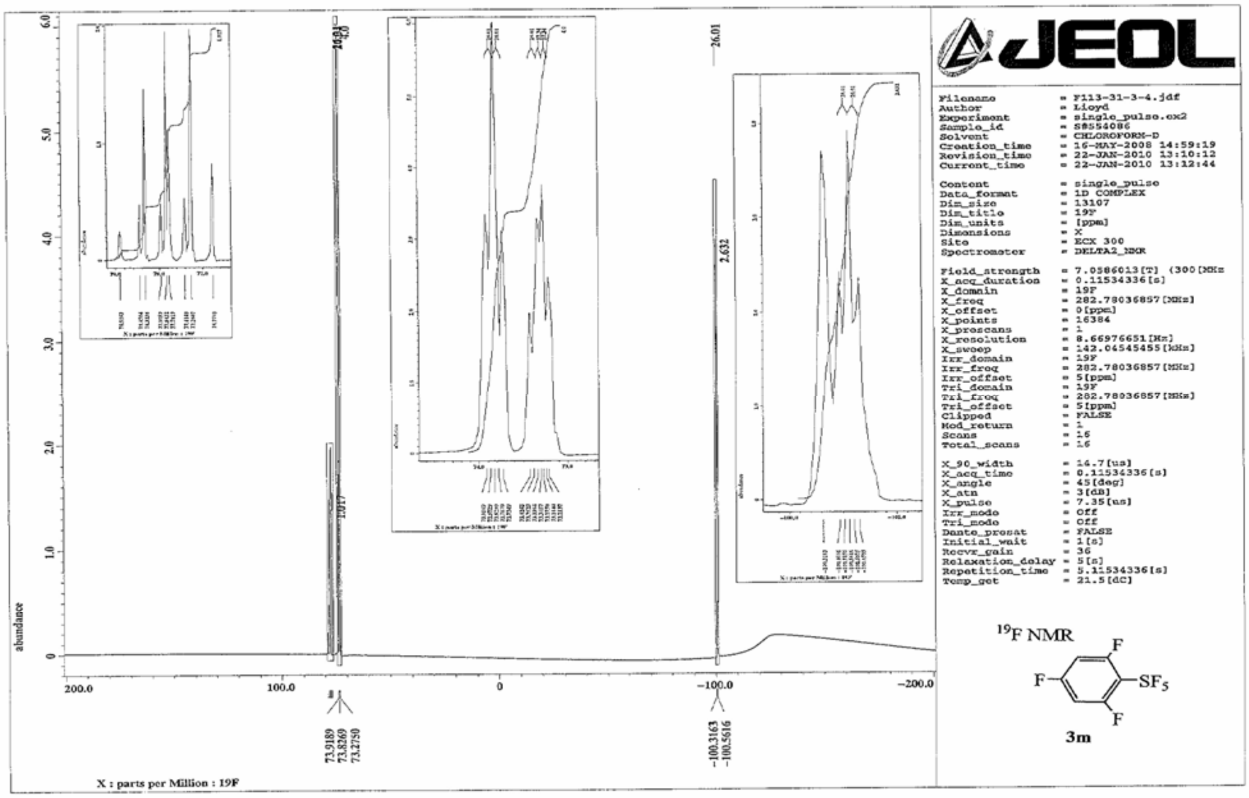

abundance
X : parts per Million : 19F
73.9189
73.8269
73.2750
−100.3163
−100.5616
−26.01
2.632
Filename = F113-31-3-4.jdf
Author = Lioyd
Experiment = single_pulse.ex2
Sample_id = S#554086
Solvent = CHLOROFORM-D
Creation_time = 16-MAY-2008 14:59:19
Revision_time = 22-JAN-2010 13:10:12
Current_time = 22-JAN-2010 13:12:44
Content = single_pulse
Data_format = 1D COMPLEX
Dim_size = 13107
Dim_title = 19F
Dim_units = [ppm]
Dimensions = X
Site = ECX 300
Spectrometer = DELTA2_NMR
Field_strength = 7.0586013[T] (300[MHz
X_acq_duration = 0.11534336[s]
X_domain = 19F
X_freq = 282.78036857[MHz]
X_offset = 0[ppm]
X_points = 16384
X_prescans = 1
X_resolution = 8.66976651[Hz]
X_sweep = 142.04545455[kHz]
Irr_domain = 19F
Irr_freq = 282.78036857[MHz]
Irr_offset = 5[ppm]
Tri_domain = 19F
Tri_freq = 282.78036857[MHz]
Tri_offset = 5[ppm]
Clipped = FALSE
Mod_return = 1
Scans = 16
Total_scans = 16
X_90_width = 14.7[us]
X_acq_time = 0.11534336[s]
X_angle = 45[deg]
X_atn = 3[dB]
X_pulse = 7.35[us]
Irr_mode = Off
Tri_mode = Off
Dante_presat = FALSE
Initial_wait = 1[s]
Recvr_gain = 36
Relaxation_delay = 5[s]
Repetition_time = 5.11534336[s]
Temp_get = 21.5[dC]
19F NMR
F
F
SF5
F
3m

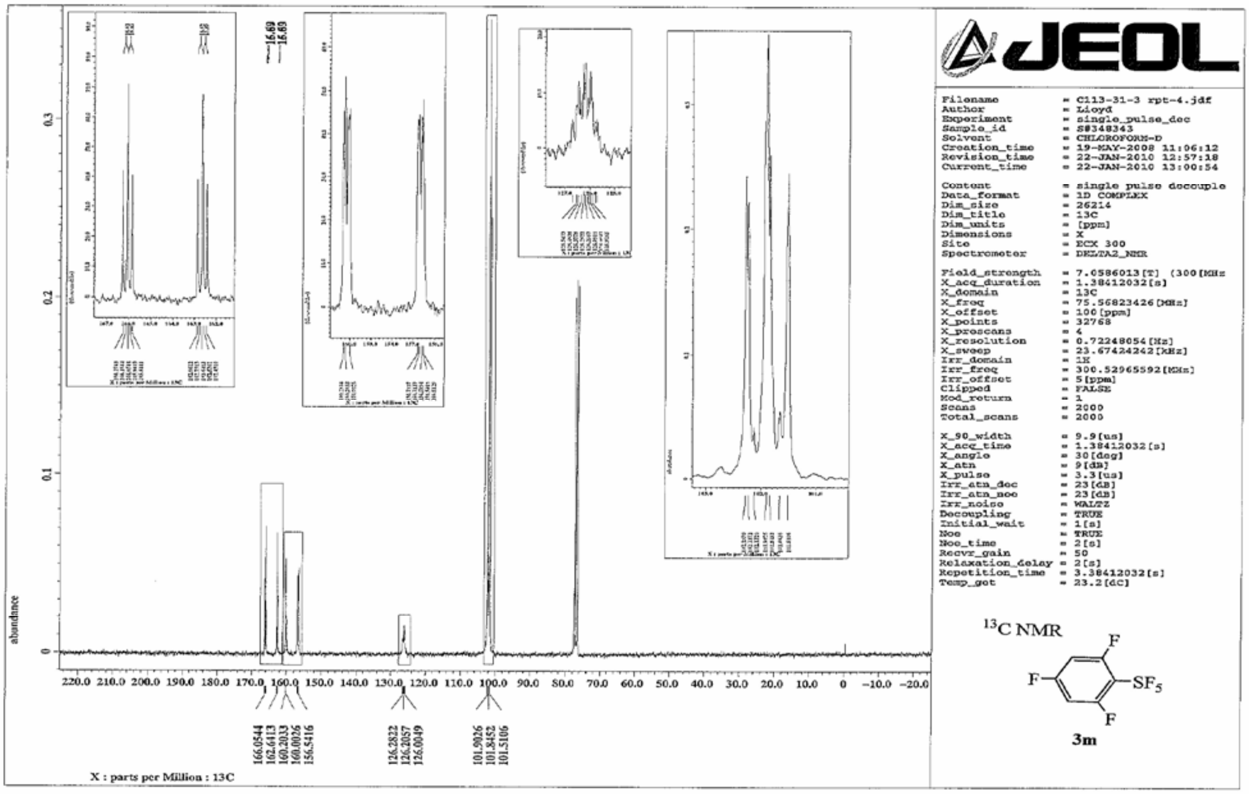
JEOL
Filename = C113-31-3 rpt-4.jdf
Author = Lloyd
Experiment = single_pulse_dec
Sample_id = S#348343
Solvent = CHLOROFORM-D
Creation_time = 19-MAY-2008 11:06:12
Revision_time = 22-JAN-2010 12:57:18
Current_time = 22-JAN-2010 13:00:54
Content = single pulse decouple
Data_format = 1D COMPLEX
Dim_size = 26214
Dim_title = 13C
Dim_units = [ppm]
Dimensions = X
Site = ECX 300
Spectrometer = DELTA2_NMR
Field_strength = 7.0586013[T] (300[MHz
X_acq_duration = 1.38412032[s]
X_domain = 13C
X_freq = 75.56823426[MHz]
X_offset = 100[ppm]
X_points = 32768
X_prescans = 4
X_resolution = 0.72248054[Hz]
X_sweep = 23.67424242[kHz]
Irr_domain = 1H
Irr_freq = 300.52965592[MHz]
Irr_offset = 5[ppm]
Clipped = FALSE
Mod_return = 1
Scans = 2000
Total_scans = 2000
X_90_width = 9.9[us]
X_acq_time = 1.38412032[s]
X_angle = 30[deg]
X_atn = 9[dB]
X_pulse = 3.3[us]
Irr_atn_dec = 23[dB]
Irr_atn_noe = 23[dB]
Irr_noise = WALTZ
Decoupling = TRUE
Initial_wait = 1[s]
Noe = TRUE
Noe_time = 2[s]
Recvr_gain = 50
Relaxation_delay = 2[s]
Repetition_time = 3.38412032[s]
Temp_get = 23.2[dC]
13C NMR
F
F
SF5
F
3m
166.0544
162.6413
160.2033
160.0026
156.5416
126.2822
126.2057
126.0049
101.9026
101.8452
101.5106
X : parts per Million : 13C
abundance

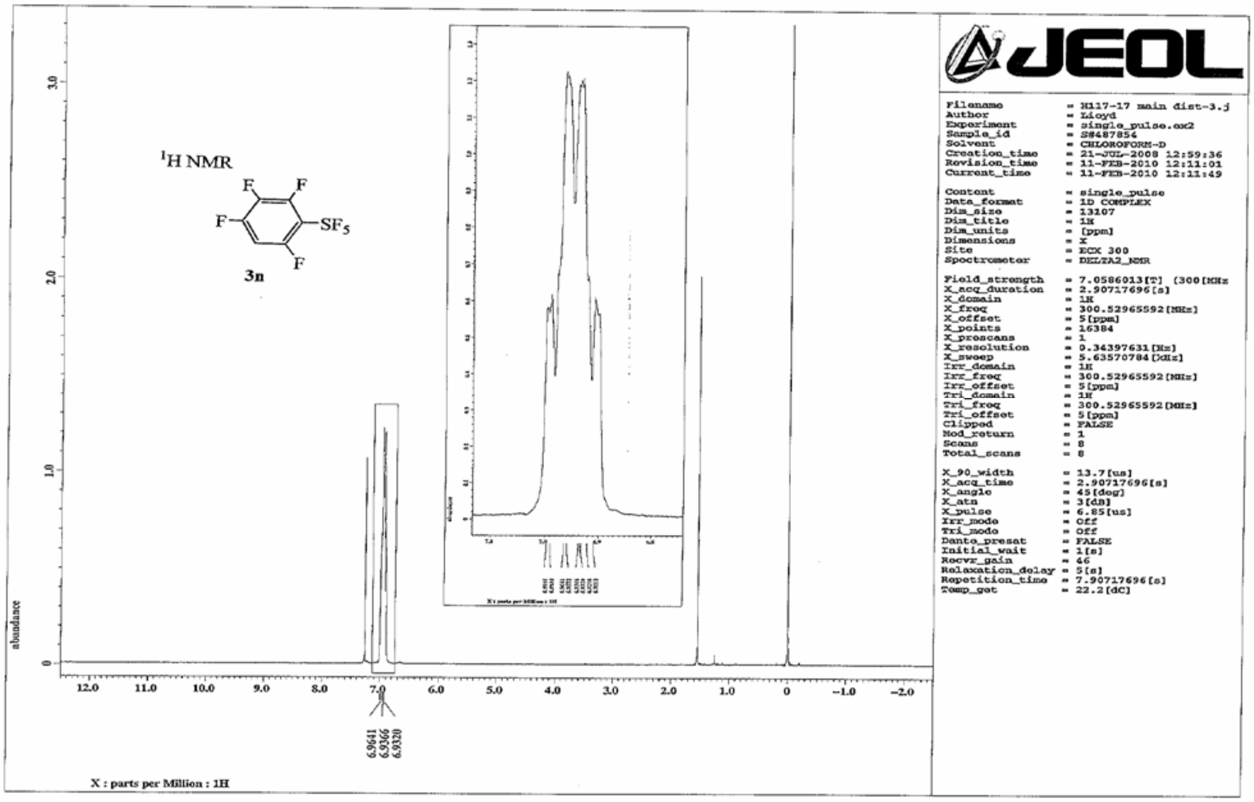
JEOL
Filename = H117-17 main dist-3.j
Author = Lloyd
Experiment = single_pulse.ex2
Sample_id = S#487854
Solvent = CHLOROFORM-D
Creation_time = 21-JUL-2008 12:59:36
Revision_time = 11-FEB-2010 12:11:01
Current_time = 11-FEB-2010 12:11:49
Content = single_pulse
Data_format = 1D COMPLEX
Dim_size = 13107
Dim_title = 1H
Dim_units = [ppm]
Dimensions = X
Site = ECX 300
Spectrometer = DELTA2_NMR
Field_strength = 7.0586013[T] (300[MHz
X_acq_duration = 2.90717696[s]
X_domain = 1H
X_freq = 300.52965592[MHz]
X_offset = 5[ppm]
X_points = 16384
X_prescans = 1
X_resolution = 0.34397631[Hz]
X_sweep = 5.63570784[kHz]
Irr_domain = 1H
Irr_freq = 300.52965592[MHz]
Irr_offset = 5[ppm]
Tri_domain = 1H
Tri_freq = 300.52965592[MHz]
Tri_offset = 5[ppm]
Clipped = FALSE
Mod_return = 1
Scans = 8
Total_scans = 8
X_90_width = 13.7[us]
X_acq_time = 2.90717696[s]
X_angle = 45[deg]
X_atn = 3[dB]
X_pulse = 6.85[us]
Irr_mode = Off
Tri_mode = Off
Dante_presat = FALSE
Initial_wait = 1[s]
Recvr_gain = 46
Relaxation_delay = 5[s]
Repetition_time = 7.90717696[s]
Temp_get = 22.2[dC]
1H NMR
F
F
F
SF5
F
3n
6.9641
6.9366
6.9320
X : parts per Million : 1H
abundance

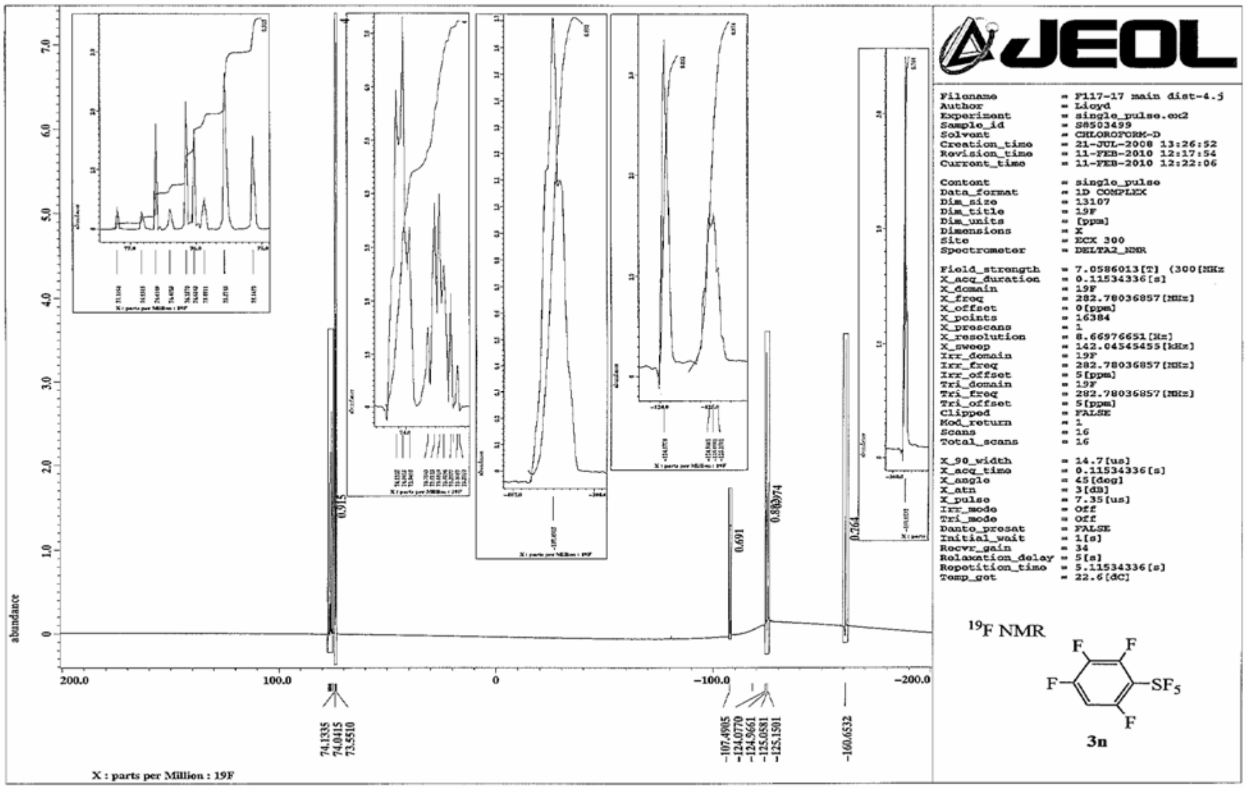
JEOL
Filename = F117-17 main dist-4.j
Author = Lloyd
Experiment = single_pulse.ex2
Sample_id = S8503499
Solvent = CHLOROFORM-D
Creation_time = 21-JUL-2008 13:26:52
Revision_time = 11-FEB-2010 12:17:54
Current_time = 11-FEB-2010 12:22:06
Content = single_pulse
Data_format = 1D COMPLEX
Dim_size = 13107
Dim_title = 19F
Dim_units = [ppm]
Dimensions = X
Site = ECX 300
Spectrometer = DELTA2_NMR
Field_strength = 7.0586013[T] (300[MHz
X_acq_duration = 0.11534336[s]
X_domain = 19F
X_freq = 282.78036857[MHz]
X_offset = 0[ppm]
X_points = 16384
X_prescans = 1
X_resolution = 8.66976651[Hz]
X_sweep = 142.04545455[kHz]
Irr_domain = 19F
Irr_freq = 282.78036857[MHz]
Irr_offset = 5[ppm]
Tri_domain = 19F
Tri_freq = 282.78036857[MHz]
Tri_offset = 5[ppm]
Clipped = FALSE
Mod_return = 1
Scans = 16
Total_scans = 16
X_90_width = 14.7[us]
X_acq_time = 0.11534336[s]
X_angle = 45[deg]
X_atn = 3[dB]
X_pulse = 7.35[us]
Irr_mode = Off
Tri_mode = Off
Dante_presat = FALSE
Initial_wait = 1[s]
Recvr_gain = 34
Relaxation_delay = 5[s]
Repetition_time = 5.11534336[s]
Temp_get = 22.6[dC]
19F NMR
3n
abundance
X : parts per Million : 19F
74.1335
74.0415
73.5510
-107.4905
-124.0770
-124.9661
-125.0581
-125.1501
-160.6532
0.915
0.691
0.883
0.974
0.764

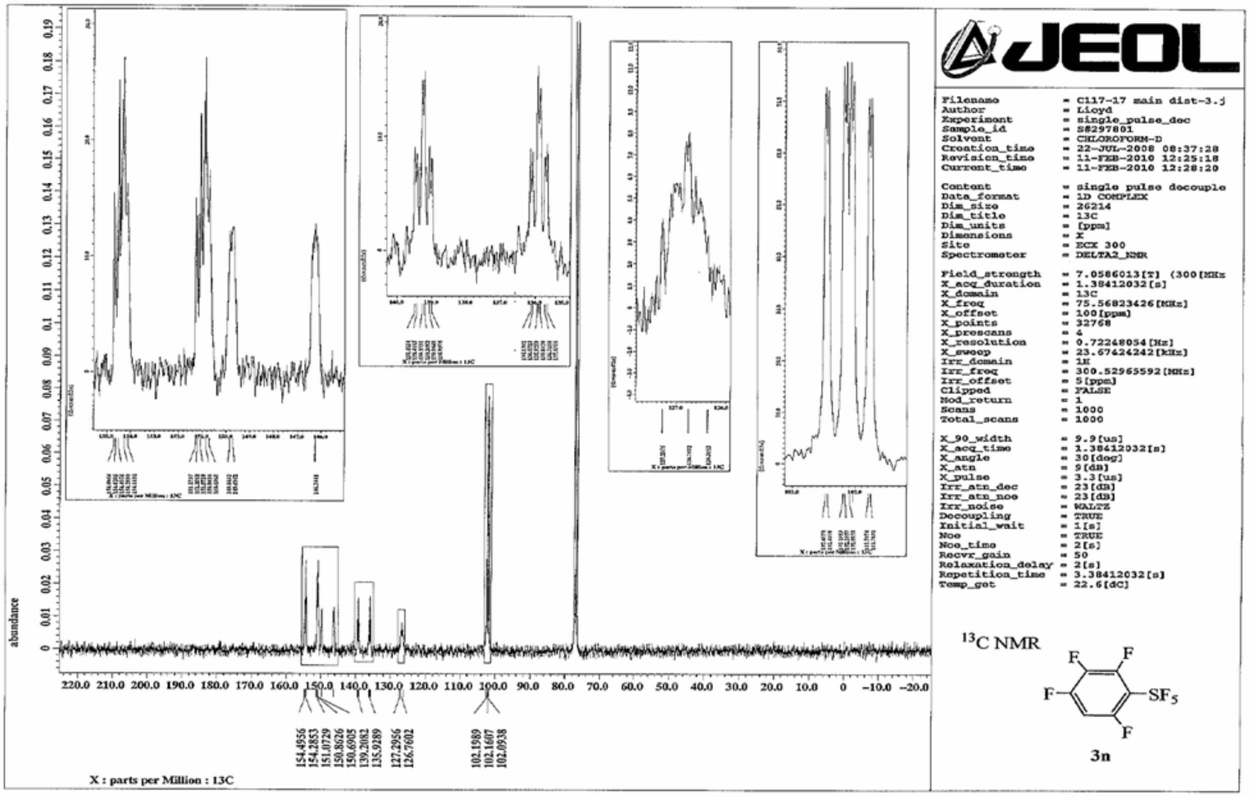
JEOL
Filename = C117-17 main dist-3.j
Author = Lloyd
Experiment = single_pulse_dec
Sample_id = S8297801
Solvent = CHLOROFORM-D
Creation_time = 22-JUL-2008 08:37:28
Revision_time = 11-FEB-2010 12:25:18
Current_time = 11-FEB-2010 12:28:20
Content = single pulse decouple
Data_format = 1D COMPLEX
Dim_size = 26214
Dim_title = 13C
Dim_units = [ppm]
Dimensions = X
Site = ECX 300
Spectrometer = DELTA2_NMR
Field_strength = 7.0586013[T] (300[MHz
X_acq_duration = 1.38412032[s]
X_domain = 13C
X_freq = 75.56823426[MHz]
X_offset = 100[ppm]
X_points = 32768
X_prescans = 4
X_resolution = 0.72248054[Hz]
X_sweep = 23.67424242[kHz]
Irr_domain = 1H
Irr_freq = 300.52965592[MHz]
Irr_offset = 5[ppm]
Clipped = FALSE
Mod_return = 1
Scans = 1000
Total_scans = 1000
X_90_width = 9.9[us]
X_acq_time = 1.38412032[s]
X_angle = 30[deg]
X_atn = 9[dB]
X_pulse = 3.3[us]
Irr_atn_dec = 23[dB]
Irr_atn_noe = 23[dB]
Irr_noise = WALTZ
Decoupling = TRUE
Initial_wait = 1[s]
Noe = TRUE
Noe_time = 2[s]
Recvr_gain = 50
Relaxation_delay = 2[s]
Repetition_time = 3.38412032[s]
Temp_get = 22.6[dC]
13C NMR
3n
abundance
X : parts per Million : 13C
154.4956
154.2853
151.0729
150.8626
150.6905
139.2082
135.9289
127.2956
126.7602
102.1989
102.1607
102.0938

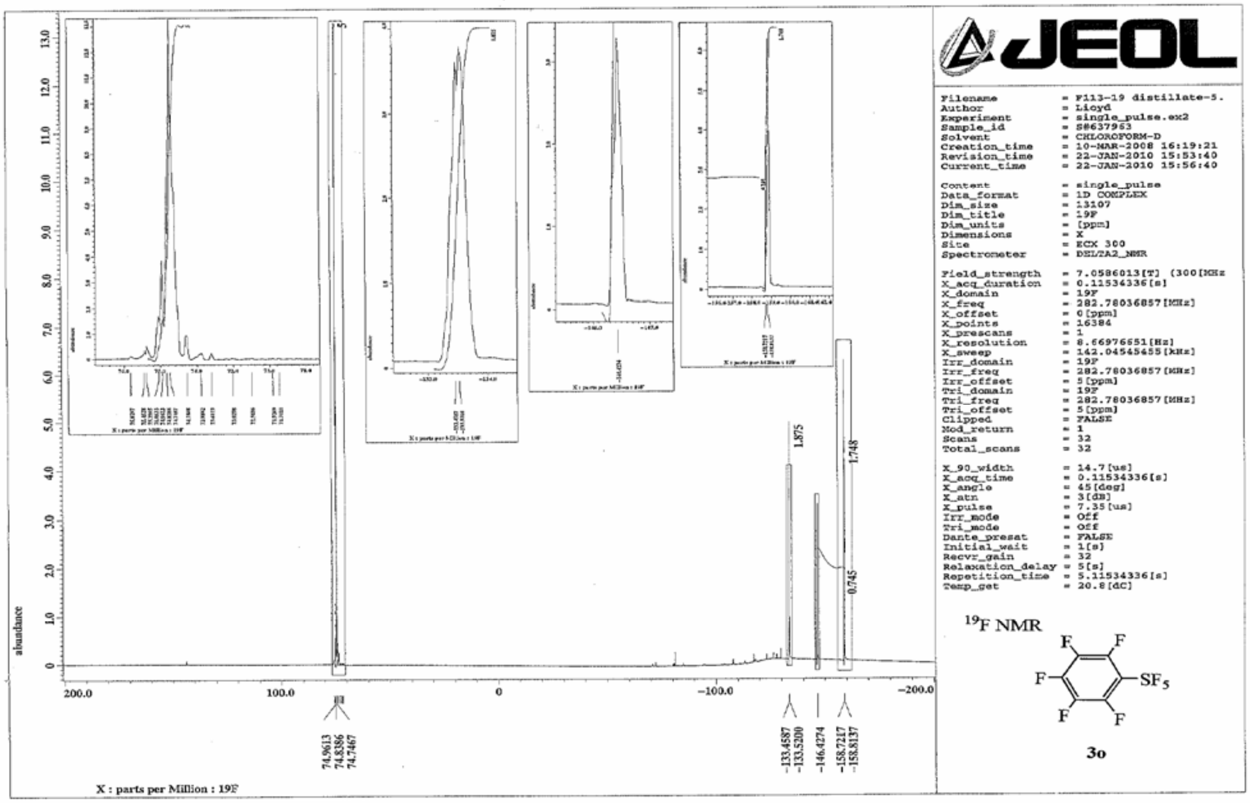
JEOL
Filename = F113-19 distillate-5.
Author = Lioyd
Experiment = single_pulse.ex2
Sample_id = S8637963
Solvent = CHLOROFORM-D
Creation_time = 10-MAR-2008 16:19:21
Revision_time = 22-JAN-2010 15:53:40
Current_time = 22-JAN-2010 15:56:40
Content = single_pulse
Data_format = 1D COMPLEX
Dim_size = 13107
Dim_title = 19F
Dim_units = [ppm]
Dimensions = X
Site = ECX 300
Spectrometer = DELTA2_NMR
Field_strength = 7.0586013[T] (300[MHz
X_acq_duration = 0.11534336[s]
X_domain = 19F
X_freq = 282.78036857[MHz]
X_offset = 0[ppm]
X_points = 16384
X_prescans = 1
X_resolution = 8.66976651[Hz]
X_sweep = 142.04545455[kHz]
Irr_domain = 19F
Irr_freq = 282.78036857[MHz]
Irr_offset = 5[ppm]
Tri_domain = 19F
Tri_freq = 282.78036857[MHz]
Tri_offset = 5[ppm]
Clipped = FALSE
Mod_return = 1
Scans = 32
Total_scans = 32
X_90_width = 14.7[us]
X_acq_time = 0.11534336[s]
X_angle = 45[deg]
X_atn = 3[dB]
X_pulse = 7.35[us]
Irr_mode = Off
Tri_mode = Off
Dante_presat = FALSE
Initial_wait = 1[s]
Recvr_gain = 32
Relaxation_delay = 5[s]
Repetition_time = 5.11534336[s]
Temp_get = 20.8[dC]
^{19}F NMR
F
SF$_5$
3o
1.875
1.748
0.745
74.9613
74.8386
74.7467
-133.4587
-133.5200
-146.4274
-158.7217
-158.8137
200.0
100.0
0
-100.0
-200.0
abundance
X : parts per Million : 19F

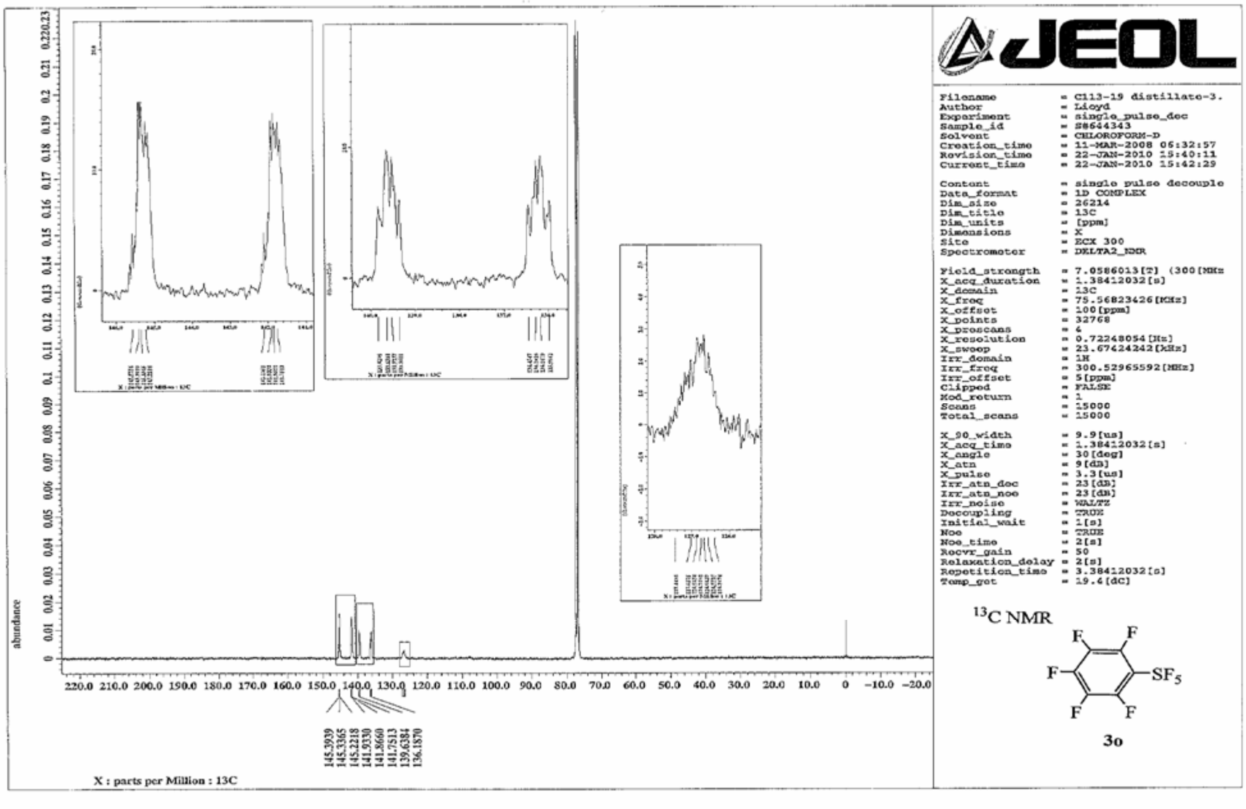
JEOL
Filename = C113-19 distillate-3.
Author = Lioyd
Experiment = single_pulse_dec
Sample_id = S8644343
Solvent = CHLOROFORM-D
Creation_time = 11-MAR-2008 06:32:57
Revision_time = 22-JAN-2010 15:40:11
Current_time = 22-JAN-2010 15:42:29
Content = single pulse decouple
Data_format = 1D COMPLEX
Dim_size = 26214
Dim_title = 13C
Dim_units = [ppm]
Dimensions = X
Site = ECX 300
Spectrometer = DELTA2_NMR
Field_strength = 7.0586013[T] (300[MHz
X_acq_duration = 1.38412032[s]
X_domain = 13C
X_freq = 75.56823426[MHz]
X_offset = 100[ppm]
X_points = 32768
X_prescans = 4
X_resolution = 0.72248054[Hz]
X_sweep = 23.67424242[kHz]
Irr_domain = 1H
Irr_freq = 300.52965592[MHz]
Irr_offset = 5[ppm]
Clipped = FALSE
Mod_return = 1
Scans = 15000
Total_scans = 15000
X_90_width = 9.9[us]
X_acq_time = 1.38412032[s]
X_angle = 30[deg]
X_atn = 9[dB]
X_pulse = 3.3[us]
Irr_atn_dec = 23[dB]
Irr_atn_noe = 23[dB]
Irr_noise = WALTZ
Decoupling = TRUE
Initial_wait = 1[s]
Noe = TRUE
Noe_time = 2[s]
Recvr_gain = 50
Relaxation_delay = 2[s]
Repetition_time = 3.38412032[s]
Temp_get = 19.4[dC]
^{13}C NMR
F
SF$_5$
3o
145.3939
145.3365
145.2218
141.9330
141.8660
141.7513
139.6384
136.1870
220.0 210.0 200.0 190.0 180.0 170.0 160.0 150.0 140.0 130.0 120.0 110.0 100.0 90.0 80.0 70.0 60.0 50.0 40.0 30.0 20.0 10.0 0 -10.0 -20.0
abundance
X : parts per Million : 13C

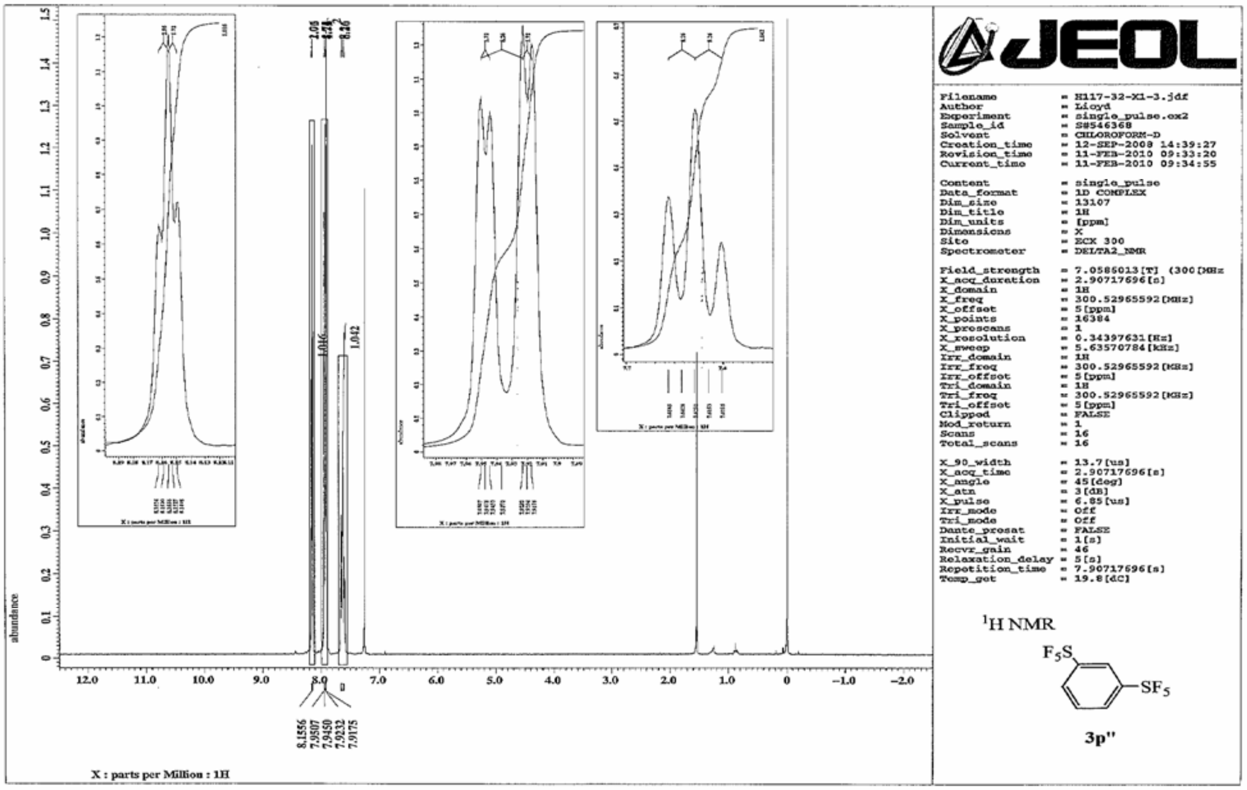
JEOL
Filename = H117-32-X1-3.jdf
Solvent = CHLOROFORM-D
Dim_title = 1H
Site = ECX 300
^{1}H NMR
F_5S
SF_5
3p''
X : parts per Million : 1H
abundance
8.1556
7.9507
7.9450
7.9232
7.9175

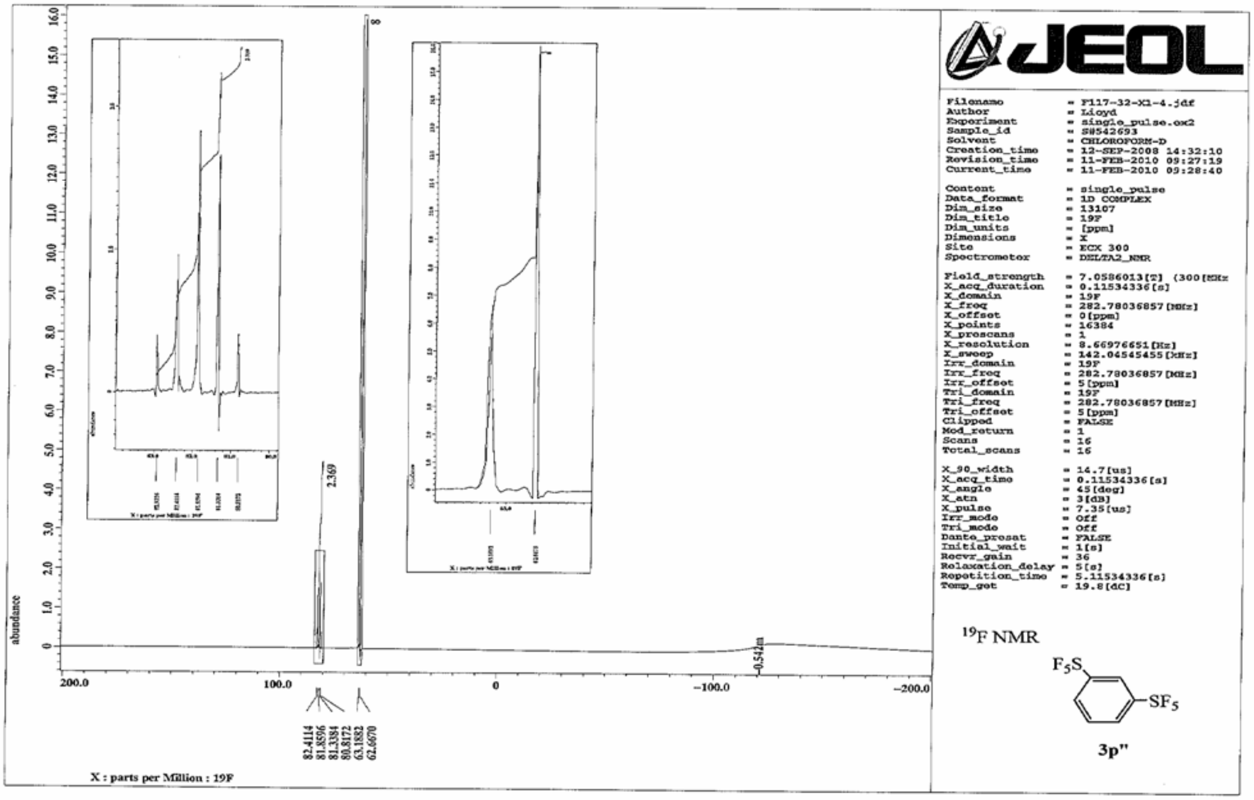
JEOL
Filename = F117-32-X1-4.jdf
Solvent = CHLOROFORM-D
Dim_title = 19F
Site = ECX 300
^{19}F NMR
F_5S
SF_5
3p''
X : parts per Million : 19F
abundance
82.4114
81.8556
81.3384
80.8172
63.1882
62.6670

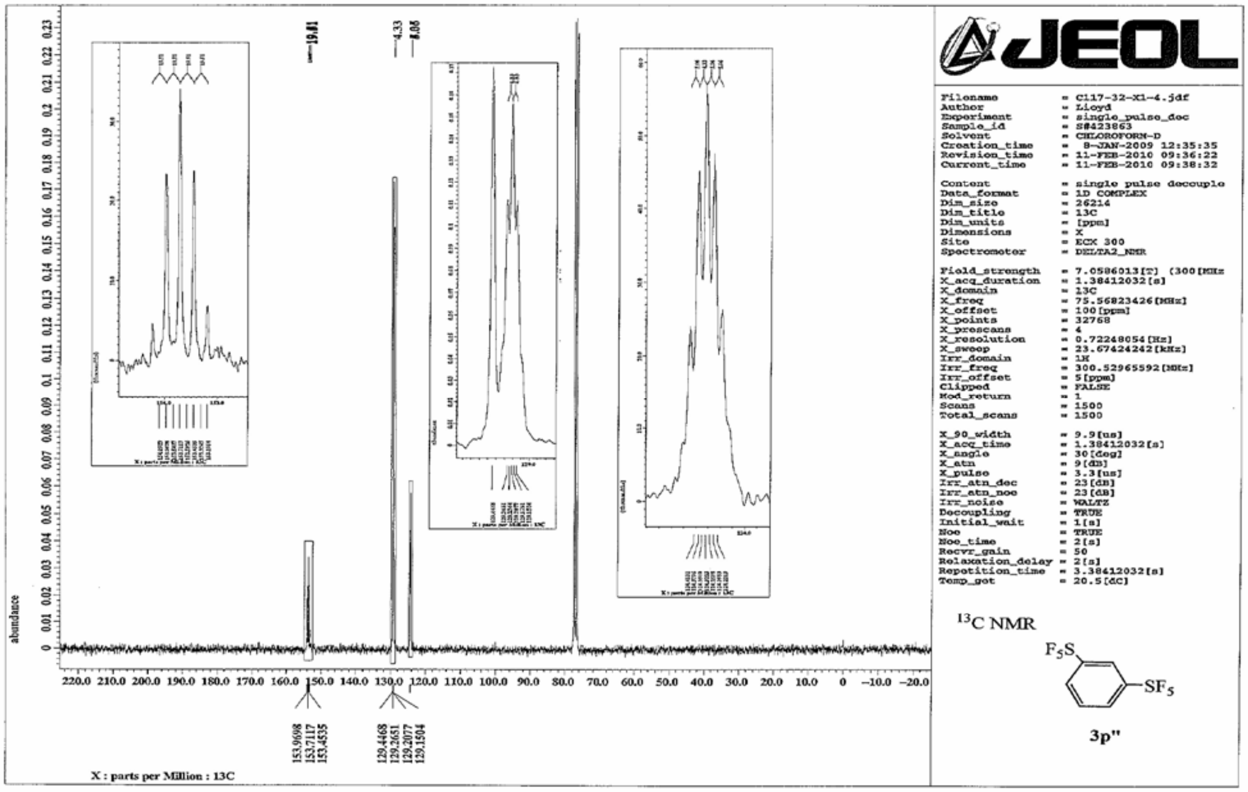
JEOL
Filename = C117-32-X1-4.jdf
Author = Lloyd
Experiment = single_pulse_dec
Solvent = CHLOROFORM-D
Content = single pulse decouple
Dim_title = 13C
Spectrometer = DELTA2_NMR
Field_strength = 7.0586013[T] (300[MHz
X_freq = 75.56823426[MHz]
Scans = 1500
Total_scans = 1500
Temp_get = 20.5[dC]
13C NMR
F_5S
SF_5
3p"
abundance
X : parts per Million : 13C
153.9698
153.7117
153.4535
129.4468
129.2651
129.2077
129.1504

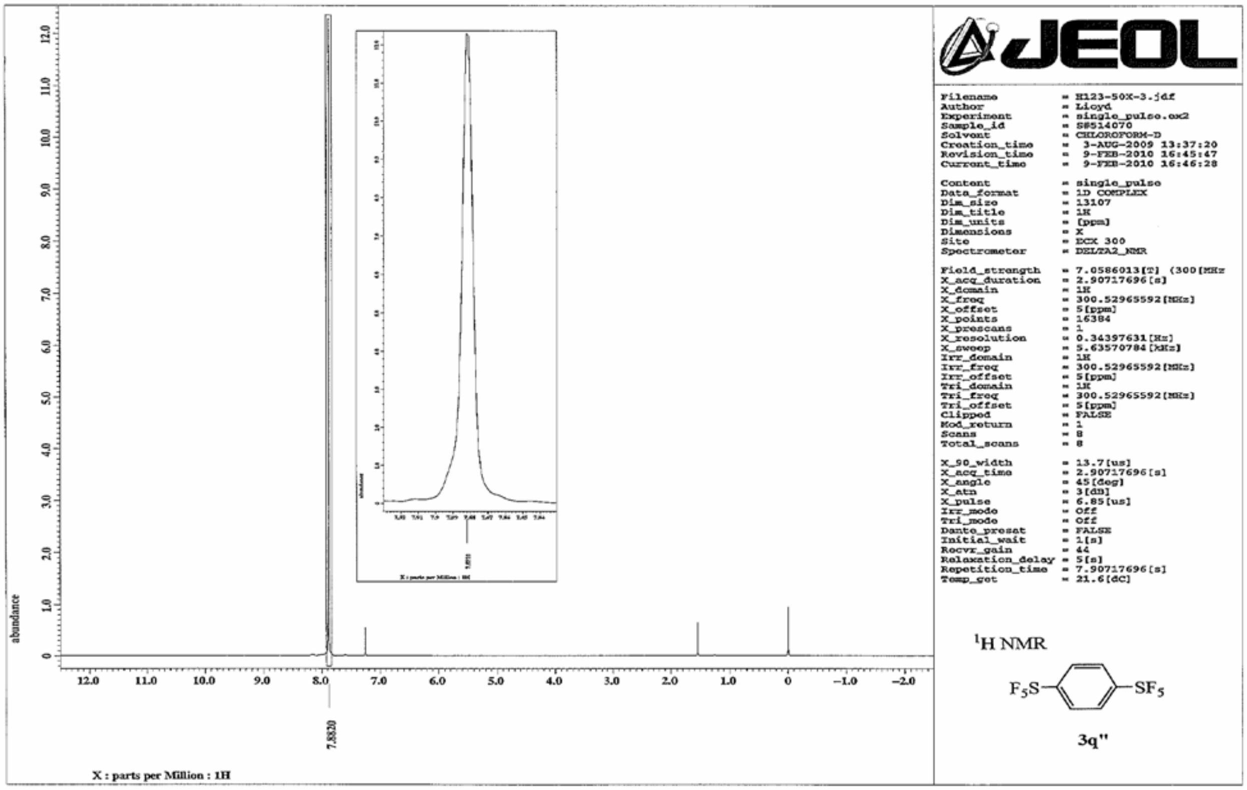
JEOL
Filename = H123-50X-3.jdf
Author = Lloyd
Experiment = single_pulse.ex2
Solvent = CHLOROFORM-D
Content = single_pulse
Dim_title = 1H
Spectrometer = DELTA2_NMR
Field_strength = 7.0586013[T] (300[MHz
X_freq = 300.52965592[MHz]
Scans = 8
Total_scans = 8
Temp_get = 21.6[dC]
1H NMR
F_5S
SF_5
3q"
abundance
X : parts per Million : 1H
7.8820

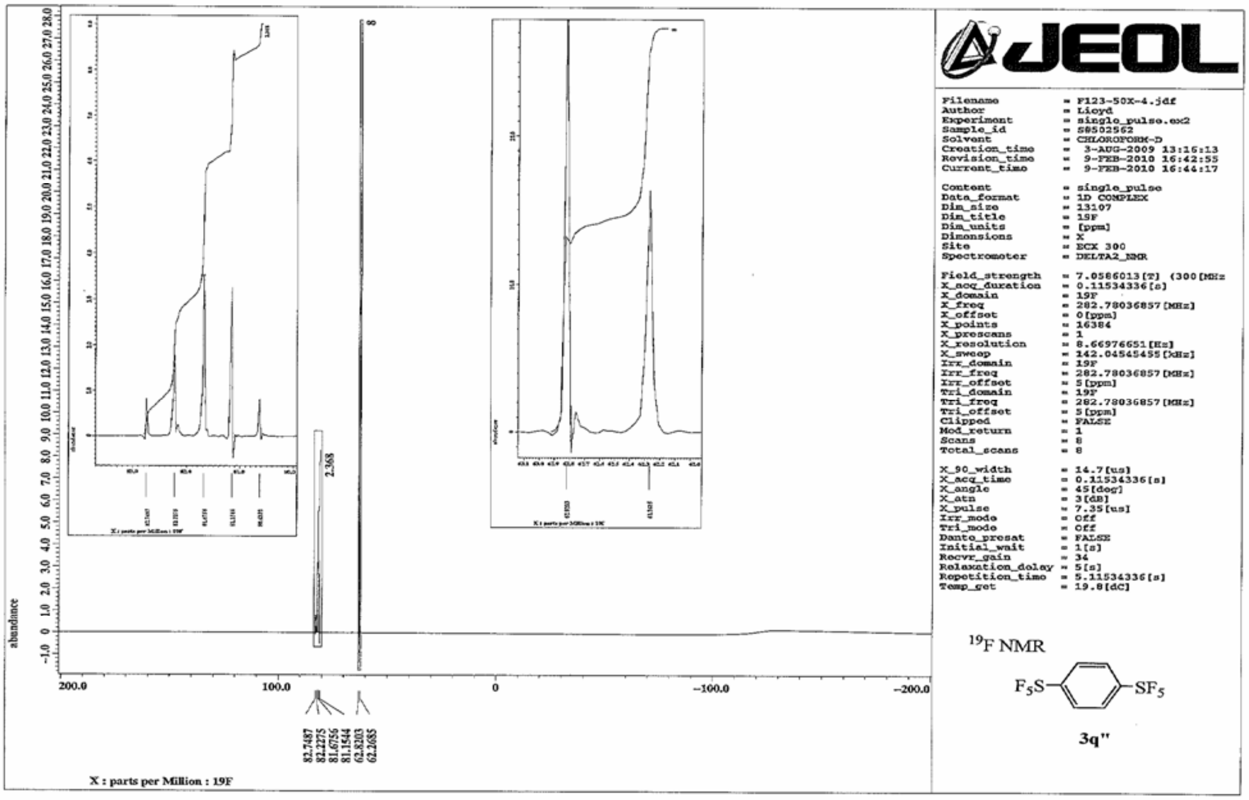
JEOL
^{19}F NMR
F_5S–C$_6$H$_4$–SF_5
3q''
X : parts per Million : 19F
Filename = F123-50X-4.jdf
Solvent = CHLOROFORM-D
82.7487
82.2275
81.6756
81.1544
62.8203
62.2685

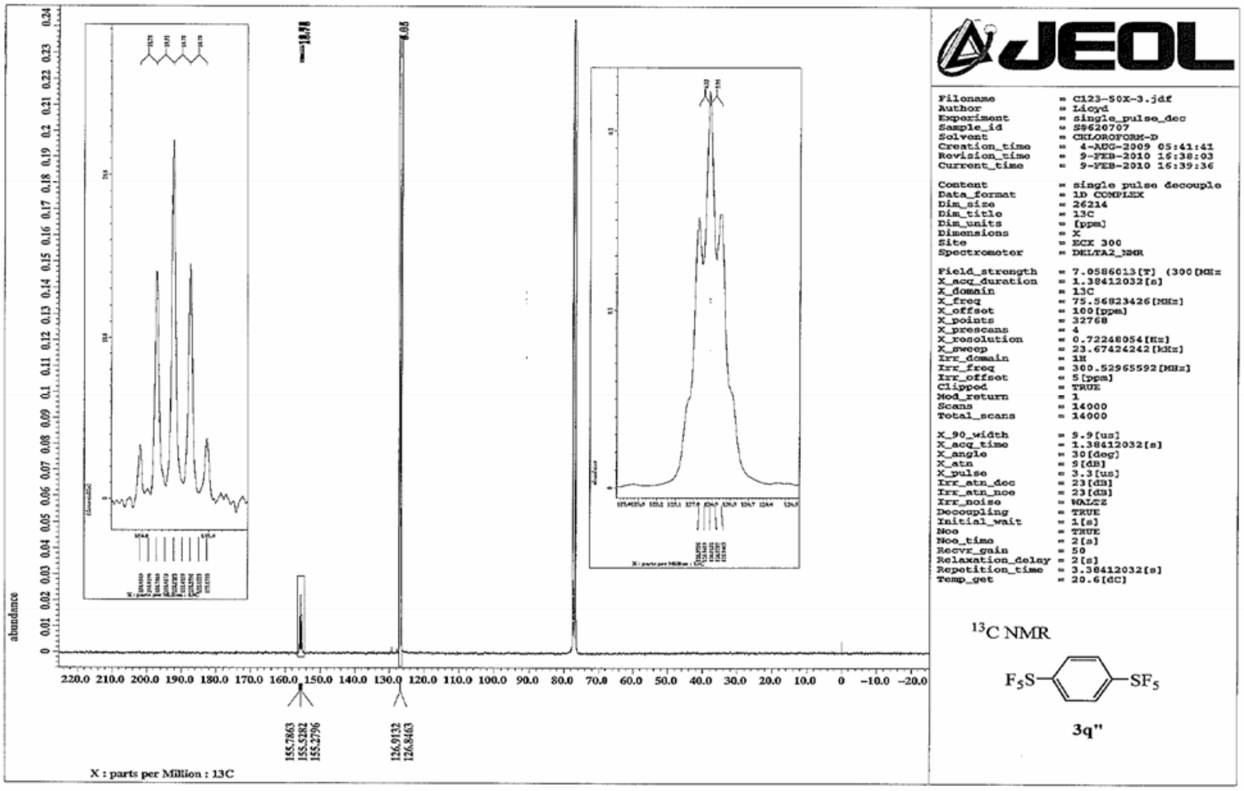
JEOL
^{13}C NMR
F_5S–C$_6$H$_4$–SF_5
3q''
X : parts per Million : 13C
Filename = C123-50X-3.jdf
Solvent = CHLOROFORM-D
155.7863
155.5282
155.2796
126.9132
126.8463

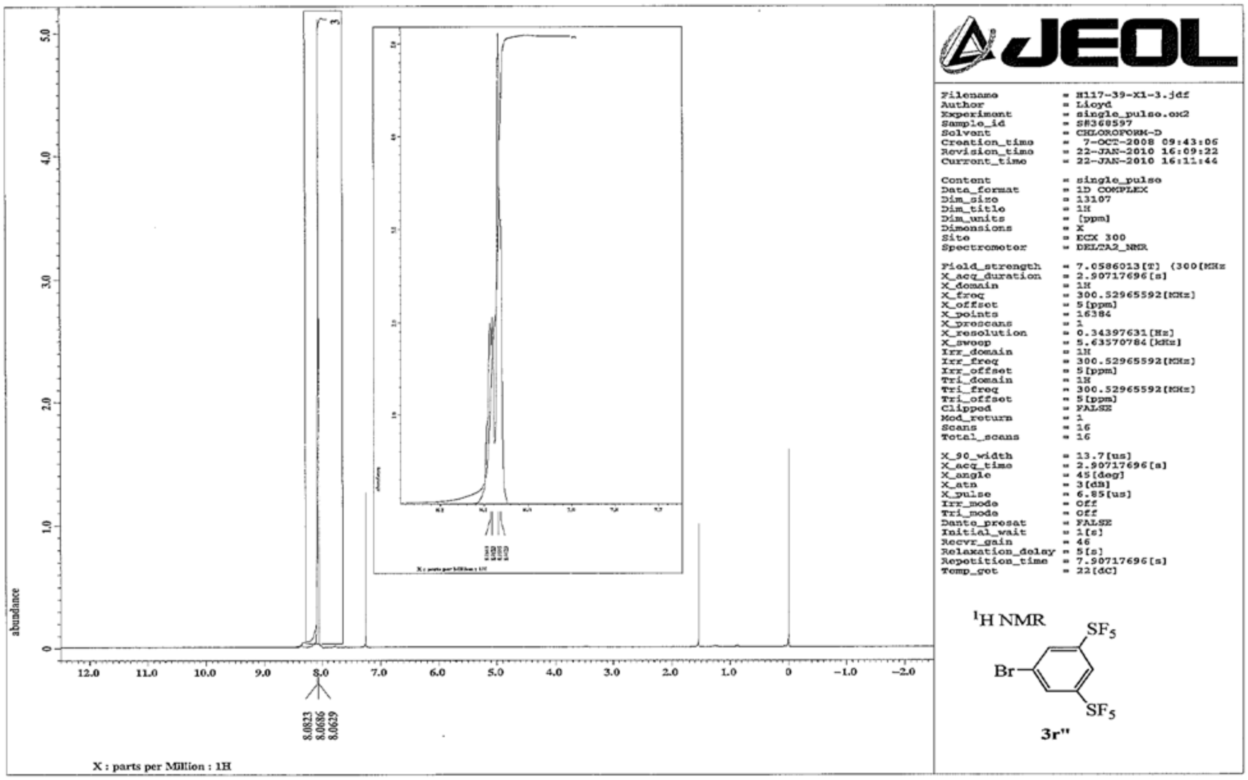
JEOL
Filename = H117-39-X1-3.jdf
Author = Lioyd
Experiment = single_pulse.ex2
Sample_id = S#368597
Solvent = CHLOROFORM-D
Creation_time = 7-OCT-2008 09:43:06
Revision_time = 22-JAN-2010 16:09:22
Current_time = 22-JAN-2010 16:11:44
Content = single_pulse
Data_format = 1D COMPLEX
Dim_size = 13107
Dim_title = 1H
Dim_units = [ppm]
Dimensions = X
Site = ECX 300
Spectrometer = DELTA2_NMR
Field_strength = 7.0586013[T] (300[MHz
X_acq_duration = 2.90717696[s]
X_domain = 1H
X_freq = 300.52965592[MHz]
X_offset = 5[ppm]
X_points = 16384
X_prescans = 1
X_resolution = 0.34397631[Hz]
X_sweep = 5.63570784[kHz]
Irr_domain = 1H
Irr_freq = 300.52965592[MHz]
Irr_offset = 5[ppm]
Tri_domain = 1H
Tri_freq = 300.52965592[MHz]
Tri_offset = 5[ppm]
Clipped = FALSE
Mod_return = 1
Scans = 16
Total_scans = 16
X_90_width = 13.7[us]
X_acq_time = 2.90717696[s]
X_angle = 45[deg]
X_atn = 3[dB]
X_pulse = 6.85[us]
Irr_mode = Off
Tri_mode = Off
Dante_presat = FALSE
Initial_wait = 1[s]
Recvr_gain = 46
Relaxation_delay = 5[s]
Repetition_time = 7.90717696[s]
Temp_get = 22[dC]
1H NMR
SF5
Br
SF5
3r''
abundance
8.0823
8.0686
8.0629
X : parts per Million : 1H

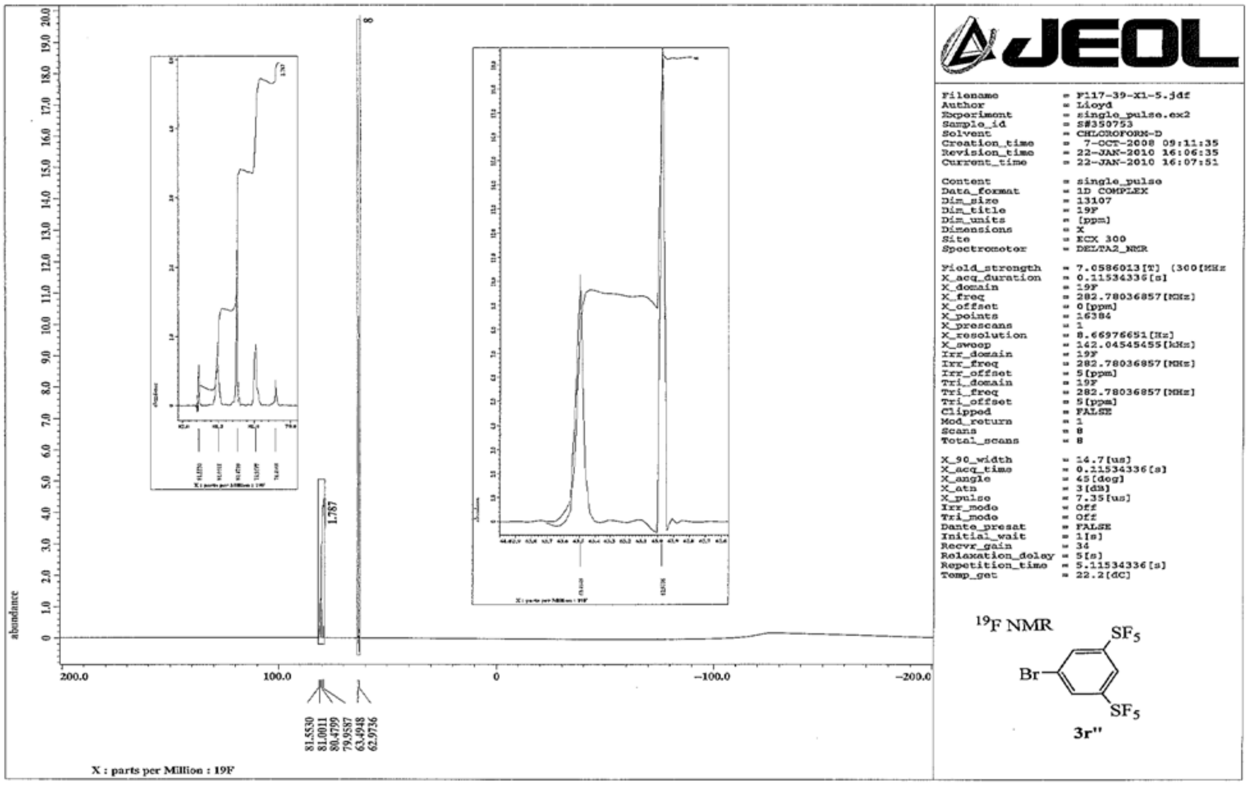
JEOL
Filename = F117-39-X1-5.jdf
Author = Lioyd
Experiment = single_pulse.ex2
Sample_id = S#350753
Solvent = CHLOROFORM-D
Creation_time = 7-OCT-2008 09:11:35
Revision_time = 22-JAN-2010 16:06:35
Current_time = 22-JAN-2010 16:07:51
Content = single_pulse
Data_format = 1D COMPLEX
Dim_size = 13107
Dim_title = 19F
Dim_units = [ppm]
Dimensions = X
Site = ECX 300
Spectrometer = DELTA2_NMR
Field_strength = 7.0586013[T] (300[MHz
X_acq_duration = 0.11534336[s]
X_domain = 19F
X_freq = 282.78036857[MHz]
X_offset = 0[ppm]
X_points = 16384
X_prescans = 1
X_resolution = 8.66976651[Hz]
X_sweep = 142.04545455[kHz]
Irr_domain = 19F
Irr_freq = 282.78036857[MHz]
Irr_offset = 5[ppm]
Tri_domain = 19F
Tri_freq = 282.78036857[MHz]
Tri_offset = 5[ppm]
Clipped = FALSE
Mod_return = 1
Scans = 8
Total_scans = 8
X_90_width = 14.7[us]
X_acq_time = 0.11534336[s]
X_angle = 45[deg]
X_atn = 3[dB]
X_pulse = 7.35[us]
Irr_mode = Off
Tri_mode = Off
Dante_presat = FALSE
Initial_wait = 1[s]
Recvr_gain = 34
Relaxation_delay = 5[s]
Repetition_time = 5.11534336[s]
Temp_get = 22.2[dC]
19F NMR
SF5
Br
SF5
3r''
abundance
1.787
81.5530
81.0011
80.4799
79.9587
63.4948
62.9736
X : parts per Million : 19F

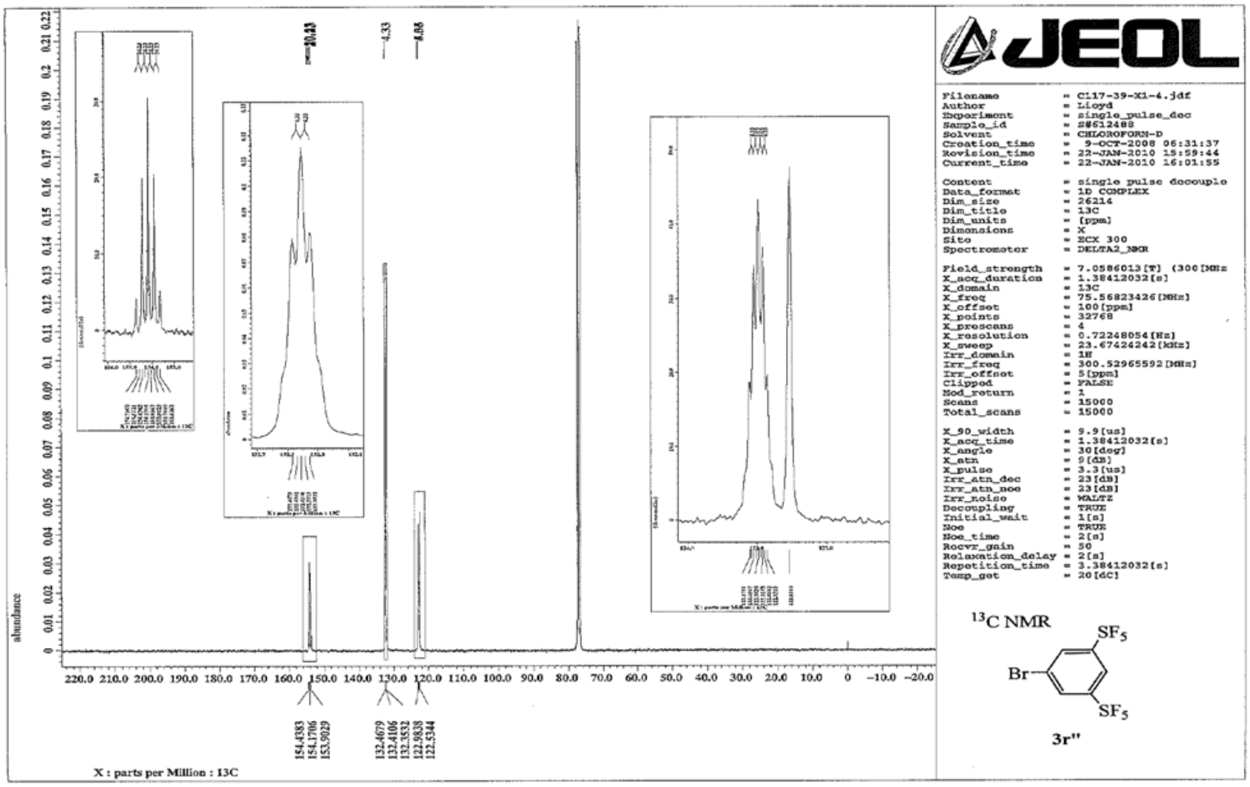
JEOL
Filename = C117-39-X1-4.jdf
Author = Lloyd
Experiment = single_pulse_dec
Sample_id = S#612488
Solvent = CHLOROFORM-D
Creation_time = 9-OCT-2008 06:31:37
Revision_time = 22-JAN-2010 15:59:44
Current_time = 22-JAN-2010 16:01:55
Content = single pulse decouple
Data_format = 1D COMPLEX
Dim_size = 26214
Dim_title = 13C
Dim_units = [ppm]
Dimensions = X
Site = ECX 300
Spectrometer = DELTA2_NMR
Field_strength = 7.0586013[T] (300[MHz
X_acq_duration = 1.38412032[s]
X_domain = 13C
X_freq = 75.56823426[MHz]
X_offset = 100[ppm]
X_points = 32768
X_prescans = 4
X_resolution = 0.72248054[Hz]
X_sweep = 23.67424242[kHz]
Irr_domain = 1H
Irr_freq = 300.52965592[MHz]
Irr_offset = 5[ppm]
Clipped = FALSE
Mod_return = 1
Scans = 15000
Total_scans = 15000
X_90_width = 9.9[us]
X_acq_time = 1.38412032[s]
X_angle = 30[deg]
X_atn = 9[dB]
X_pulse = 3.3[us]
Irr_atn_dec = 23[dB]
Irr_atn_noe = 23[dB]
Irr_noise = WALTZ
Decoupling = TRUE
Initial_wait = 1[s]
Noe = TRUE
Noe_time = 2[s]
Recvr_gain = 50
Relaxation_delay = 2[s]
Repetition_time = 3.38412032[s]
Temp_get = 20[dC]
13C NMR
SF5
Br
SF5
3r''
abundance
154.4383
154.1706
153.9029
132.4679
132.4106
132.3532
122.9838
122.5344
X : parts per Million : 13C

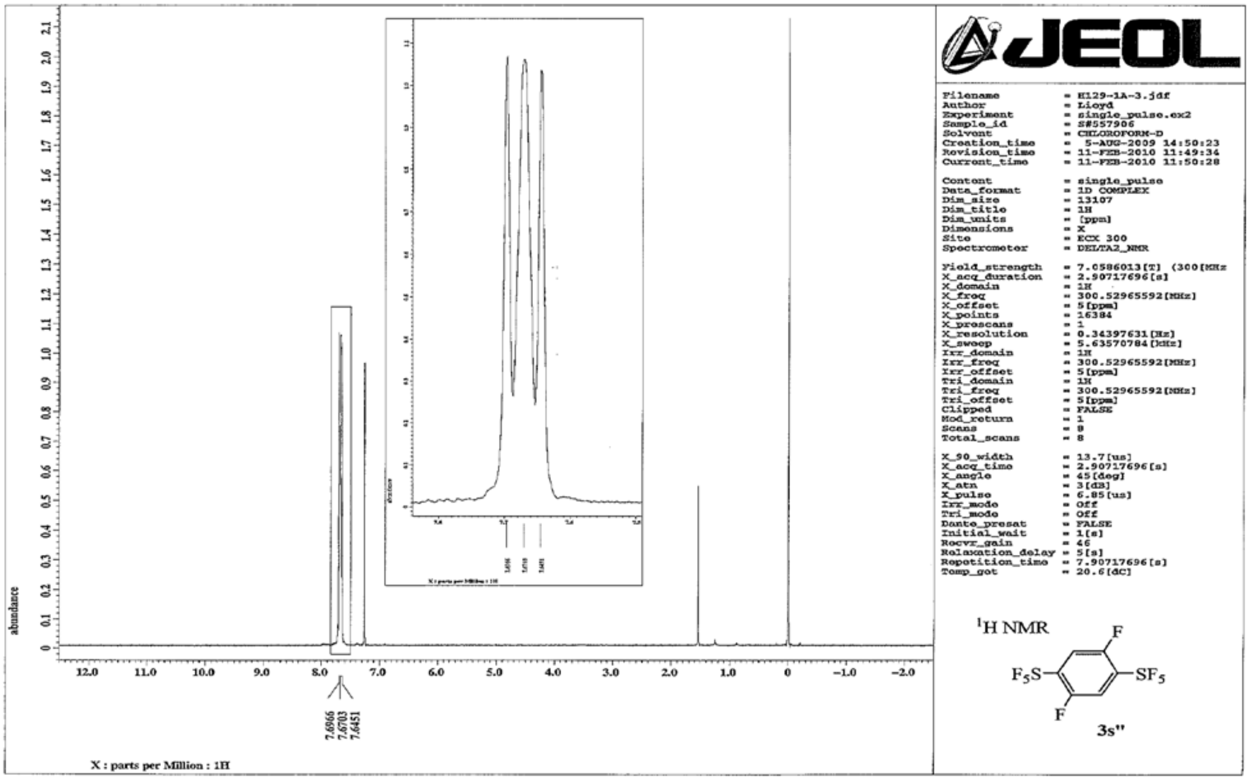
JEOL
Filename = H129-1A-3.jdf
Author = Lloyd
Experiment = single_pulse.ex2
Sample_id = S#557906
Solvent = CHLOROFORM-D
Creation_time = 5-AUG-2009 14:50:23
Revision_time = 11-FEB-2010 11:49:34
Current_time = 11-FEB-2010 11:50:28
Content = single_pulse
Data_format = 1D COMPLEX
Dim_size = 13107
Dim_title = 1H
Dim_units = [ppm]
Dimensions = X
Site = ECX 300
Spectrometer = DELTA2_NMR
Field_strength = 7.0586013[T] (300[MHz
X_acq_duration = 2.90717696[s]
X_domain = 1H
X_freq = 300.52965592[MHz]
X_offset = 5[ppm]
X_points = 16384
X_prescans = 1
X_resolution = 0.34397631[Hz]
X_sweep = 5.63570784[kHz]
Irr_domain = 1H
Irr_freq = 300.52965592[MHz]
Irr_offset = 5[ppm]
Tri_domain = 1H
Tri_freq = 300.52965592[MHz]
Tri_offset = 5[ppm]
Clipped = FALSE
Mod_return = 1
Scans = 8
Total_scans = 8
X_90_width = 13.7[us]
X_acq_time = 2.90717696[s]
X_angle = 45[deg]
X_atn = 3[dB]
X_pulse = 6.85[us]
Irr_mode = Off
Tri_mode = Off
Dante_presat = FALSE
Initial_wait = 1[s]
Recvr_gain = 46
Relaxation_delay = 5[s]
Repetition_time = 7.90717696[s]
Temp_get = 20.6[dC]
1H NMR
F
F5S
SF5
F
3s''
abundance
7.6966
7.6703
7.6451
X : parts per Million : 1H

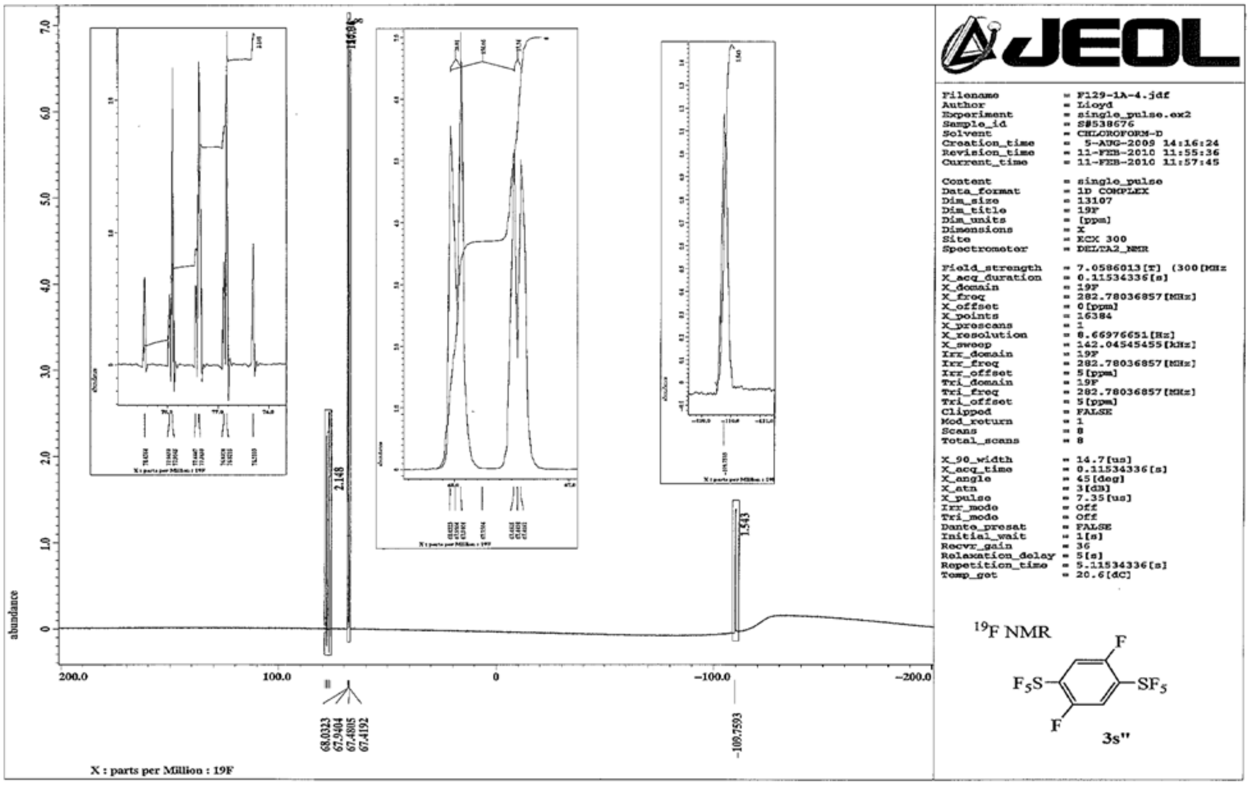

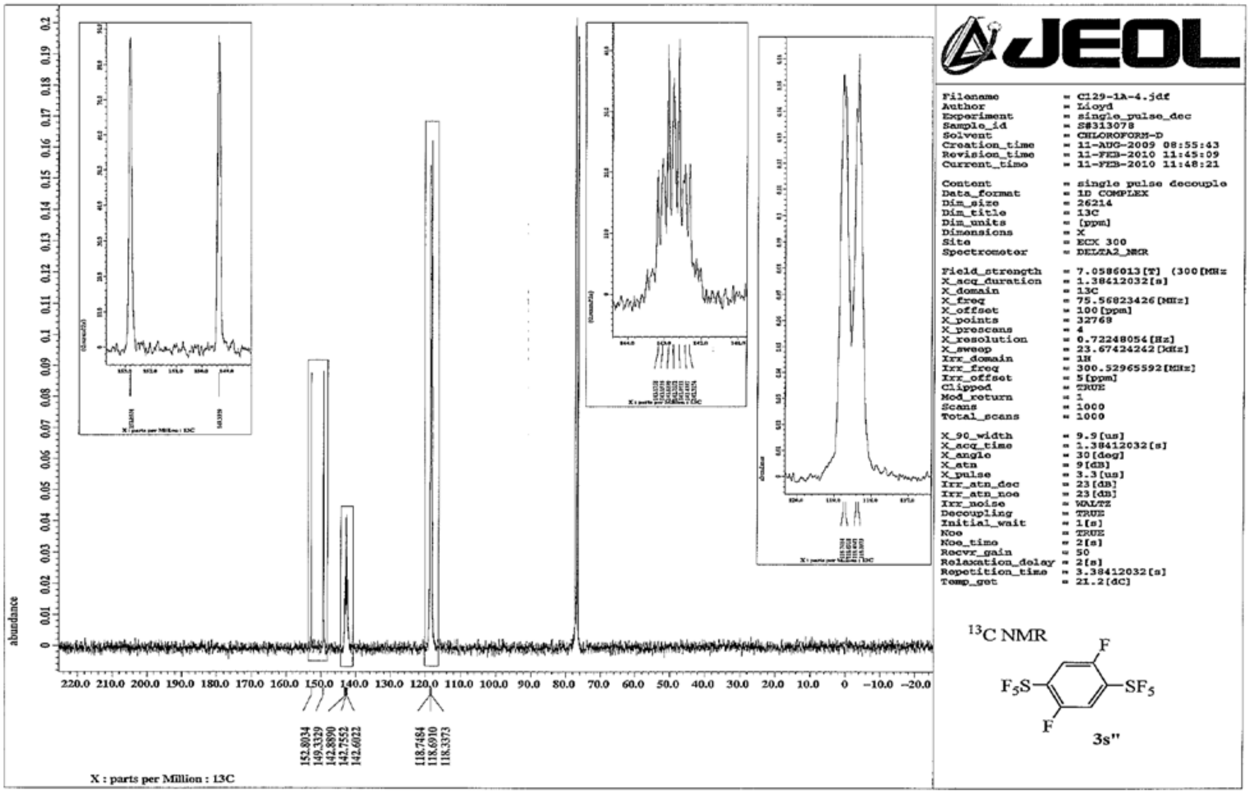

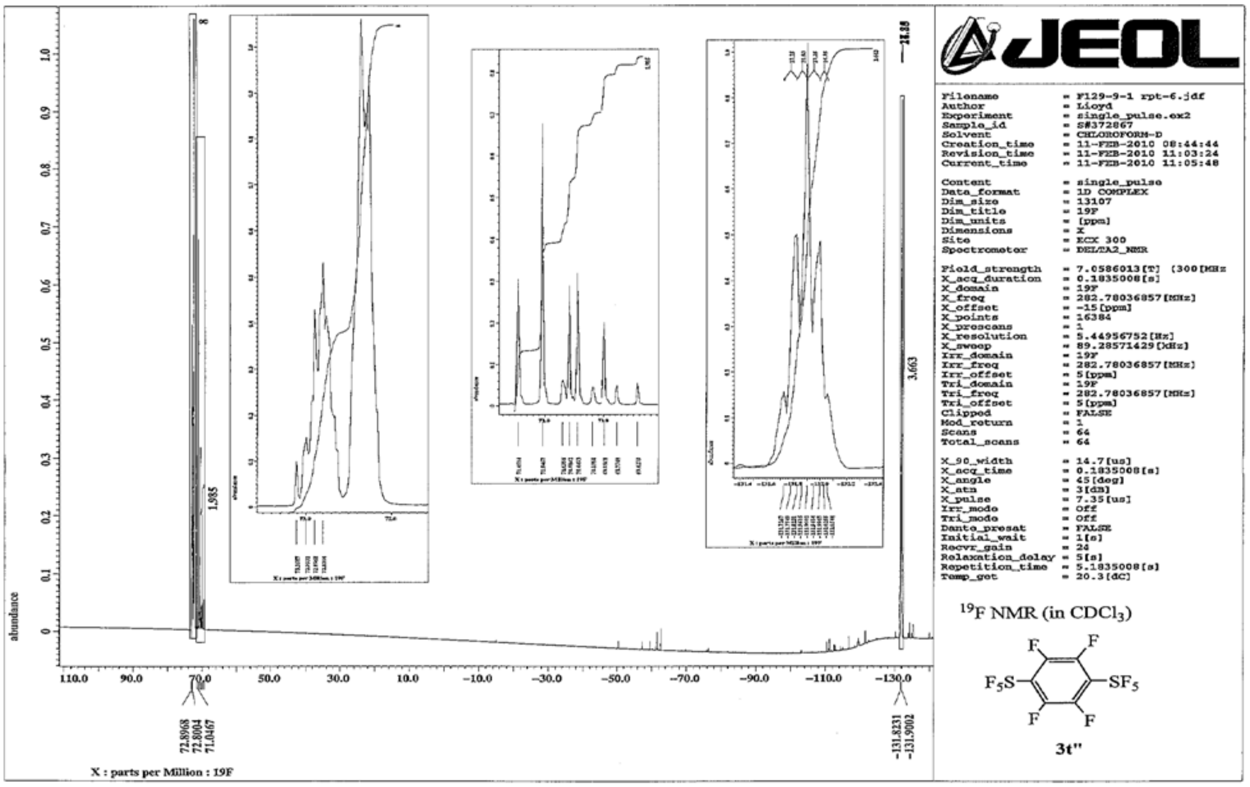
JEOL
Filename = F129-9-1 rpt-6.jdf
Author = Lloyd
Experiment = single_pulse.ex2
Sample_id = S#372867
Solvent = CHLOROFORM-D
Creation_time = 11-FEB-2010 08:44:44
Revision_time = 11-FEB-2010 11:03:24
Current_time = 11-FEB-2010 11:05:48
Content = single_pulse
Data_format = 1D COMPLEX
Dim_size = 13107
Dim_title = 19F
Dim_units = [ppm]
Dimensions = X
Site = ECX 300
Spectrometer = DELTA2_NMR
Field_strength = 7.0586013[T] (300[MHz
X_acq_duration = 0.1835008[s]
X_domain = 19F
X_freq = 282.78036857[MHz]
X_offset = -15[ppm]
X_points = 16384
X_prescans = 1
X_resolution = 5.44956752[Hz]
X_sweep = 89.28571429[kHz]
Irr_domain = 19F
Irr_freq = 282.78036857[MHz]
Irr_offset = 5[ppm]
Tri_domain = 19F
Tri_freq = 282.78036857[MHz]
Tri_offset = 5[ppm]
Clipped = FALSE
Mod_return = 1
Scans = 64
Total_scans = 64
X_90_width = 14.7[us]
X_acq_time = 0.1835008[s]
X_angle = 45[deg]
X_atn = 3[dB]
X_pulse = 7.35[us]
Irr_mode = Off
Tri_mode = Off
Dante_presat = FALSE
Initial_wait = 1[s]
Recvr_gain = 24
Relaxation_delay = 5[s]
Repetition_time = 5.1835008[s]
Temp_get = 20.3[dC]
abundance
1.985
3.663
72.8968
72.8004
71.0467
-131.8231
-131.9002
X : parts per Million : 19F
^{19}F NMR (in $CDCl_3$)
F
F
F_5S
SF_5
F
F
3t''

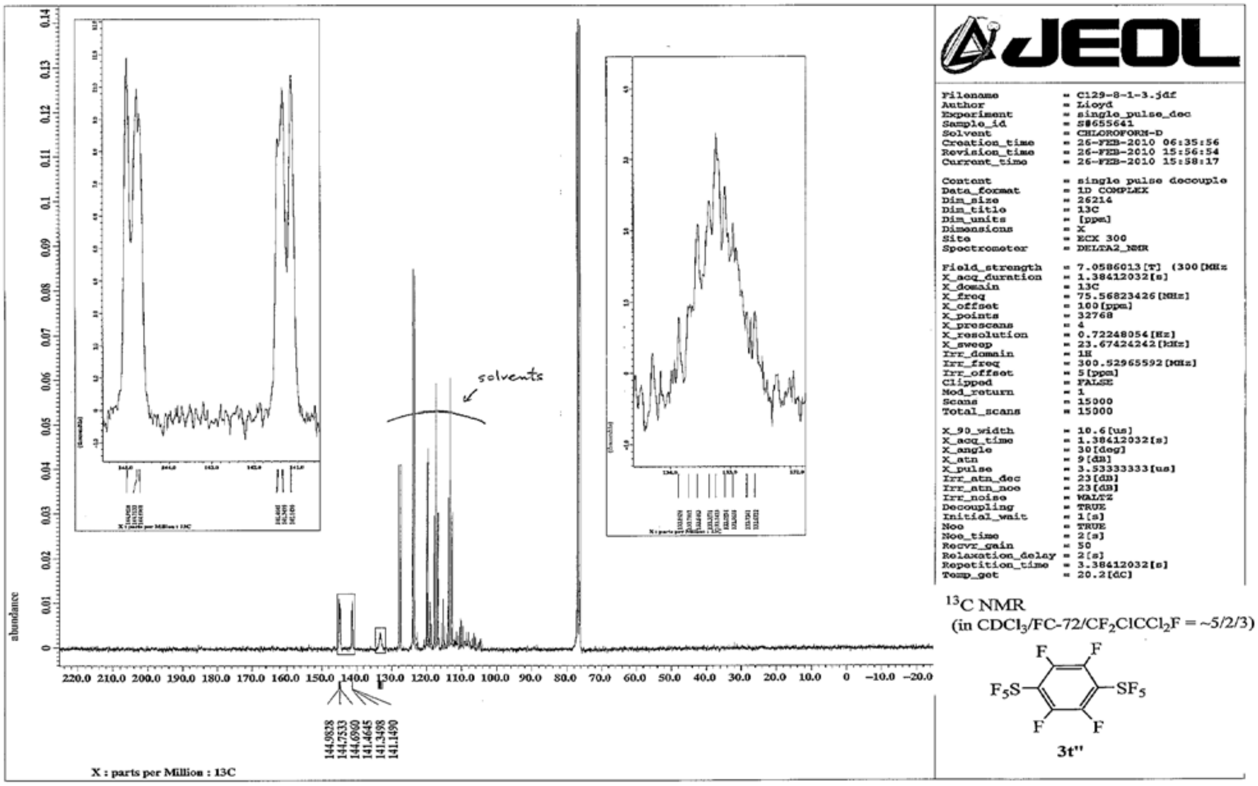
JEOL
Filename = C129-8-1-3.jdf
Author = Lloyd
Experiment = single_pulse_dec
Sample_id = S#655641
Solvent = CHLOROFORM-D
Creation_time = 26-FEB-2010 06:35:56
Revision_time = 26-FEB-2010 15:56:54
Current_time = 26-FEB-2010 15:58:17
Content = single pulse decouple
Data_format = 1D COMPLEX
Dim_size = 26214
Dim_title = 13C
Dim_units = [ppm]
Dimensions = X
Site = ECX 300
Spectrometer = DELTA2_NMR
Field_strength = 7.0586013[T] (300[MHz
X_acq_duration = 1.38412032[s]
X_domain = 13C
X_freq = 75.56823426[MHz]
X_offset = 100[ppm]
X_points = 32768
X_prescans = 4
X_resolution = 0.72248054[Hz]
X_sweep = 23.67424242[kHz]
Irr_domain = 1H
Irr_freq = 300.52965592[MHz]
Irr_offset = 5[ppm]
Clipped = FALSE
Mod_return = 1
Scans = 15000
Total_scans = 15000
X_90_width = 10.6[us]
X_acq_time = 1.38412032[s]
X_angle = 30[deg]
X_atn = 9[dB]
X_pulse = 3.53333333[us]
Irr_atn_dec = 23[dB]
Irr_atn_noe = 23[dB]
Irr_noise = WALTZ
Decoupling = TRUE
Initial_wait = 1[s]
Noe = TRUE
Noe_time = 2[s]
Recvr_gain = 50
Relaxation_delay = 2[s]
Repetition_time = 3.38412032[s]
Temp_get = 20.2[dC]
solvents
abundance
144.9828
144.7533
144.6960
141.4645
141.3498
141.1490
X : parts per Million : 13C
^{13}C NMR
(in $CDCl_3$/FC-72/CF_2ClCCl_2F = ~5/2/3)
F
F
F_5S
SF_5
F
F
3t''
